# Gene expression responses to anti-tuberculous drugs in a whole blood model

**DOI:** 10.1186/s12866-020-01766-y

**Published:** 2020-04-07

**Authors:** Philip Kam Weng Kwan, Wenwei Lin, Ahmad Nazri Mohamed Naim, Balamurugan Periaswamy, Paola Florez De Sessions, Martin L. Hibberd, Nicholas I. Paton

**Affiliations:** 1 Department of Medicine, Yong Loo Lin School of Medicine, National University of Singapore, NUHS Tower Block Level 10, 1E Kent Ridge Road, Singapore, 119228 Singapore; 2 Genome Institute of Singapore, Agency for Science, Technology and Research, Singapore, Singapore; 3 London School of Hygiene & Tropical Medicine, London, UK; 4 Department of Microbiology and Immunology, Yong Loo Lin School of Medicine, National University of Singapore, Singapore, Singapore

**Keywords:** Tuberculosis, Gene expression, Transcriptome, Mechanism of action, Anti-tuberculosis drugs, Whole blood assay, Microarray, Faropenem

## Abstract

**Background:**

There is a need for better tools to evaluate new or repurposed TB drugs. The whole blood bactericidal activity (WBA) assay has been advocated for this purpose. We investigated whether transcriptional responses in the WBA assay resemble TB responses in vivo, and whether the approach might additionally reveal mechanisms of action.

**Results:**

1422 of 1798 (79%) of differentially expressed genes in WBA incubated with the standard combination of rifampicin, isoniazid, pyrazinamide and ethambutol were also expressed in sputum (*P* < 0.0001) obtained from patients taking the same combination of drugs; these comprised well-established treatment-response genes. Gene expression profiles in WBA incubated with the standard drugs individually, or with moxifloxacin or faropenem (with amoxicillin and clavulanic acid) clustered by individual drug exposure. Distinct pathways were detected for individual drugs, although only with isoniazid did these relate to known mechanisms of drug action.

**Conclusions:**

Substantial agreement between whole blood cultures and sputum and the ability to differentiate individual drugs suggest that transcriptomics may add value to the whole blood assay for evaluating new TB drugs.

## Background

Previous studies have examined the effect of anti-tuberculous drugs on the *Mycobacterium tuberculosis* (*Mtb*) transcriptome in sputum [1, 2], broth cultures [3–5], and a macrophage model [4]. Mtb transcriptional profiling has been shown to be able to decipher drug mechanism of action, such as genes related to mycolic acid and fatty acid biosynthesis (FAS-II) when exposed to isoniazid [3–5]. One small longitudinal study delineated the changes in gene expression in the sputum during the course of standard tuberculosis (TB) treatment [1, 2]. It is possible that this approach may add value to traditional biomarker outcomes that are known to have limited value in predicting sterilizing activity in TB [6, 7]. However, further work is needed to understand transcriptome responses to individual drugs and drug combinations and whether they have ability to differentiate between drugs.

The whole blood bactericidal activity (WBA) model has been used as an early clinical screening platform for novel TB drugs/regimens prior to testing in clinical trials. It is an ex vivo model that measures the bactericidal activity of one or more drugs combined with host immune responses. This has been applied to testing a variety of established and novel antibacterial drugs and, more recently, putative host-directed therapies [8–15].

The objectives of this study were to determine if Mtb transcriptome responses in the WBA model could reflect transcriptome responses in TB patients on combination treatment and whether this approach could identify unique transcriptome responses associated with individual anti-tuberculous drugs**.**

## Results

There was evidence of strong bactericidal activity in the whole blood assay with isoniazid, rifampicin, and moxifloxacin tested individually and with isoniazid, rifampicin, pyrazinamide and ethambutol tested in combination (− 1.62, − 2.74, − 1.92 and 3.49 Δlog CFU respectively), whereas pyrazinamide, ethambutol and faropenem used individually had no substantive evidence of bactericidal activity over the 72-h incubation period (Supplementary Figure 1).

In the whole blood assays set-up for gene expression analysis, microarray signals were obtained from all experimental conditions and no sample outliers were detected after RNA normalization.

Compared to drug-free broth cultures, there were 1755 differentially expressed genes (DEG) in drug-free whole blood cultures;1798 DEG in whole-blood cultures containing the standard 4-drug combination (1541, 86% overlapping with drug-free whole blood cultures); 1701 DEG in sputum from TB patients taking the same standard combination (Supplementary Table 1 and 2 respectively). Of the 1798 DEG in whole-blood cultures with standard drugs, 1422 (79%) were also expressed in sputum (*p* < 0.00001), all but one in the same direction in the two assay conditions (267 up- and 1154 down-regulated); of these overlap genes, 1317 (90%) were also expressed in the whole blood cultures without drugs. The DEG in whole blood cultures with standard drugs included genes from pathways known to be affected by standard TB treatment, such as DosR and KstR regulon genes upregulated, and multiple ATP synthesis, fatty acid synthesis genes downregulated (Table 1) [1, 2]. The 376 DEG in the whole blood cultures that were not found in sputum, and the 279 DEG in sputum but not found in the whole blood cultures had similar Gene Ontology Molecular pathways; the majority (70–71%) were associated with the catalytic activity pathway (Supplementary Table 3).

**Table 1:** Differentially expressed genes in whole blood assay with standard combination treatment that are found in known TB pathways (as reported in Honeyborne et al) [1]

Gene pathway	DEGs found in whole blood assay with standard combination treatment	Direction of differential expression
DosR regulon	nrdZ	Up
KstR regulon	Rv1628c	Up
Triacylglycerol synthesis	Rv3087	Up
Aerobic respiration	cta/C/D/E; nuoD/G/K; qcRA/B/C	Down
ATP synthesis	atpA/B/C/D/E/F/G	Down
Citric acid cycle	gltA2; Rv0247c; Rv0248c; sucC	Down
Fatty acid synthesis	fabD; fabG1; fas; kasA/B	Down
Mycolic acid biosynthesis	cmaA2; fadD32; mmaA3/A4; pcaA; korA	Down
Ribosome biosynthesis	rplx11; rpmX5; rpoA/B/C; rpsAx13	Down

**Fig. 1:**
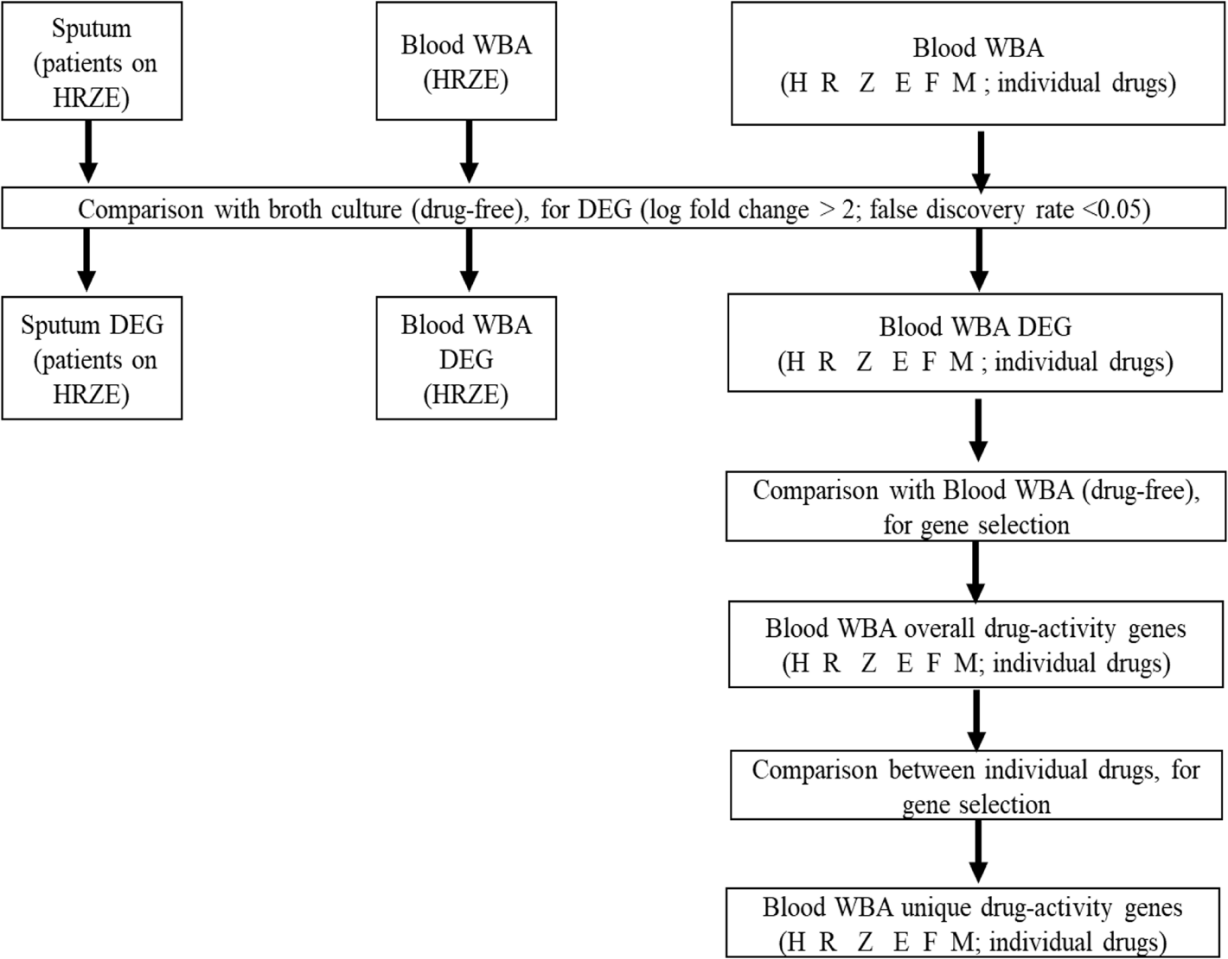
Flow chart for transcriptional analyses. DEG: Differentially expressed genes. HZRE: combination of four standard anti-tuberculosis drugs isoniazid, pyrazinamide, rifampicin and ethambutol

**Fig. 2:**
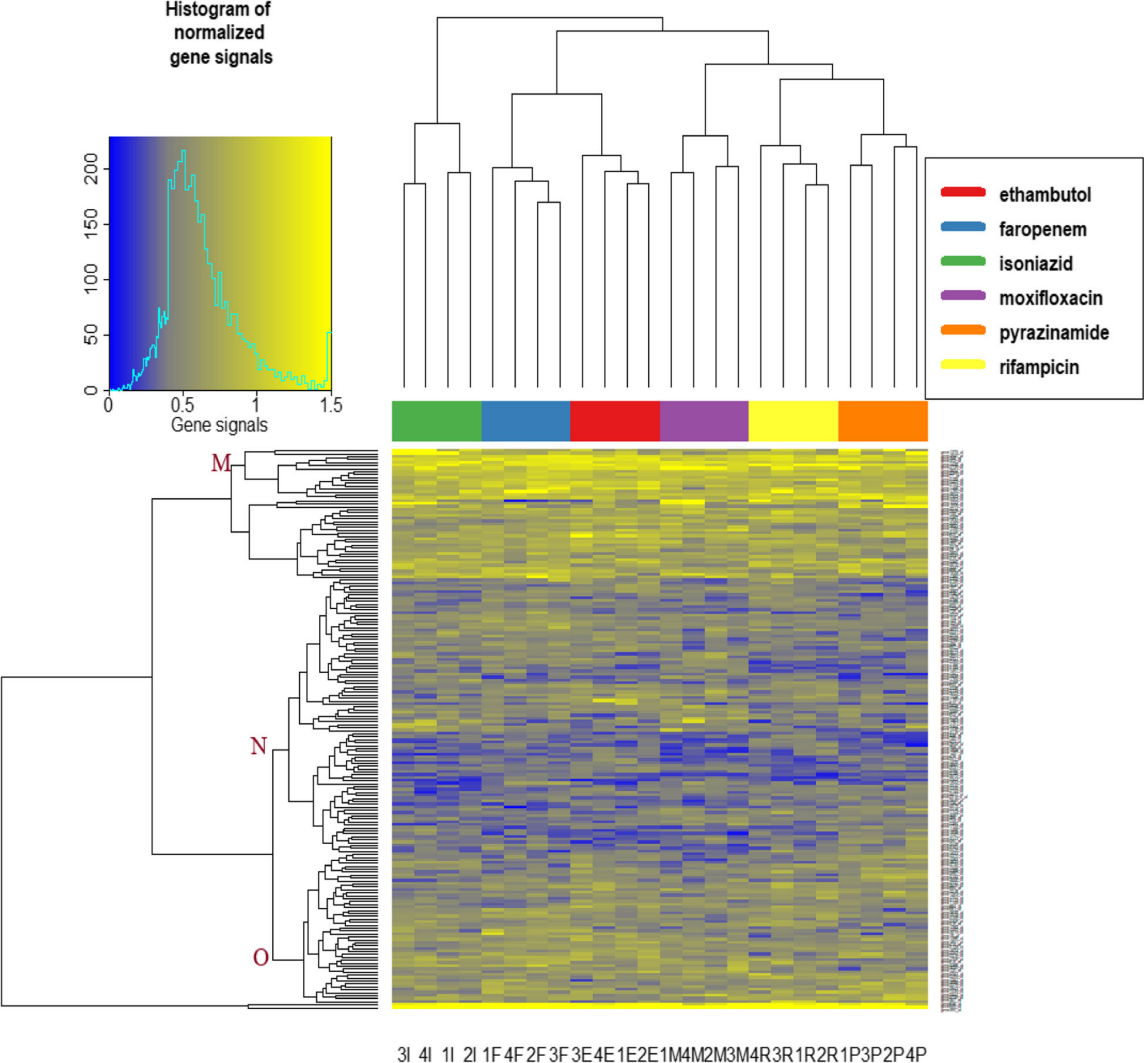
Heatmap of gene profiles obtained from cultures with individual anti-TB drugs. The dendrograms are generated by hierarchical clustering using Euclidean distance and complete linkage method. Each column represents a separate whole blood assay (*n* = 24; 6 individual drugs, each tested with blood from 4 individual volunteers); each row corresponds to a gene (*n* = 190) that was found to differ between two or more groups of cultures with individual drugs. The columns are labelled at the bottom according to patient number (1–4) and antibiotic in the culture (by first letter). The antibiotic in each assay is also shown by the colour coding at the top of the column, clearly demonstrating that there is perfect clustering by antibiotic used. Clusters of genes with identifiable pathways are shown as “M” (cell membrane or transmembrane processes; 17 genes), “N” (nucleotide binding, ATP binding, and lipid metabolism; 93 genes); “O” (oxidoreductase; 51 genes)

Analysis of the gene expression profiles obtained from cultures performed with the six individual drugs found no evidence of clustering by volunteer blood sample (AU ≥ 95% for clustering by individual antibiotics; Fig. 1; Supplementary Figure 2). Analysis of the gene clusters identified several clusters that were associated with pathways related to common modes of drug action (membrane-related processes; nucleotide-binding, ATP-binding and lipid metabolism; and oxidoreductase; Fig. 2).

**Fig. 3:**
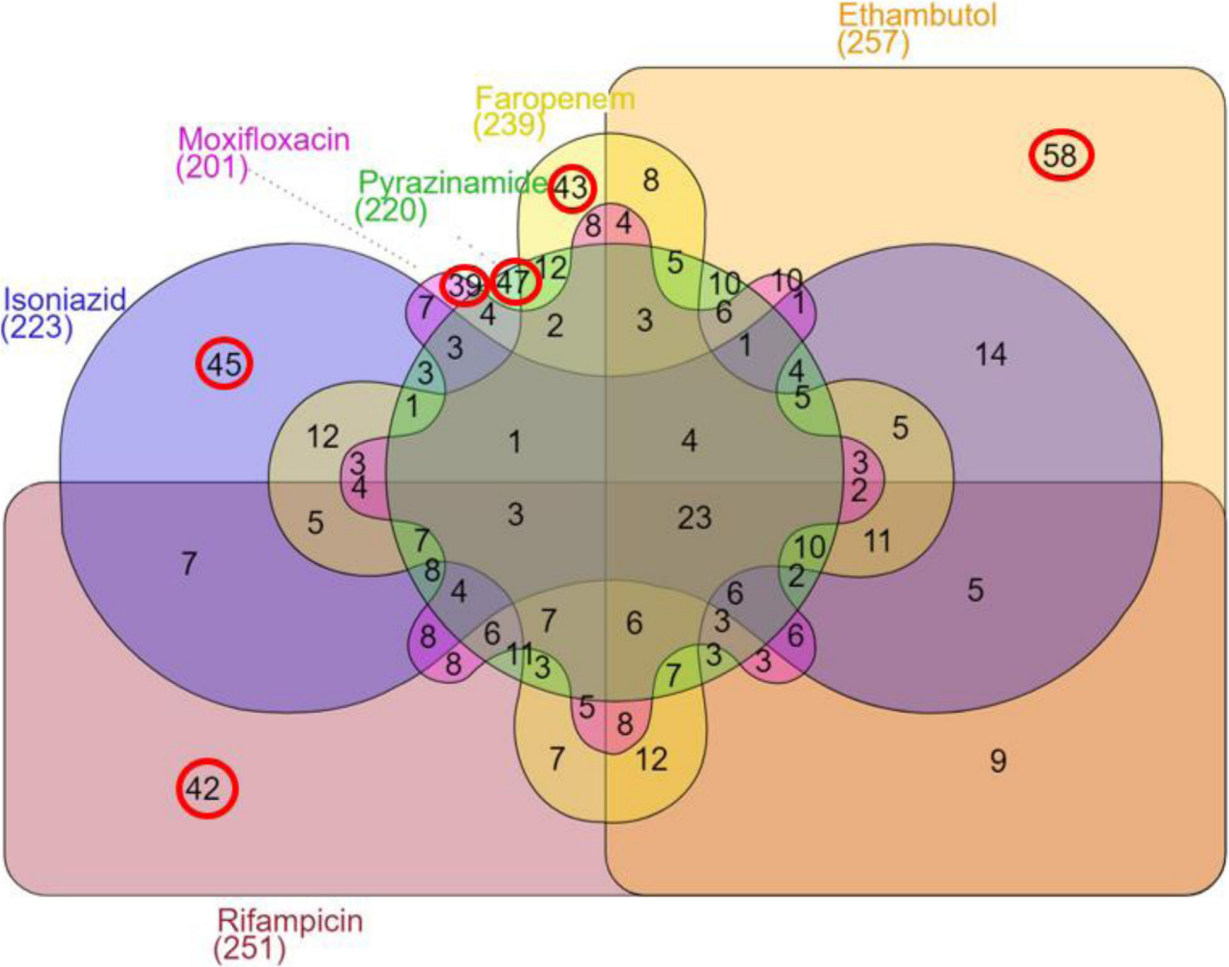
Numbers of differentially expressed genes in the overall drug-related gene expression profile (in parentheses after drug name) and in the unique drug-related gene expression profile (in circles within the set for each drug)

Analysis of DEG in whole blood cultures with individual drugs but not in the drug-free cultures (both relative to broth cultures), found genes that were that were both unique for the specific drugs and genes that overlapped with other individual drugs (Fig. 3). The overall drug-related gene expression profile (including the genes that were differentially expressed with other drugs) for each of the six individual drug whole-blood cultures had a median of 239 (range 201 to 251) differentially expressed genes (Fig. 3; Supplementary Table 4). The unique drug-related gene expression profile (excluding the genes that were differentially expressed with other drugs) for each individual drug had a median of 44 (range 39 to 58) DEGs (Fig. 3; Supplementary Table 5).

Analysis of pathways associated with the overall drug-related gene expression profile identified significantly enriched pathways for all individual drugs (Supplementary Table 6). Of the 19 significant pathways identified for isoniazid cultures, 8 were related to the known mechanisms of action of isoniazid including fatty-acyl-CoA binding, flavin adenine dinucleotide and acyl-CoA dehydrogenase activity. None of the pathways identified with other drugs appeared to be associated with known mechanisms of drug action. Analysis of pathways using the unique drug-related expression profile identified pathways for all individual drugs except faropenem and moxifloxacin; Supplementary Table 7). Of the 6 pathways identified with isoniazid, 4 were related to known mechanism of action (all were found in the 8 pathways identified with the overall drug-related gene-expression profile). Again, none of the pathways with the other drugs were plausibly related to mechanism of action.

Analysis of individual genes from the unique drug-related expression profiles identified genes directly associated with mechanism of actions of isoniazid (e.g. mycolic acid biosynthesis pathway), rifampicin (ribonucleoside binding); or indirectly related, ethambutol (membrane components) [16]; moxifloxacin (DNA-binding, *gyrA/B)* [17]; pyrazinamide (active form of pyrazinoic acid, PanD) [18] and faropenem (beta-lactam-targeted efflux pumps; Supplementary Table 8) [19].

## Discussion

Our finding that the gene expression signature from the combination of standard TB drugs (rifampicin, isoniazid, pyrazinamide and ethambutol) in whole blood culture was similar to the in vivo gene expression profile in sputum obtained from patients taking the same standard combination of drugs for a period of one to two weeks (and with the profile obtained in another study of sputum gene expression) [1, 2] lends initial support to the validity of the adapted WBA model to assess transcriptome responses to TB drugs. The advantage of evaluating bacterial transcriptome responses in the whole blood assay is that the assay includes human immune cells and this may account in part for the similarity with the bacterial transcriptome in sputum (where bacilli have also been exposed to the lung immune response) as well as the overlap with the genes expressed in the blood cultures without drugs (with all conditions differing qualitatively from cell-free broth culture). Furthermore, the DEG in whole blood culture included changes in pathways known to be affected by combination TB therapy, including those that are suppressed (e.g. aerobic respiration, citric acid cycle and ATP synthesis) and those that are induced (e.g. DosR) [1, 2]. This suggests that Mtb also responses to drugs in the whole blood environment by reducing energy levels and activating metabolic pathways associated with anaerobic conditions (e.g. DosR regulon) [20] to survive. Some differences in gene expression profile would be expected from the different assay conditions, including the difference in immune milieu, but even the genes that were unique between sputum and whole blood still shared similar molecular pathways. We cannot say whether the degree of overlap with sputum DEG is any greater than we might have seen had we done the experiment with standard TB drugs in broth culture rather than the WBA model, although the immune contribution (above) suggests that it may be; no published studies have compared gene expression responses to standard TB drugs in both broth and sputum. One study comparing the gene expression response to isoniazid alone in TB macrophage culture versus broth culture found only 40% overlap [4]. Whether the theoretical advantages of the WBA model outlined above carry any advantages for gene expression profiling over simpler culture methods will need further study.

The finding that the transcriptome profile achieved separation between individual anti-TB drugs indicates that it is responding uniquely to the drug environment in the whole blood assay and this may represent a useful new indicator of activity in this assay paradigm. This unique expression was present even for moxifloxacin that appeared to have limited activity at the first hour of incubation, and for pyrazinamide, ethambutol, and faropenem, which appeared to have minimal detectable bactericidal activity even during the full 72-h incubation period of the assay. The limited bactericidal activity of these drugs has been previously noted in the WBA paradigm; for pyrazinamide it may perhaps reflect the requirement for a hypoxic environment for activity [8, 11, 21, 22]; for faropenem it may reflect rapid degradation of the drug in whole blood during the course of incubation or that higher doses are needed for activity [9]. The adaptation of the WBA model to provide a readout of transcriptome change rather than a readout of bactericidal activity may in part overcome the limitations of the traditional WBA assay for detecting drug activity. A study analyzing transcriptomics with 74 novel or known antibacterial agents in broth cultures also demonstrated clear differences between drugs of similar classes [3]. Several studies have suggested that using a minimal number of Mtb genes in such an approach might facilitate routine drug high throughput screening [5, 23]. We included faropenem, a non-standard TB drug, in this study because previous data showing minimal activity of this drug in a WBA assay [9], contrasts with the substantial body of emerging data on the value of beta-lactams for anti-TB treatment in vivo [24, 25]. However, there is no certainty that the detection of a change in bacterial transcriptome, with faropenem or any other drug, would necessarily translate into substantive bactericidal or sterilizing activity in vivo.

Although we found some associations between the gene clusters and pathways known to be relevant to action of one or more of the antibiotics investigated, we found only limited evidence that the whole blood transcriptome model could identify the specific mechanism of action of anti-TB drugs (with the exception of isoniazid, where we found evidence of characteristic pathways as previously shown in broth cultures [3], and in microarray studies that have demonstrated isoniazid-specific gene responses from the fatty acid synthesis II (FAS-II) and mycolic acid synthesis-related pathways) [26, 27].

The limited ability of this assay to detect gene expression changes related to known mechanisms of action of the individual drugs may reflect differences in the mechanism of action according to the environment to which the bacteria are exposed. More definitive transcriptome changes were seen in a previous study with drug exposure in broth cultures [3], and it is possible that the additional presence of immune cells in the WBA paradigm modifies the response to drugs. We have demonstrated that neutrophils significantly alter the bactericidal activity of rifampicin in the WBA paradigm, possibly by intracellular bacterial sequestration [28]. In an ex vivo cellular model, the response to drugs was more characteristic of host responses than of specific effects on the primary drug targets [4].

One of the limitations of our adapted model is that we standardized to a single (1-h) incubation time. The duration of the incubation period is likely of paramount importance, as there needs to be sufficient time for the drug to have an influence on bacterial gene expression whilst not allowing for prolonged exposure and significant bacterial killing that might reduce the amount of bacterial RNA below a critical threshold for analysis (our blood volumes per assay were relatively small and already at or close to the threshold for RNA detection). The optimal time could differ between drugs, but we preferred to standardize to a single incubation time so that other factors that might influence gene expression in the assay (for instance immune-mediated bacterial killing) would be better controlled to enable us to compare more directly the transcriptome changes associated with the individual drugs. From a practical perspective, we also wanted to develop a standardized assay paradigm that might easily be translated to testing future drugs, without requiring the cumbersome step of performing multiple time-kill curves to optimize the paradigm prior to testing a new drug. Performing repeated transcriptome analysis at multiple incubation timepoints for each drug would be practically (volume of blood required for assays) and economically infeasible. It is possible that the absence of signature transcriptome changes that align directly with known mechanism of actions of the drugs (except for isoniazid) arises from inadequate exposure time to these less cidal drugs and that we might have seen more characteristic changes with a longer incubation period. Nevertheless, we found uniquely expressed genes for each of the drugs, which indicates that the exposure was long enough for the bacteria to recognize and respond to the drug challenge.

## Conclusions

In summary, we have shown that an alternative readout – change in bacterial transcriptome – can be obtained from an adapted WBA paradigm and that this responds in a similar way to the bacterial transcriptome in sputum when exposed to standard 4-drug combination therapy. The method detects gene expression responses to anti-tuberculosis drugs, even to drugs that show no overt bacterial killing in the WBA assay. However, the adapted method may have limited value in identifying pathways associated with the mechanism of action of individual drugs, although this may perhaps be overcome with further modifications to the assay such as drug-specific incubation times which remain to be explored.

## Methods

### Drug bactericidal curves in the whole blood assay

Initial experiments were performed to determine the bactericidal curves of individual drugs for the purposes of selecting a standard time point for later transcriptomic studies. For these studies, 50 ml of whole blood was drawn into heparin tubes from each of three healthy (without current acute or chronic illness) adult volunteers.

The whole blood assay was performed and calculated as previously described [22]. Briefly, the Mtb H37Rv lab strain was grown in 7H9 medium containing 0.2% glycerol, 0.05% tween 80 and ADC supplement (sodium chloride, Bovine Serum Albumin Fraction V, anhydrous Dextrose and Catalase) to mid-log phase, and aliquots were taken and frozen in glycerol at − 80 °C. Volumes of this stock were then serially diluted and each inoculated into a Mycobacteria Growth Indicator Tube (MGIT) tubes to determine the relationship between inoculum volume and time-to-positivity (TTP). The specific volume of stock that gave a TTP of 5.5 days was identified (containing ~ 10^5^ CFU/ml) and this was used as the standard inoculum in all experiments.

Whole-blood cultures were prepared by mixing 300ul heparinized blood with one of the study drugs and made up to a total volume of 600ul by adding RPMI 1640 culture medium with 25 mmol/L N-2-hydroxyethylpiperazine-N′-2 ethane sulfonic acid). These were incubated for 30 min at room temperature. Study drug was then added in the amount required to achieve a final concentration based on the published values of Cmax in pharmacokinetic studies performed with standard clinical doses: rifampicin 10 μg/ml [29, 30]; isoniazid 5 μg/ml [29]; pyrazinamide 25 μg/ml [29]; ethambutol 5 μg/ml [29]; moxifloxacin 4 μg/ml [29, 31, 32]; and faropenem sodium (Farobact, Cipla, Mumbai), amoxicillin and clavulanic acid (5, 10, and 3 μg/ml respectively). Eight separate cultures were set-up with individual rifampicin, isoniazid, pyrazinamide, ethambutol and moxifloxacin; faropenem (with amoxicillin and clavulanic acid); the combination of rifampicin, isoniazid, pyrazinamide and ethambutol; and a drug-free control culture with 0.9% dimethyl sulfoxide added (DMSO; control for the purpose of comparing the bactericidal curves). The standard volume of Mtb stock (identified as above), was added to all cultures and incubated at 37 °C with slow constant mixing. From each of the three 50 ml blood samples, 154 cultures were set-up (9 incubation times for each of 8 drug conditions, each in duplicate).

At pre-specified Mtb incubation time-points (0.5, 1, 1.5, 2, 4, 6, 24, 48, 72 h), duplicate cultures were centrifuged; blood cells lysed, pellets washed; and resuspended into mycobacterial growth inhibition tubes (MGITs) and incubated using the MGIT960 system (Becton Dickinson, Franklin Lakes, USA). The time to positivity (TTP) was recorded. Control cultures (for WBA calculations) were set-up by inoculating the standard volume of Mtb stock into MGIT tubes on the same day as the preparation of whole blood cultures.

The WBA for the samples obtained for individual drug/incubation times was calculated from the difference between the log of the volume on the standard curve that corresponded to the TTP for that sample and log of the volume corresponding to the TTP of the control culture. This is equivalent to the difference in log of bacterial colonies forming units (CFU) between the sample and the control, reported as Δ log CFU. Drugs with higher bactericidal activity have a larger change in log CFU over time (lower negative values), whereas drugs with little or no activity (bacteriostatic) have values close to zero.

An incubation time of 1 h was selected for the subsequent experiments based on the average time needed to reduce the initial inoculum by one log CFU in blood cultures containing isoniazid and rifampicin (approximately 0.5 and 1.5 h respectively), with the assumption that this would give sufficient time for drugs to demonstrate effects on gene expression whilst minimizing the reduction of viable cells.

### Gene expression profiling from the whole blood cultures

A 70 ml sample of whole blood was drawn from each of 4 healthy volunteers who had no history of chronic disease, no history of TB and who had a negative IGRA.

WBA cultures were set-up as described above, but with a 25-fold increase in volumes (to a total volume of 15 ml blood culture) whilst maintaining the ratios of blood and TB inoculum and the drug concentration unchanged. Cultures for the same eight drug/control conditions as described above were prepared in quadruplicate (each replicate assay prepared separately from the blood of one of the four healthy volunteers) and were incubated for 1 h (time selected from the kill curves, as above).

RNA was extracted from 15 ml whole blood cultures by adding Guanidium Thiocyanate solution for host cell lysis, followed by resuspension of cells in 1 ml TRIzol Reagent (Thermo Fisher Scientific, Massachusetts, USA) for Mtb inactivation, Mtb cells were mechanically lyzed using the FastPrep 24 Tissue Homogenizer (MP Biomedicals, California, USA) at 4x30s, 6.0 m/s followed by phenol-chloroform steps outlined in the TRIzol user manual. Samples were treated with DNAse-I using TURBO DNA-free kit (Thermo Fisher Scientific, Massachusetts, USA).

### Gene expression profiling from broth cultures

The Mtb H37Rv lab strain was grown (O.D.0.5–0.7) as described above, were centrifuged, washed with RNAprotect Cell Reagent (Qiagen, Hilden, Germany) and resuspended with TRIzol Reagent. RNA was extracted from the sputum samples using the same approach as whole blood cultures.

### Gene expression profiling from sputum

A single sputum sample was collected from each of 9 adult patients with drug-susceptible pulmonary TB, who were HIV-negative and without other serious chronic diseases. All cases were confirmed by culture and isolates were demonstrated to be susceptible to streptomycin, isoniazid, rifampicin and ethambutol. Five of the patients had cavitation on chest X-ray. Patients had received a mean of 9 ± 4 days standard anti-tuberculous chemotherapy (rifampicin, isoniazid, pyrazinamide and ethambutol) at the time the sputum sample was obtained. RNA was extracted from the sputum samples using the same approach as whole blood cultures.

### Microarray and functional annotation

Total RNA yield was quantified using Bioanalyzer (Agilent Technologies, California, USA) and the presence of Mtb RNA was verified by RT-qPCR using Taqman probes and primers targeting mycobacteria species’ 16 s ribosomal RNA (primer sequences obtained from a previous study) [33]. The RNA samples were prepared for microarray hybridization using the Affymetrix 3′ Pico IVT sample preparation kit (Thermo Scientific, Massachusetts, USA).

Microarray signals were generated using a custom Affymetrix Mtb chip (MTubEX1; Thermofisher scientific, Massachusetts, USA) that was designed with signal processing algorithms specific for Mtb H37Rv genes and read using GeneChip® Scanner. Signal processing and normalization was done by Robust Multiarray Analysis (RMA) [34, 35], performed using R software package Affy and Affycoretools.

The overall workflow is depicted in Fig. 1. Analyses of differentially-expressed genes (DEG) in whole blood cultures incubated with the standard rifampicin, isoniazid, pyrazinamide and ethambutol combination and in the sputum of patients taking the same combination (each relative to broth cultures) was performed using the R package Limma [36] (cut-off of log fold change > 2 in either direction and False Discovery Rate < 0.05 by the Benjamini-Hochberg procedure) [36, 37]. The overlap in DEG between blood culture and sputum was tested for significance by the exact hypergeometric probability method. Individual genes were compared to those in established pathways for treatment response in TB, as reported in an earlier study [1, 2].

Gene expression profiles were compared between individual drugs (faropenem counted as an individual drug, although tested in whole blood cultures with amoxicillin and clavulanate for synergy), by applying one-way analysis of variance (ANOVA) to the normalized genes (without removing genes that were differentially expressed in DMSO control cultures versus broth culture) to select genes that differed (*p* < 0.05) between two or more drug-culture groups (each group comprising 4 cultures with an individual drug). Hierarchical clustering was performed based on these selected genes using Euclidean distance and complete linkage, initially based on the gene profiles in individual cultures and subsequently based on individual genes and results were plotted as a heatmap. Pathway enrichment was performed for the gene clusters using Database for Annotation, Visualization and Integrated Discovery (DAVID) software [38]. Clustering analysis was done on the individual cultures samples via pvclust R software package using Approximately Unbiased (AU) *p* values by bootstrapping methods to evaluate individual dendrograms (≥95% for significant clusters) [39].

The *overall drug-related gene expression profile* for each individual drug was determined by identifying genes in whole blood culture that were differentially expressed relative to broth culture for that drug and then removing genes that were also differentially expressed (versus broth culture) in the control cultures with DMSO using the same FDR cut-offs as above. The *unique drug-related gene expression profile* for each of the individual drugs was determined by removing genes that were found in the overall drug-related gene expression profiles of any of the other individual drugs.

The functional aspects of the gene expression profiles were explored by applying pathway enrichment analysis on the overall and unique drug-related gene expression profiles using DAVID software, applying a modified Fisher Exact *P*-value < 0.05 to identify significant pathways. Annotation of genes was performed using Gene Ontology (GO) database. All analyses were done using in-house python or R software scripts.

The study protocols received ethics approval from the Domain Specific Review Board of the National Healthcare Group, Singapore.

## Supplementary information

**Additional file 1.**

## Data Availability

The datasets generated and/or analysed during the current study are available in the Genome Expression Omnibus repository, https://www.ncbi.nlm.nih.gov/geo/query/acc.cgi?acc=GSE142756 .

## Abbreviations

TB: Tuberculosis
WBA: Whole blood bactericidal activity
CFU: Colonies forming units
DEG: Differentially-expressed genes
AU: Approximately unbiased
DMSO: Dimethyl sulfoxide

## References

[1] Honeyborne I, McHugh TD, Kuittinen I, Cichonska A, Evangelopoulos D, Ronacher K (2016). Profiling persistent tubercule bacilli from patient sputa during therapy predicts early drug efficacy. BMC Med.

[2] Walter ND, Dolganov GM, Garcia BJ, Worodria W, Andama A, Musisi E (2015). Transcriptional adaptation of drug-tolerant mycobacterium tuberculosis during treatment of human tuberculosis. J Infect Dis.

[3] Boshoff HI, Myers TG, Copp BR, McNeil MR, Wilson MA, Barry CE (2004). The transcriptional responses of mycobacterium tuberculosis to inhibitors of metabolism novel insights into drug mechanisms of action. J Biol Chem.

[4] Liu Y, Tan S, Huang L, Abramovitch RB, Rohde KH, Zimmerman MD (2016). Immune activation of the host cell induces drug tolerance in mycobacterium tuberculosis both in vitro and in vivo. J Exp Med.

[5] Murima P, de Sessions PF, Lim V, Naim AN, Bifani P, Boshoff HI (2013). Exploring the mode of action of bioactive compounds by microfluidic transcriptional profiling in mycobacteria. PLoS One.

[6] Wallis RS, Kim P, Cole S, Hanna D, Andrade BB, Maeurer M (2013). Tuberculosis biomarkers discovery: developments, needs, and challenges. Lancet Infect Dis.

[7] Dooley KE, Phillips PP, Nahid P, Hoelscher M. Challenges in the clinical assessment of novel tuberculosis drugs. Adv Drug Deliv Rev. 2016. 10.1016/j.addr.2016.01.014. 10.1016/j.addr.2016.01.014 PMC4903928 26827911

[8] Naftalin CM, Verma R, Gurumurthy M, Lu Q, Zimmerman M, Yeo BCM (2017). Coadministration of allopurinol to increase antimycobacterial efficacy of pyrazinamide as evaluated in a whole-blood bactericidal activity model. Antimicrob Agents Chemother.

[9] Gurumurthy M, Verma R, Naftalin CM, Hee KH, Lu Q, Tan KH (2017). Activity of faropenem with and without rifampicin against mycobacterium tuberculosis: evaluation in a whole-blood bactericidal activity trial. J Antimicrob Chemother.

[10] Naftalin CM, Verma R, Gurumurthy M, Hee KH, Lu Q, Yeo BCM (2018). Adjunctive use of celecoxib with anti-tuberculosis drugs: evaluation in a whole-blood bactericidal activity model. Sci Rep.

[11] Wallis RS, Palaci M, Vinhas S, Hise AG, Ribeiro FC, Landen K (2001). A whole blood bactericidal assay for tuberculosis. J Infect Dis.

[12] Wallis RS (2011). Assessment of whole-blood bactericidal activity in the evaluation of new Antituberculosis drugs.

[13] Wallis RS, Dawson R, Friedrich SO, Venter A, Paige D, Zhu T (2014). Mycobactericidal activity of sutezolid (PNU-100480) in sputum (EBA) and blood (WBA) of patients with pulmonary tuberculosis. PLoS One.

[14] Wallis RS, Jakubiec WM, Kumar V, Silvia AM, Paige D, Dimitrova D (2010). Pharmacokinetics and whole-blood bactericidal activity against mycobacterium tuberculosis of single doses of PNU-100480 in healthy volunteers. J Infect Dis.

[15] Zhu T, Friedrich SO, Diacon A, Wallis RS (2014). Population pharmacokinetic/pharmacodynamic analysis of the bactericidal activities of sutezolid (PNU-100480) and its major metabolite against intracellular mycobacterium tuberculosis in ex vivo whole-blood cultures of patients with pulmonary tuberculosis. Antimicrob Agents Chemother.

[16] Goude R, Amin A, Chatterjee D, Parish TJ (2009). The arabinosyltransferase EmbC is inhibited by ethambutol in mycobacterium tuberculosis. Antimicrob Agents Chemother.

[17] Mustaev A, Malik M, Zhao X, Kurepina N, Luan G, Oppegard LM (2014). Fluoroquinolone-gyrase-DNA complexes two modes of drug binding. J Biol Chem.

[18] Gopal P, Nartey W, Ragunathan P, Sarathy J, Kaya F, Yee M (2017). Pyrazinoic acid inhibits mycobacterial coenzyme a biosynthesis by binding to aspartate decarboxylase PanD. ACS Infect Dis.

[19] Dinesh N, Sharma S, Balganesh MJ (2013). Involvement of efflux pumps in the resistance to peptidoglycan synthesis inhibitors in mycobacterium tuberculosis. Antimicrob Agents Chemother.

[20] Leistikow RL, Morton RA, Bartek IL, Frimpong I, Wagner K, Voskuil MI (2010). The *Mycobacterium tuberculosis* DosR regulon assists in metabolic homeostasis and enables rapid recovery from nonrespiring dormancy. J Bacteriol.

[21] Wallis RS, Jakubiec W, Kumar V, Bedarida G, Silvia A, Paige D (2011). Biomarker-assisted dose selection for safety and efficacy in early development of PNU-100480 for tuberculosis. Antimicrob Agents Chemother.

[22] Wallis RS, Vinhas SA, Johnson JL, Ribeiro FC, Palaci M, Peres RL (2003). Whole blood bactericidal activity during treatment of pulmonary tuberculosis. J Infect Dis.

[23] VanderVen BC, Fahey RJ, Lee W, Liu Y, Abramovitch RB, Memmott C (2015). Novel inhibitors of cholesterol degradation in mycobacterium tuberculosis reveal how the bacterium's metabolism is constrained by the intracellular environment. PLoS Pathog.

[24] van Rijn SP, van Altena R, Akkerman OW, van Soolingen D, van der Laan T, de Lange WC (2016). Pharmacokinetics of ertapenem in patients with multidrug-resistant tuberculosis. Eur Respir J.

[25] Diacon AH, van der Merwe L, Barnard M, von Groote-Bidlingmaier F, Lange C, García-Basteiro AL (2016). β-Lactams against tuberculosis—new trick for an old dog?. N Engl J Med.

[26] Betts JC, McLaren A, Lennon MG, Kelly FM, Lukey PT, Blakemore SJ (2003). Signature gene expression profiles discriminate between isoniazid-, thiolactomycin-, and triclosan-treated mycobacterium tuberculosis. Antimicrob Agents Chemother.

[27] Vilchèze C, Jacobs J, William R (2007). The mechanism of isoniazid killing: clarity through the scope of genetics. Annu Rev Microbiol.

[28] Cross GB, Yeo BC, Hutchinson PE, Tan MC, Verma R, Lu Q (2019). Impact of selective immune-cell depletion on growth of mycobacterium tuberculosis (Mtb) in a whole-blood bactericidal activity (WBA) assay. PLoS One.

[29] Alsultan A, Peloquin CA (2014). Therapeutic drug monitoring in the treatment of tuberculosis: an update. Drugs..

[30] Magis-Escurra C, Later-Nijland HM, Alffenaar JW, Broeders J, Burger DM, van Crevel R (2014). Population pharmacokinetics and limited sampling strategy for first-line tuberculosis drugs and moxifloxacin. Int J Antimicrob Agents.

[31] Lubasch A, Keller I, Borner K, Koeppe P, Lode H (2000). Comparative pharmacokinetics of ciprofloxacin, gatifloxacin, grepafloxacin, levofloxacin, trovafloxacin, and moxifloxacin after single oral administration in healthy volunteers. Antimicrob Agents Chemother.

[32] Wise R, Andrews JM, Marshall G, Hartman G (1999). Pharmacokinetics and inflammatory-fluid penetration of moxifloxacin following oral or intravenous administration. Antimicrob Agents Chemother.

[33] Honeyborne I, McHugh TD, Phillips PP, Bannoo S, Bateson A, Carroll N (2011). Molecular bacterial load assay, a culture-free biomarker for rapid and accurate quantification of sputum mycobacterium tuberculosis bacillary load during treatment. J Clin Microbiol.

[34] Irizarry RA, Bolstad BM, Collin F, Cope LM, Hobbs B, Speed TP (2003). Summaries of Affymetrix GeneChip probe level data. Nucleic Acids Res.

[35] Bolstad BM, Irizarry RA, Åstrand M, Speed TP (2003). A comparison of normalization methods for high density oligonucleotide array data based on variance and bias. Bioinformatics..

[36] Ritchie ME, Phipson B, Wu D, Hu Y, Law CW, Shi W (2015). Limma powers differential expression analyses for RNA-sequencing and microarray studies. Nucleic Acids Res.

[37] Benjamini Y, Hochberg Y (1995). Controlling the false discovery rate: a practical and powerful approach to multiple testing. J R Stat Soc B Methodol.

[38] Dennis G, Sherman BT, Hosack DA, Yang J, Gao W, Lane HC (2003). DAVID: database for annotation, visualization, and integrated discovery. Genome Biol.

[39] Suzuki R, Shimodaira H (2011). Pvclust: hierarchical clustering with P-values via multiscale bootstrap resampling. R package version.

