# Supplementary material for: Gene expression responses to anti-tuberculous drugs in a whole blood model
Source: BMC Microbiol. 2020 Apr 7;20:81. doi: 10.1186/s12866-020-01766-y (PMC7140558; doi:10.1186/s12866-020-01766-y)
Supplement: Supplementary file 1 — Additional file 1. [file 12866_2020_1766_MOESM1_ESM.docx]

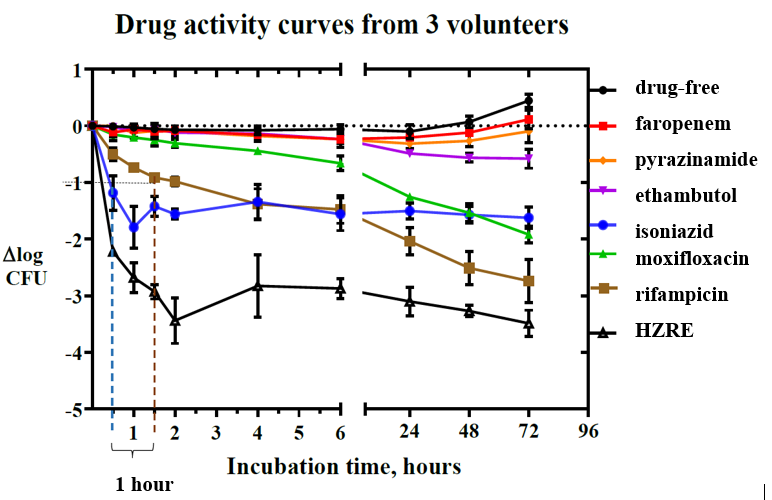


Supplementary Figure 1. Bactericidal curves of selected antibiotics. HZRE: standard TB 4-drug combination containing rifampicin, isoniazid, ethambutol and pyrazinamide.


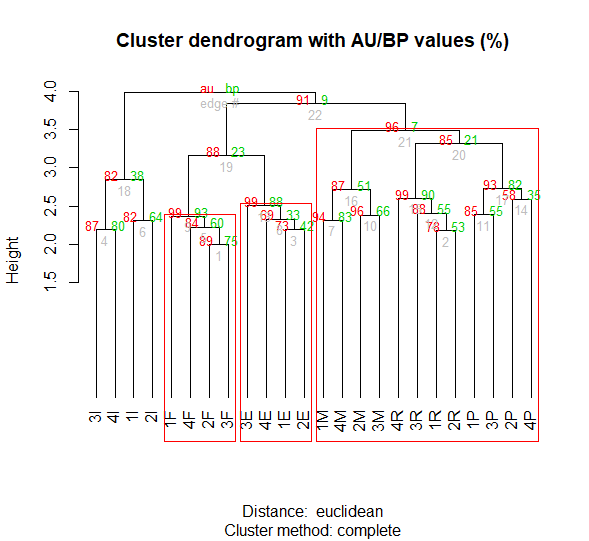


Supplementary Figure 2. Hierarchical cluster analysis results of the individual drug assays. Red values represent Approximately Unbiased (AU) p-values, Green values are Bootstrap Probability and grey values are edges (#) for the clusters. Clusters with AU larger than 95% are significant clusters supported by our gene values (highlighted in rectangular boxes). Analysis done using pvclust () R software package. The bottom area contains sample label according to patient number (numbered 1-4) next to the first letter of the antibiotic used in culture. I: isoniazid, F: faropenem, E: ethambutol, M: moxifloxacin, R:rifampicin, P:pyrazinamide.

Supplementary Table 1. List of differentially expressed genes for the 4-drug combination of isoniazid, pyrazinamide, rifampicin and ethambutol whole blood cultures relative to the broth cultures.

| Locus_tag | Gene symbol | Gene_description |
| --- | --- | --- |
| Rv2956 | Rv2956 | hypothetical protein |
| Rv3404c | Rv3404c | hypothetical protein |
| Rv2970A | Rv2970A | hypothetical protein |
| Rv1159A | Rv1159A | 4a-hydroxytetrahydrobiopterin dehydratase |
| Rv1320c | Rv1320c | adenylate cyclase |
| Rv0170 | mce1B | Mce family protein Mce1B |
| Rv0930 | pstA1 | phosphate ABC transporter permease PstA |
| Rv0156 | pntAb | NAD(P) transhydrogenase subunit alpha PntAb |
| Rv1611 | trpC | indole-3-glycerol phosphate synthase |
| Rv3681c | whiB4 | transcriptional regulator WhiB4 |
| Rv2906c | trmD | tRNA (guanine-N1)-methyltransferase |
| Rv0207c | Rv0207c | hypothetical protein |
| Rv1942c | mazF5 | toxin MazF5 |
| Rv3922c | Rv3922c | membrane protein insertion efficiency factor |
| Rv3052c | nrdI | NrdI protein |
| Rv3221c | TB7.3 | acetyl-CoA carboxylase biotin carboxyl carrier protein subunit |
| Rv3278c | Rv3278c | transmembrane protein |
| Rv0172 | mce1D | Mce family protein Mce1D |
| Rv1485 | hemZ | ferrochelatase |
| Rv2200c | ctaC | cytochrome C oxidase subunit II |
| Rv3697c | vapC48 | ribonuclease VapC48 |
| Rv1613 | trpA | tryptophan synthase subunit alpha |
| Rv3051c | nrdE | ribonucleoside-diphosphate reductase subunit alpha |
| Rv3739c | PPE67 | PPE family protein PPE67 |
| Rv0732 | secY | preprotein translocase SecY |
| Rv3320c | vapC44 | ribonuclease VapC44 |
| Rv1184c | Rv1184c | hypothetical protein |
| Rv3662c | Rv3662c | hypothetical protein |
| Rv3317 | sdhD | succinate dehydrogenase hydrophobic membrane anchor subunit |
| Rv2949c | Rv2949c | chorismate pyruvate-lyase |
| Rv1438 | tpi | triosephosphate isomerase |
| Rv0314c | Rv0314c | membrane protein |
| Rv3487c | lipF | carboxylesterase LipF |
| Rv0479c | Rv0479c | membrane protein |
| Rv3142c | Rv3142c | hypothetical protein |
| Rv3486 | Rv3486 | hypothetical protein |
| Rv1926c | mpt63 | immunogenic protein Mpt63 |
| Rv1322 | Rv1322 | hypothetical protein |
| Rv2901c | Rv2901c | hypothetical protein |
| Rv2938 | drrC | daunorubicin ABC transporter permease DrrC |
| Rv2246 | kasB | 3-oxoacyl-ACP synthase 2 |
| Rv3053c | nrdH | glutaredoxin electron transport protein NrdH |
| Rv3491 | Rv3491 | hypothetical protein |
| Rv3460c | rpsM | 30S ribosomal protein S13 |
| Rv3514 | PE_PGRS57 | PE-PGRS family protein PE_PGRS57 |
| Rv0247c | Rv0247c | succinate dehydrogenase iron-sulfur subunit |
| Rv1298 | rpmE | 50S ribosomal protein L31 |
| Rv2575 | Rv2575 | membrane protein |
| Rv1433 | Rv1433 | hypothetical protein |
| Rv0287 | esxG | ESAT-6 like protein EsxG |
| Rv3466 | Rv3466 | hypothetical protein |
| Rv3136A | Rv3136A | hypothetical protein |
| Rv3492c | Rv3492c | Mce associated protein |
| Rv3154 | nuoJ | NADH-quinone oxidoreductase subunit J |
| Rv2375 | Rv2375 | hypothetical protein |
| Rv3316 | sdhC | succinate dehydrogenase cytochrome B-556 subunit |
| Rv0268c | Rv0268c | hypothetical protein |
| Rv2806 | Rv2806 | membrane protein |
| Rv3582c | ispD | 2-C-methyl-D-erythritol 4-phosphate cytidylyltransferase |
| Rv2882c | frr | ribosome recycling factor |
| Rv3196A | Rv3196A | hypothetical protein |
| Rv1641 | infC | initiation factor IF-3 |
| Rv2115c | mpa | proteasome-associated ATPase |
| Rv0985c | mscL | large-conductance ion mechanosensitive channel |
| Rv0292 | eccE3 | ESX-3 secretion system protein EccE |
| Rv2948c | fadD22 | p-hydroxybenzoyl--AMP ligase |
| Rv3512 | PE_PGRS56 | PE-PGRS family protein PE_PGRS56 |
| Rv3583c | Rv3583c | RNA polymerase-binding transcription factor CarD |
| Rv2954c | Rv2954c | hypothetical protein |
| Rv1183 | mmpL10 | transmembrane transport protein MmpL10 |
| Rv3842c | glpQ1 | glycerophosphoryl diester phosphodiesterase |
| Rv1498c | Rv1498c | methyltransferase |
| Rv3823c | mmpL8 | integral membrane transport protein MmpL8 |
| Rv1216c | Rv1216c | integral membrane protein |
| Rv1228a | Rv1228a | hypothetical protein |
| Rv1632c | Rv1632c | hypothetical protein |
| Rv1185c | fadD21 | fatty-acid--CoA ligase FadD21 |
| Rv2193 | ctaE | cytochrome C oxidase subunit III |
| Rv0710 | rpsQ | 30S ribosomal protein S17 |
| Rv1794 | Rv1794 | hypothetical protein |
| Rv1312 | Rv1312 | hypothetical protein |
| Rv0761c | adhB | alcohol dehydrogenase B |
| Rv1773c | Rv1773c | transcriptional regulator |
| Rv2876 | Rv2876 | transmembrane protein |
| Rv0005 | gyrB | DNA gyrase subunit B |
| Rv3209 | Rv3209 | hypothetical protein |
| Rv1846c | blaI | transcriptional repressor BlaI |
| Rv0705 | rpsS | 30S ribosomal protein S19 |
| Rv3727 | Rv3727 | oxidoreductase |
| Rv3921c | Rv3921c | membrane protein insertase YidC |
| Rv1091 | PE_PGRS22 | PE-PGRS family protein PE_PGRS22 |
| Rv3824c | papA1 | acyltransferase |
| Rv1096 | Rv1096 | glycosyl hydrolase |
| Rv2342 | Rv2342 | hypothetical protein |
| Rv1876 | bfrA | bacterioferritin BfrA |
| Rv1830 | Rv1830 | HTH-type transcriptional regulator |
| Rv1780 | Rv1780 | hypothetical protein |
| Rv0176 | Rv0176 | Mce associated transmembrane protein |
| Rv3429 | PPE59 | PPE family protein PPE59 |
| Rv2953 | Rv2953 | trans-acting enoyl reductase |
| Rv0558 | menH | demethylmenaquinone methyltransferase |
| Rv2903c | lepB | signal peptidase |
| Rv3866 | espG1 | ESX-1 secretion-associated protein EspG |
| Rv3508 | PE_PGRS54 | PE-PGRS family protein PE_PGRS54 |
| Rv1404 | Rv1404 | transcriptional regulator |
| Rv0701 | rplC | 50S ribosomal protein L3 |
| Rv3495c | lprN | Mce family lipoprotein LprN |
| Rv0502 | Rv0502 | hypothetical protein |
| Rv3004 | cfp6 | low molecular weight protein antigen 6 |
| Rv2510c | Rv2510c | hypothetical protein |
| Rv1508c | Rv1508c | membrane protein |
| Rv3222c | Rv3222c | hypothetical protein |
| Rv2011c | Rv2011c | hypothetical protein |
| Rv2647 | Rv2647 | hypothetical protein |
| Rv0298 | Rv0298 | antitoxin |
| Rv0702 | rplD | 50S ribosomal protein L4 |
| Rv3615c | espC | ESX-1 secretion-associated protein EspC |
| Rv0685 | tuf | elongation factor Tu |
| Rv3806c | ubiA | decaprenyl-phosphate phosphoribosyltransferase |
| Rv0289 | espG3 | ESX-3 secretion-associated protein EspG3 |
| Rv1078 | Rv1078 | hypothetical protein |
| Rv1872c | lldD2 | L-lactate dehydrogenase |
| Rv2878c | mpt53 | soluble secreted antigen Mpt53 |
| Rv3207c | Rv3207c | hypothetical protein |
| Rv1904 | Rv1904 | hypothetical protein |
| Rv0174 | mce1F | Mce family protein Mce1F |
| Rv1502 | Rv1502 | hypothetical protein |
| Rv0709 | rpmC | 50S ribosomal protein L29 |
| Rv0700 | rpsJ | 30S ribosomal protein S10 |
| Rv2615c | PE_PGRS45 | PE-PGRS family protein PE_PGRS45 |
| Rv0048c | Rv0048c | membrane protein |
| Rv2390c | Rv2390c | hypothetical protein |
| Rv0462 | lpdC | dihydrolipoamide dehydrogenase |
| Rv0169 | mce1A | Mce family protein Mce1A |
| Rv1342c | Rv1342c | hypothetical protein |
| Rv1182 | papA3 | acyltransferase papA3 |
| Rv1887 | Rv1887 | hypothetical protein |
| Rv0277c | vapC25 | ribonuclease VapC25 |
| Rvnt41 | serV | 0 |
| Rv2238c | ahpE | peroxiredoxin |
| Rv1366A | Rv1366A | hypothetical protein |
| Rv1111c | Rv1111c | hypothetical protein |
| Rv2628 | Rv2628 | hypothetical protein |
| Rv1636 | TB15.3 | iron-regulated universal stress protein |
| Rv0010c | Rv0010c | membrane protein |
| Rv3412 | Rv3412 | hypothetical protein |
| Rv0178 | Rv0178 | Mce associated membrane protein |
| Rv3366 | spoU | tRNA/rRNA methylase SpoU |
| Rv2604c | snoP | glutamine amidotransferase SnoP |
| Rv0692 | Rv0692 | mycofactocin system protein MftB |
| Rv0787A | Rv0787A | hypothetical protein |
| Rv0338c | Rv0338c | iron-sulfur-binding reductase |
| Rv2585c | Rv2585c | lipoprotein |
| Rv3344c | PE_PGRS49 | PE-PGRS family protein PE_PGRS49 |
| Rv3050c | Rv3050c | AsnC family transcriptional regulator |
| Rv3596c | clpC1 | ATP-dependent protease ATP-binding subunit ClpC |
| Rv0683 | rpsG | 30S ribosomal protein S7 |
| Rv3131 | Rv3131 | NAD(P)H nitroreductase |
| Rv0240 | vapC24 | ribonuclease VapC24 |
| Rv0165c | mce1R | transcriptional regulator Mce1R |
| Rv3228 | Rv3228 | hypothetical protein |
| Rv2151c | ftsQ | cell division protein FtsQ |
| Rv3918c | parA | chromosome partitioning protein ParA |
| Rv0246 | Rv0246 | integral membrane protein |
| Rv3291c | lrpA | transcriptional regulator LrpA |
| Rv1212c | glgA | capsular glucan synthase |
| Rv1225c | Rv1225c | hypothetical protein |
| Rv2345 | Rv2345 | transmembrane protein |
| Rv3022A | PE29 | PE family protein PE29 |
| Rv2674 | msrB | peptide methionine sulfoxide reductase MsrB |
| Rv0060 | Rv0060 | hypothetical protein |
| Rv2632c | Rv2632c | hypothetical protein |
| Rv0376c | Rv0376c | hypothetical protein |
| Rv3128c | Rv3128c | Rv3128c |
| Rv2774c | Rv2774c | hypothetical protein |
| Rv3409c | choD | cholesterol oxidase |
| Rv0522 | gabP | GABA permease GabP |
| Rv0579 | Rv0579 | hypothetical protein |
| Rv1584c | Rv1584c | phage protein |
| Rv1903 | Rv1903 | membrane protein |
| Rv3377c | Rv3377c | type B diterpene cyclase |
| Rv3229c | desA3 | stearoyl-CoA 9-desaturase |
| Rv2336 | Rv2336 | hypothetical protein |
| Rv3339c | icd1 | isocitrate dehydrogenase |
| Rv1095 | phoH2 | phosphate starvation-inducible protein PsiH |
| Rv2616 | Rv2616 | hypothetical protein |
| Rv2945c | lppX | lipoprotein LppX |
| Rv2775 | Rv2775 | GCN5-like N-acetyltransferase |
| Rv0374c | Rv0374c | carbon monoxyde dehydrogenase small subunit |
| Rv1898 | Rv1898 | hypothetical protein |
| Rv2289 | cdh | CDP-diacylglycerol pyrophosphatase |
| Rv1450c | PE_PGRS27 | PE-PGRS family protein PE_PGRS27 |
| Rv0061c | Rv0061c | hypothetical protein |
| Rv2459 | Rv2459 | MFS-type transporter |
| Rv0655 | mkl | ABC transporter ATP-binding protein |
| Rv1088 | PE9 | PE family protein PE9 |
| Rv1738 | Rv1738 | hypothetical protein |
| Rv2129c | Rv2129c | oxidoreductase |
| Rv0167 | yrbE1A | membrane protein |
| Rv2203 | Rv2203 | membrane protein |
| Rv1308 | atpA | ATP synthase subunit alpha |
| Rv3920c | Rv3920c | hypothetical protein |
| Rv2080 | lppJ | lipoprotein LppJ |
| Rvnt06 | thrT | 0 |
| Rv0078B | Rv0078B | hypothetical protein |
| Rv1453 | Rv1453 | transcriptional activator protein |
| Rv2957 | Rv2957 | PGL/p-HBAD biosynthesis glycosyltransferase |
| Rv0711 | atsA | arylsulfatase AtsA |
| Rv1045 | Rv1045 | hypothetical protein |
| Rv0313 | Rv0313 | hypothetical protein |
| Rv2817c | Rv2817c | CRISPR-associated endonuclease Cas1 |
| Rv3745c | Rv3745c | hypothetical protein |
| Rv0931c | pknD | serinethreonine-protein kinase PknD |
| Rv1307 | atpH | ATP synthase subunit b/delta |
| Rv3678A | Rv3678A | hypothetical protein |
| Rv1346 | mbtN | acyl-[acyl-carrier-protein] dehydrogenase MbtN |
| Rv1766 | Rv1766 | hypothetical protein |
| Rvnt30 | lysU | 0 |
| Rv3277 | Rv3277 | transmembrane protein |
| Rv2431c | PE25 | PE family protein PE25 |
| Rvnr02 | rrl | 0 |
| Rv1528c | papA4 | polyketide synthase associated protein PapA |
| Rv1101c | Rv1101c | hypothetical protein |
| Rv1303 | Rv1303 | hypothetical protein |
| Rv2283 | Rv2283 | hypothetical protein |
| Rv0649 | fabD2 | malonyl CoA-acyl carrier protein transacylase |
| Rv1761c | Rv1761c | hypothetical protein |
| Rv2214c | ephD | oxidoreductase EphD |
| Rv1614 | lgt | prolipoprotein diacylglyceryl transferase |
| Rv1310 | atpD | ATP synthase subunit beta |
| Rv3783 | rfbD | O-antigenlipopolysaccharide ABC transporter permease RfbD |
| Rv3733c | Rv3733c | hypothetical protein |
| Rv3686c | Rv3686c | hypothetical protein |
| Rv0919 | Rv0919 | GCN5-like N-acetyltransferase |
| Rv0636 | hadB | (3R)-hydroxyacyl-ACP dehydratase subunit HadB |
| Rv3810 | pirG | cell surface protein |
| Rv3413c | Rv3413c | anti-sigma-D factor RsdA |
| Rv1304 | atpB | ATP synthase subunit A |
| Rv1051c | Rv1051c | hypothetical protein |
| Rv2537c | aroD | 3-dehydroquinate dehydratase |
| Rv3390 | lpqD | lipoprotein LpqD |
| Rv3799c | accD4 | propionyl-CoA carboxylase subunit beta AccD |
| Rv3001c | ilvC | ketol-acid reductoisomerase |
| Rv2721c | Rv2721c | hypothetical protein |
| Rv0935 | pstC1 | phosphate ABC transporter permease PstC |
| Rv0933 | pstB | phosphate ABC transporter ATP-binding protein PstB |
| Rv3458c | rpsD | 30S ribosomal protein S4 |
| Rv0559c | Rv0559c | hypothetical protein |
| Rv2239c | Rv2239c | hypothetical protein |
| Rv3135 | PPE50 | PPE family protein PPE50 |
| Rv0632c | echA3 | enoyl-CoA hydratase EchA3 |
| Rv2346c | esxO | ESAT-6 like protein EsxO |
| Rv3912 | Rv3912 | anti-sigma-M factor RsmA |
| Rv0230c | php | phosphotriesterase |
| Rv1323 | fadA4 | acetyl-CoA acetyltransferase |
| Rv2556c | Rv2556c | hypothetical protein |
| Rv0870c | Rv0870c | integral membrane protein |
| Rv2146c | Rv2146c | transmembrane protein |
| Rv2202c | adoK | adenosine kinase |
| Rv0984 | moaB2 | pterin-4-alpha-carbinolamine dehydratase |
| Rv2747 | argA | L-glutamate alpha-N-acetyltranferase |
| Rv3046c | Rv3046c | hypothetical protein |
| Rv3749c | Rv3749c | hypothetical protein |
| Rv0078A | Rv0078A | hypothetical protein |
| Rv1398c | vapB10 | antitoxin VapB10 |
| Rv2166c | Rv2166c | transcriptional regulator MraZ |
| Rv2941 | fadD28 | long-chain-fatty-acid--AMP ligase FadD28 |
| Rv3919c | gid | 16S rRNA (guanine(527)-N(7))-methyltransferase RsmG |
| Rv0721 | rpsE | 30S ribosomal protein S5 |
| Rv3212 | Rv3212 | hypothetical protein |
| Rv0267 | narU | nitrite extrusion protein NarU |
| Rv0095c | Rv0095c | hypothetical protein |
| Rv1083 | Rv1083 | hypothetical protein |
| Rv3480c | Rv3480c | diacyglycerol O-acyltransferase |
| Rv0896 | gltA2 | citrate synthase 1 |
| Rv1795 | eccD5 | ESX-5 type VII secretion system protein EccD |
| Rv2128 | Rv2128 | transmembrane protein |
| Rv2890c | rpsB | 30S ribosomal protein S2 |
| Rv2529 | Rv2529 | hypothetical protein |
| Rv0894 | Rv0894 | transcriptional regulator |
| Rv1094 | desA2 | acyl-ACP desaturase DesA |
| Rv2429 | ahpD | alkyl hydroperoxide reductase AphD |
| Rv0129c | fbpC | diacylglycerol acyltransferase/mycolyltransferase Ag85C |
| Rv2907c | rimM | 16S rRNA processing protein RimM |
| Rv0703 | rplW | 50S ribosomal protein L23 |
| Rv1745c | idi | isopentenyl-diphosphate delta-isomerase |
| Rv1500 | Rv1500 | glycosyltransferase |
| Rv2603c | Rv2603c | transcriptional regulator |
| Rv0001 | dnaA | chromosomal replication initiator protein DnaA |
| Rv1978 | Rv1978 | hypothetical protein |
| Rv0009 | ppiA | iron-regulated peptidyl-prolyl cis-trans isomerase PpiA |
| Rv0440 | groEL2 | molecular chaperone GroEL |
| Rv2663 | Rv2663 | hypothetical protein |
| Rv2986c | hupB | DNA-binding protein HU |
| Rv2929 | Rv2929 | hypothetical protein |
| Rv3584 | lpqE | lipoprotein LpqE |
| Rv1040c | PE8 | PE family protein PE8 |
| Rv2477c | Rv2477c | macrolide ABC transporter ATP-binding protein |
| Rv0788 | purQ | phosphoribosylformylglycinamidine synthase |
| Rv1870c | Rv1870c | hypothetical protein |
| Rv3259 | Rv3259 | hypothetical protein |
| Rv0024 | Rv0024 | NLP/P60 family protein |
| Rv1416 | ribH | 6%2C7-dimethyl-8-ribityllumazine synthase |
| Rv1080c | greA | transcription elongation factor GreA |
| Rv1671 | Rv1671 | membrane protein |
| Rv0177 | Rv0177 | Mce associated protein |
| Rv0936 | pstA2 | phosphate ABC transporter permease PstA |
| Rv2987c | leuD | 3-isopropylmalate dehydratase small subunit |
| Rv2930 | fadD26 | fatty-acid--CoA ligase FadD26 |
| Rv1816 | Rv1816 | HTH-type transcriptional regulator |
| Rv3198A | Rv3198A | glutaredoxin protein |
| Rv2927c | Rv2927c | hypothetical protein |
| Rv0634A | Rv0634A | hypothetical protein |
| Rv0509 | hemA | glutamyl-tRNA reductase |
| Rv3323c | moaX | MoaD-MoaE fusion protein MoaX |
| Rv1282c | oppC | oligopeptide ABC transporter permease OppC |
| Rv0282 | eccA3 | ESX-3 secretion system protein EccA |
| Rv0124 | PE_PGRS2 | PE-PGRS family protein PE_PGRS2 |
| Rv3141 | fadB4 | NADPH quinone oxidoreductase FadB |
| Rv2840c | Rv2840c | hypothetical protein |
| Rv2247 | accD6 | acetyl-propionyl-CoA carboxylase subunit beta |
| Rv0464c | Rv0464c | hypothetical protein |
| Rv2740 | ephG | epoxide hydrolase |
| Rv0501 | galE2 | UDP-glucose 4-epimerase GalE |
| Rv3345c | PE_PGRS50 | PE-PGRS family protein PE_PGRS50 |
| Rv0354c | PPE7 | PPE family protein PPE7 |
| Rv1919c | Rv1919c | hypothetical protein |
| Rv0812 | pabC | 4-amino-4-deoxychorismate lyase |
| Rv2862A | vapB23 | antitoxin VapB23 |
| Rv0020c | fhaA | FHA domain-containing protein FhaA |
| Rv1973 | Rv1973 | Mce associated membrane protein |
| Rv3654c | Rv3654c | hypothetical protein |
| Rv2530A | vapB39 | antitoxin VapB39 |
| Rv0163 | Rv0163 | hypothetical protein |
| Rv1274 | lprB | lipoprotein LprB |
| Rv3804c | fbpA | diacylglycerol acyltransferase/mycolyltransferase Ag85A |
| Rv1440 | secG | protein-export membrane protein SecG |
| Rv2536 | Rv2536 | transmembrane protein |
| Rv0145 | Rv0145 | S-adenosylmethionine-dependent methyltransferase |
| Rv3216 | Rv3216 | Rv3216 |
| Rv2965c | kdtB | phosphopantetheine adenylyltransferase |
| Rv1792 | esxM | - |
| Rv3854c | ethA | monooxygenase EthA |
| Rv1962A | vapB35 | antitoxin VapB35 |
| Rv3847 | Rv3847 | hypothetical protein |
| Rv2544 | lppB | lipoprotein LppB |
| Rv1840c | PE_PGRS34 | PE-PGRS family protein PE_PGRS34 |
| Rv2548 | vapC19 | ribonuclease VapC19 |
| Rv1038c | esxJ | ESAT-6 like protein EsxJ |
| Rv2347c | esxP | ESAT-6 like protein EsxP |
| Rv1197 | esxK | ESAT-6 like protein EsxK |
| Rv0838 | lpqR | lipoprotein LpqR |
| Rv2936 | drrA | daunorubicin ABC transporter ATP-binding protein DrrA |
| Rv1797 | eccE5 | ESX-5 type VII secretion system protein EccE |
| Rv2177c | Rv2177c | transposase |
| Rv2389c | rpfD | resuscitation-promoting factor RpfD |
| Rv2194 | qcrC | ubiquinol-cytochrome C reductase cytochrome subunit C |
| Rv1360 | Rv1360 | oxidoreductase |
| Rv0745 | Rv0745 | hypothetical protein |
| Rv3483c | Rv3483c | hypothetical protein |
| Rv0065 | vapC1 | ribonuclease VapC1 |
| Rv0637 | hadC | (3R)-hydroxyacyl-ACP dehydratase subunit HadC |
| Rv1048c | Rv1048c | hypothetical protein |
| Rv3620c | esxW | ESAT-6 like protein EsxW |
| Rv0824c | desA1 | acyl-ACP desaturase DesA |
| Rv0358 | Rv0358 | hypothetical protein |
| Rv0704 | rplB | 50S ribosomal protein L2 |
| Rv3376 | Rv3376 | phosphatase |
| Rv3158 | nuoN | NADH-quinone oxidoreductase subunit N |
| Rv1983 | PE_PGRS35 | PE-PGRS family protein PE_PGRS35 |
| Rv3766 | Rv3766 | hypothetical protein |
| Rv1047 | Rv1047 | transposase |
| Rv1199c | Rv1199c | insertion sequence element IS1081 transposase |
| Rv2512c | Rv2512c | insertion sequence element IS1081 transposase |
| Rv3023c | Rv3023c | transposase |
| Rv3115 | Rv3115 | transposase |
| Rv3213c | Rv3213c | SOJ/ParA-like protein |
| Rv2090 | Rv2090 | 5'-3' exonuclease |
| Rv3786c | Rv3786c | hypothetical protein |
| Rv1430 | PE16 | PE family protein PE16 |
| Rv1413 | Rv1413 | hypothetical protein |
| Rv2919c | glnB | nitrogen regulatory protein P-II |
| Rv2773c | dapB | 4-hydroxy-tetrahydrodipicolinate reductase |
| Rv0068 | Rv0068 | oxidoreductase |
| Rv0599c | vapB27 | antitoxin VapB27 |
| Rv3513c | fadD18 | fatty-acid--CoA ligase FadD18 |
| Rv2904c | rplS | 50S ribosomal protein L19 |
| Rv1565c | Rv1565c | acyltransferase |
| Rv0059 | Rv0059 | hypothetical protein |
| Rv0831c | Rv0831c | hypothetical protein |
| Rv0017c | rodA | cell division protein RodA |
| Rv0659c | mazF2 | toxin MazF2 |
| Rv1397c | vapC10 | ribonuclease VapC10 |
| Rv1306 | atpF | ATP synthase subunit B |
| Rv0488 | Rv0488 | amino acid transporter |
| Rv2736c | recX | regulatory protein RecX |
| Rv2329c | narK1 | nitratenitrite transporter |
| Rv0483 | lprQ | lipoprotein LprQ |
| Rv0667 | rpoB | DNA-directed RNA polymerase subunit beta |
| Rv2586c | secF | protein translocase subunit SecF |
| Rv3274c | fadE25 | acyl-CoA dehydrogenase |
| Rv2114 | Rv2114 | hypothetical protein |
| Rv2116 | lppK | lipoprotein LppK |
| Rv1296 | thrB | homoserine kinase |
| Rv0543c | Rv0543c | hypothetical protein |
| Rv2670c | Rv2670c | hypothetical protein |
| Rv3443c | rplM | 50S ribosomal protein L13 |
| Rv3923c | rnpA | ribonuclease P protein component |
| Rv0412c | Rv0412c | membrane protein |
| Rv1132 | Rv1132 | hypothetical protein |
| Rv1596 | nadC | nicotinate-nucleotide pyrophosphatase |
| Rv1869c | Rv1869c | reductase |
| Rv3715c | recR | recombination protein RecR |
| Rv2444c | rne | ribonuclease E |
| Rv1925 | fadD31 | fatty-acid--CoA ligase FadD31 |
| Rv3524 | Rv3524 | membrane protein |
| Rv2653c | Rv2653c | toxin |
| Rv1703c | Rv1703c | methyltransferase |
| Rv2846c | efpA | MFS-type transporter EfpA |
| Rv2737c | recA | recombinase A |
| Rv0472c | Rv0472c | HTH-type transcriptional regulator |
| Rv0833 | PE_PGRS13 | PE-PGRS family protein PE_PGRS13 |
| Rv1374c | Rv1374c | hypothetical protein |
| Rv3157 | nuoM | NADH-quinone oxidoreductase subunit M |
| Rv1117 | Rv1117 | hypothetical protein |
| Rv2784c | lppU | lipoprotein LppU |
| Rv1893 | Rv1893 | hypothetical protein |
| Rv0820 | phoT | phosphate ABC transporter ATP-binding protein PhoT |
| Rv2573 | Rv2573 | 2-dehydropantoate 2-reductase |
| Rv2364c | era | GTPase Era |
| Rv1198 | esxL | ESAT-6 like protein EsxL |
| Rv0082 | Rv0082 | oxidoreductase |
| Rv2708c | Rv2708c | hypothetical protein |
| Rv1037c | esxI | ESAT-6 like protein EsxI |
| Rv3619c | esxV | ESAT-6 like protein EsxV |
| Rv1894c | Rv1894c | hypothetical protein |
| Rv1957 | Rv1957 | SecB-like chaperone |
| Rv0241c | htdX | 3-hydroxyacyl-thioester dehydratase HtdX |
| Rv0932c | pstS2 | phosphate ABC transporter substrate-binding lipoprotein PstS |
| Rv1386 | PE15 | PE family protein PE15 |
| Rv2950c | fadD29 | long-chain-fatty-acid--AMP ligase FadD29 |
| Rv3156 | nuoL | NADH-quinone oxidoreductase subunit L |
| Rv2082 | Rv2082 | hypothetical protein |
| Rv2240c | Rv2240c | hypothetical protein |
| Rv3831 | Rv3831 | hypothetical protein |
| Rv2361c | uppS | decaprenyl diphosphate synthase |
| Rv3019c | esxR | ESAT-6 like protein EsxR |
| Rv3138 | pflA | pyruvate formate lyase activating protein PflA |
| Rv2781c | Rv2781c | oxidoreductase |
| Rv0603 | Rv0603 | hypothetical protein |
| Rv0071 | Rv0071 | maturase |
| Rv1334 | mec | [CysO]-cysteine peptidase |
| Rv1354c | Rv1354c | hypothetical protein |
| Rv1300 | hemK | release factor glutamine methyltransferase |
| Rv3220c | Rv3220c | two component sensor kinase |
| Rv1521 | fadD25 | fatty-acid--CoA ligase FadD25 |
| Rv2926c | Rv2926c | hypothetical protein |
| Rv3334 | Rv3334 | MerR family transcriptional regulator |
| Rv0857 | Rv0857 | hypothetical protein |
| Rv0171 | mce1C | Mce family protein Mce1C |
| Rv2665 | Rv2665 | hypothetical protein |
| Rv0640 | rplK | 50S ribosomal protein L11 |
| Rv1697 | Rv1697 | hypothetical protein |
| Rv0166 | fadD5 | fatty-acid--CoA ligase FadD5 |
| Rv1042c | Rv1042c | IS2-like transposase |
| Rv1149 | Rv1149 | transposase |
| Rv0840c | pip | proline iminopeptidase |
| Rv3448 | eccD4 | ESX-4 secretion system protein EccD4 |
| Rv2668 | Rv2668 | hypothetical protein |
| Rv0563 | htpX | protease HtpX |
| Rv3872 | PE35 | PE family protein PE35 |
| Rv0002 | dnaN | DNA polymerase III subunit beta |
| Rv3859c | gltB | glutamate synthase large subunit |
| Rv2885c | Rv2885c | transposase |
| Rv2853 | PE_PGRS48 | PE-PGRS family protein PE_PGRS48 |
| Rv3284 | Rv3284 | hypothetical protein |
| Rv1826 | gcvH | glycine cleavage system protein H |
| Rv1356c | Rv1356c | hypothetical protein |
| Rv1419 | Rv1419 | hypothetical protein |
| Rv1542c | glbN | hemoglobin GlbN |
| Rv1390 | rpoZ | DNA-directed RNA polymerase subunit omega |
| Rv0875c | Rv0875c | hypothetical protein |
| Rv0980c | PE_PGRS18 | PE-PGRS family protein PE_PGRS18 |
| Rv3430a | Rv3430a | hypothetical protein |
| Rv3197 | Rv3197 | ABC transporter ATP-binding protein |
| Rv0350 | dnaK | chaperone protein DnaK |
| Rv0206c | mmpL3 | transmembrane transport protein MmpL3 |
| Rv1152 | Rv1152 | transcriptional regulator |
| Rv1507c | Rv1507c | hypothetical protein |
| Rv1883c | Rv1883c | hypothetical protein |
| Rv2608 | PPE42 | PPE family protein PPE42 |
| Rv2443 | dctA | C4-dicarboxylate-transport transmembrane protein DctA |
| Rv2797c | Rv2797c | hypothetical protein |
| Rv1962c | vapC35 | ribonuclease VapC35 |
| Rv3062 | ligB | DNA ligase |
| Rv3579c | Rv3579c | 23S rRNA (guanosine(2251)-2'-O)-methyltransferase RlmB |
| Rv2391 | sirA | sulfite reductase |
| Rv3816c | Rv3816c | acyltransferase |
| Rv1109c | Rv1109c | hypothetical protein |
| Rv0466 | Rv0466 | hypothetical protein |
| Rv1688 | mpg | 3-methyladenine DNA glycosylase |
| Rv3909 | Rv3909 | hypothetical protein |
| Rv2140c | TB18.6 | hypothetical protein |
| Rv0888 | Rv0888 | hypothetical protein |
| Rv3819 | Rv3819 | hypothetical protein |
| Rv3682 | ponA2 | bifunctional penicillin-insensitive transglycosylase/penicillin-sensitive transpeptidase |
| Rv0519c | Rv0519c | membrane protein |
| Rv0979c | Rv0979c | hypothetical protein |
| Rv3008 | Rv3008 | hypothetical protein |
| Rv3802c | clp6 | membrane protein |
| Rv1050 | Rv1050 | oxidoreductase |
| Rv2808 | Rv2808 | hypothetical protein |
| Rv1138a | Rv1138a | hypothetical protein |
| Rv2328 | PE23 | PE family protein PE23 |
| Rv2707 | Rv2707 | hypothetical protein |
| Rv2861c | mapB | methionine aminopeptidase |
| Rv0545c | pitA | low-affinity inorganic phosphate transporter |
| Rv0469 | umaA | mycolic acid synthase UmaA |
| Rv3740c | Rv3740c | diacyglycerol O-acyltransferase |
| Rv2939 | papA5 | phthiocerol/phthiodiolone dimycocerosyl transferase |
| Rv0631c | recC | exonuclease V subunit gamma RecC |
| Rv3040c | Rv3040c | hypothetical protein |
| Rv0352 | dnaJ1 | chaperone protein DnaJ |
| Rv2828A | Rv2828A | hypothetical protein |
| Rv1623c | cydA | cytochrome D ubiquinol oxidase subunit I CydA |
| Rv1951c | Rv1951c | hypothetical protein |
| Rv2393 | che1 | ferrochelatase |
| Rv1187 | rocA | pyrroline-5-carboxylate dehydrogenase RocA |
| Rv1409 | ribG | bifunctional riboflavin biosynthesis diaminohydroxyphosphoribosylaminopyrimidine deaminase5-amino-6-(5-phosphoribosylamino) uracil reductase |
| Rv2111c | pup | ubiquitin-like protein Pup |
| Rv1626 | Rv1626 | two-component system transcriptional regulator |
| Rv0277A | vapB25 | Rv0277A |
| Rv1331 | Rv1331 | ATP-dependent Clp protease adapter protein ClpS |
| Rv2137c | Rv2137c | hypothetical protein |
| Rv0954 | Rv0954 | transmembrane protein |
| Rv1513 | Rv1513 | hypothetical protein |
| Rv3478 | PPE60 | PE family protein PPE60 |
| Rv1827 | garA | glycogen accumulation regulator GarA |
| Rv3677c | Rv3677c | beta lactamase |
| Rv1385 | pyrF | orotidine 5'-phosphate decarboxylase |
| Rv3768 | Rv3768 | hypothetical protein |
| Rv2620c | Rv2620c | transmembrane protein |
| Rv1164 | narI | nitrate reductase subunit gamma |
| Rv1134 | Rv1134 | hypothetical protein |
| Rv0624 | vapC30 | ribonuclease VapC30 |
| Rv0823c | Rv0823c | tRNA-dihydrouridine synthase |
| Rv2856 | nicT | nickel-transport integral membrane protein NicT |
| Rv1979c | Rv1979c | permease |
| Rv3747 | Rv3747 | hypothetical protein |
| Rv2857c | Rv2857c | 3-oxoacyl-ACP reductase |
| Rv2241 | aceE | pyruvate dehydrogenase E1 component |
| Rv3875 | esxA | ESAT-6 protein EsxA |
| Rv1489 | Rv1489 | hypothetical protein |
| Rv2635 | Rv2635 | hypothetical protein |
| Rv2245 | kasA | 3-oxoacyl-ACP synthase 1 |
| Rv3917c | parB | chromosome partitioning protein ParB |
| Rv3180c | Rv3180c | ribonuclease VapC45 |
| Rv2136c | Rv2136c | undecaprenyl-diphosphatase |
| Rv2645 | Rv2645 | hypothetical protein |
| Rv2891 | Rv2891 | hypothetical protein |
| Rv1555 | frdD | fumarate reductase membrane anchor subunit |
| Rv0532 | PE_PGRS6 | PE-PGRS family protein PE_PGRS6 |
| Rv0535 | Rv0535 | 5'-methylthioadenosine phosphorylase |
| Rv3868 | eccA1 | ESX-1 secretion system protein EccA1 |
| Rv0140 | Rv0140 | hypothetical protein |
| Rv1734c | Rv1734c | hypothetical protein |
| Rv3130c | tgs1 | diacyglycerol O-acyltransferase |
| Rv2142A | parD2 | antitoxin ParD2 |
| Rv2430c | PPE41 | PPE family protein PPE41 |
| Rv0424c | Rv0424c | hypothetical protein |
| Rv2093c | tatC | Sec-independent protein translocase transmembrane protein TatC |
| Rv1881c | lppE | lipoprotein LppE |
| RVnc0036 | MTS1338 | 0 |
| Rv1776c | Rv1776c | transcriptional regulator |
| Rv0805 | Rv0805 | 3'%2C5'-cyclic adenosine monophosphate phosphodiesterase CpdA |
| Rv3382c | Rv3382c | 4-hydroxy-3-methylbut-2-enyl diphosphate reductase |
| Rv1223 | htrA | serine protease HtrA |
| Rv1646 | PE17 | PE family protein PE17 |
| Rv3614c | espD | ESX-1 secretion-associated protein EspD |
| Rv1072 | Rv1072 | transmembrane protein |
| Rv1482c | Rv1482c | hypothetical protein |
| Rv0877 | Rv0877 | hypothetical protein |
| Rv0726c | Rv0726c | S-adenosylmethionine-dependent methyltransferase |
| Rv3527 | Rv3527 | hypothetical protein |
| Rv1834 | lipZ | hydrolase |
| Rv1461 | Rv1461 | hypothetical protein |
| Rv1436 | gap | glyceraldehyde 3-phosphate dehydrogenase |
| Rv0387c | Rv0387c | Rv0387c |
| Rv3477 | PE31 | PE family protein PE31 |
| Rv2109c | prcA | proteasome subunit alpha |
| Rv1801 | PPE29 | PPE family protein PPE29 |
| Rv0006 | gyrA | DNA gyrase subunit A |
| Rv1490 | Rv1490 | membrane protein |
| Rv1141c | echA11 | enoyl-CoA hydratase EchA11 |
| RVnc0035 | MTS1082 | 0 |
| Rv3826 | fadD23 | long-chain-fatty-acid--CoA ligase FadD23 |
| Rv0420c | Rv0420c | transmembrane protein |
| Rv2256a | Rv2256a | hypothetical protein |
| Rv0634c | Rv0634c | glyoxalase II |
| Rv0083 | Rv0083 | oxidoreductase |
| Rv2553c | Rv2553c | membrane protein |
| Rv0717 | rpsN1 | 30S ribosomal protein S14 |
| Rv2368c | phoH1 | phosphate starvation-inducible protein PhoH |
| Rv1868 | Rv1868 | hypothetical protein |
| Rv1969 | mce3D | Mce family protein Mce3D |
| Rv0364 | Rv0364 | transmembrane protein |
| Rv1825 | Rv1825 | hypothetical protein |
| Rv0455c | Rv0455c | hypothetical protein |
| Rv0492A | Rv0492A | hypothetical protein |
| Rv1352 | Rv1352 | hypothetical protein |
| Rv0719 | rplF | 50S ribosomal protein L6 |
| Rv0658c | Rv0658c | integral membrane protein |
| Rv1569 | bioF1 | 8-amino-7-oxononanoate synthase |
| Rv0249c | Rv0249c | succinate dehydrogenase membrane anchor subunit |
| Rv0602c | tcrA | two component DNA binding transcriptional regulator TcrA |
| Rv3092c | Rv3092c | integral membrane protein |
| Rv0830 | Rv0830 | S-adenosylmethionine-dependent methyltransferase |
| Rv3851 | Rv3851 | membrane protein |
| Rv3205c | Rv3205c | hypothetical protein |
| Rv3853 | rraA | RNase E regulator RraA |
| Rv2412 | rpsT | 30S ribosomal protein S20 |
| Rv1260 | Rv1260 | oxidoreductase |
| Rv0904c | accD3 | acetyl-CoAcarboxylase carboxyl transferase subunit beta |
| Rv0135c | Rv0135c | transcriptional regulator |
| Rv3680 | Rv3680 | anion transporter ATPase |
| Rv2828c | Rv2828c | hypothetical protein |
| Rv2463 | lipP | esteraselipase LipP |
| Rv0787 | Rv0787 | hypothetical protein |
| Rv0446c | Rv0446c | transmembrane protein |
| Rv0790c | Rv0790c | hypothetical protein |
| Rv3143 | Rv3143 | response regulator |
| Rv0865 | mog | molybdopterin biosynthesis protein |
| Rv2696c | Rv2696c | hypothetical protein |
| Rv0345 | Rv0345 | hypothetical protein |
| Rv2019 | Rv2019 | hypothetical protein |
| Rv1321 | Rv1321 | endonuclease NucS |
| Rv2496c | bkdB | 3-methyl-2-oxobutanoate dehydrogenase subunit beta |
| Rv1297 | rho | transcription termination factor Rho |
| Rv2460c | clpP2 | ATP-dependent CLP protease proteolytic subunit 2 |
| Rv3116 | moeB2 | molybdenum cofactor biosynthesis protein MoeB |
| Rv3136 | PPE51 | PPE family protein PPE51 |
| Rv3916c | Rv3916c | hypothetical protein |
| Rv1509 | Rv1509 | hypothetical protein |
| Rv0951 | sucC | succinyl-CoA ligase subunit beta |
| Rv3803c | fbpD | MPT51MPB51 antigen |
| Rv1594 | nadA | quinolinate synthetase A |
| Rv0164 | TB18.5 | hypothetical protein |
| Rv2015c | Rv2015c | hypothetical protein |
| Rv0715 | rplX | 50S ribosomal protein L24 |
| Rv0148 | Rv0148 | short-chain type dehydrogenase/reductase |
| Rv3408 | vapC47 | ribonuclease VapC47 |
| Rv3048c | nrdF2 | ribonucleoside-diphosphate reductase subunit beta NrdF2 |
| Rv2588c | yajC | membrane protein secretion factor YajC |
| Rv0284 | eccC3 | ESX-3 secretion system protein EccC3 |
| Rv1948c | Rv1948c | hypothetical protein |
| Rv1259 | udgB | uracil DNA glycosylase |
| Rv3098c | Rv3098c | hypothetical protein |
| Rv0173 | lprK | Mce family lipoprotein LprK |
| Rv3241c | Rv3241c | hypothetical protein |
| Rv1539 | lspA | lipoprotein signal peptidase |
| Rv1481 | Rv1481 | membrane protein |
| Rv1501 | Rv1501 | hypothetical protein |
| Rv0279c | PE_PGRS4 | PE-PGRS family protein PE_PGRS4 |
| Rv1793 | esxN | ESAT-6 like protein EsxN |
| Rv2999 | lppY | lipoprotein LppY |
| Rv3368c | Rv3368c | oxidoreductase |
| Rv2943A | Rv2943A | transposase |
| Rv3251c | rubA | rubredoxin RubA |
| Rv3155 | nuoK | NADH-quinone oxidoreductase subunit K |
| Rv2392 | cysH | phosphoadenosine phosphosulfate reductase |
| Rv0800 | pepC | M18 family aminopeptidase |
| Rv0076c | Rv0076c | membrane protein |
| Rv3843c | Rv3843c | transmembrane protein |
| Rv0669c | Rv0669c | neutral ceramidase |
| Rv1322A | Rv1322A | hypothetical protein |
| Rv1012 | Rv1012 | hypothetical protein |
| Rv3732 | Rv3732 | hypothetical protein |
| Rv3624c | hpt | hypoxanthine-guanine phosphoribosyltransferase |
| Rv1600 | hisC1 | histidinol-phosphate aminotransferase |
| Rv2349c | plcC | phospholipase C |
| Rv2197c | Rv2197c | transmembrane protein |
| Rv1981c | nrdF1 | ribonucleoside-diphosphate reductase subunit beta NrdF1 |
| Rv2001 | Rv2001 | hypothetical protein |
| Rv3634c | galE1 | UDP-glucose 4-epimerase |
| Rv1765c | Rv1765c | hypothetical protein |
| Rv3087 | Rv3087 | diacyglycerol O-acyltransferase |
| Rv1698 | mctB | copper transporter MctB |
| Rv1102c | mazF3 | mRNA interferase MazF3 |
| Rv3517 | Rv3517 | hypothetical protein |
| Rv0641 | rplA | 50S ribosomal protein L1 |
| Rv1941 | Rv1941 | short-chain type dehydrogenase/reductase |
| Rv3684 | Rv3684 | lyase |
| Rv0019c | fhaB | FHA domain-containing protein FhaB |
| Rv0300 | vapB2 | antitoxin VapB2 |
| Rv2749 | Rv2749 | hypothetical protein |
| Rv0786c | Rv0786c | hypothetical protein |
| Rv1593c | Rv1593c | hypothetical protein |
| Rv3691 | Rv3691 | hypothetical protein |
| Rv3348 | Rv3348 | transposase |
| Rv3844 | Rv3844 | transposase |
| Rv1852 | ureG | urease accessory protein UreG |
| Rv0064A | vapB1 | antitoxin VapB1 |
| Rv0470c | pcaA | cyclopropane mycolic acid synthase |
| Rv0682 | rpsL | 30S ribosomal protein S12 |
| Rv1963c | mce3R | transcriptional repressor Mce3R |
| Rv3335c | Rv3335c | integral membrane protein |
| Rv3509c | ilvX | acetohydroxyacid synthase large subunit |
| Rv1591 | Rv1591 | transmembrane protein |
| Rv1211 | Rv1211 | hypothetical protein |
| Rv0713 | Rv0713 | transmembrane protein |
| Rv3905c | esxF | ESAT-6 like protein EsxF |
| Rv0491 | regX3 | two component sensory transduction protein RegX |
| Rv0137c | msrA | peptide methionine sulfoxide reductase MsrA |
| Rv2767c | Rv2767c | membrane protein |
| Rv1121 | zwf1 | glucose-6-phosphate 1-dehydrogenase |
| Rv0063 | Rv0063 | oxidoreductase |
| Rv3255c | manA | mannose-6-phosphate isomerase |
| Rv3860 | Rv3860 | hypothetical protein |
| Rv3280 | accD5 | propionyl-CoA carboxylase subunit beta |
| Rv0598c | vapC27 | ribonuclease VapC27 |
| Rv1028c | kdpD | sensor protein KdpD |
| Rv1648 | Rv1648 | transmembrane protein |
| Rv1441c | PE_PGRS26 | PE-PGRS family protein PE_PGRS26 |
| Rv2182c | Rv2182c | 1-acylglycerol-3-phosphate O-acyltransferase |
| Rv2108 | PPE36 | PPE family protein PPE36 |
| Rv0101 | nrp | peptide synthetase Nrp |
| Rv2174 | mptA | alpha(1->6)-mannopyranosyltransferase A |
| Rv2190c | Rv2190c | endopeptidase |
| RVnc0008 | F6 | 0 |
| Rv3129 | Rv3129 | Rv3129 |
| Rv1192 | Rv1192 | hypothetical protein |
| Rv0809 | purM | phosphoribosylformylglycinamidine cyclo-ligase PurM |
| Rv3232c | ppk2 | polyphosphate kinase |
| Rv2837c | Rv2837c | bifunctional oligoribonuclease/PAP phosphatase NrnA |
| Rv3837c | Rv3837c | phosphoglycerate mutase |
| Rv3884c | eccA2 | ESX-2 secretion system protein EccA |
| Rv3146 | nuoB | NADH-quinone oxidoreductase subunit B |
| Rv0368c | Rv0368c | hypothetical protein |
| Rv2698 | Rv2698 | transmembrane protein |
| Rv3211 | rhlE | ATP-dependent RNA helicase RhlE |
| Rv3357 | relJ | antitoxin RelJ |
| Rv3362c | Rv3362c | ATPGTP-binding protein |
| Rv1704c | cycA | D-serinealanineglycine transporter protein CycA |
| Rv2199c | Rv2199c | cytochrome c oxidase polypeptide 4 |
| Rv2699c | Rv2699c | hypothetical protein |
| Rv0620 | galK | galactokinase |
| Rv0759c | Rv0759c | hypothetical protein |
| Rv2010 | vapC15 | ribonuclease VapC15 |
| Rv3295 | Rv3295 | TetR family transcriptional regulator |
| Rv3506 | fadD17 | long-chain-fatty-acid--CoA ligase FadD17 |
| Rv1932 | tpx | 2-Cys peroxiredoxin |
| Rv3221A | rshA | anti-sigma factor RshA |
| Rv2524c | fas | fatty acid synthase |
| Rv1311 | atpC | ATP synthase subunit epsilon |
| Rv2187 | fadD15 | long-chain-fatty-acid--CoA ligase FadD15 |
| Rv1305 | atpE | ATP synthase subunit C |
| Rv3424c | Rv3424c | hypothetical protein |
| Rv2920c | amt | ammonium transporter integral membrane protein |
| Rv2195 | qcrA | ubiquinol-cytochrome C reductase rieske iron-sulfur subunit |
| Rv2126c | PE_PGRS37 | PE-PGRS family protein PE_PGRS37 |
| Rv0495c | Rv0495c | hypothetical protein |
| Rv0203 | Rv0203 | hypothetical protein |
| Rv2414c | Rv2414c | hypothetical protein |
| Rv2363 | amiA2 | amidase |
| Rv3821 | Rv3821 | integral membrane protein |
| Rv2888c | amiC | amidase AmiC |
| Rv3723 | Rv3723 | transmembrane protein |
| Rv0211 | pckA | phosphoenolpyruvate carboxykinase |
| Rv2457c | clpX | ATP-dependent CLP protease ATP-binding subunit ClpX |
| Rvnt08 | trpT | 0 |
| Rv0597c | Rv0597c | hypothetical protein |
| Rv0880 | Rv0880 | HTH-type transcriptional regulator |
| Rv3132c | devS | two component sensor histidine kinase DevS |
| Rv2881c | cdsA | phosphatidate cytidylyltransferase |
| Rv3591c | Rv3591c | hydrolase |
| Rv1835c | Rv1835c | serine esterase |
| Rv0272c | Rv0272c | hypothetical protein |
| Rv2442c | rplU | 50S ribosomal protein L21 |
| Rv1339 | Rv1339 | hypothetical protein |
| Rv2081c | Rv2081c | transmembrane protein |
| Rv2267c | Rv2267c | hypothetical protein |
| Rv1959c | parE1 | toxin ParE1 |
| Rv0346c | ansP2 | L-asparagine permease |
| Rv2196 | qcrB | ubiquinol-cytochrome C reductase cytochrome subunit B |
| Rv1777 | cyp144 | cytochrome P450 Cyp144 |
| Rv2913c | Rv2913c | D-amino acid aminohydrolase |
| Rv2098c | PE_PGRS36 | Rv2098c |
| Rv3127 | Rv3127 | hypothetical protein |
| Rv1479 | moxR1 | transcriptional regulator MoxR1 |
| Rv3436c | glmS | glucosamine--fructose-6-phosphate aminotransferase |
| Rv2441c | rpmA | 50S ribosomal protein L27 |
| Rv3165c | Rv3165c | hypothetical protein |
| Rv3190A | Rv3190A | hypothetical protein |
| Rv3289c | Rv3289c | transmembrane protein |
| Rv2785c | rpsO | 30S ribosomal protein S15 |
| Rv2732c | Rv2732c | transmembrane protein |
| Rv0821c | phoY2 | phosphate-transport system transcriptional regulator PhoY2 |
| Rv2079 | Rv2079 | hypothetical protein |
| Rv1347c | mbtK | lysine N-acetyltransferase MbtK |
| Rv1700 | Rv1700 | NUDIX hydrolase |
| Rv1574 | Rv1574 | phage protein |
| Rv1415 | ribA2 | bifunctional riboflavin biosynthesis GTP cyclohydrolase II3%2C4-dihydroxy-2-butanone 4-phosphate synthase |
| Rv3464 | rmlB | dTDP-glucose 4%2C6-dehydratase |
| Rv0375c | Rv0375c | carbon monoxyde dehydrogenase medium subunit |
| Rv1049 | Rv1049 | transcriptional repressor |
| Rv2602 | vapC41 | ribonuclease VapC41 |
| Rv2714 | Rv2714 | hypothetical protein |
| Rv0708 | rplP | 50S ribosomal protein L16 |
| Rv3167c | Rv3167c | TetR family transcriptional regulator |
| Rv1785c | cyp143 | cytochrome P450 Cyp143 |
| Rv0301 | vapC2 | ribonuclease VapC2 |
| Rv2993c | Rv2993c | 2-hydroxyhepta-2%2C4-diene-1%2C7-dioate isomerase |
| Rv2932 | ppsB | phthiocerol synthesis polyketide synthase type I PpsB |
| Rv0676c | mmpL5 | transmembrane transport protein MmpL5 |
| Rv3166c | Rv3166c | hypothetical protein |
| Rv1081c | Rv1081c | membrane protein |
| Rv3271c | Rv3271c | integral membrane protein |
| Rv2745c | clgR | transcriptional regulator ClgR |
| Rv2148c | Rv2148c | hypothetical protein |
| Rv3169 | Rv3169 | hypothetical protein |
| Rv1299 | prfA | peptide chain release factor PrfA |
| Rv0377 | Rv0377 | HTH-type transcriptional regulator |
| Rv2892c | PPE45 | PPE family protein PPE45 |
| Rv0430 | Rv0430 | hypothetical protein |
| Rv0807 | Rv0807 | hypothetical protein |
| Rv3452 | cut4 | cutinase |
| Rv0970 | Rv0970 | integral membrane protein |
| Rv3616c | espA | ESX-1 secretion-associated protein EspA |
| Rv0746 | PE_PGRS9 | PE-PGRS family protein PE_PGRS9 |
| Rv3924c | rpmH | 50S ribosomal protein L34 |
| Rv0937c | mku | non-homologous end joining protein Ku |
| Rv0014c | pknB | serinethreonine-protein kinase PknB |
| Rv3394c | Rv3394c | hypothetical protein |
| Rv2495c | bkdC | branched-chain keto acid dehydrogenase E2 component |
| Rv3016 | lpqA | lipoprotein LpqA |
| Rv3778c | Rv3778c | aminotransferase |
| Rv1327c | glgE | alpha-1%2C4-glucan:maltose-1-phosphate maltosyltransferase |
| Rv3721c | dnaZX | DNA polymerase III subunit gammatau |
| Rv0253 | nirD | nitrite reductase small subunit NirD |
| Rv2642 | Rv2642 | ArsR family transcriptional regulator |
| Rv1068c | PE_PGRS20 | PE-PGRS family protein PE_PGRS20 |
| Rv3152 | nuoH | NADH-quinone oxidoreductase subunit H |
| Rv0600c | Rv0600c | two component sensor kinase HK1 |
| Rv1033c | trcR | two component transcriptional regulator TrcR |
| Rv1172c | PE12 | PE family protein PE12 |
| Rv3388 | PE_PGRS52 | PE-PGRS family protein PE_PGRS52 |
| Rv2516c | Rv2516c | hypothetical protein |
| Rv3196 | Rv3196 | hypothetical protein |
| Rv2746c | pgsA3 | CDP-diacylglycerol--glycerol-3-phosphate 3-phosphatidyltransferase |
| Rv3119 | moaE1 | molybdopterin synthase catalytic subunit 1 |
| Rv0882 | Rv0882 | transmembrane protein |
| Rv1980c | mpt64 | immunogenic protein Mpt64 |
| Rv0190 | Rv0190 | hypothetical protein |
| Rv1597 | Rv1597 | hypothetical protein |
| Rv1309 | atpG | ATP synthase subunit gamma |
| Rv2044c | Rv2044c | hypothetical protein |
| Rv3736 | Rv3736 | AraC/XylS family transcriptional regulator |
| Rv3056 | dinP | DNA polymerase IV 2 |
| Rv0011c | Rv0011c | cell division protein CrgA |
| Rv0988 | Rv0988 | hypothetical protein |
| Rv3515c | fadD19 | acyl-CoA synthetase |
| Rv3411c | guaB2 | inosine-5'-monophosphate dehydrogenase |
| Rv3759c | proX | glycine betainecarnitinecholineL-proline ABC transporter substrate-binding lipoprotein ProX |
| Rv2951c | Rv2951c | phthiodiolone/phenolphthiodiolone dimycocerosates ketoreductase |
| Rv0654 | Rv0654 | carotenoid cleavage oxygenase |
| Rv0283 | eccB3 | ESX-3 secretion system protein EccB3 |
| Rv2172c | Rv2172c | hypothetical protein |
| Rv3687c | rsfB | anti-anti-sigma factor RsfB |
| Rv0128 | Rv0128 | transmembrane protein |
| Rv2641 | cadI | cadmium inducible protein CadI |
| Rv0660c | mazE2 | antitoxin MazE2 |
| Rv2097c | pafA | proteasome accessory factor PafA |
| Rv1673c | Rv1673c | hypothetical protein |
| Rv0443 | Rv0443 | hypothetical protein |
| Rv2470 | glbO | hemoglobin GlbO |
| Rv3580c | cysS1 | cysteine--tRNA ligase |
| Rv3039c | echA17 | enoyl-CoA hydratase EchA17 |
| Rv2178c | aroG | phospho-2-dehydro-3-deoxyheptonate aldolase AroG |
| Rv2054 | Rv2054 | hypothetical protein |
| Rv0725c | Rv0725c | hypothetical protein |
| Rv1406 | fmt | methionyl-tRNA formyltransferase |
| Rv2513 | Rv2513 | hypothetical protein |
| Rv1016c | lpqT | lipoprotein LpqT |
| Rv2937 | drrB | daunorubicin ABC transporter permease DrrB |
| Rv2858c | aldC | aldehyde dehydrogenase AldC |
| Rv2968c | Rv2968c | integral membrane protein |
| Rv0108c | Rv0108c | hypothetical protein |
| Rv3217c | Rv3217c | integral membrane protein |
| Rv3011c | gatA | glutamyl-tRNA(GLN) amidotransferase subunit A |
| Rvnr01 | rrs | 0 |
| Rv1222 | rseA | anti-sigma E factor RseA |
| Rv1124 | ephC | epoxide hydrolase EphC |
| Rv2353c | PPE39 | PPE family protein PPE39 |
| Rv1264 | Rv1264 | adenylyl cyclase |
| Rv0008c | Rv0008c | cell wall synthesis protein CwsA |
| Rv0310c | Rv0310c | hypothetical protein |
| Rv0373c | Rv0373c | carbon monoxyde dehydrogenase large subunit |
| Rv3664c | dppC | dipeptide ABC transporter permease DppC |
| Rv3288c | usfY | hypothetical protein |
| Rv2427A | oxyR' | Rv2427A |
| Rv1877 | Rv1877 | MFS-type transporter |
| Rv0159c | PE3 | PE family protein PE3 |
| Rv1066 | Rv1066 | hypothetical protein |
| Rv0940c | Rv0940c | oxidoreductase |
| Rv0383c | Rv0383c | hypothetical protein |
| Rv3484 | Rv3484 | hypothetical protein |
| Rv3459c | rpsK | 30S ribosomal protein S11 |
| Rv2461c | clpP1 | ATP-dependent CLP protease proteolytic subunit 1 |
| Rv0718 | rpsH | 30S ribosomal protein S8 |
| Rv2035 | Rv2035 | hypothetical protein |
| Rv1316c | ogt | methylated-DNA--protein-cysteine methyltransferase |
| Rv1608c | bcpB | peroxiredoxin |
| Rv0090 | Rv0090 | membrane protein |
| Rv3133c | devR | two component transcriptional regulator DevR |
| Rv1403c | Rv1403c | methyltransferase |
| Rv0873 | fadE10 | acyl-CoA dehydrogenase |
| Rv2041c | Rv2041c | sugar ABC transporter substrate-binding lipoprotein |
| Rv1503c | Rv1503c | Rv1503c |
| Rv3002c | ilvN | acetolactate synthase small subunit |
| Rv1931c | Rv1931c | transcriptional regulator |
| Rv2710 | sigB | RNA polymerase sigma factor SigB |
| Rv3623 | lpqG | lipoprotein LpqG |
| Rv0223c | Rv0223c | aldehyde dehydrogenase |
| Rv2135c | Rv2135c | hypothetical protein |
| Rv2144c | Rv2144c | transmembrane protein |
| Rv1652 | argC | N-acetyl-gamma-glutamyl-phoshate reductase |
| Rv3825c | pks2 | phthioceranichydroxyphthioceranic acid synthase |
| Rvnt34 | valU | 0 |
| Rv2399c | cysT | sulfate ABC transporter permease CysT |
| Rv2680 | Rv2680 | hypothetical protein |
| Rv2085 | Rv2085 | hypothetical protein |
| Rv1902c | nanT | sialic acid-transport integral membrane protein NanT |
| Rv1115 | Rv1115 | hypothetical protein |
| Rv2150c | ftsZ | cell division protein FtsZ |
| Rv0487 | Rv0487 | hypothetical protein |
| Rv2077A | Rv2077A | hypothetical protein |
| RVnc0036a | MTS2823 | 0 |
| Rv2715 | Rv2715 | hydrolase |
| Rv0336 | Rv0336 | hypothetical protein |
| Rv3493c | Rv3493c | Mce associated protein |
| Rv0642c | mmaA4 | hydroxymycolate synthase MmaA4 |
| Rv2692 | ceoC | TRK system potassium uptake protein CeoC |
| Rv3309c | upp | uracil phosphoribosyltransferase |
| Rv3858c | gltD | glutamate synthase small subunit |
| Rv1043c | Rv1043c | hypothetical protein |
| Rv1640c | lysX | bifunctional lysine--tRNA ligasephosphatidylglycerol lysyltransferase |
| Rv1290A | Rv1290A | hypothetical protein |
| Rv0198c | zmp1 | zinc metalloprotease |
| Rv3801c | fadD32 | long-chain-fatty-acid--AMP ligase FadD32 |
| Rv1414 | Rv1414 | hypothetical protein |
| Rv2474c | Rv2474c | hypothetical protein |
| Rv3471c | Rv3471c | hypothetical protein |
| Rv3894c | eccC2 | ESX-2 type VII secretion system protein EccC |
| Rv1472 | echA12 | enoyl-CoA hydratase EchA12 |
| Rv1041c | Rv1041c | IS2-like transposase |
| Rvnt03 | leuT | 0 |
| Rv2028c | Rv2028c | universal stress protein |
| Rv2636 | Rv2636 | O-phosphotransferase |
| Rv0955 | Rv0955 | integral membrane protein |
| Rv0652 | rplL | 50S ribosomal protein L7/L12 |
| Rv1069c | Rv1069c | hypothetical protein |
| Rv0733 | adk | adenylate kinase |
| Rv3465 | rmlC | dTDP-4-dehydrorhamnose 3%2C5-epimerase |
| Rv3450c | eccB4 | ESX-4 secretion system protein EccB4 |
| Rv1271c | Rv1271c | hypothetical protein |
| Rv1960c | parD1 | antitoxin ParD1 |
| Rv3755c | Rv3755c | hypothetical protein |
| Rv0063a | Rv0063a | hypothetical protein |
| Rv2769c | PE27 | PE family protein PE27 |
| Rv3718c | Rv3718c | hypothetical protein |
| Rv1032c | trcS | two component sensor histidine kinase TrcS |
| Rv2779c | Rv2779c | Lrp/AsnC family transcriptional regulator |
| Rv0706 | rplV | 50S ribosomal protein L22 |
| Rv2422 | Rv2422 | hypothetical protein |
| Rv3719 | Rv3719 | hypothetical protein |
| Rv0503c | cmaA2 | cyclopropane mycolic acid synthase |
| Rv1387 | PPE20 | PPE family protein PPE20 |
| Rv2541 | Rv2541 | hypothetical protein |
| Rv3395c | Rv3395c | hypothetical protein |
| Rv2792c | Rv2792c | resolvase |
| Rv3273 | Rv3273 | transmembrane carbonic anhydrase |
| Rv2049c | Rv2049c | hypothetical protein |
| Rv0763c | Rv0763c | ferredoxin |
| Rv1097c | Rv1097c | hypothetical protein |
| Rv2316 | uspA | sugar ABC transporter permease UspA |
| Rv1783 | eccC5 | ESX-5 type VII secretion system protein EccC5 |
| Rv3219 | whiB1 | transcriptional regulator WhiB1 |
| Rvnt33 | cysU | 0 |
| Rv1928c | Rv1928c | short-chain type dehydrogenasereductase |
| Rv3800c | pks13 | polyketide synthase |
| Rv2547 | vapB19 | antitoxin VapB19 |
| Rv0175 | Rv0175 | Mce associated membrane protein |
| Rv1382 | Rv1382 | hypothetical protein |
| Rv1710 | scpB | segregation and condensation protein ScpB |
| Rv1006 | Rv1006 | hypothetical protein |
| Rv2871 | vapB43 | antitoxin VapB43 |
| Rv1831 | Rv1831 | hypothetical protein |
| Rv0497 | Rv0497 | transmembrane protein |
| Rv1178 | Rv1178 | aminotransferase |
| Rv1194c | Rv1194c | hypothetical protein |
| Rv0779c | Rv0779c | transmembrane protein |
| Rv3230c | Rv3230c | stearoyl-CoA 9-desaturase electron transfer protein |
| Rv1180 | pks3 | polyketide beta-ketoacyl synthase |
| Rv3140 | fadE23 | acyl-CoA dehydrogenase FadE23 |
| Rv3147 | nuoC | NADH-quinone oxidoreductase subunit C |
| Rv2236c | cobD | cobalamin biosynthesis transmembrane protein CobD |
| Rv1829 | Rv1829 | hypothetical protein |
| Rv3286c | sigF | RNA polymerase sigma factor SigF |
| Rv1319c | Rv1319c | adenylate cyclase |
| Rv0666 | Rv0666 | membrane protein |
| Rv2811 | Rv2811 | hypothetical protein |
| Rv0987 | Rv0987 | adhesion component ABC transporter permease |
| Rv1822 | pgsA2 | CDP-diacylglycerol--glycerol-3-phosphate 3-phosphatidyltransferase |
| Rv0391 | metZ | O-succinylhomoserine sulfhydrylase |
| Rv2765 | Rv2765 | hydrolase |
| Rv0452 | Rv0452 | transcriptional regulator |
| Rv3657c | Rv3657c | membrane protein |
| Rv3528c | Rv3528c | hypothetical protein |
| Rv1999c | Rv1999c | transporter |
| Rv2222c | glnA2 | glutamine synthetase |
| Rv1882c | Rv1882c | short-chain type dehydrogenasereductase |
| Rv3385c | vapB46 | antitoxin VapB46 |
| Rv2113 | Rv2113 | integral membrane protein |
| Rv2899c | fdhD | formate dehydrogenase accessory protein FdhD |
| Rv0668 | rpoC | DNA-directed RNA polymerase subunit beta' |
| Rv3878 | espJ | ESX-1 secretion-associated protein EspJ |
| Rv3197A | whiB7 | transcriptional regulator WhiB7 |
| Rv3447c | eccC4 | ESX-4 secretion system protein EccC4 |
| Rv3433c | Rv3433c | bifunctional ADP-dependent (S)-NAD(P)H-hydrate dehydrataseNAD(P)H-hydrate epimerase |
| Rv1405c | Rv1405c | methyltransferase |
| Rv3669 | Rv3669 | transmembrane protein |
| Rv1782 | eccB5 | ESX-5 type VII secretion system protein EccB5 |
| Rv3416 | whiB3 | redox-responsive transcriptional regulator WhiB3 |
| Rv3629c | Rv3629c | integral membrane protein |
| Rv3713 | cobQ2 | cobyric acid synthase CobQ |
| Rv2060 | Rv2060 | integral membrane protein |
| Rvnt12 | aspT | 0 |
| Rv2868c | gcpE | 4-hydroxy-3-methylbut-2-en-1-yl diphosphate synthase (flavodoxin) |
| Rv2120c | Rv2120c | integral membrane protein |
| Rv0463 | Rv0463 | membrane protein |
| Rv0839 | Rv0839 | hypothetical protein |
| Rv3869 | eccB1 | ESX-1 secretion system protein EccB |
| Rv0722 | rpmD | 50S ribosomal protein L30 |
| Rv0841 | Rv0841 | transmembrane protein |
| Rv0344c | lpqJ | lipoprotein LpqJ |
| Rv2063 | mazE7 | antitoxin MazE7 |
| Rv0309 | Rv0309 | hypothetical protein |
| Rv3763 | lpqH | lipoprotein LpqH |
| Rv0204c | Rv0204c | transmembrane protein |
| Rv0474 | Rv0474 | HTH-type transcriptional regulator |
| Rv2813 | Rv2813 | hypothetical protein |
| Rv0639 | nusG | transcription termination/antitermination protein NusG |
| Rv1494 | mazE4 | antitoxin MazE4 |
| Rv0679c | Rv0679c | hypothetical protein |
| Rv1568 | bioA | adenosylmethionine--8-amino-7-oxononanoate aminotransferase BioA |
| Rv1833c | Rv1833c | haloalkane dehalogenase |
| Rv0633c | Rv0633c | hypothetical protein |
| Rv2801A | mazE9 | antitoxin MazE9 |
| Rv3043c | ctaD | cytochrome C oxidase cytochrome 1 |
| Rv2317 | uspB | sugar ABC transporter permease UspB |
| Rv0023 | Rv0023 | transcriptional regulator |
| Rv3414c | sigD | ECF RNA polymerase sigma factor SigD |
| Rv3741c | Rv3741c | oxidoreductase |
| Rv1458c | Rv1458c | antibiotic ABC transporter ATP-binding protein |
| Rv0465c | Rv0465c | HTH-type transcriptional regulator |
| Rv3457c | rpoA | DNA-directed RNA polymerase subunit alpha |
| Rv0971c | echA7 | enoyl-CoA hydratase EchA7 |
| Rv0075 | Rv0075 | aminotransferase |
| Rv2445c | ndkA | nucleoside diphosphate kinase |
| Rv0196 | Rv0196 | HTH-type transcriptional regulator |
| Rv2551c | Rv2551c | hypothetical protein |
| Rv0436c | pssA | CDP-diacylglycerol--serine O-phosphatidyltransferase |
| Rv2985 | mutT1 | 8-oxo-dGTP diphosphatase |
| Rv1548c | PPE21 | PPE family protein PPE21 |
| Rv3881c | espB | ESX-1 secretion-associated protein EspB |
| Rv1238 | sugC | sugar ABC transporter ATP-binding protein SugC |
| Rv1344 | mbtL | acyl carrier protein MbtL |
| Rv1195 | PE13 | PE family protein PE13 |
| Rv1325c | PE_PGRS24 | PE-PGRS family protein PE_PGRS24 |
| Rv0909 | Rv0909 | antitoxin |
| Rv0323c | Rv0323c | hypothetical protein |
| Rv1173 | fbiC | FO synthase |
| Rv3321c | vapB44 | antitoxin VapB44 |
| Rv0395 | Rv0395 | hypothetical protein |
| Rv0819 | mshD | mycothiol acetyltransferase |
| Rv3558 | PPE64 | PPE family protein PPE64 |
| Rv1204c | Rv1204c | hypothetical protein |
| Rv0270 | fadD2 | fatty-acid--CoA ligase FadD2 |
| Rv1531 | Rv1531 | hypothetical protein |
| Rv0607 | Rv0607 | hypothetical protein |
| Rv3622c | PE32 | PE family protein PE32 |
| Rv1749c | Rv1749c | integral membrane protein |
| Rv3645 | Rv3645 | transmembrane protein |
| Rv0117 | oxyS | oxidative stress response regulatory protein OxyS |
| Rv0656c | vapC6 | ribonuclease VapC6 |
| Rv3178 | Rv3178 | nitroreductase |
| Rv2256c | Rv2256c | hypothetical protein |
| Rv3297 | nei | endonuclease VIII |
| Rv1880c | cyp140 | cytochrome P450 Cyp140 |
| Rv3581c | ispF | 2C-methyl-D-erythritol 2%2C4-cyclodiphosphate synthase |
| Rv0514 | Rv0514 | transmembrane protein |
| Rv1214c | PE14 | PE family protein PE14 |
| Rv3260c | whiB2 | transcriptional regulator WhiB2 |
| Rv2100 | Rv2100 | hypothetical protein |
| Rv1735c | Rv1735c | membrane protein |
| Rv0546c | Rv0546c | hypothetical protein |
| Rv1162 | narH | nitrate reductase subunit beta |
| Rv2711 | ideR | iron-dependent repressor and activator IdeR |
| Rv3045 | adhC | NADP-dependent alcohol dehydrogenase |
| Rv1291c | Rv1291c | hypothetical protein |
| Rv1470 | trxA | thioredoxin TrxA |
| Rv0691A | Rv0691A | mycofactocin precursor |
| Rv3095 | Rv3095 | HTH-type transcriptional regulator |
| Rv1885c | *MtCM | chorismate mutase |
| Rv1391 | dfp | bifunctional phosphopantothenoylcysteine decarboxylase/phosphopantothenate--cysteine ligase |
| Rv0235c | Rv0235c | transmembrane protein |
| Rv3445c | esxU | ESAT-6 like protein EsxU |
| Rv2667 | clpC2 | ATP-dependent protease ATP-binding subunit ClpC |
| Rv1474c | Rv1474c | transcriptional regulator |
| Rv3049c | Rv3049c | monooxygenase |
| Rv3708c | asd | aspartate-semialdehyde dehydrogenase |
| Rv1294 | thrA | homoserine dehydrogenase |
| Rv1217c | Rv1217c | tetronasin ABC transporter integral membrane protein |
| Rv1571 | Rv1571 | hypothetical protein |
| Rv1261c | Rv1261c | hypothetical protein |
| Rv0922 | Rv0922 | transposase |
| Rv0500A | Rv0500A | DNA-binding protein |
| Rv2545 | vapB18 | antitoxin VapB18 |
| Rv3324c | moaC3 | cyclic pyranopterin monophosphate synthase accessory protein |
| Rv2690c | Rv2690c | integral membrane protein |
| Rv1301 | Rv1301 | threonylcarbamoyl-AMP synthase |
| Rv2500c | fadE19 | acyl-CoA dehydrogenase FadE19 |
| Rv0476 | Rv0476 | transmembrane protein |
| Rv2162c | PE_PGRS38 | PE-PGRS family protein PE_PGRS38 |
| Rv1004c | Rv1004c | membrane protein |
| Rv1507A | Rv1507A | hypothetical protein |
| Rv0157 | pntB | NAD(P) transhydrogenase subunit beta PntB |
| Rv0916c | PE7 | PE family protein PE7 |
| Rv0500 | proC | pyrroline-5-carboxylate reductase |
| Rv3562 | fadE31 | acyl-CoA dehydrogenase FadE31 |
| Rv2590 | fadD9 | fatty-acid--CoA ligase FadD9 |
| Rv1119c | Rv1119c | hypothetical protein |
| Rv3437 | Rv3437 | transmembrane protein |
| Rv0157A | Rv0157A | hypothetical protein |
| Rv1073 | Rv1073 | hypothetical protein |
| Rv2640c | Rv2640c | ArsR family transcriptional regulator |
| Rv1087 | PE_PGRS21 | PE-PGRS family protein PE_PGRS21 |
| Rv0799c | Rv0799c | hypothetical protein |
| Rv0372c | Rv0372c | hypothetical protein |
| Rv3652 | PE_PGRS60 | PE-PGRS family-related protein PE_PGRS60 |
| Rv1815 | Rv1815 | hypothetical protein |
| Rv2587c | secD | protein translocase subunit SecD |
| Rv2102 | Rv2102 | hypothetical protein |
| Rv2988c | leuC | 3-isopropylmalate dehydratase large subunit |
| Rv1838c | vapC13 | ribonuclease VapC13 |
| Rv1519 | Rv1519 | hypothetical protein |
| Rv0738 | Rv0738 | hypothetical protein |
| Rv0699 | Rv0699 | hypothetical protein |
| Rv1560 | vapB11 | antitoxin VapB11 |
| Rv2934 | ppsD | phthiocerol synthesis polyketide synthase type I PpsD |
| Rv0215c | Rv0215c | Rv0215c |
| Rv0680c | Rv0680c | transmembrane protein |
| Rv1177 | fdxC | ferredoxin FdxC |
| Rv2503c | scoB | succinyl-CoA:3-ketoacid-CoA transferase subunit B |
| Rv0073 | Rv0073 | glutamine ABC transporter ATP-binding protein |
| Rv2228c | Rv2228c | multifunctional RNASE H/alpha-ribazole phosphatase/acid phosphatase |
| Rv3208A | TB9.4 | hypothetical protein |
| Rv1955 | higB | toxin HigB |
| Rv3653 | PE_PGRS61 | PE-PGRS family-related protein PE_PGRS61 |
| Rv0943c | Rv0943c | monooxygenase |
| Rv0617 | vapC29 | ribonuclease VapC29 |
| Rv3446c | Rv3446c | hypothetical protein |
| Rv1245c | Rv1245c | short-chain type dehydrogenasereductase |
| Rv2629 | Rv2629 | hypothetical protein |
| Rv0042c | Rv0042c | transcriptional regulator |
| Rv2897c | Rv2897c | hypothetical protein |
| Rv1812c | Rv1812c | dehydrogenase |
| Rv0038 | Rv0038 | hypothetical protein |
| Rv1570 | bioD | ATP-dependent dethiobiotin synthetase BioD |
| Rv2960c | Rv2960c | hypothetical protein |
| Rv1944c | Rv1944c | hypothetical protein |
| Rv2204c | Rv2204c | hypothetical protein |
| Rv1871c | Rv1871c | hypothetical protein |
| Rv0208c | Rv0208c | tRNA (guanine-N(7)-)-methyltransferase |
| Rv0684 | fusA1 | elongation factor G |
| Rv3075c | Rv3075c | hypothetical protein |
| Rv3865 | espF | ESX-1 secretion-associated protein EspF |
| Rvnt07 | metT | 0 |
| Rv0219 | Rv0219 | transmembrane protein |
| Rv3010c | pfkA | 6-phosphofructokinase |
| Rv2700 | Rv2700 | hypothetical protein |
| Rv3820c | papA2 | trehalose-2-sulfate acyltransferase |
| Rv2307A | Rv2307A | hypothetical protein |
| Rv0197 | Rv0197 | oxidoreductase |
| Rv1499 | Rv1499 | hypothetical protein |
| Rv0910 | Rv0910 | toxin |
| Rv1691 | Rv1691 | hypothetical protein |
| Rv0494 | Rv0494 | HTH-type transcriptional regulator |
| Rv1437 | pgk | phosphoglycerate kinase |
| Rv2606c | snzP | pyridoxine biosynthesis protein |
| Rv0043c | Rv0043c | HTH-type transcriptional regulator |
| Rv2408 | PE24 | PE family protein PE24 |
| Rv1383 | carA | carbamoyl-phosphate synthase small subunit |
| Rv1789 | PPE26 | PPE family protein PPE26 |
| Rv3248c | sahH | adenosylhomocysteinase |
| Rv0691c | Rv0691c | mycofactocin biosynthesis transcriptional regulator MftR |
| Rv0103c | ctpB | cation-transporter P-type ATPase B |
| Rv3264c | manB | D-alpha-D-mannose-1-phosphate guanylyltransferase ManB |
| Rv0161 | Rv0161 | oxidoreductase |
| Rv3252c | alkB | transmembrane alkane 1-monooxygenase AlkB |
| Rv0397A | Rv0397A | hypothetical protein |
| Rv3174 | Rv3174 | short-chain dehydrogenase/reductase |
| Rv1612 | trpB | tryptophan synthase subunit beta |
| Rv1263 | amiB2 | amidase AmiB |
| Rv3407 | vapB47 | antitoxin VapB47 |
| RVnc0046 | ssr | #N/A |
| Rv2123 | PPE37 | PPE family protein PPE37 |
| Rv0645c | mmaA1 | mycolic acid methyltransferase MmaA1 |
| Rv1434 | Rv1434 | hypothetical protein |
| Rv0239 | vapB24 | antitoxin VapB24 |
| Rv2293c | Rv2293c | hypothetical protein |
| Rv0205 | Rv0205 | transmembrane protein |
| Rv2751 | Rv2751 | hypothetical protein |
| Rv3547 | ddn | deazaflavin-dependent nitroreductase |
| Rv0214 | fadD4 | fatty-acid--CoA ligase FadD4 |
| Rv3870 | eccCa1 | ESX-1 secretion system protein EccCa |
| Rv0757 | phoP | two component system response transcriptional positive regulator PhoP |
| Rv2450c | rpfE | resuscitation-promoting factor RpfE |
| Rv2944 | Rv2944 | insertion sequence element IS1533 transposase |
| Rv3398c | idsA1 | multifunctional dimethylallyltransferasegeranyltranstransferasefarnesyltranstransferase |
| Rv1628c | Rv1628c | hypothetical protein |
| Rv3734c | tgs2 | diacyglycerol O-acyltransferase |
| Rv1968 | mce3C | Mce family protein Mce3C |
| Rv0261c | narK3 | nitratenitrite transporter |
| Rv3896c | Rv3896c | hypothetical protein |
| Rv2007c | fdxA | ferredoxin |
| Rv1554 | frdC | fumarate reductase membrane anchor subunit |
| Rv3567c | hsaB | flavin-dependent monooxygenase reductase subunit HsaB |
| Rv2695 | Rv2695 | hypothetical protein |
| Rv3267 | Rv3267 | hypothetical protein |
| Rv0155 | pntAa | NAD(P) transhydrogenase subunit alpha PntAa |
| Rv3871 | eccCb1 | ESX-1 secretion system protein EccCb |
| Rv0628c | Rv0628c | hypothetical protein |
| Rv0515 | Rv0515 | hypothetical protein |
| Rv2734 | Rv2734 | hypothetical protein |
| Rv2439c | proB | glutamate 5-kinase protein |
| Rv0238 | Rv0238 | transcriptional regulator |
| Rv1547 | dnaE1 | DNA polymerase III subunit alpha |
| Rv2498c | citE | citrate (pro-3S)-lyase subunit beta |
| Rv0224c | Rv0224c | methyltransferase |
| Rv2778c | Rv2778c | hypothetical protein |
| Rv1330c | pncB1 | nicotinic acid phosphoribosyltransferase PncB1 |
| Rv3902c | Rv3902c | hypothetical protein |
| Rv1821 | secA2 | accessory Sec system translocase SecA2 |
| Rv2154c | ftsW | lipid II flippase FtsW |
| Rv3012c | gatC | glutamyl-tRNA(GLN) amidotransferase subunit C |
| Rv0695 | Rv0695 | mycofactocin system creatinine amidohydrolase family protein MftE |
| Rv0414c | thiE | thiamine-phosphate synthase |
| Rv2367c | Rv2367c | endoribonuclease |
| Rv3101c | ftsX | cell division protein FtsX |
| Rv1728c | Rv1728c | hypothetical protein |
| Rv1426c | lipO | esterase LipO |
| Rv0665 | vapC8 | ribonuclease VapC8 |
| Rv1224 | tatB | Sec-independent protein translocase protein TatB |
| Rv0307c | Rv0307c | hypothetical protein |
| Rv1800 | PPE28 | PPE family protein PPE28 |
| Rv2625c | Rv2625c | zinc metalloprotease Rip3 |
| Rv1918c | PPE35 | PPE family protein PPE35 |
| Rv1912c | fadB5 | oxidoreductase FadB |
| Rv3134c | Rv3134c | universal stress protein |
| Rv0601c | Rv0601c | two component sensor kinase HK2 |
| Rv2560 | Rv2560 | hypothetical protein |
| Rv3787c | Rv3787c | S-adenosyl-L-methionine-dependent methyltransferase |
| Rv2243 | fabD | malonyl CoA-acyl carrier protein transacylase |
| Rv1208 | gpgS | glucosyl-3-phosphoglycerate synthase |
| Rv3915 | cwlM | peptidoglycan hydrolase |
| Rv1796 | mycP5 | membrane-anchored mycosin MycP |
| Rv1607 | chaA | ionic transporter integral membrane protein ChaA |
| Rv3722c | Rv3722c | hypothetical protein |
| Rv2438c | nadE | glutamine-dependent NAD(+) synthetase |
| Rv1954c | Rv1954c | hypothetical protein |
| Rv1110 | lytB2 | 4-hydroxy-3-methylbut-2-enyl diphosphate reductase |
| Rv0672 | fadE8 | acyl-CoA dehydrogenase FadE8 |
| Rv2091c | Rv2091c | membrane protein |
| Rv3632 | Rv3632 | membrane protein |
| Rv0355c | PPE8 | PPE family protein PPE8 |
| Rv2359 | zur | zinc uptake regulation protein |
| Rv3714c | Rv3714c | hypothetical protein |
| Rv3779 | Rv3779 | transmembrane protein |
| Rv2531c | adi | amino acid decarboxylase |
| Rv2237A | Rv2237A | hypothetical protein |
| Rv1158c | Rv1158c | hypothetical protein |
| Rv1686c | Rv1686c | ABC transporter permease |
| Rv3808c | glfT2 | galactofuranosyl transferase GlfT |
| Rv1677 | dsbF | lipoprotein DsbF |
| Rv0096 | PPE1 | PPE family protein PPE1 |
| Rv3874 | esxB | ESAT-6-like protein EsxB |
| Rv1567c | Rv1567c | membrane protein |
| Rv2530c | vapC39 | ribonuclease VapC39 |
| Rv0806c | cpsY | exopolysaccharide phosphotransferase CpsY |
| Rv0064 | Rv0064 | transmembrane protein |
| Rv3738c | PPE66 | PPE family protein PPE66 |
| Rv3895c | eccB2 | ESX-2 secretion system protein EccB |
| Rv3246c | mtrA | two component DNA-binding response regulator MtrA |
| Rv3668c | Rv3668c | protease |
| Rv3679 | Rv3679 | anion transporter ATPase |
| Rv3709c | ask | aspartokinase |
| Rv2633c | Rv2633c | hypothetical protein |
| Rv0308 | Rv0308 | integral membrane protein |
| Rv2303c | Rv2303c | antibiotic-resistance protein |
| Rv0294 | tam | trans-aconitate methyltransferase |
| Rv2554c | Rv2554c | Holliday junction resolvase |
| Rv1967 | mce3B | Mce family protein Mce3B |
| Rv1516c | Rv1516c | sugar transferase |
| Rv3846 | sodA | superoxide dismutase |
| Rv0650 | Rv0650 | sugar kinase |
| Rv1891 | Rv1891 | hypothetical protein |
| Rv2831 | echA16 | enoyl-CoA hydratase EchA16 |
| Rv3107c | agpS | alkyldihydroxyacetonephosphate synthase |
| Rv1189 | sigI | ECF RNA polymerase sigma factor SigI |
| Rv2771c | Rv2771c | hypothetical protein |
| Rv2022c | Rv2022c | hypothetical protein |
| Rv2790c | ltp1 | lipid-transfer protein |
| Rv0549c | vapC3 | ribonuclease VapC3 |
| Rv1082 | mca | mycothiol S-conjugate amidase |
| Rv2752c | Rv2752c | ribonuclease J |
| Rv2163c | pbpB | penicillin-binding membrane protein PbpB |
| Rv0731c | Rv0731c | S-adenosylmethionine-dependent methyltransferase |
| Rvnt24 | metV | 0 |
| Rv1631 | coaE | dephospho-CoA kinase CoaE |
| Rv2942 | mmpL7 | transmembrane transport protein MmpL7 |
| Rv3796 | atsH | hypothetical protein |
| Rv0981 | mprA | two-component response regulator MrpA |
| Rv0694 | Rv0694 | mycofactocin system heme/flavin oxidoreductase MftD |
| Rv0689c | Rv0689c | hypothetical protein |
| Rv3099c | Rv3099c | hypothetical protein |
| Rv1018c | glmU | bifunctional UDP-N-acetylglucosamine pyrophosphorylaseglucosamine-1-phosphate N-acetyltransferase |
| Rv0596c | vapB4 | antitoxin VapB4 |
| Rv3841 | bfrB | bacterioferritin BfrB |
| Rv2053c | fxsA | transmembrane protein FxsA |
| Rv2504c | scoA | succinyl-CoA:3-ketoacid-CoA transferase subunit A |
| Rv3086 | adhD | alcohol dehydrogenase D |
| Rv1483 | fabG1 | 3-oxoacyl-ACP reductase FabG |
| Rv0234c | gabD1 | succinate-semialdehyde dehydrogenase |
| Rv0418 | lpqL | lipoprotein aminopeptidase LpqL |
| Rv0751c | mmsB | 3-hydroxyisobutyrate dehydrogenase |
| Rv2830c | vapB22 | antitoxin VapB22 |
| Rv0556 | Rv0556 | transmembrane protein |
| Rv0513 | Rv0513 | transmembrane protein |
| Rv0589 | mce2A | Mce family protein Mce2A |
| Rv2401 | Rv2401 | hypothetical protein |
| Rv1736c | narX | nitrate reductase-like protein NarX |
| Rv2682c | dxs1 | 1-deoxy-D-xylulose 5-phosphate synthase |
| Rv1541c | lprI | lipoprotein LprI |
| Rv0540 | Rv0540 | hypothetical protein |
| Rv2285 | Rv2285 | diacylglycerol acyltransferase |
| Rv3576 | lppH | lipoprotein LppH |
| Rv1411c | lprG | lipoprotein LprG |
| Rv1620c | cydC | cytochrome biosyntheisis ABC transporter ATP-binding proteinpermease CydC |
| Rv0456A | mazF1 | toxin MazF1 |
| Rv1965 | yrbE3B | integral membrane protein |
| Rv2350c | plcB | membrane-associated phospholipase B |
| Rv1866 | Rv1866 | hypothetical protein |
| Rv3769 | Rv3769 | hypothetical protein |
| Rv0116c | ldtA | L%2CD-transpeptidase LdtA |
| Rv0222 | echA1 | enoyl-CoA hydratase EchA1 |
| Rv0453 | PPE11 | PPE family protein PPE11 |
| Rv2260 | Rv2260 | hypothetical protein |
| Rv1407 | fmu | 16S rRNA m5C967 methyltransferase |
| Rv0530 | Rv0530 | hypothetical protein |
| Rv2183c | Rv2183c | hypothetical protein |
| Rv2229c | Rv2229c | hypothetical protein |
| Rv1717 | Rv1717 | hypothetical protein |
| Rv0057 | Rv0057 | hypothetical protein |
| Rv3444c | esxT | ESAT-6 like protein EsxT |
| Rv3520c | Rv3520c | coenzyme F420-dependent oxidoreductase |
| Rv1964 | yrbE3A | integral membrane protein |
| Rv2697c | dut | deoxyuridine 5'-triphosphate nucleotidohydrolase |
| Rv0047c | Rv0047c | hypothetical protein |
| Rv3145 | nuoA | NADH-quinone oxidoreductase subunit A |
| Rv2369c | Rv2369c | hypothetical protein |
| Rv0410c | pknG | serine/threonine-protein kinase PknG |
| Rv2490a | Rv2490a | hypothetical protein |
| Rv3891c | esxD | ESAT-6 like protein EsxD |
| Rv1435c | Rv1435c | hypothetical protein |
| Rv3285 | accA3 | bifunctional protein acetyl-propionyl-CoA carboxylase subunit alpha AccA |
| Rv1227c | Rv1227c | transmembrane protein |
| Rv3488 | Rv3488 | hypothetical protein |
| Rv0243 | fadA2 | acetyl-CoA acetyltransferase FadA |
| Rv3294c | Rv3294c | hypothetical protein |
| Rv3503c | fdxD | ferredoxin FdxD |
| Rv0299 | Rv0299 | toxin |
| Rv1505c | Rv1505c | hypothetical protein |
| Rv3208 | Rv3208 | TetR family transcriptional regulator |
| Rv2518c | ldtB | L%2CD-transpeptidase LdtB |
| Rv0868c | moaD2 | cyclic pyranopterin monophosphate synthase |
| Rv0712 | Rv0712 | hypothetical protein |
| Rv3648c | cspA | cold shock protein A |
| Rv1209 | Rv1209 | hypothetical protein |
| Rv1970 | lprM | Mce family lipoprotein LprM |
| Rv0686 | Rv0686 | membrane protein |
| Rv3021c | PPE47 | Rv3021c |
| Rv0846c | Rv0846c | oxidase |
| Rv0432 | sodC | superoxide dismutase |
| Rv2825c | Rv2825c | hypothetical protein |
| Rv0331 | Rv0331 | dehydrogenase/reductase |
| Rv0927c | Rv0927c | oxidoreductase |
| Rv1410c | P55 | aminoglycosides/tetracycline-transport integral membrane protein |
| Rv1774 | Rv1774 | oxidoreductase |
| Rv2761c | hsdS | type I restriction/modification system specificity determinant HsdS |
| Rv3658c | Rv3658c | transmembrane protein |
| Rv0884c | serC | phosphoserine aminotransferase |
| Rv0834c | PE_PGRS14 | PE-PGRS family protein PE_PGRS14 |
| Rv0648 | Rv0648 | alpha-mannosidase |
| Rv1974 | Rv1974 | membrane protein |
| Rv0127 | mak | maltokinase |
| Rv1971 | mce3F | Mce family protein Mce3F |
| Rv3148 | nuoD | NADH-quinone oxidoreductase subunit D |
| Rv3400 | Rv3400 | hydrolase |
| Rv2527 | vapC17 | ribonuclease VapC17 |
| Rvnt02 | alaT | 0 |
| Rv1630 | rpsA | 30S ribosomal protein S1 |
| Rv1332 | Rv1332 | transcriptional regulator |
| Rv3057c | Rv3057c | short chain alcohol dehydrogenasereductase |
| Rv2928 | tesA | thioesterase TesA |
| Rv1160 | mutT2 | 8-oxo-dGTP diphosphatase |
| Rv2491 | Rv2491 | hypothetical protein |
| Rv3415c | Rv3415c | hypothetical protein |
| Rv3025c | iscS | cysteine desulfurase |
| Rv2627c | Rv2627c | hypothetical protein |
| Rv0427c | xthA | exodeoxyribonuclease III protein XthA |
| Rv1803c | PE_PGRS32 | PE-PGRS family protein PE_PGRS32 |
| Rv3822 | Rv3822 | hypothetical protein |
| Rv3435c | Rv3435c | transmembrane protein |
| Rv2617c | Rv2617c | transmembrane protein |
| Rv3428c | Rv3428c | transposase |
| Rv1899c | lppD | lipoprotein LppD |
| Rv3880c | espL | ESX-1 secretion-associated protein EspL |
| Rv0653c | Rv0653c | transcriptional regulator |
| Rv2395A | aprA | acid and phagosome regulated protein AprA |
| Rv2687c | Rv2687c | antibiotic ABC transporter permease |
| Rv2302 | Rv2302 | hypothetical protein |
| Rv3593 | lpqF | lipoprotein LpqF |
| Rv3462c | infA | translation initiation factor IF-1 |
| Rv3564 | fadE33 | acyl-CoA dehydrogenase FadE33 |
| Rv0677c | mmpS5 | membrane protein MmpS5 |
| Rv1837c | glcB | malate synthase |
| Rv2409c | Rv2409c | hypothetical protein |
| Rv3590c | PE_PGRS58 | PE-PGRS family protein PE_PGRS58 |
| Rv1743 | pknE | serine/threonine-protein kinase PknE |
| Rv2694c | Rv2694c | hypothetical protein |
| Rv0941c | Rv0941c | hypothetical protein |
| Rv2637 | dedA | transmembrane protein DedA |
| Rv0248c | Rv0248c | succinate dehydrogenase flavoprotein subunit |
| Rv2940c | mas | multifunctional mycocerosic acid synthase |
| Rv1375 | Rv1375 | hypothetical protein |
| Rv0991c | Rv0991c | hypothetical protein |
| Rv1634 | Rv1634 | multidrug-efflux transporter |
| Rv0635 | hadA | (3R)-hydroxyacyl-ACP dehydratase subunit HadA |
| Rv0404 | fadD30 | long-chain-fatty-acid--AMP ligase FadD30 |
| Rv2991 | Rv2991 | hypothetical protein |
| Rv3549c | Rv3549c | short-chain type dehydrogenasereductase |
| Rv0760c | Rv0760c | hypothetical protein |
| Rv1422 | Rv1422 | hypothetical protein |
| Rv3192 | Rv3192 | hypothetical protein |
| Rv3561 | fadD3 | fatty-acid--CoA ligase FadD3 |
| Rv1103c | mazE3 | antitoxin MazE3 |
| Rv2448c | valS | valine--tRNA ligase |
| Rv2656c | Rv2656c | prophage protein |
| Rv0859 | Rv0859 | acyltransferase |
| Rv1024 | Rv1024 | membrane protein |
| Rv0168 | yrbE1B | membrane protein |
| Rv2744c | 35kd_ag | hypothetical protein |
| Rv0925c | Rv0925c | hypothetical protein |
| Rv0100 | Rv0100 | hypothetical protein |
| Rv0848 | cysK2 | cysteine synthase CysK |
| Rv3245c | mtrB | two component sensory histidine kinase MtrB |
| Rv3643 | Rv3643 | hypothetical protein |
| Rv1799 | lppT | lipoprotein LppT |
| Rv1618 | tesB1 | acyl-CoA thioesterase II |
| Rv1447c | zwf2 | glucose-6-phosphate 1-dehydrogenase |
| Rv2253 | Rv2253 | hypothetical protein |
| Rv0428c | Rv0428c | GCN5-like N-acetyltransferase |
| Rv3862c | whiB6 | transcriptional regulator WhiB6 |
| Rv2733c | Rv2733c | (dimethylallyl)adenosine tRNA methylthiotransferase |
| Rv0188 | Rv0188 | transmembrane protein |
| Rv2221c | glnE | [glutamate--ammonia-ligase] adenylyltransferase |
| Rv3750c | Rv3750c | excisionase |
| Rv3151 | nuoG | NADH-quinone oxidoreductase subunit G |
| Rv0735 | sigL | ECF RNA polymerase sigma factor SigL |
| Rv3742c | Rv3742c | oxidoreductase |
| Rv0748 | vapB31 | antitoxin VapB31 |
| Rv3597c | lsr2 | iron-regulated H-NS-like protein |
| Rv2094c | tatA | Sec-independent protein translocase membrane-bound protein TatA |
| Rv2742c | Rv2742c | hypothetical protein |
| Rv0227c | Rv0227c | membrane protein |
| Rv0160c | PE4 | PE family protein PE4 |
| Rv1174c | TB8.4 | low molecular weight T-cell antigen |
| Rv0220 | lipC | esterase LipC |
| Rv0022c | whiB5 | transcriptional regulator WhiB5 |
| Rv0317c | glpQ2 | glycerophosphoryl diester phosphodiesterase GlpQ |
| Rv1288 | Rv1288 | hypothetical protein |
| Rv0392c | ndhA | NADH dehydrogenase NdhA |
| Rv2384 | mbtA | 2%2C3-dihydroxybenzoate-AMP ligase |
| Rv0506 | mmpS2 | membrane protein MmpS2 |
| Rv1887a | Rv1887a | Rv1887a |
| Rv3811 | csp | hypothetical protein |
| Rv2224d | Rv2224d | hypothetical protein |
| Rv3900c | Rv3900c | hypothetical protein |
| Rv0363c | fba | fructose-bisphosphate aldolase |
| Rv1460 | Rv1460 | transcriptional regulator |
| Rv0570 | nrdZ | vitamin B12-dependent ribonucleoside-diphosphate reductase |
| Rv0118c | oxcA | oxalyl-CoA decarboxylase OxcA |
| Rv3694c | Rv3694c | transmembrane protein |
| Rv3006 | lppZ | lipoprotein LppZ |
| Rv2812 | Rv2812 | transposase |
| Rv1010 | ksgA | rRNA small subunit methyltransferase A |
| Rv2893 | Rv2893 | oxidoreductase |
| Rv2312 | Rv2312 | hypothetical protein |
| Rv0586 | mce2R | HTH-type transcriptional regulator Mce2R |
| Rv3818 | Rv3818 | hypothetical protein |
| Rv0777 | purB | adenylosuccinate lyase PurB |
| Rv1534 | Rv1534 | transcriptional regulator |
| Rv2265 | Rv2265 | integral membrane protein |
| Rv3526 | kshA | 3-ketosteroid-9-alpha-monooxygenase oxygenase subunit |
| Rv0242c | fabG4 | 3-oxoacyl-ACP reductase FabG |
| Rv1219c | Rv1219c | transcriptional regulator |
| Rv3430c | Rv3430c | transposase |
| Rv0371c | Rv0371c | hypothetical protein |
| Rv3833 | Rv3833 | AraC family transcriptional regulator |
| Rv0643c | mmaA3 | methoxy mycolic acid synthase MmaA3 |
| Rv0036c | Rv0036c | hypothetical protein |
| Rv0747 | PE_PGRS10 | PE-PGRS family protein PE_PGRS10 |
| Rv0225 | Rv0225 | hypothetical protein |
| Rv2843 | Rv2843 | hypothetical protein |
| Rv3735 | Rv3735 | hypothetical protein |
| Rv2145c | wag31 | cell wall synthesis protein Wag31 |
| Rv2244 | acpM | meromycolate extension acyl carrier protein |
| Rv2489c | Rv2489c | hypothetical protein |
| Rv3767c | Rv3767c | S-adenosylmethionine-dependent methyltransferase |
| Rv1706A | Rv1706A | hypothetical protein |
| Rv1233c | Rv1233c | hypothetical protein |
| Rv2365c | Rv2365c | hypothetical protein |
| Rv3455c | truA | tRNA pseudouridine synthase A |
| Rv2305 | Rv2305 | hypothetical protein |
| Rv3706c | Rv3706c | hypothetical protein |
| Rv2225 | panB | 3-methyl-2-oxobutanoate hydroxymethyltransferase |
| Rv2208 | cobS | adenosylcobinamide-GDP ribazoletransferase |
| Rv1402 | priA | primosomal protein N' |
| Rv1760 | Rv1760 | diacylglycerol acyltransferase |
| Rvnt13 | pheU | 0 |
| Rv2469c | Rv2469c | hypothetical protein |
| Rv0995 | rimJ | ribosomal-protein-alanine acetyltransferase RimJ |
| Rv1996 | Rv1996 | universal stress protein |
| RVnc0034 | MTS0858 | 0 |
| Rv1771 | Rv1771 | L-gulono-1%2C4-lactone dehydrogenase |
| Rv2307D | Rv2307D | hypothetical protein |
| Rv0422c | thiD | hydroxymethylpyrimidine/phosphomethylpyrimidine kinase |
| Rv0477 | Rv0477 | hypothetical protein |
| Rv3353c | Rv3353c | hypothetical protein |
| Rv3456c | rplQ | 50S ribosomal protein L17 |
| Rv3621c | PPE65 | PPE family protein PPE65 |
| Rv0989c | grcC2 | polyprenyl-diphosphate synthase GrcC |
| Rv2883c | pyrH | uridylate kinase |
| Rv0772 | purD | phosphoribosylamine--glycine ligase |
| Rv1343c | lprD | lipoprotein LprD |
| Rv2581c | Rv2581c | glyoxalase II |
| Rv1283c | oppB | oligopeptide ABC transporter permease OppB |
| Rv0318c | Rv0318c | integral membrane protein |
| Rv2374c | hrcA | heat-inducible transcription repressor HrcA |
| Rv1712 | cmk | cytidylate kinase |
| Rv0236A | Rv0236A | hypothetical protein |
| Rv1655 | argD | acetylornithine aminotransferase |
| Rv0818 | Rv0818 | transcriptional regulator |
| Rv2490c | PE_PGRS43 | PE-PGRS family protein PE_PGRS43 |
| Rv3191c | Rv3191c | transposase |
| Rv0325 | Rv0325 | hypothetical protein |
| Rv2564 | glnQ | glutamine ABC transporter ATP-binding protein |
| Rv3164c | moxR3 | methanol dehydrogenase transcriptional regulator MoxR |
| Rv1798 | eccA5 | ESX-5 type VII secretion system protein EccA |
| Rv1079 | metB | cystathionine gamma-synthase |
| Rv0437c | psd | phosphatidylserine decarboxylase |
| Rv3307 | deoD | purine nucleoside phosphorylase |
| Rv0707 | rpsC | 30S ribosomal protein S3 |
| Rv1099c | glpX | fructose 1%2C6-bisphosphatase |
| Rv1276c | Rv1276c | hypothetical protein |
| Rv3272 | Rv3272 | hypothetical protein |
| Rv1013 | pks16 | polyketide synthase |
| Rv3834c | serS | serine--tRNA ligase |
| Rv1726 | Rv1726 | oxidoreductase |
| Rv3098A | Rv3098A | PemK-like protein |
| Rv2201 | asnB | asparagine synthetase |
| Rv1879 | Rv1879 | hypothetical protein |
| Rv1289 | Rv1289 | hypothetical protein |
| Rv2170 | Rv2170 | GCN5-like N-acetyltransferase |
| Rv3007c | Rv3007c | oxidoreductase |
| Rv2152c | murC | UDP-N-acetylmuramate--alanine ligase |
| Rv0280 | PPE3 | PPE family protein PPE3 |
| Rv0956 | purN | phosphoribosylglycinamide formyltransferase PurN |
| Rv1856c | Rv1856c | oxidoreductase |
| Rv3257c | pmmA | phosphomannomutase PmmA |
| Rv0451c | mmpS4 | membrane protein MmpS4 |
| Rv1759c | wag22 | PE-PGRS family protein Wag22 |
| Rv2540c | aroF | chorismate synthase |
| Rv0351 | grpE | stress response protein GrpE |
| Rv2165c | Rv2165c | rRNA small subunit methyltransferase H |
| Rv2314c | Rv2314c | hypothetical protein |
| Rv1975 | Rv1975 | hypothetical protein |
| Rv3282 | Rv3282 | hypothetical protein |
| Rv1210 | tagA | DNA-3-methyladenine glycosylase I TagA |
| Rv0312 | Rv0312 | hypothetical protein |
| Rv3566c | nat | arylamine N-acetyltransferase |
| Rv3161c | Rv3161c | dioxygenase |
| Rv1590 | Rv1590 | hypothetical protein |
| Rv2062c | cobN | cobalamin biosynthesis protein CobN |
| Rv2428 | ahpC | alkyl hydroperoxide reductase subunit AhpC |
| Rv2388c | hemN | oxygen-independent coproporphyrinogen III oxidase |
| Rv0423c | thiC | phosphomethylpyrimidine synthase |
| Rv1126c | Rv1126c | hypothetical protein |
| Rv3473c | bpoA | peroxidase BpoA |
| Rv1243c | PE_PGRS23 | PE-PGRS family protein PE_PGRS23 |
| Rv3172c | Rv3172c | hypothetical protein |
| Rv2455c | Rv2455c | 2-oxoglutarate oxidoreductase subunit KorA |
| Rv1454c | qor | quinone reductase |
| Rv1475c | acn | iron-regulated aconitate hydratase |
| Rv2915c | Rv2915c | hypothetical protein |
| Rv1843c | guaB1 | inosine-5'-monophosphate dehydrogenase |
| Rv2034 | Rv2034 | ArsR family HTH-type transcriptional repressor |
| Rv1181 | pks4 | polyketide beta-ketoacyl synthase |
| Rv2626c | hrp1 | hypoxic response protein |
| Rv0086 | hycQ | hydrogenase HycQ |
| Rv2024c | Rv2024c | hypothetical protein |
| Rv3378c | Rv3378c | diterpene synthase |
| Rv0557 | mgtA | GDP-mannose-dependent alpha-mannosyltransferase |
| Rv3665c | dppB | dipeptide ABC transporter permease DppB |
| Rv2975c | Rv2975c | hypothetical protein |
| Rv2869c | rip | zinc metalloprotease |
| Rv2488c | Rv2488c | LuxR family transcriptional regulator |
| Rv0784 | Rv0784 | hypothetical protein |
| Rv1727 | Rv1727 | hypothetical protein |
| Rv2227 | Rv2227 | hypothetical protein |
| Rv2525c | Rv2525c | hypothetical protein |
| Rv2169c | Rv2169c | transmembrane protein |
| Rv2688c | Rv2688c | antibiotic ABC transporter ATP-binding protein |
| Rv1579c | Rv1579c | phage protein |
| Rv0183 | Rv0183 | lysophospholipase |
| Rv2990c | Rv2990c | hypothetical protein |
| Rv3675 | Rv3675 | membrane protein |
| Rv3618 | Rv3618 | monooxygenase |
| Rv2058c | rpmB2 | 50S ribosomal protein L28 |
| Rv0478 | deoC | 2-deoxyribose-5-phosphate aldolase |
| Rv1373 | Rv1373 | glycolipid sulfotransferase |
| Rv1836c | Rv1836c | hypothetical protein |
| Rv3711c | dnaQ | DNA polymerase III subunit epsilon |
| Rv0434 | Rv0434 | hypothetical protein |
| Rv3656c | Rv3656c | hypothetical protein |
| Rv3782 | glfT1 | galactofuranosyl transferase GlfT |
| Rv3883c | mycP1 | membrane-anchored mycosin |
| Rv2475c | Rv2475c | hypothetical protein |
| Rv0441c | Rv0441c | hypothetical protein |
| Rv1008 | tatD | deoxyribonuclease TatD |
| Rv1915 | aceAa | isocitrate lyase AceAa |
| Rv2925c | rnc | ribonuclease III |
| Rv3873 | PPE68 | PPE family protein PPE68 |
| Rv3236c | kefB | integral membrane transport protein |
| Rv2924c | fpg | formamidopyrimidine-DNA glycosylase |
| Rv2494 | vapC38 | ribonuclease VapC38 |
| Rv2462c | tig | trigger factor |
| Rv2728c | Rv2728c | hypothetical protein |
| Rv2902c | rnhB | ribonuclease HII |
| Rv3022c | PPE48 | Rv3022c |
| Rv1201c | dapD | 2%2C3%2C4%2C5-tetrahydropyridine-2%2C6-dicarboxylate N-succinyltransferase |
| Rv2291 | sseB | thiosulfate sulfurtransferase SseB |
| Rv2005c | Rv2005c | universal stress protein |
| Rv3243c | Rv3243c | hypothetical protein |
| Rv1699 | pyrG | CTP synthase |
| Rv1722 | Rv1722 | carboxylase |
| Rv1329c | dinG | ATP-dependent helicase DinG |
| Rv1262c | Rv1262c | hypothetical protein |
| Rv1324 | Rv1324 | thioredoxin |
| Rv3269 | Rv3269 | hypothetical protein |
| Rv0594 | mce2F | Mce family protein Mce2F |
| Rv3195 | Rv3195 | hypothetical protein |
| Rv1767 | Rv1767 | hypothetical protein |
| Rv3563 | fadE32 | acyl-CoA dehydrogenase FadE32 |
| Rv3281 | accE5 | bifunctional protein acetyl-propionyl-CoAcarboxylase subunit epsilon AccE |
| Rv1659 | argH | argininosuccinate lyase |
| Rv2583c | relA | bifunctional (p)ppGpp synthase/hydrolase RelA |
| Rv2099c | PE21 | Rv2099c |
| Rv0562 | grcC1 | polyprenyl-diphosphate synthase GrcC |
| Rv0678 | Rv0678 | hypothetical protein |
| Rv0361 | Rv0361 | membrane protein |
| Rv1248c | Rv1248c | multifunctional 2-oxoglutarate dehydrogenase E1 component /2-oxoglutarate dehydrogenase dihydrolipoyllysine-residue succinyltransferase |
| Rv0962c | lprP | lipoprotein LprP |
| Rv1205 | Rv1205 | hypothetical protein |
| Rv1326c | glgB | 1%2C4-alpha-glucan branching protein |
| Rvnr03 | rrf | 0 |
| Rv2643 | arsC | arsenic-transport integral membrane protein ArsC |
| Rv3324A | Rv3324A | Rv3324A |
| Rv1589 | bioB | biotin synthetase |
| Rv2532c | Rv2532c | hypothetical protein |
| Rv3545c | cyp125 | steroid C26-monooxygenase |
| Rv1984c | cfp21 | cutinase |
| Rv2021c | Rv2021c | transcriptional regulator |
| Rv1486c | Rv1486c | hypothetical protein |
| Rv1039c | PPE15 | PPE family protein PPE15 |
| Rv3253c | Rv3253c | cationic amino acid transport integral membrane protein |
| Rv0381c | Rv0381c | hypothetical protein |
| Rv0716 | rplE | 50S ribosomal protein L5 |
| Rv2839c | infB | translation initiation factor IF-2 |
| Rv1027c | kdpE | transcriptional regulator KdpE |
| Rv3029c | fixA | electron transfer flavoprotein subunit beta |
| Rv0813c | Rv0813c | hypothetical protein |
| Rv1133c | metE | 5-methyltetrahydropteroyltriglutamate--homocysteine methyltransferase |
| Rv0433 | Rv0433 | carboxylate-amine ligase |
| Rv0611c | Rv0611c | hypothetical protein |
| Rv1532c | Rv1532c | hypothetical protein |
| Rv0257 | Rv0257 | hypothetical protein |
| Rv1723 | Rv1723 | hydrolase |
| Rv1864c | Rv1864c | hypothetical protein |
| Rv1923 | lipD | lipase LipD |
| Rv3570c | hsaA | flavin-dependent monooxygenase oxygenase subunit HsaA |
| Rv1566c | Rv1566c | hypothetical protein |
| Rv2181 | Rv2181 | alpha-(1-2)-phosphatidylinositol mannoside mannosyltransferase |
| Rv1577c | Rv1577c | phage prohead protease |
| Rv0216 | Rv0216 | hydratase |
| Rv0793 | Rv0793 | monooxygenase |
| Rv3299c | atsB | arylsulfatase AtsB |
| Rv2614c | thrS | threonine--tRNA ligase |
| Rv3499c | mce4A | Mce family protein Mce4A |
| Rv1064c | lpqV | lipoprotein LpqV |
| Rv3863 | Rv3863 | hypothetical protein |
| Rv3573c | fadE34 | acyl-CoA dehydrogenase FadE34 |
| Rv3397c | phyA | phytoene synthase |
| Rv3671c | Rv3671c | serine protease |
| Rv1156 | Rv1156 | hypothetical protein |
| Rv3650 | PE33 | PE family protein PE33 |
| Rv1228 | lpqX | lipoprotein LpqX |
| Rv0288 | esxH | ESAT-6-like protein EsxH |
| Rv2660c | Rv2660c | hypothetical protein |
| Rv0126 | treS | trehalose synthaseamylase TreS |
| Rv3290c | lat | L-lysine-epsilon aminotransferase |
| Rv2031c | hspX | alpha-crystallin |
| Rv1561 | vapC11 | ribonuclease VapC11 |
| Rv2611c | Rv2611c | phosphatidylinositol mannoside acyltransferase |
| Rv3403c | Rv3403c | hypothetical protein |
| Rv1451 | Rv1451 | protoheme IX farnesyltransferase |
| RVnc0024 | mcr7 | 0 |
| Rv3543c | fadE29 | acyl-CoA dehydrogenase FadE29 |
| Rv2905 | lppW | lipoprotein LppW |
| Rv0337c | aspC | aspartate aminotransferase |
| Rv0911 | Rv0911 | hypothetical protein |
| Rv0638 | secE1 | preprotein translocase SecE |
| Rv3602c | panC | pantothenate synthetase |
| Rv2410c | Rv2410c | hypothetical protein |
| Rv3502c | hsd4A | 3-oxoacyl-ACP reductase |
| Rv2394 | ggtB | gamma-glutamyltranspeptidase precursor GgtB |
| Rv0085 | hycP | hydrogenase HycP |
| Rv2067c | Rv2067c | hypothetical protein |
| Rv2280 | Rv2280 | #N/A |
| Rv0822c | Rv0822c | hypothetical protein |
| Rv0018c | pstP | phosphoserine/threonine phosphatase PstP |
| Rv2849c | cobO | cob(I)alamin adenosyltransferase |
| Rv1911c | lppC | lipoprotein LppC |
| Rv2212 | Rv2212 | adenylyl cyclase |
| Rv1200 | Rv1200 | integral membrane transport protein |
| Rv3351c | Rv3351c | hypothetical protein |
| Rv3449 | mycP4 | membrane-anchored mycosin |
| Rv0885 | Rv0885 | hypothetical protein |
| Rv0518 | Rv0518 | hypothetical protein |
| Rv1169c | lipX | lipase LipX |
| Rv3604c | Rv3604c | transmembrane protein |
| Rv3405c | Rv3405c | HTH-type transcriptional regulator |
| Rv2259 | mscR | S-nitrosomycothiol reductase MscR |
| Rv1735a | Rv1735a | hypothetical protein |
| Rv1442 | bisC | biotin sulfoxide reductase BisC |
| Rvnt15 | alaV | 0 |
| Rv3850 | Rv3850 | hypothetical protein |
| Rv2719c | Rv2719c | membrane protein |
| Rv2138 | lppL | lipoprotein LppL |
| Rv1886c | fbpB | diacylglycerol acyltransferase/mycolyltransferase Ag85B |
| Rv1935c | echA13 | enoyl-CoA hydratase EchA13 |
| Rv2076c | Rv2076c | hypothetical protein |
| Rv0608 | vapB28 | antitoxin VapB28 |
| Rv1357c | Rv1357c | hypothetical protein |
| Rv2149c | yfiH | laccase domain-containing protein |
| Rv2497c | bkdA | 3-methyl-2-oxobutanoate dehydrogenase subunit alpha |
| Rv2975a | Rv2975a | 50S ribosomal protein L28 |
| Rv0015c | pknA | serinethreonine-protein kinase PknA |
| Rv1595 | nadB | L-aspartate oxidase |
| Rv0810c | Rv0810c | hypothetical protein |
| Rv3780 | Rv3780 | hypothetical protein |
| Rv2580c | hisS | histidine--tRNA ligase |
| Rv0504c | Rv0504c | hypothetical protein |
| Rv1982A | vapB36 | antitoxin VapB36 |
| Rv3642c | Rv3642c | hypothetical protein |
| Rv2404c | lepA | GTP-binding protein LepA |
| Rv0510 | hemC | porphobilinogen deaminase |

Supplementary Table 2. List of differentially expressed genes for sputum samples of TB patients undergoing 1-2 weeks of standard anti-tuberculosis therapy relative to the broth cultures.

| Locus tag | Gene symbol | Gene description |
| --- | --- | --- |
| Rv0001 | dnaA | chromosomal replication initiator protein DnaA |
| Rv0002 | dnaN | DNA polymerase III subunit beta |
| Rv0009 | ppiA | iron-regulated peptidyl-prolyl cis-trans isomerase PpiA |
| Rv0095c | Rv0095c | hypothetical protein |
| Rv0976c | Rv0976c | hypothetical protein |
| Rv0979c | Rv0979c | hypothetical protein |
| Rv0980c | PE_PGRS18 | PE-PGRS family protein PE_PGRS18 |
| Rv0981 | mprA | two-component response regulator MrpA |
| Rv0982 | mprB | two component histidine-protein kinase/phosphatase MprB |
| Rv0984 | moaB2 | pterin-4-alpha-carbinolamine dehydratase |
| Rv0985c | mscL | large-conductance ion mechanosensitive channel |
| Rv0987 | Rv0987 | adhesion component ABC transporter permease |
| Rv0988 | Rv0988 | hypothetical protein |
| Rv0989c | grcC2 | polyprenyl-diphosphate synthase GrcC |
| Rv0991c | Rv0991c | hypothetical protein |
| Rv0997 | Rv0997 | hypothetical protein |
| Rv1000c | Rv1000c | hypothetical protein |
| Rv1004c | Rv1004c | membrane protein |
| Rv1006 | Rv1006 | hypothetical protein |
| Rv1010 | ksgA | rRNA small subunit methyltransferase A |
| Rv1011 | ispE | 4-diphosphocytidyl-2C-methyl-D-erythritol kinase |
| Rv1013 | pks16 | polyketide synthase |
| Rv1015c | rplY | 50S ribosomal protein L25general stress protein Ctc |
| Rv1016c | lpqT | lipoprotein LpqT |
| Rv0100 | Rv0100 | hypothetical protein |
| Rv1024 | Rv1024 | membrane protein |
| Rv1027c | kdpE | transcriptional regulator KdpE |
| Rv1028c | kdpD | sensor protein KdpD |
| Rv0101 | nrp | peptide synthetase Nrp |
| Rv1033c | trcR | two component transcriptional regulator TrcR |
| Rv1037c | esxI | ESAT-6 like protein EsxI |
| Rv1038c | esxJ | ESAT-6 like protein EsxJ |
| Rv1040c | PE8 | PE family protein PE8 |
| Rv1041c | Rv1041c | IS2-like transposase |
| Rv1042c | Rv1042c | IS2-like transposase |
| Rv1043c | Rv1043c | hypothetical protein |
| Rv1045 | Rv1045 | hypothetical protein |
| Rv1047 | Rv1047 | transposase |
| Rv1050 | Rv1050 | oxidoreductase |
| Rv1051c | Rv1051c | hypothetical protein |
| Rv0103c | ctpB | cation-transporter P-type ATPase B |
| Rv1064c | lpqV | lipoprotein LpqV |
| Rv1066 | Rv1066 | hypothetical protein |
| Rv1067c | PE_PGRS19 | PE-PGRS family protein PE_PGRS19 |
| Rv1068c | PE_PGRS20 | PE-PGRS family protein PE_PGRS20 |
| Rv1069c | Rv1069c | hypothetical protein |
| Rv1070c | echA8 | enoyl-CoA hydratase EchA8 |
| Rv1072 | Rv1072 | transmembrane protein |
| Rv1073 | Rv1073 | hypothetical protein |
| Rv1075c | Rv1075c | hypothetical protein |
| Rv0010c | Rv0010c | membrane protein |
| Rv0105c | rpmB1 | 50S ribosomal protein L28 |
| Rv1078 | Rv1078 | hypothetical protein |
| Rv1079 | metB | cystathionine gamma-synthase |
| Rv1080c | greA | transcription elongation factor GreA |
| Rv1081c | Rv1081c | membrane protein |
| Rv1082 | mca | mycothiol S-conjugate amidase |
| Rv1083 | Rv1083 | hypothetical protein |
| Rv1088 | PE9 | PE family protein PE9 |
| Rv1091 | PE_PGRS22 | PE-PGRS family protein PE_PGRS22 |
| RVnc0034 | MTS0858 | 0 |
| Rv1094 | desA2 | acyl-ACP desaturase DesA |
| Rv1095 | phoH2 | phosphate starvation-inducible protein PsiH |
| Rv1096 | Rv1096 | glycosyl hydrolase |
| Rv1097c | Rv1097c | hypothetical protein |
| Rv1099c | glpX | fructose 1%2C6-bisphosphatase |
| Rv1101c | Rv1101c | hypothetical protein |
| Rv1102c | mazF3 | mRNA interferase MazF3 |
| Rv1103c | mazE3 | antitoxin MazE3 |
| Rv0108c | Rv0108c | hypothetical protein |
| Rv1109c | Rv1109c | hypothetical protein |
| Rv1111c | Rv1111c | hypothetical protein |
| Rv1112 | Rv1112 | GTP-binding protein |
| Rv1115 | Rv1115 | hypothetical protein |
| Rv1117 | Rv1117 | hypothetical protein |
| Rv0109 | PE_PGRS1 | PE-PGRS family protein PE_PGRS1 |
| Rv1121 | zwf1 | glucose-6-phosphate 1-dehydrogenase |
| Rv1126c | Rv1126c | hypothetical protein |
| Rv1132 | Rv1132 | hypothetical protein |
| Rv1133c | metE | 5-methyltetrahydropteroyltriglutamate--homocysteine methyltransferase |
| Rv1134 | Rv1134 | hypothetical protein |
| Rv1138a | Rv1138a | hypothetical protein |
| Rv1140 | Rv1140 | integral membrane protein |
| Rv1141c | echA11 | enoyl-CoA hydratase EchA11 |
| Rv1142c | echA10 | enoyl-CoA hydratase EchA10 |
| Rv1149 | Rv1149 | transposase |
| Rv1150 | Rv1150 | Rv1150 |
| Rv1152 | Rv1152 | transcriptional regulator |
| Rv1155a | Rv1155a | hypothetical protein |
| Rv1158c | Rv1158c | hypothetical protein |
| Rv1159A | Rv1159A | 4a-hydroxytetrahydrobiopterin dehydratase |
| Rv1160 | mutT2 | 8-oxo-dGTP diphosphatase |
| Rv1162 | narH | nitrate reductase subunit beta |
| Rv1169c | lipX | lipase LipX |
| Rv1172c | PE12 | PE family protein PE12 |
| Rv1173 | fbiC | FO synthase |
| Rv1174c | TB8.4 | low molecular weight T-cell antigen |
| Rv0011c | Rv0011c | cell division protein CrgA |
| Rv1177 | fdxC | ferredoxin FdxC |
| Rv1178 | Rv1178 | aminotransferase |
| Rv1181 | pks4 | polyketide beta-ketoacyl synthase |
| Rv1182 | papA3 | acyltransferase papA3 |
| Rv1183 | mmpL10 | transmembrane transport protein MmpL10 |
| Rv1184c | Rv1184c | hypothetical protein |
| Rv1185c | fadD21 | fatty-acid--CoA ligase FadD21 |
| Rv1186c | Rv1186c | hypothetical protein |
| Rv1187 | rocA | pyrroline-5-carboxylate dehydrogenase RocA |
| Rv1188 | Rv1188 | proline dehydrogenase |
| Rv1192 | Rv1192 | hypothetical protein |
| Rv1194c | Rv1194c | hypothetical protein |
| Rv1195 | PE13 | PE family protein PE13 |
| Rv0116c | ldtA | L%2CD-transpeptidase LdtA |
| Rv1197 | esxK | ESAT-6 like protein EsxK |
| Rv1198 | esxL | ESAT-6 like protein EsxL |
| Rv1199c | Rv1199c | insertion sequence element IS1081 transposase |
| Rv1200 | Rv1200 | integral membrane transport protein |
| Rv1201c | dapD | 2%2C3%2C4%2C5-tetrahydropyridine-2%2C6-dicarboxylate N-succinyltransferase |
| Rv1204c | Rv1204c | hypothetical protein |
| Rv0117 | oxyS | oxidative stress response regulatory protein OxyS |
| Rv1211 | Rv1211 | hypothetical protein |
| Rv1212c | glgA | capsular glucan synthase |
| Rv1214c | PE14 | PE family protein PE14 |
| Rv1215c | Rv1215c | hypothetical protein |
| Rv1216c | Rv1216c | integral membrane protein |
| Rv1217c | Rv1217c | tetronasin ABC transporter integral membrane protein |
| Rv1219c | Rv1219c | transcriptional regulator |
| Rv1221 | sigE | ECF RNA polymerase sigma factor SigE |
| Rv1222 | rseA | anti-sigma E factor RseA |
| Rv1223 | htrA | serine protease HtrA |
| Rv1224 | tatB | Sec-independent protein translocase protein TatB |
| Rv1225c | Rv1225c | hypothetical protein |
| Rv1227c | Rv1227c | transmembrane protein |
| Rv1228a | Rv1228a | hypothetical protein |
| Rv1238 | sugC | sugar ABC transporter ATP-binding protein SugC |
| Rv1243c | PE_PGRS23 | PE-PGRS family protein PE_PGRS23 |
| Rv1248c | Rv1248c | multifunctional 2-oxoglutarate dehydrogenase E1 component /2-oxoglutarate dehydrogenase dihydrolipoyllysine-residue succinyltransferase |
| Rv1259 | udgB | uracil DNA glycosylase |
| Rv1260 | Rv1260 | oxidoreductase |
| Rv1261c | Rv1261c | hypothetical protein |
| Rv1262c | Rv1262c | hypothetical protein |
| Rv1263 | amiB2 | amidase AmiB |
| Rv1264 | Rv1264 | adenylyl cyclase |
| Rv1271c | Rv1271c | hypothetical protein |
| Rv1274 | lprB | lipoprotein LprB |
| Rv0124 | PE_PGRS2 | PE-PGRS family protein PE_PGRS2 |
| Rv1282c | oppC | oligopeptide ABC transporter permease OppC |
| Rv1283c | oppB | oligopeptide ABC transporter permease OppB |
| Rv1288 | Rv1288 | hypothetical protein |
| Rv1290A | Rv1290A | hypothetical protein |
| Rv1291c | Rv1291c | hypothetical protein |
| Rv1293 | lysA | diaminopimelate decarboxylase |
| Rv1294 | thrA | homoserine dehydrogenase |
| Rv1296 | thrB | homoserine kinase |
| Rv1297 | rho | transcription termination factor Rho |
| Rv1298 | rpmE | 50S ribosomal protein L31 |
| Rv1299 | prfA | peptide chain release factor PrfA |
| Rv1300 | hemK | release factor glutamine methyltransferase |
| Rv1301 | Rv1301 | threonylcarbamoyl-AMP synthase |
| Rv1303 | Rv1303 | hypothetical protein |
| Rv1304 | atpB | ATP synthase subunit A |
| Rv1305 | atpE | ATP synthase subunit C |
| Rv1306 | atpF | ATP synthase subunit B |
| Rv1307 | atpH | ATP synthase subunit b/delta |
| Rv1308 | atpA | ATP synthase subunit alpha |
| Rv1309 | atpG | ATP synthase subunit gamma |
| Rv1310 | atpD | ATP synthase subunit beta |
| Rv1311 | atpC | ATP synthase subunit epsilon |
| Rv1312 | Rv1312 | hypothetical protein |
| Rv0128 | Rv0128 | transmembrane protein |
| Rv1313c | Rv1313c | insertion sequence element IS1557 transposase |
| Rvnr01 | rrs | 0 |
| Rvnr02 | rrl | 0 |
| Rv1316c | ogt | methylated-DNA--protein-cysteine methyltransferase |
| Rv1318c | Rv1318c | adenylate cyclase |
| Rv0129c | fbpC | diacylglycerol acyltransferase/mycolyltransferase Ag85C |
| Rv1320c | Rv1320c | adenylate cyclase |
| Rv1321 | Rv1321 | endonuclease NucS |
| Rv1322 | Rv1322 | hypothetical protein |
| Rv1322A | Rv1322A | hypothetical protein |
| Rv1323 | fadA4 | acetyl-CoA acetyltransferase |
| Rv1324 | Rv1324 | thioredoxin |
| Rv1326c | glgB | 1%2C4-alpha-glucan branching protein |
| Rv1327c | glgE | alpha-1%2C4-glucan:maltose-1-phosphate maltosyltransferase |
| Rv1330c | pncB1 | nicotinic acid phosphoribosyltransferase PncB1 |
| Rv1331 | Rv1331 | ATP-dependent Clp protease adapter protein ClpS |
| Rv1332 | Rv1332 | transcriptional regulator |
| Rv1334 | mec | [CysO]-cysteine peptidase |
| Rv1339 | Rv1339 | hypothetical protein |
| Rv1342c | Rv1342c | hypothetical protein |
| Rv1343c | lprD | lipoprotein LprD |
| Rv1347c | mbtK | lysine N-acetyltransferase MbtK |
| Rv1352 | Rv1352 | hypothetical protein |
| Rv1354c | Rv1354c | hypothetical protein |
| Rv1356c | Rv1356c | hypothetical protein |
| Rv1357c | Rv1357c | hypothetical protein |
| Rv1360 | Rv1360 | oxidoreductase |
| Rv1362c | Rv1362c | membrane protein |
| Rv0013 | trpG | anthranilate synthase component II |
| Rv1366A | Rv1366A | hypothetical protein |
| Rv1368 | lprF | lipoprotein LprF |
| Rv1374c | Rv1374c | hypothetical protein |
| RVnc0035 | MTS1082 | 0 |
| Rv1375 | Rv1375 | hypothetical protein |
| Rv0135c | Rv0135c | transcriptional regulator |
| Rv1381 | pyrC | dihydroorotase |
| Rv1382 | Rv1382 | hypothetical protein |
| Rv1383 | carA | carbamoyl-phosphate synthase small subunit |
| Rv1385 | pyrF | orotidine 5'-phosphate decarboxylase |
| Rv1386 | PE15 | PE family protein PE15 |
| Rv1389 | gmk | guanylate kinase |
| Rv1390 | rpoZ | DNA-directed RNA polymerase subunit omega |
| Rv1393c | Rv1393c | monoxygenase |
| Rv1395 | Rv1395 | HTH-type transcriptional regulator |
| Rv0137c | msrA | peptide methionine sulfoxide reductase MsrA |
| Rv1397c | vapC10 | ribonuclease VapC10 |
| Rv1398c | vapB10 | antitoxin VapB10 |
| Rv1403c | Rv1403c | methyltransferase |
| Rv1404 | Rv1404 | transcriptional regulator |
| Rv0138 | Rv0138 | hypothetical protein |
| Rv1407 | fmu | 16S rRNA m5C967 methyltransferase |
| Rv1409 | ribG | bifunctional riboflavin biosynthesis diaminohydroxyphosphoribosylaminopyrimidine deaminase5-amino-6-(5-phosphoribosylamino) uracil reductase |
| Rv1414 | Rv1414 | hypothetical protein |
| Rv1415 | ribA2 | bifunctional riboflavin biosynthesis GTP cyclohydrolase II3%2C4-dihydroxy-2-butanone 4-phosphate synthase |
| Rv1416 | ribH | 6%2C7-dimethyl-8-ribityllumazine synthase |
| Rv1419 | Rv1419 | hypothetical protein |
| Rv1425 | Rv1425 | diacyglycerol O-acyltransferase |
| Rv0140 | Rv0140 | hypothetical protein |
| Rv1430 | PE16 | PE family protein PE16 |
| Rv1433 | Rv1433 | hypothetical protein |
| Rv1434 | Rv1434 | hypothetical protein |
| Rv1435c | Rv1435c | hypothetical protein |
| Rv1436 | gap | glyceraldehyde 3-phosphate dehydrogenase |
| Rv1437 | pgk | phosphoglycerate kinase |
| Rv1438 | tpi | triosephosphate isomerase |
| Rv1440 | secG | protein-export membrane protein SecG |
| Rv1441c | PE_PGRS26 | PE-PGRS family protein PE_PGRS26 |
| Rv1445c | devB | 6-phosphogluconolactonase |
| Rv0142 | Rv0142 | hypothetical protein |
| Rv1447c | zwf2 | glucose-6-phosphate 1-dehydrogenase |
| Rv1450c | PE_PGRS27 | PE-PGRS family protein PE_PGRS27 |
| Rv1451 | Rv1451 | protoheme IX farnesyltransferase |
| Rv1453 | Rv1453 | transcriptional activator protein |
| Rv1458c | Rv1458c | antibiotic ABC transporter ATP-binding protein |
| Rv1461 | Rv1461 | hypothetical protein |
| Rv0014c | pknB | serinethreonine-protein kinase PknB |
| Rv1466 | Rv1466 | hypothetical protein |
| Rv1470 | trxA | thioredoxin TrxA |
| Rv0145 | Rv0145 | S-adenosylmethionine-dependent methyltransferase |
| Rv1475c | acn | iron-regulated aconitate hydratase |
| Rv1479 | moxR1 | transcriptional regulator MoxR1 |
| Rv1481 | Rv1481 | membrane protein |
| Rv1483 | fabG1 | 3-oxoacyl-ACP reductase FabG |
| Rv1485 | hemZ | ferrochelatase |
| Rv1487 | Rv1487 | hypothetical protein |
| Rv1488 | Rv1488 | hypothetical protein |
| Rv1490 | Rv1490 | membrane protein |
| Rv1494 | mazE4 | antitoxin MazE4 |
| Rv1498c | Rv1498c | methyltransferase |
| Rv1500 | Rv1500 | glycosyltransferase |
| Rv1501 | Rv1501 | hypothetical protein |
| Rv1502 | Rv1502 | hypothetical protein |
| Rv0148 | Rv0148 | short-chain type dehydrogenase/reductase |
| Rv1503c | Rv1503c | Rv1503c |
| Rv1505c | Rv1505c | hypothetical protein |
| Rv1507c | Rv1507c | hypothetical protein |
| Rv1507A | Rv1507A | hypothetical protein |
| Rv1508c | Rv1508c | membrane protein |
| Rv1509 | Rv1509 | hypothetical protein |
| Rv1513 | Rv1513 | hypothetical protein |
| Rv1521 | fadD25 | fatty-acid--CoA ligase FadD25 |
| Rv1526c | Rv1526c | glycosyltransferase |
| Rv1528c | papA4 | polyketide synthase associated protein PapA |
| Rv1531 | Rv1531 | hypothetical protein |
| Rv1538c | ansA | L-aparaginase |
| Rv1539 | lspA | lipoprotein signal peptidase |
| Rv1541c | lprI | lipoprotein LprI |
| Rv1542c | glbN | hemoglobin GlbN |
| Rv1547 | dnaE1 | DNA polymerase III subunit alpha |
| Rv1548c | PPE21 | PPE family protein PPE21 |
| Rv0153c | ptbB | phosphotyrosine protein phosphatase |
| Rv1554 | frdC | fumarate reductase membrane anchor subunit |
| Rv1555 | frdD | fumarate reductase membrane anchor subunit |
| Rv1560 | vapB11 | antitoxin VapB11 |
| Rv0015c | pknA | serinethreonine-protein kinase PknA |
| Rv1565c | Rv1565c | acyltransferase |
| Rv1566c | Rv1566c | hypothetical protein |
| Rv1567c | Rv1567c | membrane protein |
| Rv1568 | bioA | adenosylmethionine--8-amino-7-oxononanoate aminotransferase BioA |
| Rv1569 | bioF1 | 8-amino-7-oxononanoate synthase |
| Rv1570 | bioD | ATP-dependent dethiobiotin synthetase BioD |
| Rv1571 | Rv1571 | hypothetical protein |
| Rv0155 | pntAa | NAD(P) transhydrogenase subunit alpha PntAa |
| Rv1577c | Rv1577c | phage prohead protease |
| Rv1578c | Rv1578c | phage protein |
| Rv1579c | Rv1579c | phage protein |
| Rv1583c | Rv1583c | phage protein |
| Rv0156 | pntAb | NAD(P) transhydrogenase subunit alpha PntAb |
| Rv1584c | Rv1584c | phage protein |
| Rv1586c | Rv1586c | phage integrase |
| Rv1589 | bioB | biotin synthetase |
| Rv1592c | Rv1592c | hypothetical protein |
| Rv1593c | Rv1593c | hypothetical protein |
| Rv0157 | pntB | NAD(P) transhydrogenase subunit beta PntB |
| Rv1594 | nadA | quinolinate synthetase A |
| Rv1595 | nadB | L-aspartate oxidase |
| Rv1596 | nadC | nicotinate-nucleotide pyrophosphatase |
| Rv1597 | Rv1597 | hypothetical protein |
| Rv1600 | hisC1 | histidinol-phosphate aminotransferase |
| Rv0157A | Rv0157A | hypothetical protein |
| Rv1607 | chaA | ionic transporter integral membrane protein ChaA |
| Rv1608c | bcpB | peroxiredoxin |
| Rv1611 | trpC | indole-3-glycerol phosphate synthase |
| Rv1612 | trpB | tryptophan synthase subunit beta |
| Rv1613 | trpA | tryptophan synthase subunit alpha |
| Rv1614 | lgt | prolipoprotein diacylglyceryl transferase |
| Rv1618 | tesB1 | acyl-CoA thioesterase II |
| Rv1620c | cydC | cytochrome biosyntheisis ABC transporter ATP-binding proteinpermease CydC |
| Rv1623c | cydA | cytochrome D ubiquinol oxidase subunit I CydA |
| Rv0159c | PE3 | PE family protein PE3 |
| Rv1626 | Rv1626 | two-component system transcriptional regulator |
| Rv1628c | Rv1628c | hypothetical protein |
| Rv1630 | rpsA | 30S ribosomal protein S1 |
| Rv1631 | coaE | dephospho-CoA kinase CoaE |
| Rv1632c | Rv1632c | hypothetical protein |
| Rv1636 | TB15.3 | iron-regulated universal stress protein |
| Rv1641 | infC | initiation factor IF-3 |
| Rv0161 | Rv0161 | oxidoreductase |
| Rv1643 | rplT | 50S ribosomal protein L20 |
| Rv1645c | Rv1645c | hypothetical protein |
| Rv1646 | PE17 | PE family protein PE17 |
| Rv1652 | argC | N-acetyl-gamma-glutamyl-phoshate reductase |
| Rv1653 | argJ | bifunctional glutamate N-acetyltransferase/amino-acid acetyltransferase |
| Rv1654 | argB | acetylglutamate kinase |
| Rv1655 | argD | acetylornithine aminotransferase |
| Rv1656 | argF | ornithine carbamoyltransferase |
| Rv1657 | argR | arginine repressor |
| Rv0163 | Rv0163 | hypothetical protein |
| Rv0164 | TB18.5 | hypothetical protein |
| Rv1673c | Rv1673c | hypothetical protein |
| Rv1674c | Rv1674c | transcriptional regulator |
| Rv1677 | dsbF | lipoprotein DsbF |
| Rv0165c | mce1R | transcriptional regulator Mce1R |
| Rv1688 | mpg | 3-methyladenine DNA glycosylase |
| RVnc0010 | G2 | 0 |
| Rv1690 | lprJ | lipoprotein LprJ |
| Rv1691 | Rv1691 | hypothetical protein |
| Rv0166 | fadD5 | fatty-acid--CoA ligase FadD5 |
| Rv1697 | Rv1697 | hypothetical protein |
| Rv1698 | mctB | copper transporter MctB |
| Rv1699 | pyrG | CTP synthase |
| Rv0167 | yrbE1A | membrane protein |
| Rv1703c | Rv1703c | methyltransferase |
| Rv1704c | cycA | D-serinealanineglycine transporter protein CycA |
| Rv1706A | Rv1706A | hypothetical protein |
| Rv1710 | scpB | segregation and condensation protein ScpB |
| Rv1715 | fadB3 | 3-hydroxybutyryl-CoA dehydrogenase FadB |
| Rv1717 | Rv1717 | hypothetical protein |
| Rvnt21 | proT | 0 |
| Rv0169 | mce1A | Mce family protein Mce1A |
| Rv1726 | Rv1726 | oxidoreductase |
| Rv1727 | Rv1727 | hypothetical protein |
| Rv0170 | mce1B | Mce family protein Mce1B |
| Rv1734c | Rv1734c | hypothetical protein |
| Rv1735c | Rv1735c | membrane protein |
| Rv1735a | Rv1735a | hypothetical protein |
| Rv0171 | mce1C | Mce family protein Mce1C |
| Rv1738 | Rv1738 | hypothetical protein |
| Rv1740 | vapB34 | antitoxin VapB34 |
| Rv1743 | pknE | serine/threonine-protein kinase PknE |
| Rv1745c | idi | isopentenyl-diphosphate delta-isomerase |
| Rv1746 | pknF | serinethreonine-protein kinase PknF |
| Rv0172 | mce1D | Mce family protein Mce1D |
| Rv1749c | Rv1749c | integral membrane protein |
| Rv1753c | PPE24 | PPE family protein PPE24 |
| Rv1759c | wag22 | PE-PGRS family protein Wag22 |
| Rv0017c | rodA | cell division protein RodA |
| Rv0173 | lprK | Mce family lipoprotein LprK |
| Rv1760 | Rv1760 | diacylglycerol acyltransferase |
| Rv1761c | Rv1761c | hypothetical protein |
| Rv1765c | Rv1765c | hypothetical protein |
| Rv1766 | Rv1766 | hypothetical protein |
| Rv1767 | Rv1767 | hypothetical protein |
| Rv1768 | PE_PGRS31 | PE-PGRS family protein PE_PGRS31 |
| Rv1771 | Rv1771 | L-gulono-1%2C4-lactone dehydrogenase |
| Rv0174 | mce1F | Mce family protein Mce1F |
| Rv1773c | Rv1773c | transcriptional regulator |
| Rv1774 | Rv1774 | oxidoreductase |
| Rv1776c | Rv1776c | transcriptional regulator |
| Rv1780 | Rv1780 | hypothetical protein |
| Rv0175 | Rv0175 | Mce associated membrane protein |
| Rv1782 | eccB5 | ESX-5 type VII secretion system protein EccB5 |
| Rv1783 | eccC5 | ESX-5 type VII secretion system protein EccC5 |
| Rv1787 | PPE25 | PPE family protein PPE25 |
| Rv1792 | esxM | - |
| Rv0176 | Rv0176 | Mce associated transmembrane protein |
| Rv1793 | esxN | ESAT-6 like protein EsxN |
| Rv1794 | Rv1794 | hypothetical protein |
| Rv1795 | eccD5 | ESX-5 type VII secretion system protein EccD |
| Rv1796 | mycP5 | membrane-anchored mycosin MycP |
| Rv1797 | eccE5 | ESX-5 type VII secretion system protein EccE |
| Rv1798 | eccA5 | ESX-5 type VII secretion system protein EccA |
| Rv1799 | lppT | lipoprotein LppT |
| Rv1800 | PPE28 | PPE family protein PPE28 |
| Rv0177 | Rv0177 | Mce associated protein |
| Rv1809 | PPE33 | PPE family protein PPE33 |
| Rv1810 | Rv1810 | hypothetical protein |
| Rv1812c | Rv1812c | dehydrogenase |
| Rv0178 | Rv0178 | Mce associated membrane protein |
| Rv1815 | Rv1815 | hypothetical protein |
| Rv1816 | Rv1816 | HTH-type transcriptional regulator |
| Rv1821 | secA2 | accessory Sec system translocase SecA2 |
| Rv1822 | pgsA2 | CDP-diacylglycerol--glycerol-3-phosphate 3-phosphatidyltransferase |
| Rv0179c | lprO | lipoprotein LprO |
| Rv1825 | Rv1825 | hypothetical protein |
| Rv1826 | gcvH | glycine cleavage system protein H |
| Rv1827 | garA | glycogen accumulation regulator GarA |
| Rv1829 | Rv1829 | hypothetical protein |
| Rv1830 | Rv1830 | HTH-type transcriptional regulator |
| Rv1831 | Rv1831 | hypothetical protein |
| Rv1832 | gcvB | glycine dehydrogenase |
| Rv1833c | Rv1833c | haloalkane dehalogenase |
| Rv1834 | lipZ | hydrolase |
| Rv1837c | glcB | malate synthase |
| Rv1840c | PE_PGRS34 | PE-PGRS family protein PE_PGRS34 |
| Rv1843c | guaB1 | inosine-5'-monophosphate dehydrogenase |
| Rv1846c | blaI | transcriptional repressor BlaI |
| Rv1852 | ureG | urease accessory protein UreG |
| Rv0182c | sigG | ECF RNA polymerase sigma factor SigG |
| Rv1856c | Rv1856c | oxidoreductase |
| Rv1861 | Rv1861 | transmembrane protein |
| Rv0018c | pstP | phosphoserine/threonine phosphatase PstP |
| Rv1868 | Rv1868 | hypothetical protein |
| Rv1869c | Rv1869c | reductase |
| Rv1870c | Rv1870c | hypothetical protein |
| Rv1871c | Rv1871c | hypothetical protein |
| Rv1872c | lldD2 | L-lactate dehydrogenase |
| Rv1876 | bfrA | bacterioferritin BfrA |
| Rv1877 | Rv1877 | MFS-type transporter |
| Rv1880c | cyp140 | cytochrome P450 Cyp140 |
| Rv1881c | lppE | lipoprotein LppE |
| Rv1882c | Rv1882c | short-chain type dehydrogenasereductase |
| Rv1883c | Rv1883c | hypothetical protein |
| Rv1885c | *MtCM | chorismate mutase |
| Rv1886c | fbpB | diacylglycerol acyltransferase/mycolyltransferase Ag85B |
| Rv1887 | Rv1887 | hypothetical protein |
| Rv1891 | Rv1891 | hypothetical protein |
| Rv1893 | Rv1893 | hypothetical protein |
| Rv1894c | Rv1894c | hypothetical protein |
| Rv1898 | Rv1898 | hypothetical protein |
| Rv1901 | cinA | competence damage-inducible protein CinA |
| Rv1902c | nanT | sialic acid-transport integral membrane protein NanT |
| Rv0186A | mymT | metallothionein |
| Rv1903 | Rv1903 | membrane protein |
| Rv1904 | Rv1904 | hypothetical protein |
| Rv1909c | furA | ferric uptake regulation protein FurA |
| Rv1911c | lppC | lipoprotein LppC |
| Rv1912c | fadB5 | oxidoreductase FadB |
| Rv1915 | aceAa | isocitrate lyase AceAa |
| Rv1918c | PPE35 | PPE family protein PPE35 |
| Rv1919c | Rv1919c | hypothetical protein |
| Rv0188 | Rv0188 | transmembrane protein |
| Rv1923 | lipD | lipase LipD |
| Rv1925 | fadD31 | fatty-acid--CoA ligase FadD31 |
| Rv1926c | mpt63 | immunogenic protein Mpt63 |
| Rv1928c | Rv1928c | short-chain type dehydrogenasereductase |
| Rv1930c | Rv1930c | hypothetical protein |
| Rv1931c | Rv1931c | transcriptional regulator |
| Rv1932 | tpx | 2-Cys peroxiredoxin |
| Rv1936 | Rv1936 | monooxygenase |
| Rv1939 | Rv1939 | oxidoreductase |
| Rv1942c | mazF5 | toxin MazF5 |
| Rv0190 | Rv0190 | hypothetical protein |
| Rv1948c | Rv1948c | hypothetical protein |
| Rv1951c | Rv1951c | hypothetical protein |
| Rv1954c | Rv1954c | hypothetical protein |
| Rv1955 | higB | toxin HigB |
| Rv1957 | Rv1957 | SecB-like chaperone |
| Rv1959c | parE1 | toxin ParE1 |
| Rv1960c | parD1 | antitoxin ParD1 |
| Rv1961 | Rv1961 | hypothetical protein |
| Rv1962c | vapC35 | ribonuclease VapC35 |
| Rv1962A | vapB35 | antitoxin VapB35 |
| Rv0019c | fhaB | FHA domain-containing protein FhaB |
| Rv1964 | yrbE3A | integral membrane protein |
| Rv1965 | yrbE3B | integral membrane protein |
| Rv1967 | mce3B | Mce family protein Mce3B |
| Rv1968 | mce3C | Mce family protein Mce3C |
| Rv1969 | mce3D | Mce family protein Mce3D |
| Rv1971 | mce3F | Mce family protein Mce3F |
| Rv0193c | Rv0193c | hypothetical protein |
| Rv1973 | Rv1973 | Mce associated membrane protein |
| Rv1978 | Rv1978 | hypothetical protein |
| Rv1979c | Rv1979c | permease |
| Rv1980c | mpt64 | immunogenic protein Mpt64 |
| Rv1981c | nrdF1 | ribonucleoside-diphosphate reductase subunit beta NrdF1 |
| Rv1982A | vapB36 | antitoxin VapB36 |
| Rv1983 | PE_PGRS35 | PE-PGRS family protein PE_PGRS35 |
| Rv1984c | cfp21 | cutinase |
| Rv1989c | Rv1989c | hypothetical protein |
| Rv1993c | Rv1993c | hypothetical protein |
| Rv1996 | Rv1996 | universal stress protein |
| Rv1999c | Rv1999c | transporter |
| Rv2001 | Rv2001 | hypothetical protein |
| Rv2005c | Rv2005c | universal stress protein |
| Rv2007c | fdxA | ferredoxin |
| Rv2010 | vapC15 | ribonuclease VapC15 |
| Rv2011c | Rv2011c | hypothetical protein |
| Rv2015c | Rv2015c | hypothetical protein |
| Rv2022c | Rv2022c | hypothetical protein |
| Rv2023c | Rv2023c | hypothetical protein |
| Rv2031c | hspX | alpha-crystallin |
| Rv2033c | Rv2033c | hypothetical protein |
| Rv2035 | Rv2035 | hypothetical protein |
| Rv2040c | Rv2040c | sugar ABC transporter permease |
| Rv2044c | Rv2044c | hypothetical protein |
| Rv2053c | fxsA | transmembrane protein FxsA |
| Rv0020c | fhaA | FHA domain-containing protein FhaA |
| Rv2063 | mazE7 | antitoxin MazE7 |
| Rv2063A | mazF7 | mRNA interferase MazF7 |
| Rv2064 | cobG | precorrin-3B synthase |
| Rv2065 | cobH | precorrin-8X methylmutase |
| Rv2067c | Rv2067c | hypothetical protein |
| Rv0203 | Rv0203 | hypothetical protein |
| Rv2073c | Rv2073c | oxidoreductase |
| Rv2076c | Rv2076c | hypothetical protein |
| Rv0204c | Rv0204c | transmembrane protein |
| Rv2079 | Rv2079 | hypothetical protein |
| Rv2080 | lppJ | lipoprotein LppJ |
| Rv2081c | Rv2081c | transmembrane protein |
| Rv2082 | Rv2082 | hypothetical protein |
| Rv2085 | Rv2085 | hypothetical protein |
| Rv0205 | Rv0205 | transmembrane protein |
| Rv2091c | Rv2091c | membrane protein |
| Rv2093c | tatC | Sec-independent protein translocase transmembrane protein TatC |
| Rv2094c | tatA | Sec-independent protein translocase membrane-bound protein TatA |
| Rv2098c | PE_PGRS36 | Rv2098c |
| Rv0206c | mmpL3 | transmembrane transport protein MmpL3 |
| Rv2101 | helZ | helicase HelZ |
| Rv2102 | Rv2102 | hypothetical protein |
| Rv2104c | vapB37 | antitoxin VapB37 |
| Rv2108 | PPE36 | PPE family protein PPE36 |
| Rv2109c | prcA | proteasome subunit alpha |
| Rv0207c | Rv0207c | hypothetical protein |
| Rv2111c | pup | ubiquitin-like protein Pup |
| Rv2114 | Rv2114 | hypothetical protein |
| Rv2115c | mpa | proteasome-associated ATPase |
| Rv2116 | lppK | lipoprotein LppK |
| Rv2120c | Rv2120c | integral membrane protein |
| Rv0208c | Rv0208c | tRNA (guanine-N(7)-)-methyltransferase |
| Rv2123 | PPE37 | PPE family protein PPE37 |
| Rv2127 | ansP1 | L-asparagine permease |
| Rv2129c | Rv2129c | oxidoreductase |
| Rv0209 | Rv0209 | hypothetical protein |
| Rv2135c | Rv2135c | hypothetical protein |
| Rv2136c | Rv2136c | undecaprenyl-diphosphatase |
| Rv2137c | Rv2137c | hypothetical protein |
| Rv2138 | lppL | lipoprotein LppL |
| Rvnt22 | leuU | 0 |
| Rv2142A | parD2 | antitoxin ParD2 |
| Rv2144c | Rv2144c | transmembrane protein |
| Rv2145c | wag31 | cell wall synthesis protein Wag31 |
| Rv2146c | Rv2146c | transmembrane protein |
| Rv2148c | Rv2148c | hypothetical protein |
| Rv0211 | pckA | phosphoenolpyruvate carboxykinase |
| Rv2150c | ftsZ | cell division protein FtsZ |
| Rv2151c | ftsQ | cell division protein FtsQ |
| Rv2153c | murG | UDP-N-acetylglucosamine--N-acetylmuramyl-(pentapeptide) pyrophosphoryl-undecaprenol-N-acetylglucosamine transferase |
| Rv2154c | ftsW | lipid II flippase FtsW |
| Rvnt03 | leuT | 0 |
| Rv2160c | Rv2160c | Rv2160c |
| Rv2161c | Rv2161c | hypothetical protein |
| Rv2162c | PE_PGRS38 | PE-PGRS family protein PE_PGRS38 |
| Rv2163c | pbpB | penicillin-binding membrane protein PbpB |
| Rv2166c | Rv2166c | transcriptional regulator MraZ |
| Rv2172c | Rv2172c | hypothetical protein |
| Rv2174 | mptA | alpha(1->6)-mannopyranosyltransferase A |
| Rv2177c | Rv2177c | transposase |
| Rv2178c | aroG | phospho-2-dehydro-3-deoxyheptonate aldolase AroG |
| Rv2181 | Rv2181 | alpha-(1-2)-phosphatidylinositol mannoside mannosyltransferase |
| Rv2182c | Rv2182c | 1-acylglycerol-3-phosphate O-acyltransferase |
| Rv2183c | Rv2183c | hypothetical protein |
| Rv2190c | Rv2190c | endopeptidase |
| Rv2193 | ctaE | cytochrome C oxidase subunit III |
| Rv2194 | qcrC | ubiquinol-cytochrome C reductase cytochrome subunit C |
| Rv2195 | qcrA | ubiquinol-cytochrome C reductase rieske iron-sulfur subunit |
| Rv2196 | qcrB | ubiquinol-cytochrome C reductase cytochrome subunit B |
| Rv2197c | Rv2197c | transmembrane protein |
| Rv2199c | Rv2199c | cytochrome c oxidase polypeptide 4 |
| Rv2200c | ctaC | cytochrome C oxidase subunit II |
| Rv2202c | adoK | adenosine kinase |
| Rv2203 | Rv2203 | membrane protein |
| Rv2204c | Rv2204c | hypothetical protein |
| Rv2207 | cobT | nicotinate-nucleotide-dimethylbenzimidazol phosphoribosyltransferase |
| Rv2209 | Rv2209 | integral membrane protein |
| Rv0217c | lipW | esterase LipW |
| Rv2214c | ephD | oxidoreductase EphD |
| Rv2215 | dlaT | pyruvate dehydrogenase E2 component dihydrolipoamide acyltransferase |
| Rv2216 | Rv2216 | epimerase family protein |
| Rv2219 | Rv2219 | transmembrane protein |
| Rv0218 | Rv0218 | transmembrane protein |
| Rv2222c | glnA2 | glutamine synthetase |
| Rv2224d | Rv2224d | hypothetical protein |
| Rv0219 | Rv0219 | transmembrane protein |
| Rv2228c | Rv2228c | multifunctional RNASE H/alpha-ribazole phosphatase/acid phosphatase |
| Rv2229c | Rv2229c | hypothetical protein |
| Rv2231c | cobC | aminotransferase |
| Rv2236c | cobD | cobalamin biosynthesis transmembrane protein CobD |
| Rv0220 | lipC | esterase LipC |
| Rv2237A | Rv2237A | hypothetical protein |
| Rv2238c | ahpE | peroxiredoxin |
| Rv2239c | Rv2239c | hypothetical protein |
| Rv2241 | aceE | pyruvate dehydrogenase E1 component |
| Rv2243 | fabD | malonyl CoA-acyl carrier protein transacylase |
| Rv2244 | acpM | meromycolate extension acyl carrier protein |
| Rv2245 | kasA | 3-oxoacyl-ACP synthase 1 |
| Rv2246 | kasB | 3-oxoacyl-ACP synthase 2 |
| Rv2247 | accD6 | acetyl-propionyl-CoA carboxylase subunit beta |
| Rv2249c | glpD1 | glycerol-3-phosphate dehydrogenase |
| Rv2256c | Rv2256c | hypothetical protein |
| Rv2257c | Rv2257c | hypothetical protein |
| Rv2260 | Rv2260 | hypothetical protein |
| Rv2263 | Rv2263 | oxidoreductase |
| Rv0223c | Rv0223c | aldehyde dehydrogenase |
| Rv2267c | Rv2267c | hypothetical protein |
| Rv2273 | Rv2273 | transmembrane protein |
| Rv2283 | Rv2283 | hypothetical protein |
| Rv2285 | Rv2285 | diacylglycerol acyltransferase |
| Rv0225 | Rv0225 | hypothetical protein |
| Rv2289 | cdh | CDP-diacylglycerol pyrophosphatase |
| Rv2293c | Rv2293c | hypothetical protein |
| Rv2299c | htpG | chaperone protein HtpG |
| Rv2303c | Rv2303c | antibiotic-resistance protein |
| Rv2305 | Rv2305 | hypothetical protein |
| Rv0227c | Rv0227c | membrane protein |
| Rv2307A | Rv2307A | hypothetical protein |
| Rv2307D | Rv2307D | hypothetical protein |
| Rvnt24 | metV | 0 |
| Rv2309A | Rv2309A | hypothetical protein |
| Rv0228 | Rv0228 | acyltransferase |
| Rv2312 | Rv2312 | hypothetical protein |
| Rv2316 | uspA | sugar ABC transporter permease UspA |
| Rv2327 | Rv2327 | hypothetical protein |
| Rv2328 | PE23 | PE family protein PE23 |
| Rv2329c | narK1 | nitratenitrite transporter |
| Rv0230c | php | phosphotriesterase |
| Rv2336 | Rv2336 | hypothetical protein |
| Rv2342 | Rv2342 | hypothetical protein |
| Rv2345 | Rv2345 | transmembrane protein |
| Rv2346c | esxO | ESAT-6 like protein EsxO |
| Rv2347c | esxP | ESAT-6 like protein EsxP |
| Rv2348c | Rv2348c | hypothetical protein |
| Rv2350c | plcB | membrane-associated phospholipase B |
| Rv0022c | whiB5 | transcriptional regulator WhiB5 |
| Rv2353c | PPE39 | PPE family protein PPE39 |
| Rv2357c | glyS | glycine--tRNA ligase |
| Rv2358 | smtB | HTH-type transcriptional regulator SmtB |
| Rv2359 | zur | zinc uptake regulation protein |
| Rv2364c | era | GTPase Era |
| Rv2368c | phoH1 | phosphate starvation-inducible protein PhoH |
| Rv2374c | hrcA | heat-inducible transcription repressor HrcA |
| Rv2375 | Rv2375 | hypothetical protein |
| Rv2383c | mbtB | phenyloxazoline synthase |
| Rv0235c | Rv0235c | transmembrane protein |
| Rv2384 | mbtA | 2%2C3-dihydroxybenzoate-AMP ligase |
| Rv2388c | hemN | oxygen-independent coproporphyrinogen III oxidase |
| Rv2389c | rpfD | resuscitation-promoting factor RpfD |
| Rv2390c | Rv2390c | hypothetical protein |
| Rv2391 | sirA | sulfite reductase |
| Rv2392 | cysH | phosphoadenosine phosphosulfate reductase |
| Rv2393 | che1 | ferrochelatase |
| Rv2395 | Rv2395 | integral membrane protein |
| Rv2395A | aprA | acid and phagosome regulated protein AprA |
| Rv0236A | Rv0236A | hypothetical protein |
| Rv2401 | Rv2401 | hypothetical protein |
| Rv2401A | Rv2401A | membrane protein |
| Rv2410c | Rv2410c | hypothetical protein |
| Rv2412 | rpsT | 30S ribosomal protein S20 |
| Rv2414c | Rv2414c | hypothetical protein |
| Rv2415c | Rv2415c | hypothetical protein |
| Rv0238 | Rv0238 | transcriptional regulator |
| Rv2422 | Rv2422 | hypothetical protein |
| Rv2425c | Rv2425c | hypothetical protein |
| Rv2427A | oxyR' | Rv2427A |
| Rv0239 | vapB24 | antitoxin VapB24 |
| Rv2428 | ahpC | alkyl hydroperoxide reductase subunit AhpC |
| Rv2429 | ahpD | alkyl hydroperoxide reductase AphD |
| Rv2430c | PPE41 | PPE family protein PPE41 |
| Rv2431c | PE25 | PE family protein PE25 |
| Rv0240 | vapC24 | ribonuclease VapC24 |
| Rv2438c | nadE | glutamine-dependent NAD(+) synthetase |
| Rv2441c | rpmA | 50S ribosomal protein L27 |
| Rv2443 | dctA | C4-dicarboxylate-transport transmembrane protein DctA |
| Rv2444c | rne | ribonuclease E |
| Rv2445c | ndkA | nucleoside diphosphate kinase |
| Rv0241c | htdX | 3-hydroxyacyl-thioester dehydratase HtdX |
| Rv2450c | rpfE | resuscitation-promoting factor RpfE |
| Rv2451 | Rv2451 | hypothetical protein |
| Rv2454c | Rv2454c | 2-oxoglutarate oxidoreductase subunit KorB |
| Rv2455c | Rv2455c | 2-oxoglutarate oxidoreductase subunit KorA |
| Rv2456c | Rv2456c | MFS-type transporter |
| Rv0242c | fabG4 | 3-oxoacyl-ACP reductase FabG |
| Rv2457c | clpX | ATP-dependent CLP protease ATP-binding subunit ClpX |
| Rv2459 | Rv2459 | MFS-type transporter |
| Rv2460c | clpP2 | ATP-dependent CLP protease proteolytic subunit 2 |
| Rv2461c | clpP1 | ATP-dependent CLP protease proteolytic subunit 1 |
| Rv2463 | lipP | esteraselipase LipP |
| Rv0243 | fadA2 | acetyl-CoA acetyltransferase FadA |
| Rv2468A | Rv2468A | hypothetical protein |
| Rv2470 | glbO | hemoglobin GlbO |
| RVnc0008 | F6 | 0 |
| Rv2474c | Rv2474c | hypothetical protein |
| Rv2475c | Rv2475c | hypothetical protein |
| Rv2477c | Rv2477c | macrolide ABC transporter ATP-binding protein |
| Rv0244c | fadE5 | acyl-CoA dehydrogenase FadE5 |
| Rvnt28 | argW | 0 |
| Rv2488c | Rv2488c | LuxR family transcriptional regulator |
| Rv2489c | Rv2489c | hypothetical protein |
| Rv2490c | PE_PGRS43 | PE-PGRS family protein PE_PGRS43 |
| Rv2490a | Rv2490a | hypothetical protein |
| Rv2495c | bkdC | branched-chain keto acid dehydrogenase E2 component |
| Rv2496c | bkdB | 3-methyl-2-oxobutanoate dehydrogenase subunit beta |
| Rv2497c | bkdA | 3-methyl-2-oxobutanoate dehydrogenase subunit alpha |
| Rv2500c | fadE19 | acyl-CoA dehydrogenase FadE19 |
| Rv2503c | scoB | succinyl-CoA:3-ketoacid-CoA transferase subunit B |
| Rv0246 | Rv0246 | integral membrane protein |
| Rv2504c | scoA | succinyl-CoA:3-ketoacid-CoA transferase subunit A |
| Rv2510c | Rv2510c | hypothetical protein |
| Rvnt29 | hisT | 0 |
| Rv2512c | Rv2512c | insertion sequence element IS1081 transposase |
| Rv0247c | Rv0247c | succinate dehydrogenase iron-sulfur subunit |
| Rv2513 | Rv2513 | hypothetical protein |
| Rv2515c | Rv2515c | hypothetical protein |
| Rv2516c | Rv2516c | hypothetical protein |
| Rv2518c | ldtB | L%2CD-transpeptidase LdtB |
| Rvnt30 | lysU | 0 |
| Rv0248c | Rv0248c | succinate dehydrogenase flavoprotein subunit |
| Rv2524c | fas | fatty acid synthase |
| Rv2525c | Rv2525c | hypothetical protein |
| Rv2527 | vapC17 | ribonuclease VapC17 |
| Rv2529 | Rv2529 | hypothetical protein |
| Rv2530c | vapC39 | ribonuclease VapC39 |
| Rv0249c | Rv0249c | succinate dehydrogenase membrane anchor subunit |
| Rv2530A | vapB39 | antitoxin VapB39 |
| Rv2531c | adi | amino acid decarboxylase |
| Rv2532c | Rv2532c | hypothetical protein |
| Rv2536 | Rv2536 | transmembrane protein |
| Rv2537c | aroD | 3-dehydroquinate dehydratase |
| Rv0024 | Rv0024 | NLP/P60 family protein |
| Rv2544 | lppB | lipoprotein LppB |
| Rv2545 | vapB18 | antitoxin VapB18 |
| Rv2547 | vapB19 | antitoxin VapB19 |
| Rv2548 | vapC19 | ribonuclease VapC19 |
| Rv2550c | vapB20 | antitoxin VapB20 |
| Rv2551c | Rv2551c | hypothetical protein |
| Rv2553c | Rv2553c | membrane protein |
| Rv2554c | Rv2554c | Holliday junction resolvase |
| Rv2556c | Rv2556c | hypothetical protein |
| Rv2560 | Rv2560 | hypothetical protein |
| Rv0253 | nirD | nitrite reductase small subunit NirD |
| Rv2573 | Rv2573 | 2-dehydropantoate 2-reductase |
| Rv2575 | Rv2575 | membrane protein |
| Rv0254c | cobU | bifunctional cobinamide kinasecobinamide phosphate guanylyltransferase |
| Rv2581c | Rv2581c | glyoxalase II |
| Rv2583c | relA | bifunctional (p)ppGpp synthase/hydrolase RelA |
| Rv2584c | apt | adenine phosphoribosyltransferase |
| Rv2585c | Rv2585c | lipoprotein |
| Rv2586c | secF | protein translocase subunit SecF |
| Rv2587c | secD | protein translocase subunit SecD |
| Rv2588c | yajC | membrane protein secretion factor YajC |
| Rv2590 | fadD9 | fatty-acid--CoA ligase FadD9 |
| Rv2595 | vapB40 | antitoxin VapB40 |
| Rv2596 | vapC40 | ribonuclease VapC40 |
| Rv2599 | Rv2599 | membrane protein |
| Rv2601 | speE | spermidine synthase |
| Rv2602 | vapC41 | ribonuclease VapC41 |
| Rv2603c | Rv2603c | transcriptional regulator |
| Rv2604c | snoP | glutamine amidotransferase SnoP |
| Rv2605c | tesB2 | acyl-CoA thioesterase II |
| Rv2606c | snzP | pyridoxine biosynthesis protein |
| Rv2607 | pdxH | pyridoxine/pyridoxamine 5'-phosphate oxidase |
| Rv2608 | PPE42 | PPE family protein PPE42 |
| Rv0257 | Rv0257 | hypothetical protein |
| Rv2615c | PE_PGRS45 | PE-PGRS family protein PE_PGRS45 |
| Rv2616 | Rv2616 | hypothetical protein |
| Rv0258c | Rv0258c | hypothetical protein |
| Rv2620c | Rv2620c | transmembrane protein |
| Rv2627c | Rv2627c | hypothetical protein |
| Rv2628 | Rv2628 | hypothetical protein |
| Rv2629 | Rv2629 | hypothetical protein |
| Rv2632c | Rv2632c | hypothetical protein |
| Rv2635 | Rv2635 | hypothetical protein |
| Rv2637 | dedA | transmembrane protein DedA |
| Rv2638 | Rv2638 | hypothetical protein |
| Rv2642 | Rv2642 | ArsR family transcriptional regulator |
| Rvnt33 | cysU | 0 |
| Rvnt34 | valU | 0 |
| Rv2645 | Rv2645 | hypothetical protein |
| Rv2647 | Rv2647 | hypothetical protein |
| Rv2650c | Rv2650c | prophage protein |
| Rv2653c | Rv2653c | toxin |
| Rv2654c | Rv2654c | antitoxin |
| Rv0262c | aac | aminoglycoside 2'-N-acetyltransferase |
| Rv2660c | Rv2660c | hypothetical protein |
| Rv2663 | Rv2663 | hypothetical protein |
| Rv2667 | clpC2 | ATP-dependent protease ATP-binding subunit ClpC |
| Rv2668 | Rv2668 | hypothetical protein |
| Rv2670c | Rv2670c | hypothetical protein |
| Rv2671 | ribD | bifunctional diaminohydroxyphosphoribosylaminopyrimidine deaminase/5-amino-6-(5-phosphoribosylamino)uracil reductase |
| Rv2674 | msrB | peptide methionine sulfoxide reductase MsrB |
| Rv0264c | Rv0264c | hypothetical protein |
| Rv2680 | Rv2680 | hypothetical protein |
| Rv2687c | Rv2687c | antibiotic ABC transporter permease |
| Rv2695 | Rv2695 | hypothetical protein |
| Rv2696c | Rv2696c | hypothetical protein |
| Rv2698 | Rv2698 | transmembrane protein |
| Rv2699c | Rv2699c | hypothetical protein |
| Rv2701c | suhB | inositol-1-monophosphatase SuhB |
| Rv0267 | narU | nitrite extrusion protein NarU |
| Rv2707 | Rv2707 | hypothetical protein |
| Rv2710 | sigB | RNA polymerase sigma factor SigB |
| Rv2711 | ideR | iron-dependent repressor and activator IdeR |
| Rv2713 | sthA | pyridine nucleotide transhydrogenase |
| Rv2714 | Rv2714 | hypothetical protein |
| Rv2715 | Rv2715 | hydrolase |
| Rv0268c | Rv0268c | hypothetical protein |
| Rv2721c | Rv2721c | hypothetical protein |
| Rv2727c | miaA | tRNA delta(2)-isopentenylpyrophosphate transferase |
| Rv2732c | Rv2732c | transmembrane protein |
| Rv2733c | Rv2733c | (dimethylallyl)adenosine tRNA methylthiotransferase |
| Rv2734 | Rv2734 | hypothetical protein |
| Rv0270 | fadD2 | fatty-acid--CoA ligase FadD2 |
| Rv2737c | recA | recombinase A |
| Rv2738c | Rv2738c | hypothetical protein |
| Rv2740 | ephG | epoxide hydrolase |
| Rv2744c | 35kd_ag | hypothetical protein |
| Rv2745c | clgR | transcriptional regulator ClgR |
| Rv2747 | argA | L-glutamate alpha-N-acetyltranferase |
| Rv2749 | Rv2749 | hypothetical protein |
| Rv2752c | Rv2752c | ribonuclease J |
| Rv2753c | dapA | 4-hydroxy-tetrahydrodipicolinate synthase |
| Rv0272c | Rv0272c | hypothetical protein |
| Rv2759c | vapC42 | ribonuclease VapC42 |
| Rv2760c | vapB42 | antitoxin VapB42 |
| Rv2762c | Rv2762c | hypothetical protein |
| Rv2765 | Rv2765 | hydrolase |
| Rv2766c | fabG5 | short-chain type dehydrogenase/reductase |
| Rv2767c | Rv2767c | membrane protein |
| Rv2771c | Rv2771c | hypothetical protein |
| Rv2773c | dapB | 4-hydroxy-tetrahydrodipicolinate reductase |
| Rv2774c | Rv2774c | hypothetical protein |
| Rv2775 | Rv2775 | GCN5-like N-acetyltransferase |
| Rv2779c | Rv2779c | Lrp/AsnC family transcriptional regulator |
| Rv2781c | Rv2781c | oxidoreductase |
| Rv2784c | lppU | lipoprotein LppU |
| Rv2785c | rpsO | 30S ribosomal protein S15 |
| Rv2788 | sirR | transcriptional repressor SirR |
| Rv2789c | fadE21 | acyl-CoA dehydrogenase FadE21 |
| Rv2790c | ltp1 | lipid-transfer protein |
| Rv2791c | Rv2791c | transposase |
| Rv2797c | Rv2797c | hypothetical protein |
| Rv2801A | mazE9 | antitoxin MazE9 |
| Rv2806 | Rv2806 | membrane protein |
| Rv2808 | Rv2808 | hypothetical protein |
| Rv2811 | Rv2811 | hypothetical protein |
| Rv2812 | Rv2812 | transposase |
| Rv2813 | Rv2813 | hypothetical protein |
| Rv2817c | Rv2817c | CRISPR-associated endonuclease Cas1 |
| Rv0277A | vapB25 | Rv0277A |
| Rv2820c | Rv2820c | CRISPR type III-associated RAMP protein Csm4 |
| Rv2828c | Rv2828c | hypothetical protein |
| Rv2828A | Rv2828A | hypothetical protein |
| Rv2831 | echA16 | enoyl-CoA hydratase EchA16 |
| Rv2837c | Rv2837c | bifunctional oligoribonuclease/PAP phosphatase NrnA |
| Rv0279c | PE_PGRS4 | PE-PGRS family protein PE_PGRS4 |
| Rv2840c | Rv2840c | hypothetical protein |
| Rv2843 | Rv2843 | hypothetical protein |
| Rv2844 | Rv2844 | hypothetical protein |
| Rv2846c | efpA | MFS-type transporter EfpA |
| Rv0280 | PPE3 | PPE family protein PPE3 |
| Rv2853 | PE_PGRS48 | PE-PGRS family protein PE_PGRS48 |
| Rv2856 | nicT | nickel-transport integral membrane protein NicT |
| Rv2857c | Rv2857c | 3-oxoacyl-ACP reductase |
| Rv2858c | aldC | aldehyde dehydrogenase AldC |
| Rv2861c | mapB | methionine aminopeptidase |
| Rv2862A | vapB23 | antitoxin VapB23 |
| Rv0282 | eccA3 | ESX-3 secretion system protein EccA |
| Rv2868c | gcpE | 4-hydroxy-3-methylbut-2-en-1-yl diphosphate synthase (flavodoxin) |
| Rv2869c | rip | zinc metalloprotease |
| Rv2871 | vapB43 | antitoxin VapB43 |
| Rv2876 | Rv2876 | transmembrane protein |
| Rv0283 | eccB3 | ESX-3 secretion system protein EccB3 |
| Rv2878c | mpt53 | soluble secreted antigen Mpt53 |
| Rv2881c | cdsA | phosphatidate cytidylyltransferase |
| Rv2882c | frr | ribosome recycling factor |
| Rv2883c | pyrH | uridylate kinase |
| Rv2885c | Rv2885c | transposase |
| Rv2888c | amiC | amidase AmiC |
| Rv0284 | eccC3 | ESX-3 secretion system protein EccC3 |
| Rv2890c | rpsB | 30S ribosomal protein S2 |
| Rv2891 | Rv2891 | hypothetical protein |
| Rv2893 | Rv2893 | oxidoreductase |
| Rv2894c | xerC | tyrosine recombinase XerC |
| Rv2896c | Rv2896c | hypothetical protein |
| Rv2899c | fdhD | formate dehydrogenase accessory protein FdhD |
| Rv2900c | fdhF | formate dehydrogenase subunit alpha FdhF |
| Rv2901c | Rv2901c | hypothetical protein |
| Rv2902c | rnhB | ribonuclease HII |
| Rv2903c | lepB | signal peptidase |
| Rv2904c | rplS | 50S ribosomal protein L19 |
| Rv2906c | trmD | tRNA (guanine-N1)-methyltransferase |
| Rv2907c | rimM | 16S rRNA processing protein RimM |
| Rv0286 | PPE4 | PPE family protein PPE4 |
| Rv2911 | dacB2 | penicillin-binding protein DacB2 |
| Rv2913c | Rv2913c | D-amino acid aminohydrolase |
| Rv2919c | glnB | nitrogen regulatory protein P-II |
| Rv0287 | esxG | ESAT-6 like protein EsxG |
| Rv2920c | amt | ammonium transporter integral membrane protein |
| Rv2925c | rnc | ribonuclease III |
| Rv2926c | Rv2926c | hypothetical protein |
| Rv2927c | Rv2927c | hypothetical protein |
| Rv2928 | tesA | thioesterase TesA |
| Rv0288 | esxH | ESAT-6-like protein EsxH |
| Rv2929 | Rv2929 | hypothetical protein |
| Rv2930 | fadD26 | fatty-acid--CoA ligase FadD26 |
| Rv2932 | ppsB | phthiocerol synthesis polyketide synthase type I PpsB |
| Rv2936 | drrA | daunorubicin ABC transporter ATP-binding protein DrrA |
| Rv2937 | drrB | daunorubicin ABC transporter permease DrrB |
| Rv2938 | drrC | daunorubicin ABC transporter permease DrrC |
| Rv0289 | espG3 | ESX-3 secretion-associated protein EspG3 |
| Rv2939 | papA5 | phthiocerol/phthiodiolone dimycocerosyl transferase |
| Rv2940c | mas | multifunctional mycocerosic acid synthase |
| Rv2941 | fadD28 | long-chain-fatty-acid--AMP ligase FadD28 |
| Rv2942 | mmpL7 | transmembrane transport protein MmpL7 |
| Rv2944 | Rv2944 | insertion sequence element IS1533 transposase |
| Rv2945c | lppX | lipoprotein LppX |
| Rv2948c | fadD22 | p-hydroxybenzoyl--AMP ligase |
| Rv2949c | Rv2949c | chorismate pyruvate-lyase |
| Rv2950c | fadD29 | long-chain-fatty-acid--AMP ligase FadD29 |
| Rv2951c | Rv2951c | phthiodiolone/phenolphthiodiolone dimycocerosates ketoreductase |
| Rv2953 | Rv2953 | trans-acting enoyl reductase |
| Rv2954c | Rv2954c | hypothetical protein |
| Rv2956 | Rv2956 | hypothetical protein |
| Rv2957 | Rv2957 | PGL/p-HBAD biosynthesis glycosyltransferase |
| Rv2959c | Rv2959c | rhamnosyl O-methyltransferase |
| Rv2960c | Rv2960c | hypothetical protein |
| Rv2962c | Rv2962c | PGL/p-HBAD biosynthesis rhamnosyltransferase |
| Rv2965c | kdtB | phosphopantetheine adenylyltransferase |
| Rv0292 | eccE3 | ESX-3 secretion system protein EccE |
| Rv2968c | Rv2968c | integral membrane protein |
| Rv2970c | lipN | lipaseesterase LipN |
| Rv2970A | Rv2970A | hypothetical protein |
| Rv2971 | Rv2971 | oxidoreductase |
| Rv2985 | mutT1 | 8-oxo-dGTP diphosphatase |
| Rv0294 | tam | trans-aconitate methyltransferase |
| Rv2986c | hupB | DNA-binding protein HU |
| Rv2987c | leuD | 3-isopropylmalate dehydratase small subunit |
| Rv2988c | leuC | 3-isopropylmalate dehydratase large subunit |
| Rv2990c | Rv2990c | hypothetical protein |
| Rv2993c | Rv2993c | 2-hydroxyhepta-2%2C4-diene-1%2C7-dioate isomerase |
| Rv2995c | leuB | 3-isopropylmalate dehydrogenase |
| Rv2997 | Rv2997 | dehydrogenase |
| Rv2998 | Rv2998 | hypothetical protein |
| Rv2999 | lppY | lipoprotein LppY |
| Rv3001c | ilvC | ketol-acid reductoisomerase |
| Rv3002c | ilvN | acetolactate synthase small subunit |
| Rv3004 | cfp6 | low molecular weight protein antigen 6 |
| Rv3006 | lppZ | lipoprotein LppZ |
| Rv3007c | Rv3007c | oxidoreductase |
| Rv3008 | Rv3008 | hypothetical protein |
| Rv3010c | pfkA | 6-phosphofructokinase |
| Rv3012c | gatC | glutamyl-tRNA(GLN) amidotransferase subunit C |
| Rv3014c | ligA | DNA ligase A |
| Rv3019c | esxR | ESAT-6 like protein EsxR |
| Rv3022c | PPE48 | Rv3022c |
| Rv3022A | PE29 | PE family protein PE29 |
| Rv0298 | Rv0298 | antitoxin |
| Rv3023c | Rv3023c | transposase |
| Rv3025c | iscS | cysteine desulfurase |
| Rv3029c | fixA | electron transfer flavoprotein subunit beta |
| Rv0299 | Rv0299 | toxin |
| Rv3039c | echA17 | enoyl-CoA hydratase EchA17 |
| Rv3040c | Rv3040c | hypothetical protein |
| Rv0300 | vapB2 | antitoxin VapB2 |
| Rv3043c | ctaD | cytochrome C oxidase cytochrome 1 |
| Rv3045 | adhC | NADP-dependent alcohol dehydrogenase |
| Rv3046c | Rv3046c | hypothetical protein |
| Rv3048c | nrdF2 | ribonucleoside-diphosphate reductase subunit beta NrdF2 |
| Rv3049c | Rv3049c | monooxygenase |
| Rv3050c | Rv3050c | AsnC family transcriptional regulator |
| Rv3051c | nrdE | ribonucleoside-diphosphate reductase subunit alpha |
| Rv0301 | vapC2 | ribonuclease VapC2 |
| Rv3052c | nrdI | NrdI protein |
| Rv3053c | nrdH | glutaredoxin electron transport protein NrdH |
| Rv3056 | dinP | DNA polymerase IV 2 |
| Rv3057c | Rv3057c | short chain alcohol dehydrogenasereductase |
| Rv3060c | Rv3060c | GntR family transcriptional regulator |
| Rv3073c | Rv3073c | hypothetical protein |
| Rv3075c | Rv3075c | hypothetical protein |
| Rv3076 | Rv3076 | hypothetical protein |
| Rv3078 | hab | hydroxylaminobenzene mutase |
| Rv3080c | pknK | serinethreonine-protein kinase PknK |
| Rv3086 | adhD | alcohol dehydrogenase D |
| Rv3087 | Rv3087 | diacyglycerol O-acyltransferase |
| Rv3092c | Rv3092c | integral membrane protein |
| Rv3093c | Rv3093c | oxidoreductase |
| Rv3095 | Rv3095 | HTH-type transcriptional regulator |
| Rv3096 | Rv3096 | hypothetical protein |
| Rv3098c | Rv3098c | hypothetical protein |
| Rv3098A | Rv3098A | PemK-like protein |
| RVnc0046 | ssr | #N/A |
| Rv3101c | ftsX | cell division protein FtsX |
| Rv3103c | Rv3103c | hypothetical protein |
| Rv3106 | fprA | NADPH-ferredoxin reductase FprA |
| Rv0307c | Rv0307c | hypothetical protein |
| Rv3115 | Rv3115 | transposase |
| Rv3116 | moeB2 | molybdenum cofactor biosynthesis protein MoeB |
| Rv0308 | Rv0308 | integral membrane protein |
| Rv3119 | moaE1 | molybdopterin synthase catalytic subunit 1 |
| Rv3123 | Rv3123 | hypothetical protein |
| Rv3124 | moaR1 | transcriptional regulator MoaR |
| Rv3127 | Rv3127 | hypothetical protein |
| Rv3128c | Rv3128c | Rv3128c |
| Rv0309 | Rv0309 | hypothetical protein |
| Rv3129 | Rv3129 | Rv3129 |
| Rv3130c | tgs1 | diacyglycerol O-acyltransferase |
| Rv3131 | Rv3131 | NAD(P)H nitroreductase |
| Rv3132c | devS | two component sensor histidine kinase DevS |
| Rv3133c | devR | two component transcriptional regulator DevR |
| Rv3134c | Rv3134c | universal stress protein |
| Rv3135 | PPE50 | PPE family protein PPE50 |
| Rv3136 | PPE51 | PPE family protein PPE51 |
| Rv3136A | Rv3136A | hypothetical protein |
| Rv0310c | Rv0310c | hypothetical protein |
| Rv3138 | pflA | pyruvate formate lyase activating protein PflA |
| Rv3140 | fadE23 | acyl-CoA dehydrogenase FadE23 |
| Rv3141 | fadB4 | NADPH quinone oxidoreductase FadB |
| Rv3142c | Rv3142c | hypothetical protein |
| Rv3143 | Rv3143 | response regulator |
| Rv3145 | nuoA | NADH-quinone oxidoreductase subunit A |
| Rv3146 | nuoB | NADH-quinone oxidoreductase subunit B |
| Rv3147 | nuoC | NADH-quinone oxidoreductase subunit C |
| Rv3148 | nuoD | NADH-quinone oxidoreductase subunit D |
| Rv3149 | nuoE | NADH-quinone oxidoreductase subunit E |
| Rv3151 | nuoG | NADH-quinone oxidoreductase subunit G |
| Rv3152 | nuoH | NADH-quinone oxidoreductase subunit H |
| Rv3154 | nuoJ | NADH-quinone oxidoreductase subunit J |
| Rv3155 | nuoK | NADH-quinone oxidoreductase subunit K |
| Rv3156 | nuoL | NADH-quinone oxidoreductase subunit L |
| Rv3157 | nuoM | NADH-quinone oxidoreductase subunit M |
| Rv0312 | Rv0312 | hypothetical protein |
| Rv3158 | nuoN | NADH-quinone oxidoreductase subunit N |
| Rv3159c | PPE53 | PPE family protein PPE53 |
| Rv3160c | Rv3160c | TetR family transcriptional regulator |
| Rv3161c | Rv3161c | dioxygenase |
| Rv3163c | Rv3163c | hypothetical protein |
| Rv3166c | Rv3166c | hypothetical protein |
| Rv3167c | Rv3167c | TetR family transcriptional regulator |
| Rv0313 | Rv0313 | hypothetical protein |
| Rv3169 | Rv3169 | hypothetical protein |
| Rv0314c | Rv0314c | membrane protein |
| Rv3178 | Rv3178 | nitroreductase |
| Rv3180c | Rv3180c | ribonuclease VapC45 |
| Rv0315 | Rv0315 | beta-1%2C3-glucanase |
| Rv3190A | Rv3190A | hypothetical protein |
| Rv3191c | Rv3191c | transposase |
| Rv3193c | Rv3193c | transmembrane protein |
| Rv3196 | Rv3196 | hypothetical protein |
| Rv3196A | Rv3196A | hypothetical protein |
| Rv3197 | Rv3197 | ABC transporter ATP-binding protein |
| Rv3197A | whiB7 | transcriptional regulator WhiB7 |
| Rv3198A | Rv3198A | glutaredoxin protein |
| Rv3202a | Rv3202a | hypothetical protein |
| Rv3203 | lipV | lipase LipV |
| Rv3205c | Rv3205c | hypothetical protein |
| Rv3207c | Rv3207c | hypothetical protein |
| Rv3208 | Rv3208 | TetR family transcriptional regulator |
| Rv3208A | TB9.4 | hypothetical protein |
| Rv3209 | Rv3209 | hypothetical protein |
| Rv3211 | rhlE | ATP-dependent RNA helicase RhlE |
| Rv3212 | Rv3212 | hypothetical protein |
| Rv3213c | Rv3213c | SOJ/ParA-like protein |
| Rv3215 | entC | isochorismate synthase |
| Rv3216 | Rv3216 | Rv3216 |
| Rv3217c | Rv3217c | integral membrane protein |
| Rv3219 | whiB1 | transcriptional regulator WhiB1 |
| Rv3220c | Rv3220c | two component sensor kinase |
| Rv3221c | TB7.3 | acetyl-CoA carboxylase biotin carboxyl carrier protein subunit |
| Rv3221A | rshA | anti-sigma factor RshA |
| Rv3222c | Rv3222c | hypothetical protein |
| Rv3224 | Rv3224 | iron-regulated short-chain dehydrogenasereductase |
| Rv3228 | Rv3228 | hypothetical protein |
| Rv3229c | desA3 | stearoyl-CoA 9-desaturase |
| Rv3230c | Rv3230c | stearoyl-CoA 9-desaturase electron transfer protein |
| Rv3232c | ppk2 | polyphosphate kinase |
| Rv3235 | Rv3235 | hypothetical protein |
| Rv3236c | kefB | integral membrane transport protein |
| Rv3241c | Rv3241c | hypothetical protein |
| Rv3245c | mtrB | two component sensory histidine kinase MtrB |
| Rv3246c | mtrA | two component DNA-binding response regulator MtrA |
| Rv3248c | sahH | adenosylhomocysteinase |
| Rv3252c | alkB | transmembrane alkane 1-monooxygenase AlkB |
| Rv3253c | Rv3253c | cationic amino acid transport integral membrane protein |
| Rv3255c | manA | mannose-6-phosphate isomerase |
| Rv3257c | pmmA | phosphomannomutase PmmA |
| Rv3259 | Rv3259 | hypothetical protein |
| Rv3260c | whiB2 | transcriptional regulator WhiB2 |
| Rv0322 | udgA | UDP-glucose 6-dehydrogenase UdgA |
| Rv3264c | manB | D-alpha-D-mannose-1-phosphate guanylyltransferase ManB |
| Rv3267 | Rv3267 | hypothetical protein |
| Rv3269 | Rv3269 | hypothetical protein |
| Rv3270 | ctpC | manganese/zinc-exporting P-type ATPase |
| Rv3271c | Rv3271c | integral membrane protein |
| Rv3272 | Rv3272 | hypothetical protein |
| Rv3273 | Rv3273 | transmembrane carbonic anhydrase |
| Rv3274c | fadE25 | acyl-CoA dehydrogenase |
| Rv3277 | Rv3277 | transmembrane protein |
| Rv3278c | Rv3278c | transmembrane protein |
| Rv3280 | accD5 | propionyl-CoA carboxylase subunit beta |
| Rv3281 | accE5 | bifunctional protein acetyl-propionyl-CoAcarboxylase subunit epsilon AccE |
| Rv3282 | Rv3282 | hypothetical protein |
| Rv0324 | Rv0324 | transcriptional regulator |
| Rv3284 | Rv3284 | hypothetical protein |
| Rv3285 | accA3 | bifunctional protein acetyl-propionyl-CoA carboxylase subunit alpha AccA |
| Rv3286c | sigF | RNA polymerase sigma factor SigF |
| Rv3288c | usfY | hypothetical protein |
| Rv3289c | Rv3289c | transmembrane protein |
| Rv3291c | lrpA | transcriptional regulator LrpA |
| Rv3293 | pcd | piperideine-6-carboxylic acid dehydrogenase |
| Rv3294c | Rv3294c | hypothetical protein |
| Rv3295 | Rv3295 | TetR family transcriptional regulator |
| Rv3299c | atsB | arylsulfatase AtsB |
| Rv0326 | Rv0326 | hypothetical protein |
| Rv3309c | upp | uracil phosphoribosyltransferase |
| Rv3316 | sdhC | succinate dehydrogenase cytochrome B-556 subunit |
| Rv3317 | sdhD | succinate dehydrogenase hydrophobic membrane anchor subunit |
| Rv3320c | vapC44 | ribonuclease VapC44 |
| Rv3321c | vapB44 | antitoxin VapB44 |
| Rv3323c | moaX | MoaD-MoaE fusion protein MoaX |
| Rv3324A | Rv3324A | Rv3324A |
| Rv3334 | Rv3334 | MerR family transcriptional regulator |
| Rv3335c | Rv3335c | integral membrane protein |
| Rv3336c | trpS | tryptophan--tRNA ligase |
| Rv3339c | icd1 | isocitrate dehydrogenase |
| Rv3344c | PE_PGRS49 | PE-PGRS family protein PE_PGRS49 |
| Rv3345c | PE_PGRS50 | PE-PGRS family protein PE_PGRS50 |
| Rv3352c | Rv3352c | oxidoreductase |
| Rv3353c | Rv3353c | hypothetical protein |
| Rv3357 | relJ | antitoxin RelJ |
| Rv3365c | Rv3365c | hypothetical protein |
| Rv3366 | spoU | tRNA/rRNA methylase SpoU |
| Rv0333 | Rv0333 | hypothetical protein |
| Rv3376 | Rv3376 | phosphatase |
| Rv3377c | Rv3377c | type B diterpene cyclase |
| Rv3378c | Rv3378c | diterpene synthase |
| Rv3382c | Rv3382c | 4-hydroxy-3-methylbut-2-enyl diphosphate reductase |
| Rv3388 | PE_PGRS52 | PE-PGRS family protein PE_PGRS52 |
| Rv3390 | lpqD | lipoprotein LpqD |
| Rv3394c | Rv3394c | hypothetical protein |
| Rv3395c | Rv3395c | hypothetical protein |
| Rv0336 | Rv0336 | hypothetical protein |
| Rv3404c | Rv3404c | hypothetical protein |
| Rv3405c | Rv3405c | HTH-type transcriptional regulator |
| Rv3407 | vapB47 | antitoxin VapB47 |
| Rv3408 | vapC47 | ribonuclease VapC47 |
| Rv3409c | choD | cholesterol oxidase |
| Rv3412 | Rv3412 | hypothetical protein |
| Rv3413c | Rv3413c | anti-sigma-D factor RsdA |
| Rv3414c | sigD | ECF RNA polymerase sigma factor SigD |
| Rv3416 | whiB3 | redox-responsive transcriptional regulator WhiB3 |
| Rv3421c | Rv3421c | hypothetical protein |
| Rv0034 | Rv0034 | hypothetical protein |
| Rv0338c | Rv0338c | iron-sulfur-binding reductase |
| Rv3424c | Rv3424c | hypothetical protein |
| Rv3428c | Rv3428c | transposase |
| Rv3429 | PPE59 | PPE family protein PPE59 |
| Rv3430c | Rv3430c | transposase |
| Rv3430a | Rv3430a | hypothetical protein |
| Rv3433c | Rv3433c | bifunctional ADP-dependent (S)-NAD(P)H-hydrate dehydrataseNAD(P)H-hydrate epimerase |
| Rv3435c | Rv3435c | transmembrane protein |
| Rv3440c | Rv3440c | hypothetical protein |
| Rv3443c | rplM | 50S ribosomal protein L13 |
| Rv3445c | esxU | ESAT-6 like protein EsxU |
| Rv3446c | Rv3446c | hypothetical protein |
| Rv3447c | eccC4 | ESX-4 secretion system protein EccC4 |
| Rv3448 | eccD4 | ESX-4 secretion system protein EccD4 |
| Rv3449 | mycP4 | membrane-anchored mycosin |
| Rv3450c | eccB4 | ESX-4 secretion system protein EccB4 |
| Rv3452 | cut4 | cutinase |
| Rv3457c | rpoA | DNA-directed RNA polymerase subunit alpha |
| Rv3458c | rpsD | 30S ribosomal protein S4 |
| Rv3459c | rpsK | 30S ribosomal protein S11 |
| Rv3460c | rpsM | 30S ribosomal protein S13 |
| Rv3461c | rpmJ | 50S ribosomal protein L36 |
| Rv3462c | infA | translation initiation factor IF-1 |
| Rv3464 | rmlB | dTDP-glucose 4%2C6-dehydratase |
| Rv3465 | rmlC | dTDP-4-dehydrorhamnose 3%2C5-epimerase |
| Rv3466 | Rv3466 | hypothetical protein |
| Rv3473c | bpoA | peroxidase BpoA |
| Rv3477 | PE31 | PE family protein PE31 |
| Rv3478 | PPE60 | PE family protein PPE60 |
| Rv3480c | Rv3480c | diacyglycerol O-acyltransferase |
| Rv3483c | Rv3483c | hypothetical protein |
| Rv3484 | Rv3484 | hypothetical protein |
| Rv3486 | Rv3486 | hypothetical protein |
| Rv0344c | lpqJ | lipoprotein LpqJ |
| Rv3487c | lipF | carboxylesterase LipF |
| Rv3491 | Rv3491 | hypothetical protein |
| Rv3492c | Rv3492c | Mce associated protein |
| Rv3493c | Rv3493c | Mce associated protein |
| Rv3495c | lprN | Mce family lipoprotein LprN |
| Rv0345 | Rv0345 | hypothetical protein |
| Rv3499c | mce4A | Mce family protein Mce4A |
| Rv3500c | yrbE4B | integral membrane protein |
| Rv3503c | fdxD | ferredoxin FdxD |
| Rv3506 | fadD17 | long-chain-fatty-acid--CoA ligase FadD17 |
| Rv0346c | ansP2 | L-asparagine permease |
| Rv3508 | PE_PGRS54 | PE-PGRS family protein PE_PGRS54 |
| Rv3512 | PE_PGRS56 | PE-PGRS family protein PE_PGRS56 |
| Rv3513c | fadD18 | fatty-acid--CoA ligase FadD18 |
| Rv3514 | PE_PGRS57 | PE-PGRS family protein PE_PGRS57 |
| Rv3515c | fadD19 | acyl-CoA synthetase |
| Rv3520c | Rv3520c | coenzyme F420-dependent oxidoreductase |
| Rv3524 | Rv3524 | membrane protein |
| Rv3526 | kshA | 3-ketosteroid-9-alpha-monooxygenase oxygenase subunit |
| Rv3527 | Rv3527 | hypothetical protein |
| Rv3528c | Rv3528c | hypothetical protein |
| Rv3538 | hsd4B | dehydrogenase |
| Rv3541c | Rv3541c | hypothetical protein |
| Rv3545c | cyp125 | steroid C26-monooxygenase |
| Rv3546 | fadA5 | acetyl-CoA acetyltransferase FadA |
| Rv0350 | dnaK | chaperone protein DnaK |
| Rv3547 | ddn | deazaflavin-dependent nitroreductase |
| Rv3550 | echA20 | enoyl-CoA hydratase EchA20 |
| Rv0351 | grpE | stress response protein GrpE |
| Rv3563 | fadE32 | acyl-CoA dehydrogenase FadE32 |
| Rv3566c | nat | arylamine N-acetyltransferase |
| Rv0352 | dnaJ1 | chaperone protein DnaJ |
| Rv3567c | hsaB | flavin-dependent monooxygenase reductase subunit HsaB |
| Rv3569c | hsaD | 4%2C5-9%2C10-diseco-3-hydroxy-5%2C9%2C17-trioxoandrosta-1(10)%2C2-diene-4-oate hydrolase |
| Rv3572 | Rv3572 | hypothetical protein |
| Rv3575c | Rv3575c | LacI family transcriptional regulator |
| Rv3576 | lppH | lipoprotein LppH |
| Rv3579c | Rv3579c | 23S rRNA (guanosine(2251)-2'-O)-methyltransferase RlmB |
| Rv3581c | ispF | 2C-methyl-D-erythritol 2%2C4-cyclodiphosphate synthase |
| Rv3582c | ispD | 2-C-methyl-D-erythritol 4-phosphate cytidylyltransferase |
| Rv3583c | Rv3583c | RNA polymerase-binding transcription factor CarD |
| Rv3584 | lpqE | lipoprotein LpqE |
| Rv0354c | PPE7 | PPE family protein PPE7 |
| Rv3596c | clpC1 | ATP-dependent protease ATP-binding subunit ClpC |
| Rv3597c | lsr2 | iron-regulated H-NS-like protein |
| Rv3603c | Rv3603c | hypothetical protein |
| Rv3614c | espD | ESX-1 secretion-associated protein EspD |
| Rv3615c | espC | ESX-1 secretion-associated protein EspC |
| Rv3616c | espA | ESX-1 secretion-associated protein EspA |
| Rv3619c | esxV | ESAT-6 like protein EsxV |
| Rv3620c | esxW | ESAT-6 like protein EsxW |
| Rv3623 | lpqG | lipoprotein LpqG |
| Rv3624c | hpt | hypoxanthine-guanine phosphoribosyltransferase |
| Rv3627c | Rv3627c | hypothetical protein |
| Rv0036c | Rv0036c | hypothetical protein |
| Rv0358 | Rv0358 | hypothetical protein |
| Rv3632 | Rv3632 | membrane protein |
| Rv3634c | galE1 | UDP-glucose 4-epimerase |
| Rv3636 | Rv3636 | Rv3636 |
| Rv3645 | Rv3645 | transmembrane protein |
| Rv3648c | cspA | cold shock protein A |
| Rv3650 | PE33 | PE family protein PE33 |
| Rv3652 | PE_PGRS60 | PE-PGRS family-related protein PE_PGRS60 |
| Rv3653 | PE_PGRS61 | PE-PGRS family-related protein PE_PGRS61 |
| Rv3654c | Rv3654c | hypothetical protein |
| Rv3655c | Rv3655c | hypothetical protein |
| Rv3657c | Rv3657c | membrane protein |
| Rv0361 | Rv0361 | membrane protein |
| Rv3658c | Rv3658c | transmembrane protein |
| Rv3660c | Rv3660c | hypothetical protein |
| RVnc0036a | MTS2823 | 0 |
| Rv3662c | Rv3662c | hypothetical protein |
| Rv3664c | dppC | dipeptide ABC transporter permease DppC |
| Rv3665c | dppB | dipeptide ABC transporter permease DppB |
| Rv3668c | Rv3668c | protease |
| Rv3669 | Rv3669 | transmembrane protein |
| Rv3671c | Rv3671c | serine protease |
| Rv3675 | Rv3675 | membrane protein |
| Rv0363c | fba | fructose-bisphosphate aldolase |
| Rv3676 | crp | cAMP receptor protein |
| Rv3677c | Rv3677c | beta lactamase |
| Rv3678A | Rv3678A | hypothetical protein |
| Rv3679 | Rv3679 | anion transporter ATPase |
| Rv3680 | Rv3680 | anion transporter ATPase |
| Rv3681c | whiB4 | transcriptional regulator WhiB4 |
| Rv3682 | ponA2 | bifunctional penicillin-insensitive transglycosylase/penicillin-sensitive transpeptidase |
| Rv3684 | Rv3684 | lyase |
| Rv0364 | Rv0364 | transmembrane protein |
| Rvnt40 | proY | 0 |
| Rv3685c | cyp137 | cytochrome P450 Cyp137 |
| Rv3686c | Rv3686c | hypothetical protein |
| Rv3691 | Rv3691 | hypothetical protein |
| Rv3693 | Rv3693 | membrane protein |
| Rv3694c | Rv3694c | transmembrane protein |
| Rv3697c | vapC48 | ribonuclease VapC48 |
| Rv3699 | Rv3699 | hypothetical protein |
| Rv3708c | asd | aspartate-semialdehyde dehydrogenase |
| Rv3709c | ask | aspartokinase |
| Rv3711c | dnaQ | DNA polymerase III subunit epsilon |
| Rv3712 | Rv3712 | ligase |
| Rv3715c | recR | recombination protein RecR |
| Rv3719 | Rv3719 | hypothetical protein |
| Rv3720 | Rv3720 | fatty acid synthase |
| Rv3721c | dnaZX | DNA polymerase III subunit gammatau |
| Rv0368c | Rv0368c | hypothetical protein |
| Rv3722c | Rv3722c | hypothetical protein |
| Rvnt41 | serV | 0 |
| Rv3723 | Rv3723 | transmembrane protein |
| Rv3726 | Rv3726 | dehydrogenase |
| Rv3727 | Rv3727 | oxidoreductase |
| Rv3728 | Rv3728 | membrane protein |
| Rv3732 | Rv3732 | hypothetical protein |
| Rv3733c | Rv3733c | hypothetical protein |
| Rv3734c | tgs2 | diacyglycerol O-acyltransferase |
| Rv3736 | Rv3736 | AraC/XylS family transcriptional regulator |
| Rv3738c | PPE66 | PPE family protein PPE66 |
| Rv3739c | PPE67 | PPE family protein PPE67 |
| Rv3740c | Rv3740c | diacyglycerol O-acyltransferase |
| Rv3741c | Rv3741c | oxidoreductase |
| Rv3742c | Rv3742c | oxidoreductase |
| Rv3745c | Rv3745c | hypothetical protein |
| Rv3747 | Rv3747 | hypothetical protein |
| Rv3748 | Rv3748 | hypothetical protein |
| Rv3749c | Rv3749c | hypothetical protein |
| Rv3750c | Rv3750c | excisionase |
| Rv3755c | Rv3755c | hypothetical protein |
| Rv3759c | proX | glycine betainecarnitinecholineL-proline ABC transporter substrate-binding lipoprotein ProX |
| Rv0372c | Rv0372c | hypothetical protein |
| Rv3766 | Rv3766 | hypothetical protein |
| Rv3768 | Rv3768 | hypothetical protein |
| Rv3769 | Rv3769 | hypothetical protein |
| Rv3773c | Rv3773c | hypothetical protein |
| Rv0374c | Rv0374c | carbon monoxyde dehydrogenase small subunit |
| Rv3779 | Rv3779 | transmembrane protein |
| Rv3782 | glfT1 | galactofuranosyl transferase GlfT |
| Rv3783 | rfbD | O-antigenlipopolysaccharide ABC transporter permease RfbD |
| Rv3786c | Rv3786c | hypothetical protein |
| Rv3796 | atsH | hypothetical protein |
| Rv0376c | Rv0376c | hypothetical protein |
| Rv3798 | Rv3798 | insertion sequence element IS1557 transposase |
| Rv3799c | accD4 | propionyl-CoA carboxylase subunit beta AccD |
| Rv3801c | fadD32 | long-chain-fatty-acid--AMP ligase FadD32 |
| Rv3802c | clp6 | membrane protein |
| Rv3803c | fbpD | MPT51MPB51 antigen |
| Rv3804c | fbpA | diacylglycerol acyltransferase/mycolyltransferase Ag85A |
| Rv3805c | aftB | terminal beta-(1->2)-arabinofuranosyltransferase |
| Rv3806c | ubiA | decaprenyl-phosphate phosphoribosyltransferase |
| Rv0377 | Rv0377 | HTH-type transcriptional regulator |
| Rv3810 | pirG | cell surface protein |
| Rv3811 | csp | hypothetical protein |
| Rv3812 | PE_PGRS62 | PE-PGRS family protein PE_PGRS62 |
| Rv3813c | Rv3813c | hypothetical protein |
| Rv3816c | Rv3816c | acyltransferase |
| Rv3817 | Rv3817 | phosphotransferase |
| Rv3819 | Rv3819 | hypothetical protein |
| Rv3820c | papA2 | trehalose-2-sulfate acyltransferase |
| Rv3821 | Rv3821 | integral membrane protein |
| Rv3822 | Rv3822 | hypothetical protein |
| Rv3823c | mmpL8 | integral membrane transport protein MmpL8 |
| Rv3824c | papA1 | acyltransferase |
| Rv3825c | pks2 | phthioceranichydroxyphthioceranic acid synthase |
| Rv3826 | fadD23 | long-chain-fatty-acid--CoA ligase FadD23 |
| Rv3831 | Rv3831 | hypothetical protein |
| Rv3834c | serS | serine--tRNA ligase |
| Rv3835 | Rv3835 | hypothetical protein |
| Rv3838c | pheA | prephenate dehydratase |
| Rv3841 | bfrB | bacterioferritin BfrB |
| Rv3842c | glpQ1 | glycerophosphoryl diester phosphodiesterase |
| Rv3843c | Rv3843c | transmembrane protein |
| Rv3845 | Rv3845 | hypothetical protein |
| Rv3846 | sodA | superoxide dismutase |
| Rv3847 | Rv3847 | hypothetical protein |
| Rv3851 | Rv3851 | membrane protein |
| Rv3853 | rraA | RNase E regulator RraA |
| Rv3854c | ethA | monooxygenase EthA |
| Rv3856c | Rv3856c | hypothetical protein |
| Rv3858c | gltD | glutamate synthase small subunit |
| Rv3859c | gltB | glutamate synthase large subunit |
| Rv3860 | Rv3860 | hypothetical protein |
| Rv3863 | Rv3863 | hypothetical protein |
| Rv3864 | espE | ESX-1 secretion-associated protein EspE |
| Rv3865 | espF | ESX-1 secretion-associated protein EspF |
| Rv3866 | espG1 | ESX-1 secretion-associated protein EspG |
| Rv0383c | Rv0383c | hypothetical protein |
| Rv3868 | eccA1 | ESX-1 secretion system protein EccA1 |
| Rv3869 | eccB1 | ESX-1 secretion system protein EccB |
| Rv3870 | eccCa1 | ESX-1 secretion system protein EccCa |
| Rv3871 | eccCb1 | ESX-1 secretion system protein EccCb |
| Rv3872 | PE35 | PE family protein PE35 |
| Rv3873 | PPE68 | PPE family protein PPE68 |
| Rv3874 | esxB | ESAT-6-like protein EsxB |
| Rv3875 | esxA | ESAT-6 protein EsxA |
| Rv3878 | espJ | ESX-1 secretion-associated protein EspJ |
| Rv3880c | espL | ESX-1 secretion-associated protein EspL |
| Rv3881c | espB | ESX-1 secretion-associated protein EspB |
| Rv3884c | eccA2 | ESX-2 secretion system protein EccA |
| Rv3891c | esxD | ESAT-6 like protein EsxD |
| Rv3894c | eccC2 | ESX-2 type VII secretion system protein EccC |
| Rv3895c | eccB2 | ESX-2 secretion system protein EccB |
| Rv3901c | Rv3901c | membrane protein |
| Rv3905c | esxF | ESAT-6 like protein EsxF |
| Rv3909 | Rv3909 | hypothetical protein |
| Rv3911 | sigM | ECF RNA polymerase sigma factor SigM |
| Rv3912 | Rv3912 | anti-sigma-M factor RsmA |
| Rv3915 | cwlM | peptidoglycan hydrolase |
| Rv3916c | Rv3916c | hypothetical protein |
| Rv0005 | gyrB | DNA gyrase subunit B |
| Rv3917c | parB | chromosome partitioning protein ParB |
| Rv3918c | parA | chromosome partitioning protein ParA |
| Rv3919c | gid | 16S rRNA (guanine(527)-N(7))-methyltransferase RsmG |
| Rv3920c | Rv3920c | hypothetical protein |
| Rv3921c | Rv3921c | membrane protein insertase YidC |
| Rv3922c | Rv3922c | membrane protein insertion efficiency factor |
| Rv3923c | rnpA | ribonuclease P protein component |
| Rv3924c | rpmH | 50S ribosomal protein L34 |
| Rv0391 | metZ | O-succinylhomoserine sulfhydrylase |
| Rv0395 | Rv0395 | hypothetical protein |
| Rv0397A | Rv0397A | hypothetical protein |
| Rv0040c | mtc28 | hypothetical protein |
| Rv0402c | mmpL1 | transmembrane transport protein MmpL1 |
| Rv0410c | pknG | serine/threonine-protein kinase PknG |
| Rv0412c | Rv0412c | membrane protein |
| Rv0042c | Rv0042c | transcriptional regulator |
| Rv0418 | lpqL | lipoprotein aminopeptidase LpqL |
| Rv0419 | lpqM | lipoprotein peptidase LpqM |
| Rv0420c | Rv0420c | transmembrane protein |
| Rv0422c | thiD | hydroxymethylpyrimidine/phosphomethylpyrimidine kinase |
| Rv0424c | Rv0424c | hypothetical protein |
| Rv0430 | Rv0430 | hypothetical protein |
| Rv0433 | Rv0433 | carboxylate-amine ligase |
| Rv0436c | pssA | CDP-diacylglycerol--serine O-phosphatidyltransferase |
| Rv0437c | psd | phosphatidylserine decarboxylase |
| Rv0440 | groEL2 | molecular chaperone GroEL |
| Rv0441c | Rv0441c | hypothetical protein |
| Rv0443 | Rv0443 | hypothetical protein |
| Rv0446c | Rv0446c | transmembrane protein |
| Rv0449c | Rv0449c | hypothetical protein |
| Rv0450c | mmpL4 | transmembrane transport protein MmpL4 |
| Rv0451c | mmpS4 | membrane protein MmpS4 |
| Rv0452 | Rv0452 | transcriptional regulator |
| Rv0455c | Rv0455c | hypothetical protein |
| Rv0046c | ino1 | inositol-3-phosphate synthase |
| Rv0462 | lpdC | dihydrolipoamide dehydrogenase |
| Rv0463 | Rv0463 | membrane protein |
| Rv0464c | Rv0464c | hypothetical protein |
| Rv0466 | Rv0466 | hypothetical protein |
| Rv0047c | Rv0047c | hypothetical protein |
| Rv0468 | fadB2 | 3-hydroxybutyryl-CoA dehydrogenase |
| Rv0469 | umaA | mycolic acid synthase UmaA |
| Rv0470c | pcaA | cyclopropane mycolic acid synthase |
| Rv0472c | Rv0472c | HTH-type transcriptional regulator |
| Rv0474 | Rv0474 | HTH-type transcriptional regulator |
| Rv0048c | Rv0048c | membrane protein |
| Rv0476 | Rv0476 | transmembrane protein |
| Rv0477 | Rv0477 | hypothetical protein |
| Rv0479c | Rv0479c | membrane protein |
| Rv0483 | lprQ | lipoprotein LprQ |
| Rv0485 | Rv0485 | transcriptional regulator |
| Rv0006 | gyrA | DNA gyrase subunit A |
| Rv0487 | Rv0487 | hypothetical protein |
| Rv0491 | regX3 | two component sensory transduction protein RegX |
| Rv0492A | Rv0492A | hypothetical protein |
| Rv0495c | Rv0495c | hypothetical protein |
| Rv0497 | Rv0497 | transmembrane protein |
| Rv0500 | proC | pyrroline-5-carboxylate reductase |
| Rv0500A | Rv0500A | DNA-binding protein |
| Rv0501 | galE2 | UDP-glucose 4-epimerase GalE |
| Rv0502 | Rv0502 | hypothetical protein |
| Rv0503c | cmaA2 | cyclopropane mycolic acid synthase |
| Rv0506 | mmpS2 | membrane protein MmpS2 |
| Rv0509 | hemA | glutamyl-tRNA reductase |
| Rv0511 | hemD | uroporphyrin-III C-methyltransferase |
| Rv0513 | Rv0513 | transmembrane protein |
| Rv0515 | Rv0515 | hypothetical protein |
| Rv0518 | Rv0518 | hypothetical protein |
| Rv0519c | Rv0519c | membrane protein |
| Rv0522 | gabP | GABA permease GabP |
| Rv0530A | Rv0530A | hypothetical protein |
| Rv0532 | PE_PGRS6 | PE-PGRS family protein PE_PGRS6 |
| Rv0054 | ssb | single-strand DNA-binding protein |
| Rv0535 | Rv0535 | 5'-methylthioadenosine phosphorylase |
| Rv0540 | Rv0540 | hypothetical protein |
| Rv0545c | pitA | low-affinity inorganic phosphate transporter |
| Rv0546c | Rv0546c | hypothetical protein |
| Rv0553 | menC | muconate cycloisomerase |
| Rv0056 | rplI | 50S ribosomal protein L9 |
| Rv0557 | mgtA | GDP-mannose-dependent alpha-mannosyltransferase |
| Rv0558 | menH | demethylmenaquinone methyltransferase |
| Rv0559c | Rv0559c | hypothetical protein |
| Rv0562 | grcC1 | polyprenyl-diphosphate synthase GrcC |
| Rv0563 | htpX | protease HtpX |
| Rv0057 | Rv0057 | hypothetical protein |
| Rv0568 | cyp135B1 | cytochrome P450 Cyp135B1 |
| Rv0570 | nrdZ | vitamin B12-dependent ribonucleoside-diphosphate reductase |
| Rv0058 | dnaB | replicative DNA helicase |
| Rv0579 | Rv0579 | hypothetical protein |
| Rv0581 | vapB26 | antitoxin VapB26 |
| Rv0059 | Rv0059 | hypothetical protein |
| Rv0586 | mce2R | HTH-type transcriptional regulator Mce2R |
| Rv0589 | mce2A | Mce family protein Mce2A |
| Rv0594 | mce2F | Mce family protein Mce2F |
| Rv0060 | Rv0060 | hypothetical protein |
| Rv0596c | vapB4 | antitoxin VapB4 |
| Rv0597c | Rv0597c | hypothetical protein |
| Rv0598c | vapC27 | ribonuclease VapC27 |
| Rv0599c | vapB27 | antitoxin VapB27 |
| Rv0600c | Rv0600c | two component sensor kinase HK1 |
| Rv0601c | Rv0601c | two component sensor kinase HK2 |
| Rv0602c | tcrA | two component DNA binding transcriptional regulator TcrA |
| Rv0603 | Rv0603 | hypothetical protein |
| Rv0061c | Rv0061c | hypothetical protein |
| Rv0608 | vapB28 | antitoxin VapB28 |
| Rv0617 | vapC29 | ribonuclease VapC29 |
| Rv0618 | galT | - |
| Rv0620 | galK | galactokinase |
| Rv0624 | vapC30 | ribonuclease VapC30 |
| Rv0063 | Rv0063 | oxidoreductase |
| Rv0625c | Rv0625c | transmembrane protein |
| Rv0626 | vapB5 | antitoxin VapB5 |
| Rv0631c | recC | exonuclease V subunit gamma RecC |
| Rv0632c | echA3 | enoyl-CoA hydratase EchA3 |
| Rv0633c | Rv0633c | hypothetical protein |
| Rv0634c | Rv0634c | glyoxalase II |
| Rv0063a | Rv0063a | hypothetical protein |
| Rv0634A | Rv0634A | hypothetical protein |
| Rvnt06 | thrT | 0 |
| Rvnt07 | metT | 0 |
| Rv0634B | rpmG2 | 50S ribosomal protein L33 |
| Rv0635 | hadA | (3R)-hydroxyacyl-ACP dehydratase subunit HadA |
| Rv0636 | hadB | (3R)-hydroxyacyl-ACP dehydratase subunit HadB |
| Rv0637 | hadC | (3R)-hydroxyacyl-ACP dehydratase subunit HadC |
| Rv0639 | nusG | transcription termination/antitermination protein NusG |
| Rv0064 | Rv0064 | transmembrane protein |
| Rv0640 | rplK | 50S ribosomal protein L11 |
| Rv0641 | rplA | 50S ribosomal protein L1 |
| Rv0642c | mmaA4 | hydroxymycolate synthase MmaA4 |
| Rv0643c | mmaA3 | methoxy mycolic acid synthase MmaA3 |
| Rv0648 | Rv0648 | alpha-mannosidase |
| Rv0649 | fabD2 | malonyl CoA-acyl carrier protein transacylase |
| Rv0064A | vapB1 | antitoxin VapB1 |
| Rv0650 | Rv0650 | sugar kinase |
| Rv0651 | rplJ | 50S ribosomal protein L10 |
| Rv0652 | rplL | 50S ribosomal protein L7/L12 |
| Rv0653c | Rv0653c | transcriptional regulator |
| Rv0654 | Rv0654 | carotenoid cleavage oxygenase |
| Rv0655 | mkl | ABC transporter ATP-binding protein |
| Rv0658c | Rv0658c | integral membrane protein |
| Rv0659c | mazF2 | toxin MazF2 |
| Rv0065 | vapC1 | ribonuclease VapC1 |
| Rv0660c | mazE2 | antitoxin MazE2 |
| Rv0661c | vapC7 | ribonuclease VapC7 |
| Rv0662c | vapB7 | antitoxin VapB7 |
| Rv0665 | vapC8 | ribonuclease VapC8 |
| Rv0666 | Rv0666 | membrane protein |
| Rv0667 | rpoB | DNA-directed RNA polymerase subunit beta |
| Rv0668 | rpoC | DNA-directed RNA polymerase subunit beta' |
| Rv0674 | Rv0674 | hypothetical protein |
| Rv0676c | mmpL5 | transmembrane transport protein MmpL5 |
| Rv0677c | mmpS5 | membrane protein MmpS5 |
| Rv0678 | Rv0678 | hypothetical protein |
| Rv0679c | Rv0679c | hypothetical protein |
| Rvnt01 | ileT | 0 |
| Rv0682 | rpsL | 30S ribosomal protein S12 |
| Rv0683 | rpsG | 30S ribosomal protein S7 |
| Rv0684 | fusA1 | elongation factor G |
| Rv0685 | tuf | elongation factor Tu |
| Rv0686 | Rv0686 | membrane protein |
| Rv0068 | Rv0068 | oxidoreductase |
| Rv0691c | Rv0691c | mycofactocin biosynthesis transcriptional regulator MftR |
| Rv0691A | Rv0691A | mycofactocin precursor |
| Rv0692 | Rv0692 | mycofactocin system protein MftB |
| Rv0694 | Rv0694 | mycofactocin system heme/flavin oxidoreductase MftD |
| Rv0699 | Rv0699 | hypothetical protein |
| Rv0700 | rpsJ | 30S ribosomal protein S10 |
| Rv0701 | rplC | 50S ribosomal protein L3 |
| Rv0702 | rplD | 50S ribosomal protein L4 |
| Rv0703 | rplW | 50S ribosomal protein L23 |
| Rv0704 | rplB | 50S ribosomal protein L2 |
| Rv0705 | rpsS | 30S ribosomal protein S19 |
| Rv0706 | rplV | 50S ribosomal protein L22 |
| Rv0707 | rpsC | 30S ribosomal protein S3 |
| Rv0708 | rplP | 50S ribosomal protein L16 |
| Rv0709 | rpmC | 50S ribosomal protein L29 |
| Rv0710 | rpsQ | 30S ribosomal protein S17 |
| Rv0711 | atsA | arylsulfatase AtsA |
| Rv0712 | Rv0712 | hypothetical protein |
| Rv0713 | Rv0713 | transmembrane protein |
| Rv0715 | rplX | 50S ribosomal protein L24 |
| Rv0716 | rplE | 50S ribosomal protein L5 |
| Rv0717 | rpsN1 | 30S ribosomal protein S14 |
| Rv0718 | rpsH | 30S ribosomal protein S8 |
| Rv0071 | Rv0071 | maturase |
| Rv0719 | rplF | 50S ribosomal protein L6 |
| Rv0721 | rpsE | 30S ribosomal protein S5 |
| Rv0722 | rpmD | 50S ribosomal protein L30 |
| Rv0724 | sppA | protease IV SppA |
| Rv0726c | Rv0726c | S-adenosylmethionine-dependent methyltransferase |
| Rv0728c | serA2 | D-3-phosphoglycerate dehydrogenase SerA |
| Rv0729 | xylB | D-xylulose kinase XylB |
| Rv0731c | Rv0731c | S-adenosylmethionine-dependent methyltransferase |
| Rv0732 | secY | preprotein translocase SecY |
| Rv0738 | Rv0738 | hypothetical protein |
| Rv0073 | Rv0073 | glutamine ABC transporter ATP-binding protein |
| Rv0745 | Rv0745 | hypothetical protein |
| Rv0746 | PE_PGRS9 | PE-PGRS family protein PE_PGRS9 |
| Rv0748 | vapB31 | antitoxin VapB31 |
| Rv0754 | PE_PGRS11 | PE-PGRS family protein PE_PGRS11 |
| Rv0755c | PPE12 | PPE family protein PPE12 |
| Rv0757 | phoP | two component system response transcriptional positive regulator PhoP |
| Rv0075 | Rv0075 | aminotransferase |
| Rv0759c | Rv0759c | hypothetical protein |
| Rv0760c | Rv0760c | hypothetical protein |
| Rv0761c | adhB | alcohol dehydrogenase B |
| Rv0076c | Rv0076c | membrane protein |
| Rv0774c | Rv0774c | hypothetical protein |
| Rv0777 | purB | adenylosuccinate lyase PurB |
| Rvnt02 | alaT | 0 |
| Rv0779c | Rv0779c | transmembrane protein |
| Rv0786c | Rv0786c | hypothetical protein |
| Rv0787 | Rv0787 | hypothetical protein |
| Rv0787A | Rv0787A | hypothetical protein |
| Rv0788 | purQ | phosphoribosylformylglycinamidine synthase |
| Rv0790c | Rv0790c | hypothetical protein |
| Rv0793 | Rv0793 | monooxygenase |
| Rv0797 | Rv0797 | insertion sequence element IS1547 transposase |
| Rv0799c | Rv0799c | hypothetical protein |
| Rv0800 | pepC | M18 family aminopeptidase |
| Rv0078A | Rv0078A | hypothetical protein |
| Rv0805 | Rv0805 | 3'%2C5'-cyclic adenosine monophosphate phosphodiesterase CpdA |
| Rv0806c | cpsY | exopolysaccharide phosphotransferase CpsY |
| Rv0807 | Rv0807 | hypothetical protein |
| Rv0809 | purM | phosphoribosylformylglycinamidine cyclo-ligase PurM |
| Rv0078B | Rv0078B | hypothetical protein |
| Rv0812 | pabC | 4-amino-4-deoxychorismate lyase |
| Rv0818 | Rv0818 | transcriptional regulator |
| Rv0819 | mshD | mycothiol acetyltransferase |
| Rv0820 | phoT | phosphate ABC transporter ATP-binding protein PhoT |
| Rv0821c | phoY2 | phosphate-transport system transcriptional regulator PhoY2 |
| Rv0823c | Rv0823c | tRNA-dihydrouridine synthase |
| Rv0824c | desA1 | acyl-ACP desaturase DesA |
| Rv0830 | Rv0830 | S-adenosylmethionine-dependent methyltransferase |
| Rv0831c | Rv0831c | hypothetical protein |
| Rvnt12 | aspT | 0 |
| Rv0832 | PE_PGRS12 | PE-PGRS family protein PE_PGRS12 |
| Rv0833 | PE_PGRS13 | PE-PGRS family protein PE_PGRS13 |
| Rv0838 | lpqR | lipoprotein LpqR |
| Rv0839 | Rv0839 | hypothetical protein |
| Rv0840c | pip | proline iminopeptidase |
| Rv0841 | Rv0841 | transmembrane protein |
| Rv0082 | Rv0082 | oxidoreductase |
| Rv0848 | cysK2 | cysteine synthase CysK |
| Rv0856 | Rv0856 | hypothetical protein |
| Rv0857 | Rv0857 | hypothetical protein |
| Rv0083 | Rv0083 | oxidoreductase |
| Rv0860 | fadB | fatty oxidation protein FadB |
| Rv0865 | mog | molybdopterin biosynthesis protein |
| Rv0870c | Rv0870c | integral membrane protein |
| Rv0873 | fadE10 | acyl-CoA dehydrogenase |
| Rv0875c | Rv0875c | hypothetical protein |
| Rv0877 | Rv0877 | hypothetical protein |
| Rv0085 | hycP | hydrogenase HycP |
| Rv0880 | Rv0880 | HTH-type transcriptional regulator |
| Rv0882 | Rv0882 | transmembrane protein |
| Rv0885 | Rv0885 | hypothetical protein |
| Rv0888 | Rv0888 | hypothetical protein |
| Rv0894 | Rv0894 | transcriptional regulator |
| Rv0896 | gltA2 | citrate synthase 1 |
| Rv0904c | accD3 | acetyl-CoAcarboxylase carboxyl transferase subunit beta |
| Rv0909 | Rv0909 | antitoxin |
| Rv0910 | Rv0910 | toxin |
| Rv0911 | Rv0911 | hypothetical protein |
| Rv0915c | PPE14 | PPE family protein PPE14 |
| Rv0916c | PE7 | PE family protein PE7 |
| Rv0918 | Rv0918 | hypothetical protein |
| Rv0919 | Rv0919 | GCN5-like N-acetyltransferase |
| Rvnt14 | argT | 0 |
| Rv0922 | Rv0922 | transposase |
| Rv0925c | Rv0925c | hypothetical protein |
| Rv0090 | Rv0090 | membrane protein |
| Rv0927c | Rv0927c | oxidoreductase |
| Rv0930 | pstA1 | phosphate ABC transporter permease PstA |
| Rv0931c | pknD | serinethreonine-protein kinase PknD |
| Rv0932c | pstS2 | phosphate ABC transporter substrate-binding lipoprotein PstS |
| Rv0933 | pstB | phosphate ABC transporter ATP-binding protein PstB |
| Rv0935 | pstC1 | phosphate ABC transporter permease PstC |
| Rv0936 | pstA2 | phosphate ABC transporter permease PstA |
| Rv0937c | mku | non-homologous end joining protein Ku |
| Rv0940c | Rv0940c | oxidoreductase |
| Rv0942 | Rv0942 | hypothetical protein |
| Rv0943c | Rv0943c | monooxygenase |
| Rv0951 | sucC | succinyl-CoA ligase subunit beta |
| Rv0962c | lprP | lipoprotein LprP |
| Rv0964c | Rv0964c | hypothetical protein |
| Rv0965c | Rv0965c | hypothetical protein |
| Rv0967 | csoR | copper-sensing transcriptional repressor CsoR |
| Rv0970 | Rv0970 | integral membrane protein |

Supplementary Table 3. Non-overlapping genes from HZRE blood cultures and week1-2-sputum relative to broth share similar GeneOntology molecular pathways

| Gene Ontology (GO): Molecular | HZRE_BLOOD | SPUTUM_week1&2 |
| --- | --- | --- |
| catalytic activity (GO:0003824) | 71% | 70% |
| binding (GO:0005488) | 16% | 17% |
| transporter activity (GO:0005215) | 3% | 9% |
| structural molecule activity (GO:0005198) | 5% | 2% |
| transcription regulator activity (GO:0140110) | 5% | 2% |
| molecular transducer activity (GO:0060089) | 1% | 1% |
| translation regulator activity (GO:0045182) | 0% | 1% |

Supplementary Table 4. List of overall drug-specific genes for all six individual-drug blood cultures.

| Drug | Locus tag | Gene symbol | Gene description |
| --- | --- | --- | --- |
| ethambutol | Rv0912 | Rv0912 | cell wall and cell processes |
| ethambutol | Rv3171c | hpx | virulence, detoxification, adaptation |
| ethambutol | Rv3784 | epiB | intermediary metabolism and respiration |
| ethambutol | Rv1567c | Rv1567c | cell wall and cell processes |
| ethambutol | Rv3100c | smpB | virulence, detoxification, adaptation |
| ethambutol | Rv0536 | galE3 | intermediary metabolism and respiration |
| ethambutol | Rv2631 | Rv2631 | conserved hypotheticals |
| ethambutol | Rv2395 | Rv2395 | cell wall and cell processes |
| ethambutol | Rv1289 | Rv1289 | conserved hypotheticals |
| ethambutol | Rv2911 | dacB2 | cell wall and cell processes |
| ethambutol | Rv0574c | Rv0574c | conserved hypotheticals |
| ethambutol | Rv0903c | prrA | regulatory proteins |
| ethambutol | Rv1010 | ksgA | information pathways |
| ethambutol | Rv1177 | fdxC | intermediary metabolism and respiration |
| ethambutol | Rv1474c | Rv1474c | regulatory proteins |
| ethambutol | Rv1961 | Rv1961 | conserved hypotheticals |
| ethambutol | Rv0513 | Rv0513 | cell wall and cell processes |
| ethambutol | Rv2831 | echA16 | lipid metabolism |
| ethambutol | Rv0089 | Rv0089 | intermediary metabolism and respiration |
| ethambutol | Rv0804 | Rv0804 | conserved hypotheticals |
| ethambutol | Rv3447c | eccC4 | cell wall and cell processes |
| ethambutol | Rv2408 | PE24 | PE/PPE |
| ethambutol | Rv2438A | Rv2438A | conserved hypotheticals |
| ethambutol | Rv3889c | espG2 | cell wall and cell processes |
| ethambutol | Rv1699 | pyrG | intermediary metabolism and respiration |
| ethambutol | Rv2564 | glnQ | cell wall and cell processes |
| ethambutol | Rv2640c | Rv2640c | regulatory proteins |
| ethambutol | Rv0478 | deoC | intermediary metabolism and respiration |
| ethambutol | Rv1226c | Rv1226c | cell wall and cell processes |
| ethambutol | Rv3125c | PPE49 | PE/PPE |
| ethambutol | Rv1368 | lprF | cell wall and cell processes |
| ethambutol | Rv1362c | Rv1362c | cell wall and cell processes |
| ethambutol | Rv0132c | fgd2 | intermediary metabolism and respiration |
| ethambutol | Rv2273 | Rv2273 | cell wall and cell processes |
| ethambutol | Rv2833c | ugpB | cell wall and cell processes |
| ethambutol | Rv0872c | PE_PGRS15 | PE/PPE |
| ethambutol | Rv3528c | Rv3528c | conserved hypotheticals |
| ethambutol | Rv3757c | proW | virulence, detoxification, adaptation |
| ethambutol | Rv2077A | Rv2077A | conserved hypotheticals |
| ethambutol | Rv2488c | Rv2488c | regulatory proteins |
| ethambutol | Rv2309A | Rv2309A | conserved hypotheticals |
| ethambutol | Rv2400c | subI | cell wall and cell processes |
| ethambutol | Rv3580c | cysS1 | information pathways |
| ethambutol | Rv3591c | Rv3591c | intermediary metabolism and respiration |
| ethambutol | Rv3621c | PPE65 | PE/PPE |
| ethambutol | Rv2451 | Rv2451 | conserved hypotheticals |
| ethambutol | Rv0186A | mymT | virulence, detoxification, adaptation |
| ethambutol | Rv2656c | Rv2656c | insertion seqs and phages |
| ethambutol | Rv0340 | Rv0340 | conserved hypotheticals |
| ethambutol | Rv1048c | Rv1048c | conserved hypotheticals |
| ethambutol | Rv0090 | Rv0090 | cell wall and cell processes |
| ethambutol | Rv0271c | fadE6 | lipid metabolism |
| ethambutol | Rv3828c | Rv3828c | insertion seqs and phages |
| ethambutol | Rv2360c | Rv2360c | conserved hypotheticals |
| ethambutol | Rv3123 | Rv3123 | conserved hypotheticals |
| ethambutol | Rv0434 | Rv0434 | conserved hypotheticals |
| ethambutol | Rv1531 | Rv1531 | conserved hypotheticals |
| ethambutol | Rv3707c | Rv3707c | conserved hypotheticals |
| ethambutol | Rv3293 | pcd | intermediary metabolism and respiration |
| ethambutol | Rv0456B | mazE1 | virulence, detoxification, adaptation |
| ethambutol | Rv1019 | Rv1019 | regulatory proteins |
| ethambutol | Rv3331 | sugI | cell wall and cell processes |
| ethambutol | Rv1963c | mce3R | regulatory proteins |
| ethambutol | Rv1653 | argJ | intermediary metabolism and respiration |
| ethambutol | Rv2744c | 35kd_ag | conserved hypotheticals |
| ethambutol | Rv0832 | PE_PGRS12 | PE/PPE |
| ethambutol | Rv2723 | Rv2723 | cell wall and cell processes |
| ethambutol | Rv0862c | Rv0862c | conserved hypotheticals |
| ethambutol | Rv2019 | Rv2019 | conserved hypotheticals |
| ethambutol | Rv1655 | argD | intermediary metabolism and respiration |
| ethambutol | Rv0160c | PE4 | PE/PPE |
| ethambutol | Rv1103c | mazE3 | virulence, detoxification, adaptation |
| ethambutol | Rv0726c | Rv0726c | lipid metabolism |
| ethambutol | Rv0393 | Rv0393 | insertion seqs and phages |
| ethambutol | Rv1574 | Rv1574 | insertion seqs and phages |
| ethambutol | Rv1245c | Rv1245c | intermediary metabolism and respiration |
| ethambutol | Rv2394 | ggtB | intermediary metabolism and respiration |
| ethambutol | Rv0427c | xthA | information pathways |
| ethambutol | Rv1067c | PE_PGRS19 | PE/PPE |
| ethambutol | Rv0609A | Rv0609A | conserved hypotheticals |
| ethambutol | Rv3071 | Rv3071 | conserved hypotheticals |
| ethambutol | Rv1070c | echA8 | lipid metabolism |
| ethambutol | Rv1536 | ileS | information pathways |
| ethambutol | Rv2798c | Rv2798c | conserved hypotheticals |
| ethambutol | Rvnt29 | hisT | stable RNAs |
| ethambutol | Rv0839 | Rv0839 | conserved hypotheticals |
| ethambutol | Rv3224 | Rv3224 | intermediary metabolism and respiration |
| ethambutol | Rv2259 | mscR | intermediary metabolism and respiration |
| ethambutol | Rv1975 | Rv1975 | conserved hypotheticals |
| ethambutol | Rv1150 | Rv1150 | insertion seqs and phages |
| ethambutol | Rv1179c | Rv1179c | conserved hypotheticals |
| ethambutol | Rv3905c | esxF | cell wall and cell processes |
| ethambutol | Rv0043c | Rv0043c | regulatory proteins |
| ethambutol | Rv0651 | rplJ | information pathways |
| ethambutol | Rv2227 | Rv2227 | conserved hypotheticals |
| ethambutol | Rv2369c | Rv2369c | conserved hypotheticals |
| ethambutol | Rv3593 | lpqF | cell wall and cell processes |
| ethambutol | Rv1912c | fadB5 | lipid metabolism |
| ethambutol | Rv3147 | nuoC | intermediary metabolism and respiration |
| ethambutol | Rv1248c | Rv1248c | intermediary metabolism and respiration |
| ethambutol | Rv1318c | Rv1318c | intermediary metabolism and respiration |
| ethambutol | Rv0395 | Rv0395 | conserved hypotheticals |
| ethambutol | Rv2257c | Rv2257c | conserved hypotheticals |
| ethambutol | Rv0733 | adk | intermediary metabolism and respiration |
| ethambutol | Rv3560c | fadE30 | lipid metabolism |
| ethambutol | Rv2916c | ffh | cell wall and cell processes |
| ethambutol | Rv2708c | Rv2708c | conserved hypotheticals |
| ethambutol | Rv2958c | Rv2958c | intermediary metabolism and respiration |
| ethambutol | Rv2160c | Rv2160c | regulatory proteins |
| ethambutol | Rv0356c | Rv0356c | conserved hypotheticals |
| ethambutol | Rv1967 | mce3B | virulence, detoxification, adaptation |
| ethambutol | Rv1628c | Rv1628c | conserved hypotheticals |
| ethambutol | Rv3365c | Rv3365c | conserved hypotheticals |
| ethambutol | Rv0453 | PPE11 | PE/PPE |
| ethambutol | Rv1319c | Rv1319c | intermediary metabolism and respiration |
| ethambutol | Rv2489c | Rv2489c | conserved hypotheticals |
| ethambutol | Rv3107c | agpS | lipid metabolism |
| ethambutol | Rv1534 | Rv1534 | regulatory proteins |
| ethambutol | Rv1497 | lipL | intermediary metabolism and respiration |
| ethambutol | Rv3235 | Rv3235 | conserved hypotheticals |
| ethambutol | Rv2917 | Rv2917 | conserved hypotheticals |
| ethambutol | Rv2305 | Rv2305 | conserved hypotheticals |
| ethambutol | Rv3532 | PPE61 | PE/PPE |
| ethambutol | Rv0775 | Rv0775 | conserved hypotheticals |
| ethambutol | Rv0691c | Rv0691c | regulatory proteins |
| ethambutol | Rv0834c | PE_PGRS14 | PE/PPE |
| ethambutol | Rv0157 | pntB | intermediary metabolism and respiration |
| ethambutol | Rv0127 | mak | virulence, detoxification, adaptation |
| ethambutol | Rv1110 | lytB2 | cell wall and cell processes |
| ethambutol | Rv1142c | echA10 | lipid metabolism |
| ethambutol | Rv2339 | mmpL9 | cell wall and cell processes |
| ethambutol | Rv2843 | Rv2843 | cell wall and cell processes |
| ethambutol | Rv0955 | Rv0955 | cell wall and cell processes |
| ethambutol | Rv2976c | ung | information pathways |
| ethambutol | Rv0196 | Rv0196 | regulatory proteins |
| ethambutol | Rv1221 | sigE | information pathways |
| ethambutol | Rv0742 | Rv0742 | PE/PPE |
| ethambutol | Rv3270 | ctpC | cell wall and cell processes |
| ethambutol | Rv0155 | pntAa | intermediary metabolism and respiration |
| ethambutol | Rv0262c | aac | virulence, detoxification, adaptation |
| ethambutol | Rv0514 | Rv0514 | cell wall and cell processes |
| ethambutol | Rv0431 | Rv0431 | cell wall and cell processes |
| ethambutol | Rv3887c | eccD2 | cell wall and cell processes |
| ethambutol | Rv3750c | Rv3750c | insertion seqs and phages |
| ethambutol | Rv2672 | Rv2672 | intermediary metabolism and respiration |
| ethambutol | Rv1258c | Rv1258c | cell wall and cell processes |
| ethambutol | Rv3787c | Rv3787c | conserved hypotheticals |
| ethambutol | Rv2671 | ribD | intermediary metabolism and respiration |
| ethambutol | Rv2231c | cobC | intermediary metabolism and respiration |
| ethambutol | Rv0570 | nrdZ | information pathways |
| ethambutol | Rv1576c | Rv1576c | insertion seqs and phages |
| ethambutol | Rv3354 | Rv3354 | conserved hypotheticals |
| ethambutol | Rv0797 | Rv0797 | insertion seqs and phages |
| ethambutol | Rv2894c | xerC | insertion seqs and phages |
| ethambutol | Rv3603c | Rv3603c | conserved hypotheticals |
| ethambutol | Rv1972 | Rv1972 | cell wall and cell processes |
| ethambutol | Rv3891c | esxD | cell wall and cell processes |
| ethambutol | Rv0996 | Rv0996 | cell wall and cell processes |
| ethambutol | Rv0116c | ldtA | cell wall and cell processes |
| ethambutol | Rv3124 | moaR1 | regulatory proteins |
| ethambutol | Rv3638 | Rv3638 | insertion seqs and phages |
| ethambutol | Rv1787 | PPE25 | PE/PPE |
| ethambutol | Rv3818 | Rv3818 | conserved hypotheticals |
| ethambutol | Rv0595c | vapC4 | virulence, detoxification, adaptation |
| ethambutol | Rv1810 | Rv1810 | conserved hypotheticals |
| ethambutol | Rv2120c | Rv2120c | cell wall and cell processes |
| ethambutol | Rv2384 | mbtA | lipid metabolism |
| ethambutol | Rv2527 | vapC17 | virulence, detoxification, adaptation |
| ethambutol | Rv1777 | cyp144 | intermediary metabolism and respiration |
| ethambutol | Rv2331 | Rv2331 | conserved hypotheticals |
| ethambutol | Rv3298c | lpqC | cell wall and cell processes |
| ethambutol | Rv0596c | vapB4 | virulence, detoxification, adaptation |
| ethambutol | Rv3813c | Rv3813c | conserved hypotheticals |
| ethambutol | Rv2540c | aroF | intermediary metabolism and respiration |
| ethambutol | Rv2428 | ahpC | virulence, detoxification, adaptation |
| ethambutol | Rv3096 | Rv3096 | conserved hypotheticals |
| ethambutol | Rv3081 | Rv3081 | conserved hypotheticals |
| ethambutol | Rv2427A | oxyR' | virulence, detoxification, adaptation |
| ethambutol | Rv0433 | Rv0433 | conserved hypotheticals |
| ethambutol | Rv2065 | cobH | intermediary metabolism and respiration |
| ethambutol | Rv2176 | pknL | regulatory proteins |
| ethambutol | Rv3498c | mce4B | virulence, detoxification, adaptation |
| ethambutol | Rv1568 | bioA | intermediary metabolism and respiration |
| ethambutol | Rv3811 | csp | conserved hypotheticals |
| ethambutol | Rv2149c | yfiH | conserved hypotheticals |
| ethambutol | Rv1016c | lpqT | cell wall and cell processes |
| ethambutol | Rv1316c | ogt | information pathways |
| ethambutol | Rv1087 | PE_PGRS21 | PE/PPE |
| ethambutol | Rv2219 | Rv2219 | cell wall and cell processes |
| ethambutol | Rv1740 | vapB34 | virulence, detoxification, adaptation |
| ethambutol | Rv0828c | Rv0828c | intermediary metabolism and respiration |
| ethambutol | Rv1970 | lprM | cell wall and cell processes |
| ethambutol | Rv3455c | truA | information pathways |
| ethambutol | Rv0696 | Rv0696 | intermediary metabolism and respiration |
| ethambutol | Rv3706c | Rv3706c | conserved hypotheticals |
| ethambutol | Rv1003 | Rv1003 | conserved hypotheticals |
| ethambutol | Rv1486c | Rv1486c | conserved hypotheticals |
| ethambutol | Rv1422 | Rv1422 | conserved hypotheticals |
| ethambutol | Rv0326 | Rv0326 | unknown |
| ethambutol | Rv1887a | Rv1887a | NA |
| ethambutol | Rv1341 | Rv1341 | conserved hypotheticals |
| ethambutol | Rv2028c | Rv2028c | virulence, detoxification, adaptation |
| ethambutol | Rv3164c | moxR3 | regulatory proteins |
| ethambutol | Rv2997 | Rv2997 | intermediary metabolism and respiration |
| ethambutol | Rv1984c | cfp21 | cell wall and cell processes |
| ethambutol | Rv1560 | vapB11 | virulence, detoxification, adaptation |
| ethambutol | Rv0149 | Rv0149 | intermediary metabolism and respiration |
| ethambutol | Rv3073c | Rv3073c | conserved hypotheticals |
| ethambutol | Rv1591 | Rv1591 | cell wall and cell processes |
| ethambutol | Rv0387c | Rv0387c | conserved hypotheticals |
| ethambutol | Rv2923c | Rv2923c | conserved hypotheticals |
| ethambutol | Rv3231c | Rv3231c | conserved hypotheticals |
| ethambutol | Rv0540 | Rv0540 | conserved hypotheticals |
| ethambutol | Rv1654 | argB | intermediary metabolism and respiration |
| ethambutol | Rv3159c | PPE53 | PE/PPE |
| ethambutol | Rv3752c | Rv3752c | intermediary metabolism and respiration |
| ethambutol | Rv2703 | sigA | information pathways |
| ethambutol | Rv2314c | Rv2314c | conserved hypotheticals |
| ethambutol | Rv3857c | Rv3857c | cell wall and cell processes |
| ethambutol | Rv0396 | Rv0396 | conserved hypotheticals |
| ethambutol | Rv3077 | atsF | intermediary metabolism and respiration |
| ethambutol | Rv3748 | Rv3748 | conserved hypotheticals |
| ethambutol | Rv1210 | tagA | information pathways |
| ethambutol | Rv1686c | Rv1686c | cell wall and cell processes |
| ethambutol | Rv1024 | Rv1024 | cell wall and cell processes |
| ethambutol | Rv1262c | Rv1262c | conserved hypotheticals |
| ethambutol | Rv0600c | Rv0600c | regulatory proteins |
| ethambutol | Rvnt33 | cysU | stable RNAs |
| ethambutol | Rv1157c | Rv1157c | conserved hypotheticals |
| ethambutol | Rv3169 | Rv3169 | conserved hypotheticals |
| ethambutol | Rv2643 | arsC | cell wall and cell processes |
| ethambutol | Rv2682c | dxs1 | intermediary metabolism and respiration |
| ethambutol | Rv3194c | Rv3194c | cell wall and cell processes |
| ethambutol | Rv0233 | nrdB | information pathways |
| ethambutol | Rv3087 | Rv3087 | lipid metabolism |
| ethambutol | Rv1516c | Rv1516c | intermediary metabolism and respiration |
| ethambutol | Rv0107c | ctpI | cell wall and cell processes |
| ethambutol | Rv3014c | ligA | information pathways |
| ethambutol | Rv0763c | Rv0763c | intermediary metabolism and respiration |
| ethambutol | Rv2695 | Rv2695 | conserved hypotheticals |
| ethambutol | Rv2526 | vapB17 | virulence, detoxification, adaptation |
| ethambutol | Rv3668c | Rv3668c | intermediary metabolism and respiration |
| ethambutol | Rv2808 | Rv2808 | conserved hypotheticals |
| ethambutol | Rv0755c | PPE12 | PE/PPE |
| ethambutol | Rv3678c | Rv3678c | conserved hypotheticals |
| ethambutol | Rv1202 | dapE | intermediary metabolism and respiration |
| ethambutol | Rv3017c | esxQ | cell wall and cell processes |
| ethambutol | Rv2619c | Rv2619c | conserved hypotheticals |
| ethambutol | Rv1407 | fmu | information pathways |
| ethambutol | Rv1948c | Rv1948c | conserved hypotheticals |
| ethambutol | Rv1487 | Rv1487 | cell wall and cell processes |
| ethambutol | Rv1944c | Rv1944c | conserved hypotheticals |
| ethambutol | Rv0884c | serC | intermediary metabolism and respiration |
| ethambutol | Rv0826 | Rv0826 | conserved hypotheticals |
| ethambutol | Rv2735c | Rv2735c | conserved hypotheticals |
| ethambutol | Rv3039c | echA17 | lipid metabolism |
| ethambutol | Rv1261c | Rv1261c | conserved hypotheticals |
| faropenem | Rv3784 | epiB | intermediary metabolism and respiration |
| faropenem | Rv3031 | Rv3031 | conserved hypotheticals |
| faropenem | Rv1567c | Rv1567c | cell wall and cell processes |
| faropenem | Rv0754 | PE_PGRS11 | PE/PPE |
| faropenem | Rv2631 | Rv2631 | conserved hypotheticals |
| faropenem | Rv1289 | Rv1289 | conserved hypotheticals |
| faropenem | Rv2911 | dacB2 | cell wall and cell processes |
| faropenem | Rv3090 | Rv3090 | conserved hypotheticals |
| faropenem | Rv3353c | Rv3353c | conserved hypotheticals |
| faropenem | Rv1156 | Rv1156 | conserved hypotheticals |
| faropenem | Rv0903c | prrA | regulatory proteins |
| faropenem | Rv1010 | ksgA | information pathways |
| faropenem | Rv1177 | fdxC | intermediary metabolism and respiration |
| faropenem | Rv1376 | Rv1376 | conserved hypotheticals |
| faropenem | Rv1586c | Rv1586c | insertion seqs and phages |
| faropenem | Rv1961 | Rv1961 | conserved hypotheticals |
| faropenem | Rv0513 | Rv0513 | cell wall and cell processes |
| faropenem | Rv2253 | Rv2253 | cell wall and cell processes |
| faropenem | Rv2831 | echA16 | lipid metabolism |
| faropenem | Rv0804 | Rv0804 | conserved hypotheticals |
| faropenem | Rv2621c | Rv2621c | regulatory proteins |
| faropenem | Rv0468 | fadB2 | lipid metabolism |
| faropenem | Rv3447c | eccC4 | cell wall and cell processes |
| faropenem | Rv2408 | PE24 | PE/PPE |
| faropenem | Rv1284 | canA | intermediary metabolism and respiration |
| faropenem | Rv1545 | Rv1545 | conserved hypotheticals |
| faropenem | Rv2438A | Rv2438A | conserved hypotheticals |
| faropenem | Rv1861 | Rv1861 | cell wall and cell processes |
| faropenem | Rv1140 | Rv1140 | cell wall and cell processes |
| faropenem | Rv1647 | Rv1647 | intermediary metabolism and respiration |
| faropenem | Rv3521 | Rv3521 | conserved hypotheticals |
| faropenem | Rv1368 | lprF | cell wall and cell processes |
| faropenem | Rv2509 | Rv2509 | intermediary metabolism and respiration |
| faropenem | Rv2273 | Rv2273 | cell wall and cell processes |
| faropenem | Rv3528c | Rv3528c | conserved hypotheticals |
| faropenem | Rv2488c | Rv2488c | regulatory proteins |
| faropenem | Rv2309A | Rv2309A | conserved hypotheticals |
| faropenem | Rv0504c | Rv0504c | conserved hypotheticals |
| faropenem | Rv2400c | subI | cell wall and cell processes |
| faropenem | Rv2555c | alaS | information pathways |
| faropenem | Rv1015c | rplY | information pathways |
| faropenem | Rv3621c | PPE65 | PE/PPE |
| faropenem | Rv0186A | mymT | virulence, detoxification, adaptation |
| faropenem | Rv1344 | mbtL | lipid metabolism |
| faropenem | Rv0807 | Rv0807 | conserved hypotheticals |
| faropenem | Rv0510 | hemC | intermediary metabolism and respiration |
| faropenem | Rv1835c | Rv1835c | conserved hypotheticals |
| faropenem | Rv0090 | Rv0090 | cell wall and cell processes |
| faropenem | Rv3712 | Rv3712 | intermediary metabolism and respiration |
| faropenem | Rv3268 | Rv3268 | conserved hypotheticals |
| faropenem | Rv2528c | mrr | information pathways |
| faropenem | Rv1175c | fadH | lipid metabolism |
| faropenem | Rv2626c | hrp1 | conserved hypotheticals |
| faropenem | Rv0429c | def | information pathways |
| faropenem | Rv0434 | Rv0434 | conserved hypotheticals |
| faropenem | Rv1400c | lipI | intermediary metabolism and respiration |
| faropenem | Rv1531 | Rv1531 | conserved hypotheticals |
| faropenem | Rv0735 | sigL | information pathways |
| faropenem | Rv2925c | rnc | information pathways |
| faropenem | Rv1019 | Rv1019 | regulatory proteins |
| faropenem | Rv3331 | sugI | cell wall and cell processes |
| faropenem | Rv2744c | 35kd_ag | conserved hypotheticals |
| faropenem | Rv2357c | glyS | information pathways |
| faropenem | Rv1288 | Rv1288 | conserved hypotheticals |
| faropenem | Rv3243c | Rv3243c | conserved hypotheticals |
| faropenem | Rv0493c | Rv0493c | conserved hypotheticals |
| faropenem | Rv2416c | eis | virulence, detoxification, adaptation |
| faropenem | Rv1901 | cinA | virulence, detoxification, adaptation |
| faropenem | Rv1655 | argD | intermediary metabolism and respiration |
| faropenem | Rv1103c | mazE3 | virulence, detoxification, adaptation |
| faropenem | Rv0726c | Rv0726c | lipid metabolism |
| faropenem | Rv0393 | Rv0393 | insertion seqs and phages |
| faropenem | Rv0015c | pknA | regulatory proteins |
| faropenem | Rv1574 | Rv1574 | insertion seqs and phages |
| faropenem | Rv1245c | Rv1245c | intermediary metabolism and respiration |
| faropenem | Rv2394 | ggtB | intermediary metabolism and respiration |
| faropenem | Rv3401 | Rv3401 | intermediary metabolism and respiration |
| faropenem | Rv1067c | PE_PGRS19 | PE/PPE |
| faropenem | Rv0609A | Rv0609A | conserved hypotheticals |
| faropenem | Rv1070c | echA8 | lipid metabolism |
| faropenem | Rvnt29 | hisT | stable RNAs |
| faropenem | Rv0839 | Rv0839 | conserved hypotheticals |
| faropenem | Rv2110c | prcB | intermediary metabolism and respiration |
| faropenem | Rv2259 | mscR | intermediary metabolism and respiration |
| faropenem | Rv1930c | Rv1930c | conserved hypotheticals |
| faropenem | Rv3471c | Rv3471c | conserved hypotheticals |
| faropenem | Rv1762c | Rv1762c | conserved hypotheticals |
| faropenem | Rv1179c | Rv1179c | conserved hypotheticals |
| faropenem | Rv3905c | esxF | cell wall and cell processes |
| faropenem | Rv1373 | Rv1373 | intermediary metabolism and respiration |
| faropenem | Rv2436 | rbsK | intermediary metabolism and respiration |
| faropenem | Rv0043c | Rv0043c | regulatory proteins |
| faropenem | Rv1106c | Rv1106c | intermediary metabolism and respiration |
| faropenem | Rv0651 | rplJ | information pathways |
| faropenem | Rv2369c | Rv2369c | conserved hypotheticals |
| faropenem | Rv3593 | lpqF | cell wall and cell processes |
| faropenem | Rv1912c | fadB5 | lipid metabolism |
| faropenem | Rv2716 | Rv2716 | conserved hypotheticals |
| faropenem | Rv3147 | nuoC | intermediary metabolism and respiration |
| faropenem | Rv3204 | Rv3204 | information pathways |
| faropenem | Rv1318c | Rv1318c | intermediary metabolism and respiration |
| faropenem | Rv0395 | Rv0395 | conserved hypotheticals |
| faropenem | Rv2062c | cobN | intermediary metabolism and respiration |
| faropenem | Rv1900c | lipJ | intermediary metabolism and respiration |
| faropenem | Rv0081 | Rv0081 | regulatory proteins |
| faropenem | Rv3560c | fadE30 | lipid metabolism |
| faropenem | Rv2916c | ffh | cell wall and cell processes |
| faropenem | Rv2708c | Rv2708c | conserved hypotheticals |
| faropenem | Rv2958c | Rv2958c | intermediary metabolism and respiration |
| faropenem | Rv0356c | Rv0356c | conserved hypotheticals |
| faropenem | Rv1967 | mce3B | virulence, detoxification, adaptation |
| faropenem | Rv2030c | Rv2030c | conserved hypotheticals |
| faropenem | Rv1628c | Rv1628c | conserved hypotheticals |
| faropenem | Rv0453 | PPE11 | PE/PPE |
| faropenem | Rv2489c | Rv2489c | conserved hypotheticals |
| faropenem | Rv3290c | lat | intermediary metabolism and respiration |
| faropenem | Rv2127 | ansP1 | cell wall and cell processes |
| faropenem | Rv0373c | Rv0373c | intermediary metabolism and respiration |
| faropenem | Rv2687c | Rv2687c | cell wall and cell processes |
| faropenem | Rv0834c | PE_PGRS14 | PE/PPE |
| faropenem | Rv1532c | Rv1532c | conserved hypotheticals |
| faropenem | Rv1468c | PE_PGRS29 | PE/PPE |
| faropenem | Rv2646 | Rv2646 | insertion seqs and phages |
| faropenem | Rv1110 | lytB2 | cell wall and cell processes |
| faropenem | Rv3688c | Rv3688c | conserved hypotheticals |
| faropenem | Rv2843 | Rv2843 | cell wall and cell processes |
| faropenem | Rv0955 | Rv0955 | cell wall and cell processes |
| faropenem | Rv0196 | Rv0196 | regulatory proteins |
| faropenem | Rv0742 | Rv0742 | PE/PPE |
| faropenem | Rv3270 | ctpC | cell wall and cell processes |
| faropenem | Rv1557 | mmpL6 | cell wall and cell processes |
| faropenem | Rv2877c | merT | cell wall and cell processes |
| faropenem | Rv3573c | fadE34 | lipid metabolism |
| faropenem | Rv3399 | Rv3399 | lipid metabolism |
| faropenem | Rv0294 | tam | intermediary metabolism and respiration |
| faropenem | Rv1252c | lprE | cell wall and cell processes |
| faropenem | Rv3509c | ilvX | intermediary metabolism and respiration |
| faropenem | Rv3750c | Rv3750c | insertion seqs and phages |
| faropenem | Rv3787c | Rv3787c | conserved hypotheticals |
| faropenem | Rv2147c | Rv2147c | conserved hypotheticals |
| faropenem | Rv2671 | ribD | intermediary metabolism and respiration |
| faropenem | Rv2946c | pks1 | lipid metabolism |
| faropenem | Rv1148c | Rv1148c | insertion seqs and phages |
| faropenem | Rv0096 | PPE1 | PE/PPE |
| faropenem | Rv1576c | Rv1576c | insertion seqs and phages |
| faropenem | Rv0797 | Rv0797 | insertion seqs and phages |
| faropenem | Rv3603c | Rv3603c | conserved hypotheticals |
| faropenem | Rv0996 | Rv0996 | cell wall and cell processes |
| faropenem | Rv3504 | fadE26 | lipid metabolism |
| faropenem | Rv1160 | mutT2 | information pathways |
| faropenem | Rv3538 | hsd4B | intermediary metabolism and respiration |
| faropenem | Rv3124 | moaR1 | regulatory proteins |
| faropenem | Rv3444c | esxT | cell wall and cell processes |
| faropenem | Rv3638 | Rv3638 | insertion seqs and phages |
| faropenem | Rv3324c | moaC3 | intermediary metabolism and respiration |
| faropenem | Rv1787 | PPE25 | PE/PPE |
| faropenem | Rv3818 | Rv3818 | conserved hypotheticals |
| faropenem | Rvnt19 | leuW | stable RNAs |
| faropenem | Rv1367c | Rv1367c | conserved hypotheticals |
| faropenem | Rv2835c | ugpA | cell wall and cell processes |
| faropenem | Rv2120c | Rv2120c | cell wall and cell processes |
| faropenem | Rv2384 | mbtA | lipid metabolism |
| faropenem | Rv3501c | yrbE4A | virulence, detoxification, adaptation |
| faropenem | Rv0596c | vapB4 | virulence, detoxification, adaptation |
| faropenem | Rv3813c | Rv3813c | conserved hypotheticals |
| faropenem | Rv1007c | metS | information pathways |
| faropenem | Rv1049 | Rv1049 | regulatory proteins |
| faropenem | Rv2428 | ahpC | virulence, detoxification, adaptation |
| faropenem | Rv1075c | Rv1075c | cell wall and cell processes |
| faropenem | Rv3033 | Rv3033 | conserved hypotheticals |
| faropenem | Rv0433 | Rv0433 | conserved hypotheticals |
| faropenem | Rv2427A | oxyR' | virulence, detoxification, adaptation |
| faropenem | Rv3705A | Rv3705A | conserved hypotheticals |
| faropenem | Rv1568 | bioA | intermediary metabolism and respiration |
| faropenem | Rv3811 | csp | conserved hypotheticals |
| faropenem | Rv0133 | Rv0133 | intermediary metabolism and respiration |
| faropenem | Rv3153 | nuoI | intermediary metabolism and respiration |
| faropenem | Rv1316c | ogt | information pathways |
| faropenem | Rv3642c | Rv3642c | conserved hypotheticals |
| faropenem | Rv2076c | Rv2076c | conserved hypotheticals |
| faropenem | Rv2063A | mazF7 | virulence, detoxification, adaptation |
| faropenem | Rv1129c | Rv1129c | regulatory proteins |
| faropenem | Rvnt24 | metV | stable RNAs |
| faropenem | Rv1740 | vapB34 | virulence, detoxification, adaptation |
| faropenem | Rv1970 | lprM | cell wall and cell processes |
| faropenem | Rv2908c | Rv2908c | conserved hypotheticals |
| faropenem | Rv3649 | Rv3649 | information pathways |
| faropenem | Rv0560c | Rv0560c | intermediary metabolism and respiration |
| faropenem | Rv3234c | tgs3 | lipid metabolism |
| faropenem | Rv1422 | Rv1422 | conserved hypotheticals |
| faropenem | Rv3208A | TB9.4 | conserved hypotheticals |
| faropenem | Rv2028c | Rv2028c | virulence, detoxification, adaptation |
| faropenem | Rv1560 | vapB11 | virulence, detoxification, adaptation |
| faropenem | Rv3073c | Rv3073c | conserved hypotheticals |
| faropenem | Rv0387c | Rv0387c | conserved hypotheticals |
| faropenem | Rv3231c | Rv3231c | conserved hypotheticals |
| faropenem | Rv3202c | Rv3202c | information pathways |
| faropenem | Rv3758c | proV | virulence, detoxification, adaptation |
| faropenem | Rv1575 | Rv1575 | insertion seqs and phages |
| faropenem | Rv2314c | Rv2314c | conserved hypotheticals |
| faropenem | Rv3720 | Rv3720 | lipid metabolism |
| faropenem | Rv0661c | vapC7 | virulence, detoxification, adaptation |
| faropenem | Rv2490c | PE_PGRS43 | PE/PPE |
| faropenem | Rv1686c | Rv1686c | cell wall and cell processes |
| faropenem | Rv0437c | psd | lipid metabolism |
| faropenem | Rv1024 | Rv1024 | cell wall and cell processes |
| faropenem | Rv3548c | Rv3548c | intermediary metabolism and respiration |
| faropenem | Rv1262c | Rv1262c | conserved hypotheticals |
| faropenem | Rv0600c | Rv0600c | regulatory proteins |
| faropenem | Rv3862c | whiB6 | regulatory proteins |
| faropenem | Rvnt33 | cysU | stable RNAs |
| faropenem | Rv3476c | kgtP | cell wall and cell processes |
| faropenem | Rv3169 | Rv3169 | conserved hypotheticals |
| faropenem | Rv2643 | arsC | cell wall and cell processes |
| faropenem | Rv2682c | dxs1 | intermediary metabolism and respiration |
| faropenem | Rv0965c | Rv0965c | conserved hypotheticals |
| faropenem | Rv3087 | Rv3087 | lipid metabolism |
| faropenem | Rv1516c | Rv1516c | intermediary metabolism and respiration |
| faropenem | Rv2762c | Rv2762c | conserved hypotheticals |
| faropenem | Rv0573c | pncB2 | intermediary metabolism and respiration |
| faropenem | Rv2500c | fadE19 | lipid metabolism |
| faropenem | Rv3014c | ligA | information pathways |
| faropenem | Rv2695 | Rv2695 | conserved hypotheticals |
| faropenem | Rv3062 | ligB | information pathways |
| faropenem | Rv1850 | ureC | intermediary metabolism and respiration |
| faropenem | Rv2122c | hisE | intermediary metabolism and respiration |
| faropenem | Rv2808 | Rv2808 | conserved hypotheticals |
| faropenem | Rv0755c | PPE12 | PE/PPE |
| faropenem | Rvnt01 | ileT | stable RNAs |
| faropenem | Rv1948c | Rv1948c | conserved hypotheticals |
| faropenem | Rv1982A | vapB36 | virulence, detoxification, adaptation |
| faropenem | Rv1944c | Rv1944c | conserved hypotheticals |
| faropenem | Rv0826 | Rv0826 | conserved hypotheticals |
| faropenem | Rv1261c | Rv1261c | conserved hypotheticals |
| faropenem | Rv0197 | Rv0197 | intermediary metabolism and respiration |
| faropenem | Rv3561 | fadD3 | lipid metabolism |
| faropenem | Rv2234 | ptpA | regulatory proteins |
| faropenem | Rv2142c | parE2 | virulence, detoxification, adaptation |
| faropenem | Rv0851c | Rv0851c | intermediary metabolism and respiration |
| isoniazid | Rv2813 | Rv2813 | conserved hypotheticals |
| isoniazid | Rv1567c | Rv1567c | cell wall and cell processes |
| isoniazid | Rvnt34 | valU | stable RNAs |
| isoniazid | Rv2395 | Rv2395 | cell wall and cell processes |
| isoniazid | Rv1289 | Rv1289 | conserved hypotheticals |
| isoniazid | Rv0892 | Rv0892 | intermediary metabolism and respiration |
| isoniazid | Rv1432 | Rv1432 | intermediary metabolism and respiration |
| isoniazid | Rv1156 | Rv1156 | conserved hypotheticals |
| isoniazid | Rv0903c | prrA | regulatory proteins |
| isoniazid | Rv1010 | ksgA | information pathways |
| isoniazid | Rv1586c | Rv1586c | insertion seqs and phages |
| isoniazid | Rv0513 | Rv0513 | cell wall and cell processes |
| isoniazid | Rv2831 | echA16 | lipid metabolism |
| isoniazid | Rv0804 | Rv0804 | conserved hypotheticals |
| isoniazid | Rv2850c | Rv2850c | intermediary metabolism and respiration |
| isoniazid | Rv3447c | eccC4 | cell wall and cell processes |
| isoniazid | Rv1030 | kdpB | cell wall and cell processes |
| isoniazid | Rv2408 | PE24 | PE/PPE |
| isoniazid | Rv1861 | Rv1861 | cell wall and cell processes |
| isoniazid | Rv3889c | espG2 | cell wall and cell processes |
| isoniazid | Rv1699 | pyrG | intermediary metabolism and respiration |
| isoniazid | Rv2640c | Rv2640c | regulatory proteins |
| isoniazid | Rv3314c | deoA | intermediary metabolism and respiration |
| isoniazid | Rv0478 | deoC | intermediary metabolism and respiration |
| isoniazid | Rv2782c | pepR | intermediary metabolism and respiration |
| isoniazid | Rv3215 | entC | intermediary metabolism and respiration |
| isoniazid | Rv1448c | tal | intermediary metabolism and respiration |
| isoniazid | Rv1368 | lprF | cell wall and cell processes |
| isoniazid | Rv0132c | fgd2 | intermediary metabolism and respiration |
| isoniazid | Rv3528c | Rv3528c | conserved hypotheticals |
| isoniazid | Rv2209 | Rv2209 | cell wall and cell processes |
| isoniazid | Rv0302 | Rv0302 | regulatory proteins |
| isoniazid | Rv2589 | gabT | intermediary metabolism and respiration |
| isoniazid | Rv1781c | malQ | intermediary metabolism and respiration |
| isoniazid | Rv2263 | Rv2263 | intermediary metabolism and respiration |
| isoniazid | Rv2077A | Rv2077A | conserved hypotheticals |
| isoniazid | Rv2488c | Rv2488c | regulatory proteins |
| isoniazid | Rv2309A | Rv2309A | conserved hypotheticals |
| isoniazid | Rv0992c | Rv0992c | conserved hypotheticals |
| isoniazid | Rv2400c | subI | cell wall and cell processes |
| isoniazid | Rv3580c | cysS1 | information pathways |
| isoniazid | Rv0184 | Rv0184 | conserved hypotheticals |
| isoniazid | Rv3591c | Rv3591c | intermediary metabolism and respiration |
| isoniazid | Rv1634 | Rv1634 | cell wall and cell processes |
| isoniazid | Rv0186A | mymT | virulence, detoxification, adaptation |
| isoniazid | Rv1484 | inhA | lipid metabolism |
| isoniazid | Rv0340 | Rv0340 | conserved hypotheticals |
| isoniazid | Rv2092c | helY | information pathways |
| isoniazid | Rv1991c | mazF6 | virulence, detoxification, adaptation |
| isoniazid | Rv0778 | cyp126 | intermediary metabolism and respiration |
| isoniazid | Rv1839c | vapB13 | virulence, detoxification, adaptation |
| isoniazid | Rv0475 | hbhA | cell wall and cell processes |
| isoniazid | Rv1531 | Rv1531 | conserved hypotheticals |
| isoniazid | Rv3707c | Rv3707c | conserved hypotheticals |
| isoniazid | Rv0976c | Rv0976c | conserved hypotheticals |
| isoniazid | Rv2925c | rnc | information pathways |
| isoniazid | Rv0450c | mmpL4 | cell wall and cell processes |
| isoniazid | Rv0097 | Rv0097 | intermediary metabolism and respiration |
| isoniazid | Rv1653 | argJ | intermediary metabolism and respiration |
| isoniazid | Rv3502c | hsd4A | intermediary metabolism and respiration |
| isoniazid | Rv1934c | fadE17 | lipid metabolism |
| isoniazid | Rv2744c | 35kd_ag | conserved hypotheticals |
| isoniazid | Rv0832 | PE_PGRS12 | PE/PPE |
| isoniazid | Rv3422c | Rv3422c | conserved hypotheticals |
| isoniazid | Rv2019 | Rv2019 | conserved hypotheticals |
| isoniazid | Rv1655 | argD | intermediary metabolism and respiration |
| isoniazid | Rv3415c | Rv3415c | conserved hypotheticals |
| isoniazid | Rv0393 | Rv0393 | insertion seqs and phages |
| isoniazid | Rv1824 | Rv1824 | cell wall and cell processes |
| isoniazid | Rv0015c | pknA | regulatory proteins |
| isoniazid | Rv3468c | rmlB3 | intermediary metabolism and respiration |
| isoniazid | Rv1245c | Rv1245c | intermediary metabolism and respiration |
| isoniazid | Rv3401 | Rv3401 | intermediary metabolism and respiration |
| isoniazid | Rv1067c | PE_PGRS19 | PE/PPE |
| isoniazid | Rv0419 | lpqM | cell wall and cell processes |
| isoniazid | Rv2599 | Rv2599 | cell wall and cell processes |
| isoniazid | Rv1930c | Rv1930c | conserved hypotheticals |
| isoniazid | Rv2259 | mscR | intermediary metabolism and respiration |
| isoniazid | Rv1975 | Rv1975 | conserved hypotheticals |
| isoniazid | Rv2759c | vapC42 | virulence, detoxification, adaptation |
| isoniazid | Rv0863 | Rv0863 | conserved hypotheticals |
| isoniazid | Rv1506c | Rv1506c | unknown |
| isoniazid | Rv3905c | esxF | cell wall and cell processes |
| isoniazid | Rv0651 | rplJ | information pathways |
| isoniazid | Rv2227 | Rv2227 | conserved hypotheticals |
| isoniazid | Rv2369c | Rv2369c | conserved hypotheticals |
| isoniazid | Rv1912c | fadB5 | lipid metabolism |
| isoniazid | Rv2298 | Rv2298 | conserved hypotheticals |
| isoniazid | Rv3000 | Rv3000 | cell wall and cell processes |
| isoniazid | Rv0395 | Rv0395 | conserved hypotheticals |
| isoniazid | Rv1679 | fadE16 | lipid metabolism |
| isoniazid | Rv0081 | Rv0081 | regulatory proteins |
| isoniazid | Rv2257c | Rv2257c | conserved hypotheticals |
| isoniazid | Rv1858 | modB | cell wall and cell processes |
| isoniazid | Rv2916c | ffh | cell wall and cell processes |
| isoniazid | Rv2708c | Rv2708c | conserved hypotheticals |
| isoniazid | Rv0356c | Rv0356c | conserved hypotheticals |
| isoniazid | Rv1967 | mce3B | virulence, detoxification, adaptation |
| isoniazid | Rv2030c | Rv2030c | conserved hypotheticals |
| isoniazid | Rv1181 | pks4 | lipid metabolism |
| isoniazid | Rv1319c | Rv1319c | intermediary metabolism and respiration |
| isoniazid | Rv3107c | agpS | lipid metabolism |
| isoniazid | Rv2560 | Rv2560 | cell wall and cell processes |
| isoniazid | Rv2984 | ppk1 | intermediary metabolism and respiration |
| isoniazid | Rvnt27 | glyV | stable RNAs |
| isoniazid | Rv0691c | Rv0691c | regulatory proteins |
| isoniazid | Rv0834c | PE_PGRS14 | PE/PPE |
| isoniazid | Rv1468c | PE_PGRS29 | PE/PPE |
| isoniazid | Rv0841 | Rv0841 | cell wall and cell processes |
| isoniazid | Rv0157 | pntB | intermediary metabolism and respiration |
| isoniazid | Rv1110 | lytB2 | cell wall and cell processes |
| isoniazid | Rv3688c | Rv3688c | conserved hypotheticals |
| isoniazid | Rv2854 | Rv2854 | conserved hypotheticals |
| isoniazid | Rv1841c | Rv1841c | cell wall and cell processes |
| isoniazid | Rv0955 | Rv0955 | cell wall and cell processes |
| isoniazid | Rv3139 | fadE24 | lipid metabolism |
| isoniazid | Rv0196 | Rv0196 | regulatory proteins |
| isoniazid | Rv2432c | Rv2432c | conserved hypotheticals |
| isoniazid | Rv0155 | pntAa | intermediary metabolism and respiration |
| isoniazid | Rv0262c | aac | virulence, detoxification, adaptation |
| isoniazid | Rv3399 | Rv3399 | lipid metabolism |
| isoniazid | Rv3887c | eccD2 | cell wall and cell processes |
| isoniazid | Rv0294 | tam | intermediary metabolism and respiration |
| isoniazid | Rv3509c | ilvX | intermediary metabolism and respiration |
| isoniazid | Rv2023c | Rv2023c | conserved hypotheticals |
| isoniazid | Rv3787c | Rv3787c | conserved hypotheticals |
| isoniazid | Rv2946c | pks1 | lipid metabolism |
| isoniazid | Rv0570 | nrdZ | information pathways |
| isoniazid | Rv1576c | Rv1576c | insertion seqs and phages |
| isoniazid | Rv0607 | Rv0607 | conserved hypotheticals |
| isoniazid | Rv0797 | Rv0797 | insertion seqs and phages |
| isoniazid | Rv0996 | Rv0996 | cell wall and cell processes |
| isoniazid | Rv3504 | fadE26 | lipid metabolism |
| isoniazid | Rv1160 | mutT2 | information pathways |
| isoniazid | Rv3709c | ask | intermediary metabolism and respiration |
| isoniazid | Rv0116c | ldtA | cell wall and cell processes |
| isoniazid | Rv3124 | moaR1 | regulatory proteins |
| isoniazid | Rv1401 | Rv1401 | cell wall and cell processes |
| isoniazid | Rv1787 | PPE25 | PE/PPE |
| isoniazid | Rv1810 | Rv1810 | conserved hypotheticals |
| isoniazid | Rv2120c | Rv2120c | cell wall and cell processes |
| isoniazid | Rv2384 | mbtA | lipid metabolism |
| isoniazid | Rv0596c | vapB4 | virulence, detoxification, adaptation |
| isoniazid | Rv3813c | Rv3813c | conserved hypotheticals |
| isoniazid | Rv1049 | Rv1049 | regulatory proteins |
| isoniazid | Rv0760c | Rv0760c | conserved hypotheticals |
| isoniazid | Rv0544c | Rv0544c | cell wall and cell processes |
| isoniazid | Rv0433 | Rv0433 | conserved hypotheticals |
| isoniazid | Rv2427A | oxyR' | virulence, detoxification, adaptation |
| isoniazid | Rv3367 | PE_PGRS51 | PE/PPE |
| isoniazid | Rv3705A | Rv3705A | conserved hypotheticals |
| isoniazid | Rv1568 | bioA | intermediary metabolism and respiration |
| isoniazid | Rv2149c | yfiH | conserved hypotheticals |
| isoniazid | Rv1016c | lpqT | cell wall and cell processes |
| isoniazid | Rv1316c | ogt | information pathways |
| isoniazid | Rv2076c | Rv2076c | conserved hypotheticals |
| isoniazid | Rv2063A | mazF7 | virulence, detoxification, adaptation |
| isoniazid | Rv3200c | Rv3200c | cell wall and cell processes |
| isoniazid | Rvnt24 | metV | stable RNAs |
| isoniazid | Rv1970 | lprM | cell wall and cell processes |
| isoniazid | Rv3455c | truA | information pathways |
| isoniazid | Rv3649 | Rv3649 | information pathways |
| isoniazid | Rv0560c | Rv0560c | intermediary metabolism and respiration |
| isoniazid | Rv3234c | tgs3 | lipid metabolism |
| isoniazid | Rv3439c | Rv3439c | conserved hypotheticals |
| isoniazid | Rv1422 | Rv1422 | conserved hypotheticals |
| isoniazid | Rv0645c | mmaA1 | lipid metabolism |
| isoniazid | Rv3078 | hab | intermediary metabolism and respiration |
| isoniazid | Rv3208A | TB9.4 | conserved hypotheticals |
| isoniazid | Rv3042c | serB2 | intermediary metabolism and respiration |
| isoniazid | Rv0138 | Rv0138 | conserved hypotheticals |
| isoniazid | Rv0512 | hemB | intermediary metabolism and respiration |
| isoniazid | Rv2694c | Rv2694c | conserved hypotheticals |
| isoniazid | Rv1887a | Rv1887a | NA |
| isoniazid | Rv2028c | Rv2028c | virulence, detoxification, adaptation |
| isoniazid | Rv1560 | vapB11 | virulence, detoxification, adaptation |
| isoniazid | Rv0149 | Rv0149 | intermediary metabolism and respiration |
| isoniazid | Rv3231c | Rv3231c | conserved hypotheticals |
| isoniazid | Rv0540 | Rv0540 | conserved hypotheticals |
| isoniazid | Rv1654 | argB | intermediary metabolism and respiration |
| isoniazid | Rv3758c | proV | virulence, detoxification, adaptation |
| isoniazid | Rv1684 | Rv1684 | conserved hypotheticals |
| isoniazid | Rv0918 | Rv0918 | conserved hypotheticals |
| isoniazid | Rv3029c | fixA | intermediary metabolism and respiration |
| isoniazid | Rv0661c | vapC7 | virulence, detoxification, adaptation |
| isoniazid | Rv2490c | PE_PGRS43 | PE/PPE |
| isoniazid | Rv0437c | psd | lipid metabolism |
| isoniazid | Rv3218 | Rv3218 | conserved hypotheticals |
| isoniazid | Rv1262c | Rv1262c | conserved hypotheticals |
| isoniazid | Rv0600c | Rv0600c | regulatory proteins |
| isoniazid | Rv2839c | infB | information pathways |
| isoniazid | Rv3862c | whiB6 | regulatory proteins |
| isoniazid | Rv2189c | Rv2189c | conserved hypotheticals |
| isoniazid | Rv3226c | Rv3226c | conserved hypotheticals |
| isoniazid | Rv1157c | Rv1157c | conserved hypotheticals |
| isoniazid | Rv3883c | mycP1 | intermediary metabolism and respiration |
| isoniazid | Rv2048c | pks12 | lipid metabolism |
| isoniazid | Rv3169 | Rv3169 | conserved hypotheticals |
| isoniazid | Rv2682c | dxs1 | intermediary metabolism and respiration |
| isoniazid | Rv1516c | Rv1516c | intermediary metabolism and respiration |
| isoniazid | Rv3890c | esxC | cell wall and cell processes |
| isoniazid | Rv0573c | pncB2 | intermediary metabolism and respiration |
| isoniazid | Rv2500c | fadE19 | lipid metabolism |
| isoniazid | INH | #N/A | #N/A |
| isoniazid | Rv0652 | rplL | information pathways |
| isoniazid | Rv0763c | Rv0763c | intermediary metabolism and respiration |
| isoniazid | Rv2695 | Rv2695 | conserved hypotheticals |
| isoniazid | Rv3668c | Rv3668c | intermediary metabolism and respiration |
| isoniazid | Rv2808 | Rv2808 | conserved hypotheticals |
| isoniazid | Rv3327 | Rv3327 | insertion seqs and phages |
| isoniazid | Rv3694c | Rv3694c | cell wall and cell processes |
| isoniazid | Rv3678c | Rv3678c | conserved hypotheticals |
| isoniazid | Rv1285 | cysD | intermediary metabolism and respiration |
| isoniazid | Rvnt01 | ileT | stable RNAs |
| isoniazid | Rv3017c | esxQ | cell wall and cell processes |
| isoniazid | Rv0224c | Rv0224c | intermediary metabolism and respiration |
| isoniazid | Rv1944c | Rv1944c | conserved hypotheticals |
| isoniazid | Rv1261c | Rv1261c | conserved hypotheticals |
| isoniazid | Rv3901c | Rv3901c | cell wall and cell processes |
| isoniazid | Rv3562 | fadE31 | lipid metabolism |
| isoniazid | Rv3561 | fadD3 | lipid metabolism |
| isoniazid | Rv2700 | Rv2700 | cell wall and cell processes |
| isoniazid | Rv1769 | Rv1769 | conserved hypotheticals |
| isoniazid | Rv2142c | parE2 | virulence, detoxification, adaptation |
| moxifloxacin | Rv0912 | Rv0912 | cell wall and cell processes |
| moxifloxacin | Rv3784 | epiB | intermediary metabolism and respiration |
| moxifloxacin | Rv1567c | Rv1567c | cell wall and cell processes |
| moxifloxacin | Rvnt34 | valU | stable RNAs |
| moxifloxacin | Rv1675c | cmr | regulatory proteins |
| moxifloxacin | Rv2395 | Rv2395 | cell wall and cell processes |
| moxifloxacin | Rv2297 | Rv2297 | conserved hypotheticals |
| moxifloxacin | Rv2911 | dacB2 | cell wall and cell processes |
| moxifloxacin | Rv1010 | ksgA | information pathways |
| moxifloxacin | Rv2622 | Rv2622 | intermediary metabolism and respiration |
| moxifloxacin | Rv0513 | Rv0513 | cell wall and cell processes |
| moxifloxacin | Rv2831 | echA16 | lipid metabolism |
| moxifloxacin | Rv2253 | Rv2253 | cell wall and cell processes |
| moxifloxacin | Rv0804 | Rv0804 | conserved hypotheticals |
| moxifloxacin | Rv3447c | eccC4 | cell wall and cell processes |
| moxifloxacin | Rv2408 | PE24 | PE/PPE |
| moxifloxacin | Rv3812 | PE_PGRS62 | PE/PPE |
| moxifloxacin | Rv2438A | Rv2438A | conserved hypotheticals |
| moxifloxacin | Rv1699 | pyrG | intermediary metabolism and respiration |
| moxifloxacin | Rv2564 | glnQ | cell wall and cell processes |
| moxifloxacin | Rv3521 | Rv3521 | conserved hypotheticals |
| moxifloxacin | Rv1368 | lprF | cell wall and cell processes |
| moxifloxacin | Rv3528c | Rv3528c | conserved hypotheticals |
| moxifloxacin | Rv3757c | proW | virulence, detoxification, adaptation |
| moxifloxacin | Rv2209 | Rv2209 | cell wall and cell processes |
| moxifloxacin | Rv2589 | gabT | intermediary metabolism and respiration |
| moxifloxacin | Rv1380 | pyrB | intermediary metabolism and respiration |
| moxifloxacin | Rv1736c | narX | intermediary metabolism and respiration |
| moxifloxacin | Rv2488c | Rv2488c | regulatory proteins |
| moxifloxacin | Rv2309A | Rv2309A | conserved hypotheticals |
| moxifloxacin | Rv1012 | Rv1012 | conserved hypotheticals |
| moxifloxacin | Rv2400c | subI | cell wall and cell processes |
| moxifloxacin | Rv3580c | cysS1 | information pathways |
| moxifloxacin | Rv2409c | Rv2409c | conserved hypotheticals |
| moxifloxacin | Rv1933c | fadE18 | lipid metabolism |
| moxifloxacin | Rv3591c | Rv3591c | intermediary metabolism and respiration |
| moxifloxacin | Rv1634 | Rv1634 | cell wall and cell processes |
| moxifloxacin | Rv3621c | PPE65 | PE/PPE |
| moxifloxacin | Rv1280c | oppA | cell wall and cell processes |
| moxifloxacin | Rv0510 | hemC | intermediary metabolism and respiration |
| moxifloxacin | Rv0090 | Rv0090 | cell wall and cell processes |
| moxifloxacin | Rv0271c | fadE6 | lipid metabolism |
| moxifloxacin | Rv2207 | cobT | intermediary metabolism and respiration |
| moxifloxacin | Rv2237 | Rv2237 | conserved hypotheticals |
| moxifloxacin | Rv0054 | ssb | information pathways |
| moxifloxacin | Rv0778 | cyp126 | intermediary metabolism and respiration |
| moxifloxacin | Rv0434 | Rv0434 | conserved hypotheticals |
| moxifloxacin | Rv1400c | lipI | intermediary metabolism and respiration |
| moxifloxacin | Rv0735 | sigL | information pathways |
| moxifloxacin | Rv2925c | rnc | information pathways |
| moxifloxacin | Rv0456B | mazE1 | virulence, detoxification, adaptation |
| moxifloxacin | Rv1019 | Rv1019 | regulatory proteins |
| moxifloxacin | Rv1963c | mce3R | regulatory proteins |
| moxifloxacin | Rv2366c | Rv2366c | cell wall and cell processes |
| moxifloxacin | Rv1288 | Rv1288 | conserved hypotheticals |
| moxifloxacin | Rv0493c | Rv0493c | conserved hypotheticals |
| moxifloxacin | Rv1655 | argD | intermediary metabolism and respiration |
| moxifloxacin | Rv3415c | Rv3415c | conserved hypotheticals |
| moxifloxacin | Rv0726c | Rv0726c | lipid metabolism |
| moxifloxacin | Rv3516 | echA19 | lipid metabolism |
| moxifloxacin | Rv0015c | pknA | regulatory proteins |
| moxifloxacin | Rv1245c | Rv1245c | intermediary metabolism and respiration |
| moxifloxacin | Rv0427c | xthA | information pathways |
| moxifloxacin | Rv1128c | Rv1128c | insertion seqs and phages |
| moxifloxacin | Rv1536 | ileS | information pathways |
| moxifloxacin | Rv0943c | Rv0943c | intermediary metabolism and respiration |
| moxifloxacin | Rv0839 | Rv0839 | conserved hypotheticals |
| moxifloxacin | Rv3224 | Rv3224 | intermediary metabolism and respiration |
| moxifloxacin | Rv2259 | mscR | intermediary metabolism and respiration |
| moxifloxacin | Rv3471c | Rv3471c | conserved hypotheticals |
| moxifloxacin | Rv1762c | Rv1762c | conserved hypotheticals |
| moxifloxacin | Rv0724 | sppA | cell wall and cell processes |
| moxifloxacin | Rv3905c | esxF | cell wall and cell processes |
| moxifloxacin | Rv2436 | rbsK | intermediary metabolism and respiration |
| moxifloxacin | Rv0043c | Rv0043c | regulatory proteins |
| moxifloxacin | Rv1106c | Rv1106c | intermediary metabolism and respiration |
| moxifloxacin | Rv2173 | idsA2 | lipid metabolism |
| moxifloxacin | Rv3593 | lpqF | cell wall and cell processes |
| moxifloxacin | Rv1912c | fadB5 | lipid metabolism |
| moxifloxacin | Rv3147 | nuoC | intermediary metabolism and respiration |
| moxifloxacin | Rv1318c | Rv1318c | intermediary metabolism and respiration |
| moxifloxacin | Rv2062c | cobN | intermediary metabolism and respiration |
| moxifloxacin | Rv0081 | Rv0081 | regulatory proteins |
| moxifloxacin | Rv2257c | Rv2257c | conserved hypotheticals |
| moxifloxacin | Rv0733 | adk | intermediary metabolism and respiration |
| moxifloxacin | Rv0356c | Rv0356c | conserved hypotheticals |
| moxifloxacin | Rv1181 | pks4 | lipid metabolism |
| moxifloxacin | Rv3365c | Rv3365c | conserved hypotheticals |
| moxifloxacin | Rv1497 | lipL | intermediary metabolism and respiration |
| moxifloxacin | Rv2560 | Rv2560 | cell wall and cell processes |
| moxifloxacin | Rv3235 | Rv3235 | conserved hypotheticals |
| moxifloxacin | Rv3290c | lat | intermediary metabolism and respiration |
| moxifloxacin | Rv2305 | Rv2305 | conserved hypotheticals |
| moxifloxacin | Rv2308a | Rv2308a | NA |
| moxifloxacin | Rv3201c | Rv3201c | information pathways |
| moxifloxacin | Rv0614 | Rv0614 | conserved hypotheticals |
| moxifloxacin | Rv2687c | Rv2687c | cell wall and cell processes |
| moxifloxacin | Rv0691c | Rv0691c | regulatory proteins |
| moxifloxacin | Rv1532c | Rv1532c | conserved hypotheticals |
| moxifloxacin | Rv0841 | Rv0841 | cell wall and cell processes |
| moxifloxacin | Rv0157 | pntB | intermediary metabolism and respiration |
| moxifloxacin | Rv1110 | lytB2 | cell wall and cell processes |
| moxifloxacin | Rv3688c | Rv3688c | conserved hypotheticals |
| moxifloxacin | Rv1142c | echA10 | lipid metabolism |
| moxifloxacin | Rv2843 | Rv2843 | cell wall and cell processes |
| moxifloxacin | Rv2071c | cobM | intermediary metabolism and respiration |
| moxifloxacin | Rv3336c | trpS | information pathways |
| moxifloxacin | Rv3639c | Rv3639c | conserved hypotheticals |
| moxifloxacin | Rv3270 | ctpC | cell wall and cell processes |
| moxifloxacin | Rv1557 | mmpL6 | cell wall and cell processes |
| moxifloxacin | Rv0425c | ctpH | cell wall and cell processes |
| moxifloxacin | Rv3887c | eccD2 | cell wall and cell processes |
| moxifloxacin | Rv0294 | tam | intermediary metabolism and respiration |
| moxifloxacin | Rv3552 | Rv3552 | intermediary metabolism and respiration |
| moxifloxacin | Rv2671 | ribD | intermediary metabolism and respiration |
| moxifloxacin | Rv2231c | cobC | intermediary metabolism and respiration |
| moxifloxacin | Rv0607 | Rv0607 | conserved hypotheticals |
| moxifloxacin | Rv3354 | Rv3354 | conserved hypotheticals |
| moxifloxacin | Rv0996 | Rv0996 | cell wall and cell processes |
| moxifloxacin | Rv3709c | ask | intermediary metabolism and respiration |
| moxifloxacin | Rv0116c | ldtA | cell wall and cell processes |
| moxifloxacin | Rv3124 | moaR1 | regulatory proteins |
| moxifloxacin | Rv1401 | Rv1401 | cell wall and cell processes |
| moxifloxacin | Rvns01 | rnpB | stable RNAs |
| moxifloxacin | Rv2867c | Rv2867c | intermediary metabolism and respiration |
| moxifloxacin | Rv2384 | mbtA | lipid metabolism |
| moxifloxacin | Rv3813c | Rv3813c | conserved hypotheticals |
| moxifloxacin | Rv0760c | Rv0760c | conserved hypotheticals |
| moxifloxacin | Rv1075c | Rv1075c | cell wall and cell processes |
| moxifloxacin | Rv2427A | oxyR' | virulence, detoxification, adaptation |
| moxifloxacin | Rv0433 | Rv0433 | conserved hypotheticals |
| moxifloxacin | Rv3367 | PE_PGRS51 | PE/PPE |
| moxifloxacin | Rv3550 | echA20 | lipid metabolism |
| moxifloxacin | Rv0581 | vapB26 | virulence, detoxification, adaptation |
| moxifloxacin | Rv1568 | bioA | intermediary metabolism and respiration |
| moxifloxacin | Rv3640c | Rv3640c | insertion seqs and phages |
| moxifloxacin | Rv0133 | Rv0133 | intermediary metabolism and respiration |
| moxifloxacin | Rv1016c | lpqT | cell wall and cell processes |
| moxifloxacin | Rv1316c | ogt | information pathways |
| moxifloxacin | Rv1640c | lysX | information pathways |
| moxifloxacin | Rv0723 | rplO | information pathways |
| moxifloxacin | Rv1740 | vapB34 | virulence, detoxification, adaptation |
| moxifloxacin | Rv3455c | truA | information pathways |
| moxifloxacin | Rv2908c | Rv2908c | conserved hypotheticals |
| moxifloxacin | Rv2701c | suhB | intermediary metabolism and respiration |
| moxifloxacin | Rv3649 | Rv3649 | information pathways |
| moxifloxacin | Rv0244c | fadE5 | lipid metabolism |
| moxifloxacin | Rv0222 | echA1 | lipid metabolism |
| moxifloxacin | Rv3234c | tgs3 | lipid metabolism |
| moxifloxacin | Rv0734 | mapA | intermediary metabolism and respiration |
| moxifloxacin | Rv3078 | hab | intermediary metabolism and respiration |
| moxifloxacin | Rv0961 | Rv0961 | cell wall and cell processes |
| moxifloxacin | Rv3042c | serB2 | intermediary metabolism and respiration |
| moxifloxacin | Rv0512 | hemB | intermediary metabolism and respiration |
| moxifloxacin | Rv2694c | Rv2694c | conserved hypotheticals |
| moxifloxacin | Rv1887a | Rv1887a | NA |
| moxifloxacin | Rv0482 | murB | cell wall and cell processes |
| moxifloxacin | Rv0864 | moaC2 | intermediary metabolism and respiration |
| moxifloxacin | Rv3073c | Rv3073c | conserved hypotheticals |
| moxifloxacin | Rv3520c | Rv3520c | intermediary metabolism and respiration |
| moxifloxacin | Rv1591 | Rv1591 | cell wall and cell processes |
| moxifloxacin | Rv0387c | Rv0387c | conserved hypotheticals |
| moxifloxacin | Rv1654 | argB | intermediary metabolism and respiration |
| moxifloxacin | Rv0918 | Rv0918 | conserved hypotheticals |
| moxifloxacin | Rv2505c | fadD35 | lipid metabolism |
| moxifloxacin | Rv2703 | sigA | information pathways |
| moxifloxacin | Rv2314c | Rv2314c | conserved hypotheticals |
| moxifloxacin | Rv3370c | dnaE2 | information pathways |
| moxifloxacin | Rv1210 | tagA | information pathways |
| moxifloxacin | Rv3312A | Rv3312A | cell wall and cell processes |
| moxifloxacin | Rv1255c | Rv1255c | regulatory proteins |
| moxifloxacin | Rv2490c | PE_PGRS43 | PE/PPE |
| moxifloxacin | Rv0437c | psd | lipid metabolism |
| moxifloxacin | Rv1865c | Rv1865c | intermediary metabolism and respiration |
| moxifloxacin | Rv2612c | pgsA1 | lipid metabolism |
| moxifloxacin | Rv1905c | aao | intermediary metabolism and respiration |
| moxifloxacin | Rv3169 | Rv3169 | conserved hypotheticals |
| moxifloxacin | Rv0690c | Rv0690c | conserved hypotheticals |
| moxifloxacin | Rv2682c | dxs1 | intermediary metabolism and respiration |
| moxifloxacin | Rv0490 | senX3 | regulatory proteins |
| moxifloxacin | Rv0233 | nrdB | information pathways |
| moxifloxacin | Rv2762c | Rv2762c | conserved hypotheticals |
| moxifloxacin | RVnc0010 | G2 | stable RNAs |
| moxifloxacin | Rv0652 | rplL | information pathways |
| moxifloxacin | Rv2695 | Rv2695 | conserved hypotheticals |
| moxifloxacin | Rv3062 | ligB | information pathways |
| moxifloxacin | Rv3678c | Rv3678c | conserved hypotheticals |
| moxifloxacin | Rv3549c | Rv3549c | intermediary metabolism and respiration |
| moxifloxacin | Rv0394c | Rv0394c | cell wall and cell processes |
| moxifloxacin | Rv2896c | Rv2896c | conserved hypotheticals |
| moxifloxacin | Rv1948c | Rv1948c | conserved hypotheticals |
| moxifloxacin | Rv0224c | Rv0224c | intermediary metabolism and respiration |
| moxifloxacin | Rv1944c | Rv1944c | conserved hypotheticals |
| moxifloxacin | Rv3039c | echA17 | lipid metabolism |
| moxifloxacin | Rv1261c | Rv1261c | conserved hypotheticals |
| moxifloxacin | Rv1974 | Rv1974 | cell wall and cell processes |
| moxifloxacin | Rv3287c | rsbW | information pathways |
| moxifloxacin | Rv3562 | fadE31 | lipid metabolism |
| moxifloxacin | Rv3561 | fadD3 | lipid metabolism |
| moxifloxacin | Rv0851c | Rv0851c | intermediary metabolism and respiration |
| moxifloxacin | Rv2142c | parE2 | virulence, detoxification, adaptation |
| pyrazinamide | Rv0912 | Rv0912 | cell wall and cell processes |
| pyrazinamide | Rv3784 | epiB | intermediary metabolism and respiration |
| pyrazinamide | Rv1567c | Rv1567c | cell wall and cell processes |
| pyrazinamide | Rv0754 | PE_PGRS11 | PE/PPE |
| pyrazinamide | Rv2631 | Rv2631 | conserved hypotheticals |
| pyrazinamide | Rv2911 | dacB2 | cell wall and cell processes |
| pyrazinamide | Rv1156 | Rv1156 | conserved hypotheticals |
| pyrazinamide | Rv0903c | prrA | regulatory proteins |
| pyrazinamide | Rv1010 | ksgA | information pathways |
| pyrazinamide | Rv1586c | Rv1586c | insertion seqs and phages |
| pyrazinamide | Rv3206c | moeB1 | intermediary metabolism and respiration |
| pyrazinamide | Rv0804 | Rv0804 | conserved hypotheticals |
| pyrazinamide | Rv3447c | eccC4 | cell wall and cell processes |
| pyrazinamide | Rv1030 | kdpB | cell wall and cell processes |
| pyrazinamide | Rv2408 | PE24 | PE/PPE |
| pyrazinamide | Rv1284 | canA | intermediary metabolism and respiration |
| pyrazinamide | Rv1722 | Rv1722 | lipid metabolism |
| pyrazinamide | Rv1699 | pyrG | intermediary metabolism and respiration |
| pyrazinamide | Rv0542c | menE | intermediary metabolism and respiration |
| pyrazinamide | Rv1368 | lprF | cell wall and cell processes |
| pyrazinamide | Rv3521 | Rv3521 | conserved hypotheticals |
| pyrazinamide | Rv1362c | Rv1362c | cell wall and cell processes |
| pyrazinamide | Rv1275 | lprC | cell wall and cell processes |
| pyrazinamide | Rv2273 | Rv2273 | cell wall and cell processes |
| pyrazinamide | Rv3528c | Rv3528c | conserved hypotheticals |
| pyrazinamide | Rv2892c | PPE45 | PE/PPE |
| pyrazinamide | Rv2260 | Rv2260 | conserved hypotheticals |
| pyrazinamide | Rv2484c | Rv2484c | lipid metabolism |
| pyrazinamide | Rv2209 | Rv2209 | cell wall and cell processes |
| pyrazinamide | Rv2263 | Rv2263 | intermediary metabolism and respiration |
| pyrazinamide | Rv1736c | narX | intermediary metabolism and respiration |
| pyrazinamide | Rv2309A | Rv2309A | conserved hypotheticals |
| pyrazinamide | Rv2555c | alaS | information pathways |
| pyrazinamide | Rv0184 | Rv0184 | conserved hypotheticals |
| pyrazinamide | Rv1634 | Rv1634 | cell wall and cell processes |
| pyrazinamide | Rv1484 | inhA | lipid metabolism |
| pyrazinamide | Rv1344 | mbtL | lipid metabolism |
| pyrazinamide | Rv3111 | moaC1 | intermediary metabolism and respiration |
| pyrazinamide | Rv2092c | helY | information pathways |
| pyrazinamide | Rv0510 | hemC | intermediary metabolism and respiration |
| pyrazinamide | Rv1835c | Rv1835c | conserved hypotheticals |
| pyrazinamide | Rv0271c | fadE6 | lipid metabolism |
| pyrazinamide | Rv2040c | Rv2040c | cell wall and cell processes |
| pyrazinamide | Rv2207 | cobT | intermediary metabolism and respiration |
| pyrazinamide | Rv0228 | Rv0228 | intermediary metabolism and respiration |
| pyrazinamide | Rv0054 | ssb | information pathways |
| pyrazinamide | Rv2935 | ppsE | lipid metabolism |
| pyrazinamide | Rv0778 | cyp126 | intermediary metabolism and respiration |
| pyrazinamide | Rv0429c | def | information pathways |
| pyrazinamide | Rv0115a | Rv0115a | NA |
| pyrazinamide | Rv1400c | lipI | intermediary metabolism and respiration |
| pyrazinamide | Rv1531 | Rv1531 | conserved hypotheticals |
| pyrazinamide | Rv0976c | Rv0976c | conserved hypotheticals |
| pyrazinamide | Rv2925c | rnc | information pathways |
| pyrazinamide | Rv0304c | PPE5 | PE/PPE |
| pyrazinamide | Rv3293 | pcd | intermediary metabolism and respiration |
| pyrazinamide | Rv0456B | mazE1 | virulence, detoxification, adaptation |
| pyrazinamide | Rv3331 | sugI | cell wall and cell processes |
| pyrazinamide | Rv3422c | Rv3422c | conserved hypotheticals |
| pyrazinamide | Rv0493c | Rv0493c | conserved hypotheticals |
| pyrazinamide | Rv1655 | argD | intermediary metabolism and respiration |
| pyrazinamide | Rv1103c | mazE3 | virulence, detoxification, adaptation |
| pyrazinamide | Rv0726c | Rv0726c | lipid metabolism |
| pyrazinamide | Rv1279 | Rv1279 | intermediary metabolism and respiration |
| pyrazinamide | Rv3244c | lpqB | cell wall and cell processes |
| pyrazinamide | Rv0046c | ino1 | intermediary metabolism and respiration |
| pyrazinamide | Rv0377 | Rv0377 | regulatory proteins |
| pyrazinamide | Rv1245c | Rv1245c | intermediary metabolism and respiration |
| pyrazinamide | Rv1067c | PE_PGRS19 | PE/PPE |
| pyrazinamide | Rv1070c | echA8 | lipid metabolism |
| pyrazinamide | Rv1536 | ileS | information pathways |
| pyrazinamide | Rv0943c | Rv0943c | intermediary metabolism and respiration |
| pyrazinamide | Rv1340 | rphA | information pathways |
| pyrazinamide | Rv2259 | mscR | intermediary metabolism and respiration |
| pyrazinamide | Rv3471c | Rv3471c | conserved hypotheticals |
| pyrazinamide | Rv1975 | Rv1975 | conserved hypotheticals |
| pyrazinamide | Rv1179c | Rv1179c | conserved hypotheticals |
| pyrazinamide | Rv0758 | phoR | regulatory proteins |
| pyrazinamide | Rv3905c | esxF | cell wall and cell processes |
| pyrazinamide | Rv0967 | csoR | regulatory proteins |
| pyrazinamide | Rv2227 | Rv2227 | conserved hypotheticals |
| pyrazinamide | Rv2369c | Rv2369c | conserved hypotheticals |
| pyrazinamide | Rv3593 | lpqF | cell wall and cell processes |
| pyrazinamide | Rv1912c | fadB5 | lipid metabolism |
| pyrazinamide | Rv3147 | nuoC | intermediary metabolism and respiration |
| pyrazinamide | Rvnt11 | gluT | stable RNAs |
| pyrazinamide | Rv0395 | Rv0395 | conserved hypotheticals |
| pyrazinamide | Rv2062c | cobN | intermediary metabolism and respiration |
| pyrazinamide | Rv0733 | adk | intermediary metabolism and respiration |
| pyrazinamide | Rv0115 | hddA | cell wall and cell processes |
| pyrazinamide | Rv0356c | Rv0356c | conserved hypotheticals |
| pyrazinamide | Rv2030c | Rv2030c | conserved hypotheticals |
| pyrazinamide | Rv3568c | hsaC | intermediary metabolism and respiration |
| pyrazinamide | Rv3044 | fecB | cell wall and cell processes |
| pyrazinamide | Rv2489c | Rv2489c | conserved hypotheticals |
| pyrazinamide | Rv2032 | acg | conserved hypotheticals |
| pyrazinamide | Rv1254 | Rv1254 | intermediary metabolism and respiration |
| pyrazinamide | Rv3290c | lat | intermediary metabolism and respiration |
| pyrazinamide | Rv3085 | Rv3085 | intermediary metabolism and respiration |
| pyrazinamide | Rv2127 | ansP1 | cell wall and cell processes |
| pyrazinamide | Rv0373c | Rv0373c | intermediary metabolism and respiration |
| pyrazinamide | Rv0691c | Rv0691c | regulatory proteins |
| pyrazinamide | Rv1622c | cydB | intermediary metabolism and respiration |
| pyrazinamide | Rv2646 | Rv2646 | insertion seqs and phages |
| pyrazinamide | Rv0841 | Rv0841 | cell wall and cell processes |
| pyrazinamide | Rv0157 | pntB | intermediary metabolism and respiration |
| pyrazinamide | Rv1110 | lytB2 | cell wall and cell processes |
| pyrazinamide | Rv3797 | fadE35 | lipid metabolism |
| pyrazinamide | Rv2843 | Rv2843 | cell wall and cell processes |
| pyrazinamide | Rv0955 | Rv0955 | cell wall and cell processes |
| pyrazinamide | Rv2976c | ung | information pathways |
| pyrazinamide | Rv3850 | Rv3850 | conserved hypotheticals |
| pyrazinamide | Rv0196 | Rv0196 | regulatory proteins |
| pyrazinamide | Rv1557 | mmpL6 | cell wall and cell processes |
| pyrazinamide | Rv0262c | aac | virulence, detoxification, adaptation |
| pyrazinamide | Rv3633 | Rv3633 | conserved hypotheticals |
| pyrazinamide | Rv3887c | eccD2 | cell wall and cell processes |
| pyrazinamide | Rv1252c | lprE | cell wall and cell processes |
| pyrazinamide | Rv3509c | ilvX | intermediary metabolism and respiration |
| pyrazinamide | Rv3750c | Rv3750c | insertion seqs and phages |
| pyrazinamide | Rv2671 | ribD | intermediary metabolism and respiration |
| pyrazinamide | Rv2231c | cobC | intermediary metabolism and respiration |
| pyrazinamide | Rv0607 | Rv0607 | conserved hypotheticals |
| pyrazinamide | Rv3603c | Rv3603c | conserved hypotheticals |
| pyrazinamide | Rv0674 | Rv0674 | conserved hypotheticals |
| pyrazinamide | Rv0996 | Rv0996 | cell wall and cell processes |
| pyrazinamide | Rv1445c | devB | intermediary metabolism and respiration |
| pyrazinamide | Rv1160 | mutT2 | information pathways |
| pyrazinamide | Rv0467 | icl1 | intermediary metabolism and respiration |
| pyrazinamide | Rv0116c | ldtA | cell wall and cell processes |
| pyrazinamide | Rv1315 | murA | cell wall and cell processes |
| pyrazinamide | Rv3124 | moaR1 | regulatory proteins |
| pyrazinamide | Rv3324A | Rv3324A | intermediary metabolism and respiration |
| pyrazinamide | Rv3149 | nuoE | intermediary metabolism and respiration |
| pyrazinamide | Rv0595c | vapC4 | virulence, detoxification, adaptation |
| pyrazinamide | Rvns01 | rnpB | stable RNAs |
| pyrazinamide | Rv2384 | mbtA | lipid metabolism |
| pyrazinamide | Rv1635c | Rv1635c | cell wall and cell processes |
| pyrazinamide | Rv0596c | vapB4 | virulence, detoxification, adaptation |
| pyrazinamide | Rv3813c | Rv3813c | conserved hypotheticals |
| pyrazinamide | Rv2540c | aroF | intermediary metabolism and respiration |
| pyrazinamide | Rv3033 | Rv3033 | conserved hypotheticals |
| pyrazinamide | Rv2427A | oxyR' | virulence, detoxification, adaptation |
| pyrazinamide | Rv0433 | Rv0433 | conserved hypotheticals |
| pyrazinamide | Rv2065 | cobH | intermediary metabolism and respiration |
| pyrazinamide | Rv1008 | tatD | cell wall and cell processes |
| pyrazinamide | Rv0890c | Rv0890c | regulatory proteins |
| pyrazinamide | Rv3592 | TB11.2 | intermediary metabolism and respiration |
| pyrazinamide | Rv1568 | bioA | intermediary metabolism and respiration |
| pyrazinamide | Rv3811 | csp | conserved hypotheticals |
| pyrazinamide | Rv2149c | yfiH | conserved hypotheticals |
| pyrazinamide | Rv1016c | lpqT | cell wall and cell processes |
| pyrazinamide | Rv3153 | nuoI | intermediary metabolism and respiration |
| pyrazinamide | Rv1316c | ogt | information pathways |
| pyrazinamide | Rv3432c | gadB | intermediary metabolism and respiration |
| pyrazinamide | Rv2132 | Rv2132 | conserved hypotheticals |
| pyrazinamide | Rv2914c | pknI | regulatory proteins |
| pyrazinamide | Rvnt24 | metV | stable RNAs |
| pyrazinamide | Rv3455c | truA | information pathways |
| pyrazinamide | Rv2908c | Rv2908c | conserved hypotheticals |
| pyrazinamide | Rv0696 | Rv0696 | intermediary metabolism and respiration |
| pyrazinamide | Rv3706c | Rv3706c | conserved hypotheticals |
| pyrazinamide | Rv1003 | Rv1003 | conserved hypotheticals |
| pyrazinamide | Rv0244c | fadE5 | lipid metabolism |
| pyrazinamide | Rv0222 | echA1 | lipid metabolism |
| pyrazinamide | Rv1018c | glmU | cell wall and cell processes |
| pyrazinamide | Rv0326 | Rv0326 | unknown |
| pyrazinamide | Rv1442 | bisC | intermediary metabolism and respiration |
| pyrazinamide | Rv2694c | Rv2694c | conserved hypotheticals |
| pyrazinamide | Rv0622 | Rv0622 | cell wall and cell processes |
| pyrazinamide | Rv2028c | Rv2028c | virulence, detoxification, adaptation |
| pyrazinamide | Rv2997 | Rv2997 | intermediary metabolism and respiration |
| pyrazinamide | Rv1560 | vapB11 | virulence, detoxification, adaptation |
| pyrazinamide | Rv0647c | Rv0647c | conserved hypotheticals |
| pyrazinamide | Rv1591 | Rv1591 | cell wall and cell processes |
| pyrazinamide | Rv0387c | Rv0387c | conserved hypotheticals |
| pyrazinamide | Rv3231c | Rv3231c | conserved hypotheticals |
| pyrazinamide | Rv3202c | Rv3202c | information pathways |
| pyrazinamide | Rv3758c | proV | virulence, detoxification, adaptation |
| pyrazinamide | Rv2639c | Rv2639c | cell wall and cell processes |
| pyrazinamide | Rv3370c | dnaE2 | information pathways |
| pyrazinamide | Rv1114 | vapC32 | virulence, detoxification, adaptation |
| pyrazinamide | Rv3029c | fixA | intermediary metabolism and respiration |
| pyrazinamide | Rv1210 | tagA | information pathways |
| pyrazinamide | Rv0185 | Rv0185 | conserved hypotheticals |
| pyrazinamide | Rv2490c | PE_PGRS43 | PE/PPE |
| pyrazinamide | Rv0437c | psd | lipid metabolism |
| pyrazinamide | Rv3218 | Rv3218 | conserved hypotheticals |
| pyrazinamide | Rv2593c | ruvA | information pathways |
| pyrazinamide | Rv1024 | Rv1024 | cell wall and cell processes |
| pyrazinamide | Rv1262c | Rv1262c | conserved hypotheticals |
| pyrazinamide | Rv0600c | Rv0600c | regulatory proteins |
| pyrazinamide | Rv2839c | infB | information pathways |
| pyrazinamide | Rv0982 | mprB | regulatory proteins |
| pyrazinamide | Rv3169 | Rv3169 | conserved hypotheticals |
| pyrazinamide | Rv2048c | pks12 | lipid metabolism |
| pyrazinamide | Rv0690c | Rv0690c | conserved hypotheticals |
| pyrazinamide | Rv0039c | Rv0039c | cell wall and cell processes |
| pyrazinamide | Rv0997 | Rv0997 | conserved hypotheticals |
| pyrazinamide | Rv1516c | Rv1516c | intermediary metabolism and respiration |
| pyrazinamide | Rv2500c | fadE19 | lipid metabolism |
| pyrazinamide | RVnc0010 | G2 | stable RNAs |
| pyrazinamide | Rv3594 | Rv3594 | conserved hypotheticals |
| pyrazinamide | Rv0763c | Rv0763c | intermediary metabolism and respiration |
| pyrazinamide | Rv2695 | Rv2695 | conserved hypotheticals |
| pyrazinamide | Rv3668c | Rv3668c | intermediary metabolism and respiration |
| pyrazinamide | Rv2808 | Rv2808 | conserved hypotheticals |
| pyrazinamide | Rv2791c | Rv2791c | insertion seqs and phages |
| pyrazinamide | Rv3450c | eccB4 | cell wall and cell processes |
| pyrazinamide | Rv2619c | Rv2619c | conserved hypotheticals |
| pyrazinamide | Rv1948c | Rv1948c | conserved hypotheticals |
| pyrazinamide | Rv1487 | Rv1487 | cell wall and cell processes |
| pyrazinamide | Rv0224c | Rv0224c | intermediary metabolism and respiration |
| pyrazinamide | Rv1944c | Rv1944c | conserved hypotheticals |
| pyrazinamide | Rv0826 | Rv0826 | conserved hypotheticals |
| pyrazinamide | Rv3039c | echA17 | lipid metabolism |
| pyrazinamide | Rv1261c | Rv1261c | conserved hypotheticals |
| pyrazinamide | Rv0197 | Rv0197 | intermediary metabolism and respiration |
| pyrazinamide | Rv3561 | fadD3 | lipid metabolism |
| pyrazinamide | Rv2234 | ptpA | regulatory proteins |
| rifampicin | Rv3784 | epiB | intermediary metabolism and respiration |
| rifampicin | Rv2813 | Rv2813 | conserved hypotheticals |
| rifampicin | Rv1567c | Rv1567c | cell wall and cell processes |
| rifampicin | Rvnt34 | valU | stable RNAs |
| rifampicin | Rv2631 | Rv2631 | conserved hypotheticals |
| rifampicin | Rv3729 | Rv3729 | intermediary metabolism and respiration |
| rifampicin | Rv2395 | Rv2395 | cell wall and cell processes |
| rifampicin | Rv2911 | dacB2 | cell wall and cell processes |
| rifampicin | Rv3353c | Rv3353c | conserved hypotheticals |
| rifampicin | Rv1156 | Rv1156 | conserved hypotheticals |
| rifampicin | Rv0903c | prrA | regulatory proteins |
| rifampicin | Rv1010 | ksgA | information pathways |
| rifampicin | Rv1177 | fdxC | intermediary metabolism and respiration |
| rifampicin | Rv1586c | Rv1586c | insertion seqs and phages |
| rifampicin | Rv1961 | Rv1961 | conserved hypotheticals |
| rifampicin | Rv0513 | Rv0513 | cell wall and cell processes |
| rifampicin | Rv2850c | Rv2850c | intermediary metabolism and respiration |
| rifampicin | Rv0804 | Rv0804 | conserved hypotheticals |
| rifampicin | Rv0468 | fadB2 | lipid metabolism |
| rifampicin | Rv3447c | eccC4 | cell wall and cell processes |
| rifampicin | Rv1118c | Rv1118c | conserved hypotheticals |
| rifampicin | Rv1030 | kdpB | cell wall and cell processes |
| rifampicin | Rv2408 | PE24 | PE/PPE |
| rifampicin | Rv3812 | PE_PGRS62 | PE/PPE |
| rifampicin | Rv2438A | Rv2438A | conserved hypotheticals |
| rifampicin | Rv2664 | Rv2664 | conserved hypotheticals |
| rifampicin | Rv1806 | PE20 | PE/PPE |
| rifampicin | Rv1722 | Rv1722 | lipid metabolism |
| rifampicin | Rv1699 | pyrG | intermediary metabolism and respiration |
| rifampicin | Rv3889c | espG2 | cell wall and cell processes |
| rifampicin | Rv0542c | menE | intermediary metabolism and respiration |
| rifampicin | Rv3215 | entC | intermediary metabolism and respiration |
| rifampicin | Rv3605c | Rv3605c | cell wall and cell processes |
| rifampicin | Rv2310 | Rv2310 | insertion seqs and phages |
| rifampicin | Rv3521 | Rv3521 | conserved hypotheticals |
| rifampicin | Rv1368 | lprF | cell wall and cell processes |
| rifampicin | Rv1663 | pks17 | lipid metabolism |
| rifampicin | Rv3528c | Rv3528c | conserved hypotheticals |
| rifampicin | Rv2260 | Rv2260 | conserved hypotheticals |
| rifampicin | Rv2589 | gabT | intermediary metabolism and respiration |
| rifampicin | Rv2263 | Rv2263 | intermediary metabolism and respiration |
| rifampicin | Rv0538 | Rv0538 | cell wall and cell processes |
| rifampicin | Rv1012 | Rv1012 | conserved hypotheticals |
| rifampicin | Rv3591c | Rv3591c | intermediary metabolism and respiration |
| rifampicin | Rv1634 | Rv1634 | cell wall and cell processes |
| rifampicin | Rv3621c | PPE65 | PE/PPE |
| rifampicin | Rv2656c | Rv2656c | insertion seqs and phages |
| rifampicin | Rv1991c | mazF6 | virulence, detoxification, adaptation |
| rifampicin | Rv0090 | Rv0090 | cell wall and cell processes |
| rifampicin | Rv3712 | Rv3712 | intermediary metabolism and respiration |
| rifampicin | Rv2040c | Rv2040c | cell wall and cell processes |
| rifampicin | Rv2207 | cobT | intermediary metabolism and respiration |
| rifampicin | Rv3828c | Rv3828c | insertion seqs and phages |
| rifampicin | Rv0054 | ssb | information pathways |
| rifampicin | Rv1753c | PPE24 | PE/PPE |
| rifampicin | Rv0778 | cyp126 | intermediary metabolism and respiration |
| rifampicin | Rv0429c | def | information pathways |
| rifampicin | Rv0115a | Rv0115a | NA |
| rifampicin | Rv2163c | pbpB | cell wall and cell processes |
| rifampicin | Rv1400c | lipI | intermediary metabolism and respiration |
| rifampicin | Rv1531 | Rv1531 | conserved hypotheticals |
| rifampicin | Rv0735 | sigL | information pathways |
| rifampicin | Rv0976c | Rv0976c | conserved hypotheticals |
| rifampicin | Rv2925c | rnc | information pathways |
| rifampicin | Rv1019 | Rv1019 | regulatory proteins |
| rifampicin | Rv1963c | mce3R | regulatory proteins |
| rifampicin | Rv1653 | argJ | intermediary metabolism and respiration |
| rifampicin | Rv2744c | 35kd_ag | conserved hypotheticals |
| rifampicin | Rv3422c | Rv3422c | conserved hypotheticals |
| rifampicin | Rv2723 | Rv2723 | cell wall and cell processes |
| rifampicin | Rv0493c | Rv0493c | conserved hypotheticals |
| rifampicin | Rv0862c | Rv0862c | conserved hypotheticals |
| rifampicin | Rv2019 | Rv2019 | conserved hypotheticals |
| rifampicin | Rv1655 | argD | intermediary metabolism and respiration |
| rifampicin | Rv1103c | mazE3 | virulence, detoxification, adaptation |
| rifampicin | Rv0726c | Rv0726c | lipid metabolism |
| rifampicin | Rv0393 | Rv0393 | insertion seqs and phages |
| rifampicin | Rv1702c | Rv1702c | insertion seqs and phages |
| rifampicin | Rv1245c | Rv1245c | intermediary metabolism and respiration |
| rifampicin | Rv1067c | PE_PGRS19 | PE/PPE |
| rifampicin | Rv0609A | Rv0609A | conserved hypotheticals |
| rifampicin | Rv1070c | echA8 | lipid metabolism |
| rifampicin | Rv1536 | ileS | information pathways |
| rifampicin | Rv0943c | Rv0943c | intermediary metabolism and respiration |
| rifampicin | Rv2259 | mscR | intermediary metabolism and respiration |
| rifampicin | Rv0724 | sppA | cell wall and cell processes |
| rifampicin | Rv3905c | esxF | cell wall and cell processes |
| rifampicin | Rv0043c | Rv0043c | regulatory proteins |
| rifampicin | Rv0651 | rplJ | information pathways |
| rifampicin | Rv2227 | Rv2227 | conserved hypotheticals |
| rifampicin | Rv2369c | Rv2369c | conserved hypotheticals |
| rifampicin | Rv1912c | fadB5 | lipid metabolism |
| rifampicin | Rv2716 | Rv2716 | conserved hypotheticals |
| rifampicin | Rv3147 | nuoC | intermediary metabolism and respiration |
| rifampicin | Rv1318c | Rv1318c | intermediary metabolism and respiration |
| rifampicin | Rv2062c | cobN | intermediary metabolism and respiration |
| rifampicin | Rv2299c | htpG | virulence, detoxification, adaptation |
| rifampicin | Rv0081 | Rv0081 | regulatory proteins |
| rifampicin | Rv2257c | Rv2257c | conserved hypotheticals |
| rifampicin | Rv0733 | adk | intermediary metabolism and respiration |
| rifampicin | Rv2611c | Rv2611c | lipid metabolism |
| rifampicin | Rv2916c | ffh | cell wall and cell processes |
| rifampicin | Rv2708c | Rv2708c | conserved hypotheticals |
| rifampicin | Rv0356c | Rv0356c | conserved hypotheticals |
| rifampicin | Rv1967 | mce3B | virulence, detoxification, adaptation |
| rifampicin | Rv2215 | dlaT | intermediary metabolism and respiration |
| rifampicin | Rv1628c | Rv1628c | conserved hypotheticals |
| rifampicin | Rv1181 | pks4 | lipid metabolism |
| rifampicin | Rv2489c | Rv2489c | conserved hypotheticals |
| rifampicin | Rv3107c | agpS | lipid metabolism |
| rifampicin | Rv2560 | Rv2560 | cell wall and cell processes |
| rifampicin | Rv3290c | lat | intermediary metabolism and respiration |
| rifampicin | Rv0141c | Rv0141c | conserved hypotheticals |
| rifampicin | Rv2305 | Rv2305 | conserved hypotheticals |
| rifampicin | Rv0691c | Rv0691c | regulatory proteins |
| rifampicin | Rv0834c | PE_PGRS14 | PE/PPE |
| rifampicin | Rv3227 | aroA | intermediary metabolism and respiration |
| rifampicin | Rv1532c | Rv1532c | conserved hypotheticals |
| rifampicin | Rv2646 | Rv2646 | insertion seqs and phages |
| rifampicin | Rv0841 | Rv0841 | cell wall and cell processes |
| rifampicin | Rv0157 | pntB | intermediary metabolism and respiration |
| rifampicin | Rv1110 | lytB2 | cell wall and cell processes |
| rifampicin | Rv0955 | Rv0955 | cell wall and cell processes |
| rifampicin | Rv3336c | trpS | information pathways |
| rifampicin | Rv3850 | Rv3850 | conserved hypotheticals |
| rifampicin | Rv0196 | Rv0196 | regulatory proteins |
| rifampicin | Rv1462 | Rv1462 | conserved hypotheticals |
| rifampicin | Rv1557 | mmpL6 | cell wall and cell processes |
| rifampicin | Rv0514 | Rv0514 | cell wall and cell processes |
| rifampicin | Rv2033c | Rv2033c | conserved hypotheticals |
| rifampicin | Rv3887c | eccD2 | cell wall and cell processes |
| rifampicin | Rv0294 | tam | intermediary metabolism and respiration |
| rifampicin | Rv3509c | ilvX | intermediary metabolism and respiration |
| rifampicin | Rv3750c | Rv3750c | insertion seqs and phages |
| rifampicin | Rv0570 | nrdZ | information pathways |
| rifampicin | Rv1576c | Rv1576c | insertion seqs and phages |
| rifampicin | Rv0996 | Rv0996 | cell wall and cell processes |
| rifampicin | Rv1445c | devB | intermediary metabolism and respiration |
| rifampicin | Rv3242c | Rv3242c | conserved hypotheticals |
| rifampicin | Rv1160 | mutT2 | information pathways |
| rifampicin | Rv3709c | ask | intermediary metabolism and respiration |
| rifampicin | Rv0116c | ldtA | cell wall and cell processes |
| rifampicin | Rv3638 | Rv3638 | insertion seqs and phages |
| rifampicin | Rv1787 | PPE25 | PE/PPE |
| rifampicin | Rv3818 | Rv3818 | conserved hypotheticals |
| rifampicin | Rv2464c | Rv2464c | information pathways |
| rifampicin | Rv0869c | moaA2 | intermediary metabolism and respiration |
| rifampicin | Rv2120c | Rv2120c | cell wall and cell processes |
| rifampicin | Rv2384 | mbtA | lipid metabolism |
| rifampicin | Rv1088a | Rv1088a | NA |
| rifampicin | Rv1112 | Rv1112 | conserved hypotheticals |
| rifampicin | Rv3813c | Rv3813c | conserved hypotheticals |
| rifampicin | Rv1049 | Rv1049 | regulatory proteins |
| rifampicin | Rv2540c | aroF | intermediary metabolism and respiration |
| rifampicin | Rv0760c | Rv0760c | conserved hypotheticals |
| rifampicin | Rv2428 | ahpC | virulence, detoxification, adaptation |
| rifampicin | Rv0544c | Rv0544c | cell wall and cell processes |
| rifampicin | Rv3033 | Rv3033 | conserved hypotheticals |
| rifampicin | Rv2427A | oxyR' | virulence, detoxification, adaptation |
| rifampicin | Rv0433 | Rv0433 | conserved hypotheticals |
| rifampicin | Rv0581 | vapB26 | virulence, detoxification, adaptation |
| rifampicin | Rv2697c | dut | intermediary metabolism and respiration |
| rifampicin | Rv2373c | dnaJ2 | virulence, detoxification, adaptation |
| rifampicin | Rv1723 | Rv1723 | intermediary metabolism and respiration |
| rifampicin | Rv3811 | csp | conserved hypotheticals |
| rifampicin | Rv0133 | Rv0133 | intermediary metabolism and respiration |
| rifampicin | Rv2149c | yfiH | conserved hypotheticals |
| rifampicin | Rv1016c | lpqT | cell wall and cell processes |
| rifampicin | Rv1316c | ogt | information pathways |
| rifampicin | Rv3642c | Rv3642c | conserved hypotheticals |
| rifampicin | Rv0458 | Rv0458 | intermediary metabolism and respiration |
| rifampicin | Rv1029 | kdpA | cell wall and cell processes |
| rifampicin | Rv1640c | lysX | information pathways |
| rifampicin | Rv2132 | Rv2132 | conserved hypotheticals |
| rifampicin | Rv2787 | Rv2787 | conserved hypotheticals |
| rifampicin | Rvnt24 | metV | stable RNAs |
| rifampicin | Rv1740 | vapB34 | virulence, detoxification, adaptation |
| rifampicin | Rv2908c | Rv2908c | conserved hypotheticals |
| rifampicin | Rv0696 | Rv0696 | intermediary metabolism and respiration |
| rifampicin | Rv1003 | Rv1003 | conserved hypotheticals |
| rifampicin | Rv2701c | suhB | intermediary metabolism and respiration |
| rifampicin | Rv0560c | Rv0560c | intermediary metabolism and respiration |
| rifampicin | Rv0244c | fadE5 | lipid metabolism |
| rifampicin | Rv0222 | echA1 | lipid metabolism |
| rifampicin | Rv3234c | tgs3 | lipid metabolism |
| rifampicin | Rv1422 | Rv1422 | conserved hypotheticals |
| rifampicin | Rv3078 | hab | intermediary metabolism and respiration |
| rifampicin | Rv3208A | TB9.4 | conserved hypotheticals |
| rifampicin | Rv1018c | glmU | cell wall and cell processes |
| rifampicin | Rv2924c | fpg | information pathways |
| rifampicin | Rv2694c | Rv2694c | conserved hypotheticals |
| rifampicin | Rv1887a | Rv1887a | NA |
| rifampicin | Rv1330c | pncB1 | intermediary metabolism and respiration |
| rifampicin | Rv1560 | vapB11 | virulence, detoxification, adaptation |
| rifampicin | Rv0647c | Rv0647c | conserved hypotheticals |
| rifampicin | Rv3073c | Rv3073c | conserved hypotheticals |
| rifampicin | Rv0387c | Rv0387c | conserved hypotheticals |
| rifampicin | Rv3231c | Rv3231c | conserved hypotheticals |
| rifampicin | Rv2220 | glnA1 | intermediary metabolism and respiration |
| rifampicin | Rv1654 | argB | intermediary metabolism and respiration |
| rifampicin | Rv3758c | proV | virulence, detoxification, adaptation |
| rifampicin | Rv1684 | Rv1684 | conserved hypotheticals |
| rifampicin | Rv3159c | PPE53 | PE/PPE |
| rifampicin | Rv3726 | Rv3726 | intermediary metabolism and respiration |
| rifampicin | Rv2703 | sigA | information pathways |
| rifampicin | Rv3857c | Rv3857c | cell wall and cell processes |
| rifampicin | Rv3533c | PPE62 | PE/PPE |
| rifampicin | Rv3029c | fixA | intermediary metabolism and respiration |
| rifampicin | Rv0661c | vapC7 | virulence, detoxification, adaptation |
| rifampicin | Rv0185 | Rv0185 | conserved hypotheticals |
| rifampicin | Rv2490c | PE_PGRS43 | PE/PPE |
| rifampicin | Rv2426c | Rv2426c | conserved hypotheticals |
| rifampicin | Rv1686c | Rv1686c | cell wall and cell processes |
| rifampicin | Rv0437c | psd | lipid metabolism |
| rifampicin | Rv3218 | Rv3218 | conserved hypotheticals |
| rifampicin | Rv1024 | Rv1024 | cell wall and cell processes |
| rifampicin | Rv3400 | Rv3400 | intermediary metabolism and respiration |
| rifampicin | Rv0600c | Rv0600c | regulatory proteins |
| rifampicin | Rv2839c | infB | information pathways |
| rifampicin | Rv3862c | whiB6 | regulatory proteins |
| rifampicin | Rv1905c | aao | intermediary metabolism and respiration |
| rifampicin | Rvnt33 | cysU | stable RNAs |
| rifampicin | Rv2048c | pks12 | lipid metabolism |
| rifampicin | Rv3169 | Rv3169 | conserved hypotheticals |
| rifampicin | Rv0690c | Rv0690c | conserved hypotheticals |
| rifampicin | Rv2682c | dxs1 | intermediary metabolism and respiration |
| rifampicin | Rv0965c | Rv0965c | conserved hypotheticals |
| rifampicin | Rv3194c | Rv3194c | cell wall and cell processes |
| rifampicin | Rv3087 | Rv3087 | lipid metabolism |
| rifampicin | Rv2762c | Rv2762c | conserved hypotheticals |
| rifampicin | Rv2500c | fadE19 | lipid metabolism |
| rifampicin | Rv3014c | ligA | information pathways |
| rifampicin | Rv2526 | vapB17 | virulence, detoxification, adaptation |
| rifampicin | Rv2122c | hisE | intermediary metabolism and respiration |
| rifampicin | Rv2808 | Rv2808 | conserved hypotheticals |
| rifampicin | Rv0975c | fadE13 | lipid metabolism |
| rifampicin | Rv0755c | PPE12 | PE/PPE |
| rifampicin | Rv3694c | Rv3694c | cell wall and cell processes |
| rifampicin | Rv3678c | Rv3678c | conserved hypotheticals |
| rifampicin | Rv1948c | Rv1948c | conserved hypotheticals |
| rifampicin | Rv2404c | lepA | intermediary metabolism and respiration |
| rifampicin | Rv0183 | Rv0183 | intermediary metabolism and respiration |
| rifampicin | Rv1944c | Rv1944c | conserved hypotheticals |
| rifampicin | Rv1455 | Rv1455 | conserved hypotheticals |
| rifampicin | Rv3039c | echA17 | lipid metabolism |
| rifampicin | Rv1261c | Rv1261c | conserved hypotheticals |
| rifampicin | Rv2931 | ppsA | lipid metabolism |
| rifampicin | Rv0817c | Rv0817c | cell wall and cell processes |
| rifampicin | Rv3562 | fadE31 | lipid metabolism |
| rifampicin | Rv2142c | parE2 | virulence, detoxification, adaptation |
| rifampicin | Rv0851c | Rv0851c | intermediary metabolism and respiration |

Supplementary Table 5. List of unique drug-specific genes for all six individual-drug blood cultures.

| Drug | Locus tag | Gene symbol | Gene description |
| --- | --- | --- | --- |
| ethambutol | Rv3171c | hpx | non-heme haloperoxidase Hpx |
| ethambutol | Rv3100c | smpB | SsrA-binding protein |
| ethambutol | Rv0536 | galE3 | UDP-glucose 4-epimerase GalE |
| ethambutol | Rv0127 | mak | maltokinase |
| ethambutol | Rv2339 | mmpL9 | transmembrane transport protein MmpL9 |
| ethambutol | Rv0574c | Rv0574c | hypothetical protein |
| ethambutol | Rv1221 | sigE | ECF RNA polymerase sigma factor SigE |
| ethambutol | Rv0431 | Rv0431 | tuberculin-like peptide |
| ethambutol | Rv1474c | Rv1474c | transcriptional regulator |
| ethambutol | Rv0089 | Rv0089 | methyltransferase |
| ethambutol | Rv2672 | Rv2672 | protease |
| ethambutol | Rv1258c | Rv1258c | multidrug-efflux transporter |
| ethambutol | Rv1226c | Rv1226c | transmembrane protein |
| ethambutol | Rv3125c | PPE49 | PPE family protein PPE49 |
| ethambutol | Rv2894c | xerC | tyrosine recombinase XerC |
| ethambutol | Rv1972 | Rv1972 | Mce associated membrane protein |
| ethambutol | Rv3891c | esxD | ESAT-6 like protein EsxD |
| ethambutol | Rv2833c | ugpB | sn-glycerol-3-phosphate ABC transporter substrate-binding lipoprotein UgpB |
| ethambutol | Rv0872c | PE_PGRS15 | PE-PGRS family protein PE_PGRS15 |
| ethambutol | Rv2527 | vapC17 | ribonuclease VapC17 |
| ethambutol | Rv1777 | cyp144 | cytochrome P450 Cyp144 |
| ethambutol | Rv2331 | Rv2331 | hypothetical protein |
| ethambutol | Rv3298c | lpqC | esterase LpqC |
| ethambutol | Rv3096 | Rv3096 | hypothetical protein |
| ethambutol | Rv3081 | Rv3081 | hypothetical protein |
| ethambutol | Rv2451 | Rv2451 | hypothetical protein |
| ethambutol | Rv2176 | pknL | serinethreonine-protein kinase PknL |
| ethambutol | Rv3498c | mce4B | Mce family protein Mce4B |
| ethambutol | Rv1048c | Rv1048c | hypothetical protein |
| ethambutol | Rv1087 | PE_PGRS21 | PE-PGRS family protein PE_PGRS21 |
| ethambutol | Rv2360c | Rv2360c | hypothetical protein |
| ethambutol | Rv2219 | Rv2219 | transmembrane protein |
| ethambutol | Rv3123 | Rv3123 | hypothetical protein |
| ethambutol | Rv0828c | Rv0828c | deaminase |
| ethambutol | Rv1486c | Rv1486c | hypothetical protein |
| ethambutol | Rv1341 | Rv1341 | non-canonical purine NTP pyrophosphatase |
| ethambutol | Rv3164c | moxR3 | methanol dehydrogenase transcriptional regulator MoxR |
| ethambutol | Rv1984c | cfp21 | cutinase |
| ethambutol | Rv0160c | PE4 | PE family protein PE4 |
| ethambutol | Rv2923c | Rv2923c | hypothetical protein |
| ethambutol | Rv3071 | Rv3071 | hypothetical protein |
| ethambutol | Rv2798c | Rv2798c | hypothetical protein |
| ethambutol | Rv3752c | Rv3752c | cytidinedeoxycytidylate deaminase |
| ethambutol | Rv0396 | Rv0396 | hypothetical protein |
| ethambutol | Rv3077 | atsF | hydrolase |
| ethambutol | Rv1150 | Rv1150 | Rv1150 |
| ethambutol | Rv3748 | Rv3748 | hypothetical protein |
| ethambutol | Rv1248c | Rv1248c | multifunctional 2-oxoglutarate dehydrogenase E1 component /2-oxoglutarate dehydrogenase dihydrolipoyllysine-residue succinyltransferase |
| ethambutol | Rv0107c | ctpI | cation-transporter ATPase I |
| ethambutol | Rv2160c | Rv2160c | Rv2160c |
| ethambutol | Rv1202 | dapE | succinyl-diaminopimelate desuccinylase DapE |
| ethambutol | Rv1407 | fmu | 16S rRNA m5C967 methyltransferase |
| ethambutol | Rv0884c | serC | phosphoserine aminotransferase |
| ethambutol | Rv1534 | Rv1534 | transcriptional regulator |
| ethambutol | Rv2735c | Rv2735c | hypothetical protein |
| ethambutol | Rv2917 | Rv2917 | hypothetical protein |
| ethambutol | Rv3532 | PPE61 | PPE family protein PPE61 |
| ethambutol | Rv0775 | Rv0775 | hypothetical protein |
| faropenem | Rv3031 | Rv3031 | 1%2C4-alpha-glucan-branching protein |
| faropenem | Rv3090 | Rv3090 | hypothetical protein |
| faropenem | Rv2877c | merT | integral membrane protein |
| faropenem | Rv3573c | fadE34 | acyl-CoA dehydrogenase FadE34 |
| faropenem | Rv1376 | Rv1376 | hypothetical protein |
| faropenem | Rv2621c | Rv2621c | transcriptional regulator |
| faropenem | Rv1545 | Rv1545 | hypothetical protein |
| faropenem | Rv2147c | Rv2147c | cell division protein SepF |
| faropenem | Rv1148c | Rv1148c | hypothetical protein |
| faropenem | Rv0096 | PPE1 | PPE family protein PPE1 |
| faropenem | Rv1647 | Rv1647 | adenylate cyclase |
| faropenem | Rv1140 | Rv1140 | integral membrane protein |
| faropenem | Rv2509 | Rv2509 | short-chain type dehydrogenasereductase |
| faropenem | Rv3538 | hsd4B | dehydrogenase |
| faropenem | Rv3444c | esxT | ESAT-6 like protein EsxT |
| faropenem | Rv3324c | moaC3 | cyclic pyranopterin monophosphate synthase accessory protein |
| faropenem | Rvnt19 | leuW | 0 |
| faropenem | Rv2835c | ugpA | sn-glycerol-3-phosphate ABC transporter permease UgpA |
| faropenem | Rv1367c | Rv1367c | hypothetical protein |
| faropenem | Rv0504c | Rv0504c | hypothetical protein |
| faropenem | Rv3501c | yrbE4A | integral membrane protein |
| faropenem | Rv1015c | rplY | 50S ribosomal protein L25general stress protein Ctc |
| faropenem | Rv1007c | metS | methionine--tRNA ligase |
| faropenem | Rv0807 | Rv0807 | hypothetical protein |
| faropenem | Rv3268 | Rv3268 | hypothetical protein |
| faropenem | Rv2528c | mrr | restriction system protein |
| faropenem | Rv1175c | fadH | NADPH dependent 2%2C4-dienoyl-CoA reductase FadH |
| faropenem | Rv1129c | Rv1129c | transcriptional regulator |
| faropenem | Rv2626c | hrp1 | hypoxic response protein |
| faropenem | Rv2357c | glyS | glycine--tRNA ligase |
| faropenem | Rv3243c | Rv3243c | hypothetical protein |
| faropenem | Rv2416c | eis | enhanced intracellular survival protein |
| faropenem | Rv1901 | cinA | competence damage-inducible protein CinA |
| faropenem | Rv1575 | Rv1575 | phage protein |
| faropenem | Rv2110c | prcB | proteasome subunit beta |
| faropenem | Rv3720 | Rv3720 | fatty acid synthase |
| faropenem | Rv1373 | Rv1373 | glycolipid sulfotransferase |
| faropenem | Rv3548c | Rv3548c | short-chain type dehydrogenasereductase |
| faropenem | Rv3204 | Rv3204 | DNA-methyltransferase |
| faropenem | Rv3476c | kgtP | #N/A |
| faropenem | Rv1900c | lipJ | lignin peroxidase LipJ |
| faropenem | Rv1850 | ureC | urease subunit alpha |
| faropenem | Rv1982A | vapB36 | antitoxin VapB36 |
| isoniazid | Rv2854 | Rv2854 | hypothetical protein |
| isoniazid | Rv1841c | Rv1841c | hypothetical protein |
| isoniazid | Rv0892 | Rv0892 | monooxygenase |
| isoniazid | Rv3139 | fadE24 | acyl-CoA dehydrogenase |
| isoniazid | Rv1432 | Rv1432 | dehydrogenase |
| isoniazid | Rv2432c | Rv2432c | hypothetical protein |
| isoniazid | Rv2023c | Rv2023c | hypothetical protein |
| isoniazid | Rv3314c | deoA | thymidine phosphorylase |
| isoniazid | Rv2782c | pepR | zinc protease |
| isoniazid | Rv1448c | tal | transaldolase |
| isoniazid | Rv0302 | Rv0302 | transcriptional regulator |
| isoniazid | Rv1781c | malQ | 4-alpha-glucanotransferase |
| isoniazid | Rv0992c | Rv0992c | 5-formyltetrahydrofolate cyclo-ligase |
| isoniazid | Rv3200c | Rv3200c | transmembrane cation transporter |
| isoniazid | Rv1839c | vapB13 | antitoxin VapB13 |
| isoniazid | Rv0475 | hbhA | heparin binding hemagglutinin HbhA |
| isoniazid | Rv3439c | Rv3439c | hypothetical protein |
| isoniazid | Rv0450c | mmpL4 | transmembrane transport protein MmpL4 |
| isoniazid | Rv0097 | Rv0097 | oxidoreductase |
| isoniazid | Rv0645c | mmaA1 | mycolic acid methyltransferase MmaA1 |
| isoniazid | Rv3502c | hsd4A | 3-oxoacyl-ACP reductase |
| isoniazid | Rv1934c | fadE17 | acyl-CoA dehydrogenase FadE17 |
| isoniazid | Rv0138 | Rv0138 | hypothetical protein |
| isoniazid | Rv1824 | Rv1824 | hypothetical protein |
| isoniazid | Rv3468c | rmlB3 | dTDP-glucose 4%2C6-dehydratase |
| isoniazid | Rv0419 | lpqM | lipoprotein peptidase LpqM |
| isoniazid | Rv2599 | Rv2599 | membrane protein |
| isoniazid | Rv2759c | vapC42 | ribonuclease VapC42 |
| isoniazid | Rv0863 | Rv0863 | hypothetical protein |
| isoniazid | Rv1506c | Rv1506c | hypothetical protein |
| isoniazid | Rv2189c | Rv2189c | hypothetical protein |
| isoniazid | Rv3226c | Rv3226c | hypothetical protein |
| isoniazid | Rv3883c | mycP1 | membrane-anchored mycosin |
| isoniazid | Rv2298 | Rv2298 | oxidoreductase |
| isoniazid | Rv3000 | Rv3000 | transmembrane protein |
| isoniazid | Rv1679 | fadE16 | acyl-CoA dehydrogenase FadE16 |
| isoniazid | Rv3890c | esxC | ESAT-6 like protein EsxC |
| isoniazid | Rv1858 | modB | molybdenum ABC transporter permease ModB |
| isoniazid | Rv3327 | Rv3327 | transposase fusion protein |
| isoniazid | Rv1285 | cysD | sulfate adenylyltransferase subunit 2 |
| isoniazid | Rv3901c | Rv3901c | membrane protein |
| isoniazid | Rv2984 | ppk1 | polyphosphate kinase |
| isoniazid | Rvnt27 | glyV | 0 |
| isoniazid | Rv2700 | Rv2700 | hypothetical protein |
| isoniazid | Rv1769 | Rv1769 | hypothetical protein |
| moxifloxacin | Rv1675c | cmr | HTH-type transcriptional regulator Cmr |
| moxifloxacin | Rv0394c | Rv0394c | hypothetical protein |
| moxifloxacin | Rv0425c | ctpH | metal cation transporting ATPase H |
| moxifloxacin | Rv0482 | murB | UDP-N-acetylenolpyruvoylglucosamine reductase |
| moxifloxacin | Rv0490 | senX3 | two component sensor histidine kinase SenX3 |
| moxifloxacin | Rv0614 | Rv0614 | hypothetical protein |
| moxifloxacin | Rv0723 | rplO | 50S ribosomal protein L15 |
| moxifloxacin | Rv0734 | mapA | methionine aminopeptidase |
| moxifloxacin | Rv0864 | moaC2 | cyclic pyranopterin monophosphate synthase accessory protein |
| moxifloxacin | Rv0961 | Rv0961 | integral membrane protein |
| moxifloxacin | Rv1128c | Rv1128c | hypothetical protein |
| moxifloxacin | Rv1255c | Rv1255c | HTH-type transcriptional regulator |
| moxifloxacin | Rv1280c | oppA | oligopeptide ABC transporter substrate-binding lipoprotein OppA |
| moxifloxacin | Rv1380 | pyrB | aspartate carbamoyltransferase |
| moxifloxacin | Rv1865c | Rv1865c | short-chain type dehydrogenase |
| moxifloxacin | Rv1933c | fadE18 | acyl-CoA dehydrogenase FadE18 |
| moxifloxacin | Rv1974 | Rv1974 | membrane protein |
| moxifloxacin | Rv2071c | cobM | precorrin-4 C(11)-methyltransferase |
| moxifloxacin | Rv2173 | idsA2 | geranylgeranyl pyrophosphate synthetase IdsA |
| moxifloxacin | Rv2237 | Rv2237 | hypothetical protein |
| moxifloxacin | Rv2297 | Rv2297 | hypothetical protein |
| moxifloxacin | Rv2308a | Rv2308a | hypothetical protein |
| moxifloxacin | Rv2366c | Rv2366c | transmembrane protein |
| moxifloxacin | Rv2409c | Rv2409c | hypothetical protein |
| moxifloxacin | Rv2505c | fadD35 | fatty-acid--CoA ligase FadD35 |
| moxifloxacin | Rv2612c | pgsA1 | CDP-diacylglycerol--inositol 3-phosphatidyltransferase |
| moxifloxacin | Rv2622 | Rv2622 | methyltransferase |
| moxifloxacin | Rv2867c | Rv2867c | GCN5-like N-acetyltransferase |
| moxifloxacin | Rv2896c | Rv2896c | hypothetical protein |
| moxifloxacin | Rv3201c | Rv3201c | ATP-dependent DNA helicase |
| moxifloxacin | Rv3287c | rsbW | anti-sigma factor RsbW |
| moxifloxacin | Rv3312A | Rv3312A | pilin |
| moxifloxacin | Rv3516 | echA19 | enoyl-CoA hydratase EchA19 |
| moxifloxacin | Rv3520c | Rv3520c | coenzyme F420-dependent oxidoreductase |
| moxifloxacin | Rv3549c | Rv3549c | short-chain type dehydrogenasereductase |
| moxifloxacin | Rv3550 | echA20 | enoyl-CoA hydratase EchA20 |
| moxifloxacin | Rv3552 | Rv3552 | CoA-transferase subunit beta |
| moxifloxacin | Rv3639c | Rv3639c | hypothetical protein |
| moxifloxacin | Rv3640c | Rv3640c | transposase |
| pyrazinamide | Rv1622c | cydB | cytochrome D ubiquinol oxidase subunit II CydB |
| pyrazinamide | Rv3797 | fadE35 | acyl-CoA dehydrogenase FadE35 |
| pyrazinamide | Rv3633 | Rv3633 | hypothetical protein |
| pyrazinamide | Rv3206c | moeB1 | adenylyltransferase/sulfurtransferase MoeZ |
| pyrazinamide | Rv0674 | Rv0674 | hypothetical protein |
| pyrazinamide | Rv1275 | lprC | lipoprotein LprC |
| pyrazinamide | Rv0467 | icl1 | isocitrate lyase |
| pyrazinamide | Rv1315 | murA | UDP-N-acetylglucosamine 1-carboxyvinyltransferase |
| pyrazinamide | Rv2892c | PPE45 | PPE family protein PPE45 |
| pyrazinamide | Rv3324A | Rv3324A | Rv3324A |
| pyrazinamide | Rv2484c | Rv2484c | diacyglycerol O-acyltransferase |
| pyrazinamide | Rv3149 | nuoE | NADH-quinone oxidoreductase subunit E |
| pyrazinamide | Rv1635c | Rv1635c | mannosyltransferase |
| pyrazinamide | Rv1008 | tatD | deoxyribonuclease TatD |
| pyrazinamide | Rv3111 | moaC1 | cyclic pyranopterin monophosphate synthase accessory protein |
| pyrazinamide | Rv0890c | Rv0890c | HTH-type transcriptional regulator |
| pyrazinamide | Rv3592 | TB11.2 | heme-degrading monooxygenase |
| pyrazinamide | Rv3432c | gadB | glutamate decarboxylase GadB |
| pyrazinamide | Rv0228 | Rv0228 | acyltransferase |
| pyrazinamide | Rv2935 | ppsE | phthiocerol synthesis polyketide synthase type I PpsE |
| pyrazinamide | Rv2914c | pknI | serine/threonine-protein kinase PknI |
| pyrazinamide | Rv0304c | PPE5 | PPE family protein PPE5 |
| pyrazinamide | Rv1442 | bisC | biotin sulfoxide reductase BisC |
| pyrazinamide | Rv0622 | Rv0622 | membrane protein |
| pyrazinamide | Rv1279 | Rv1279 | GMC-type oxidoreductase |
| pyrazinamide | Rv3244c | lpqB | lipoprotein LpqB |
| pyrazinamide | Rv0046c | ino1 | inositol-3-phosphate synthase |
| pyrazinamide | Rv0377 | Rv0377 | HTH-type transcriptional regulator |
| pyrazinamide | Rv2639c | Rv2639c | integral membrane protein |
| pyrazinamide | Rv1340 | rphA | ribonuclease PH |
| pyrazinamide | Rv1114 | vapC32 | ribonuclease VapC32 |
| pyrazinamide | Rv0758 | phoR | two component system response sensor kinase PhoR |
| pyrazinamide | Rv2593c | ruvA | Holliday junction ATP-dependent DNA helicase RuvA |
| pyrazinamide | Rv0967 | csoR | copper-sensing transcriptional repressor CsoR |
| pyrazinamide | Rv0982 | mprB | two component histidine-protein kinase/phosphatase MprB |
| pyrazinamide | Rvnt11 | gluT | 0 |
| pyrazinamide | Rv0039c | Rv0039c | transmembrane protein |
| pyrazinamide | Rv0997 | Rv0997 | hypothetical protein |
| pyrazinamide | Rv0115 | hddA | D-alpha-D-heptose-7-phosphate kinase HddA |
| pyrazinamide | Rv3594 | Rv3594 | hypothetical protein |
| pyrazinamide | Rv2791c | Rv2791c | transposase |
| pyrazinamide | Rv3450c | eccB4 | ESX-4 secretion system protein EccB4 |
| pyrazinamide | Rv3568c | hsaC | extradiol dioxygenase |
| pyrazinamide | Rv3044 | fecB | FeIII-dicitrate-binding periplasmic lipoprotein |
| pyrazinamide | Rv2032 | acg | NAD(P)H nitroreductase |
| pyrazinamide | Rv1254 | Rv1254 | acyltransferase |
| pyrazinamide | Rv3085 | Rv3085 | oxidoreductase SadH |
| rifampicin | Rv3227 | aroA | 3-phosphoshikimate 1-carboxyvinyltransferase |
| rifampicin | Rv3729 | Rv3729 | transferase |
| rifampicin | Rv1462 | Rv1462 | hypothetical protein |
| rifampicin | Rv2033c | Rv2033c | hypothetical protein |
| rifampicin | Rv1118c | Rv1118c | hypothetical protein |
| rifampicin | Rv1806 | PE20 | PE family protein PE20 |
| rifampicin | Rv2664 | Rv2664 | hypothetical protein |
| rifampicin | Rv3605c | Rv3605c | hypothetical protein |
| rifampicin | Rv2310 | Rv2310 | excisionase |
| rifampicin | Rv3242c | Rv3242c | hypothetical protein |
| rifampicin | Rv1663 | pks17 | polyketide synthase |
| rifampicin | Rv2464c | Rv2464c | DNA glycosylase |
| rifampicin | Rv0869c | moaA2 | molybdenum cofactor biosynthesis protein MoaA |
| rifampicin | Rv0538 | Rv0538 | membrane protein |
| rifampicin | Rv1088a | Rv1088a | hypothetical protein |
| rifampicin | Rv1112 | Rv1112 | GTP-binding protein |
| rifampicin | Rv2697c | dut | deoxyuridine 5'-triphosphate nucleotidohydrolase |
| rifampicin | Rv2373c | dnaJ2 | chaperone protein DnaJ |
| rifampicin | Rv1723 | Rv1723 | hydrolase |
| rifampicin | Rv0458 | Rv0458 | aldehyde dehydrogenase |
| rifampicin | Rv1029 | kdpA | potassium-transporting ATPase subunit A |
| rifampicin | Rv1753c | PPE24 | PPE family protein PPE24 |
| rifampicin | Rv2787 | Rv2787 | hypothetical protein |
| rifampicin | Rv2163c | pbpB | penicillin-binding membrane protein PbpB |
| rifampicin | Rv2924c | fpg | formamidopyrimidine-DNA glycosylase |
| rifampicin | Rv1330c | pncB1 | nicotinic acid phosphoribosyltransferase PncB1 |
| rifampicin | Rv1702c | Rv1702c | hypothetical protein |
| rifampicin | Rv2220 | glnA1 | glutamine synthetase |
| rifampicin | Rv3726 | Rv3726 | dehydrogenase |
| rifampicin | Rv3533c | PPE62 | PPE family protein PPE62 |
| rifampicin | Rv2426c | Rv2426c | hypothetical protein |
| rifampicin | Rv3400 | Rv3400 | hydrolase |
| rifampicin | Rv2299c | htpG | chaperone protein HtpG |
| rifampicin | Rv2611c | Rv2611c | phosphatidylinositol mannoside acyltransferase |
| rifampicin | Rv0975c | fadE13 | acyl-CoA dehydrogenase FadE13 |
| rifampicin | Rv2215 | dlaT | pyruvate dehydrogenase E2 component dihydrolipoamide acyltransferase |
| rifampicin | Rv2404c | lepA | GTP-binding protein LepA |
| rifampicin | Rv0183 | Rv0183 | lysophospholipase |
| rifampicin | Rv1455 | Rv1455 | hypothetical protein |
| rifampicin | Rv0141c | Rv0141c | hypothetical protein |
| rifampicin | Rv2931 | ppsA | phthiocerol synthesis polyketide synthase type I PpsA |
| rifampicin | Rv0817c | Rv0817c | hypothetical protein |

Supplementary Table 6. Significant DAVID pathways of overall drug-specific genes for all six individual-drug blood cultures.

| Drug | DAVID database source | Pathway name | PValue |
| --- | --- | --- | --- |
| isoniazid | SMART | SM00829:SM00829 | 0.004 |
| isoniazid | INTERPRO | IPR020843:Polyketide synthase, enoylreductase | 0.005 |
| isoniazid | INTERPRO | IPR013154:Alcohol dehydrogenase GroES-like | 0.008 |
| isoniazid | INTERPRO | IPR011032:GroES-like | 0.010 |
| isoniazid | GOTERM_MF_DIRECT | GO:0042802~identical protein binding | 0.010 |
| isoniazid | INTERPRO | IPR006091:Acyl-CoA oxidase/dehydrogenase, central domain | 0.014 |
| isoniazid | INTERPRO | IPR013149:Alcohol dehydrogenase, C-terminal | 0.021 |
| isoniazid | INTERPRO | IPR013786:Acyl-CoA dehydrogenase/oxidase, N-terminal | 0.024 |
| isoniazid | KEGG_PATHWAY | mtu01210:2-Oxocarboxylic acid metabolism | 0.025 |
| isoniazid | GOTERM_BP_DIRECT | GO:0033539~fatty acid beta-oxidation using acyl-CoA dehydrogenase | 0.028 |
| isoniazid | GOTERM_BP_DIRECT | GO:0055088~lipid homeostasis | 0.028 |
| isoniazid | UP_KEYWORDS | FAD | 0.035 |
| isoniazid | INTERPRO | IPR005814:Aminotransferase class-III | 0.038 |
| isoniazid | INTERPRO | IPR009100:Acyl-CoA dehydrogenase/oxidase | 0.039 |
| isoniazid | INTERPRO | IPR009075:Acyl-CoA dehydrogenase/oxidase C-terminal | 0.039 |
| isoniazid | GOTERM_MF_DIRECT | GO:0052890~oxidoreductase activity, acting on the CH-CH group of donors, with a flavin as acceptor | 0.041 |
| isoniazid | GOTERM_MF_DIRECT | GO:0003995~acyl-CoA dehydrogenase activity | 0.041 |
| isoniazid | GOTERM_MF_DIRECT | GO:0000062~fatty-acyl-CoA binding | 0.045 |
| isoniazid | GOTERM_BP_DIRECT | GO:0032259~methylation | 0.050 |
| rifampicin | GOTERM_CC_DIRECT | GO:0005737~cytoplasm | 0.018 |
| rifampicin | UP_KEYWORDS | Multifunctional enzyme | 0.020 |
| rifampicin | UP_SEQ_FEATURE | compositionally biased region:Asn-rich | 0.022 |
| rifampicin | GOTERM_BP_DIRECT | GO:0006260~DNA replication | 0.024 |
| rifampicin | INTERPRO | IPR005814:Aminotransferase class-III | 0.049 |
| ethambutol | GOTERM_BP_DIRECT | GO:0006526~arginine biosynthetic process | 0.019 |
| ethambutol | GOTERM_MF_DIRECT | GO:0008270~zinc ion binding | 0.032 |
| ethambutol | KEGG_PATHWAY | mtu03410:Base excision repair | 0.034 |
| ethambutol | UP_SEQ_FEATURE | region of interest:Pyridoxal phosphate binding | 0.040 |
| ethambutol | UP_SEQ_FEATURE | binding site:Pyridoxal phosphate | 0.040 |
| ethambutol | GOTERM_MF_DIRECT | GO:0004871~signal transducer activity | 0.041 |
| ethambutol | UP_SEQ_FEATURE | compositionally biased region:Ala-rich | 0.048 |
| pyrazinamide | INTERPRO | IPR006656:Molybdopterin oxidoreductase | 0.039 |
| pyrazinamide | INTERPRO | IPR005814:Aminotransferase class-III | 0.039 |
| pyrazinamide | GOTERM_BP_DIRECT | GO:0009103~lipopolysaccharide biosynthetic process | 0.049 |
| moxifloxacin | INTERPRO | IPR005814:Aminotransferase class-III | 0.002 |
| moxifloxacin | INTERPRO | IPR001753:Crotonase superfamily | 0.004 |
| moxifloxacin | KEGG_PATHWAY | mtu00930:Caprolactam degradation | 0.005 |
| moxifloxacin | KEGG_PATHWAY | mtu00281:Geraniol degradation | 0.006 |
| moxifloxacin | GOTERM_MF_DIRECT | GO:0042802~identical protein binding | 0.010 |
| moxifloxacin | GOTERM_MF_DIRECT | GO:0016853~isomerase activity | 0.010 |
| moxifloxacin | COG_ONTOLOGY | Lipid metabolism | 0.014 |
| moxifloxacin | KEGG_PATHWAY | mtu00410:beta-Alanine metabolism | 0.014 |
| moxifloxacin | KEGG_PATHWAY | mtu00627:Aminobenzoate degradation | 0.015 |
| moxifloxacin | KEGG_PATHWAY | mtu00903:Limonene and pinene degradation | 0.017 |
| moxifloxacin | UP_KEYWORDS | Aminotransferase | 0.021 |
| moxifloxacin | COG_ONTOLOGY | Signal transduction mechanisms | 0.037 |
| moxifloxacin | KEGG_PATHWAY | mtu00360:Phenylalanine metabolism | 0.045 |
| moxifloxacin | UP_KEYWORDS | Transferase | 0.048 |
| faropenem | GOTERM_BP_DIRECT | GO:0032259~methylation | 0.031 |
| faropenem | INTERPRO | IPR005814:Aminotransferase class-III | 0.044 |
| faropenem | KEGG_PATHWAY | mtu00970:Aminoacyl-tRNA biosynthesis | 0.044 |

Supplementary Table 7. Significant DAVID pathways of unique drug-specific genes for all six individual-drug blood cultures.

| Drug | DAVID database source | Pathway name | PValue |
| --- | --- | --- | --- |
| ethambutol | INTERPRO | IPR016192:APOBEC/CMP deaminase, zinc-binding | 0.0397 |
| isoniazid | INTERPRO | IPR006091:Acyl-CoA oxidase/dehydrogenase, central domain | 0.0315 |
| isoniazid | GOTERM_BP_DIRECT | GO:0033539~fatty acid beta-oxidation using acyl-CoA dehydrogenase | 0.0365 |
| isoniazid | GOTERM_BP_DIRECT | GO:0055088~lipid homeostasis | 0.0365 |
| isoniazid | UP_KEYWORDS | FAD | 0.0395 |
| isoniazid | INTERPRO | IPR013786:Acyl-CoA dehydrogenase/oxidase, N-terminal | 0.0407 |
| isoniazid | GOTERM_MF_DIRECT | GO:0050660~flavin adenine dinucleotide binding | 0.0423 |
| pyrazinamide | UP_KEYWORDS | Isopeptide bond | 0.0223 |
| pyrazinamide | UP_KEYWORDS | Ubl conjugation | 0.0223 |
| pyrazinamide | UP_KEYWORDS | Transferase | 0.0247 |
| pyrazinamide | UP_KEYWORDS | Oxidoreductase | 0.0414 |
| rifampicin | UP_KEYWORDS | Zinc-finger | 0.0091 |
| rifampicin | GOTERM_MF_DIRECT | GO:0008534~oxidized purine nucleobase lesion DNA N-glycosylase activity | 0.0212 |
| rifampicin | INTERPRO | IPR012319:DNA glycosylase/AP lyase, catalytic domain | 0.0319 |
| rifampicin | INTERPRO | IPR010663:Zinc finger, DNA glycosylase/AP lyase/isoleucyl tRNA synthetase | 0.0319 |
| rifampicin | INTERPRO | IPR015887:DNA glycosylase/AP lyase, zinc finger domain, DNA-binding site | 0.0319 |
| rifampicin | UP_SEQ_FEATURE | active site:Proton donor; for beta-elimination activity | 0.0364 |
| rifampicin | UP_SEQ_FEATURE | zinc finger region:FPG-type | 0.0364 |
| rifampicin | UP_SEQ_FEATURE | active site:Proton donor; for delta-elimination activity | 0.0364 |
| rifampicin | UP_SEQ_FEATURE | active site:Schiff-base intermediate with DNA | 0.0364 |
| rifampicin | INTERPRO | IPR015886:DNA glycosylase/AP lyase, H2TH DNA-binding | 0.0423 |
| rifampicin | INTERPRO | IPR000214:Zinc finger, DNA glycosylase/AP lyase-type | 0.0423 |
| rifampicin | UP_SEQ_FEATURE | binding site:DNA | 0.0482 |
| rifampicin | UP_SEQ_FEATURE | compositionally biased region:Asn-rich | 0.0482 |
| rifampicin | SMART | SM00898:SM00898 | 0.0493 |
| rifampicin | GOTERM_MF_DIRECT | GO:0005525~GTP binding | 0.0498 |

Supplementary Table 8. Unique drug-specific genes that are associated with the drug’s mechanism of action for each of the individual-drug blood cultures.

| Drug | Locus tag | Relevent GeneOntology pathway(s) |
| --- | --- | --- |
| isoniazid | *Rv0645* | mycolic acid biosynthesis |
| isoniazid | *Rv3139* | acyl CoA dehydrogenase and flavin adenine dinucleotide (FAD) binding pathways |
| isoniazid | *Rv1934c* | acyl CoA dehydrogenase and flavin adenine dinucleotide (FAD) binding pathways |
| isoniazid | *Rv1669* | acyl CoA dehydrogenase and flavin adenine dinucleotide (FAD) binding pathways |
| rifampicin | *Rv1112* | ribonucleoside binding pathway |
| rifampicin | *Rv0869c* | ribonucleoside binding pathway |
| rifampicin | *Rv2404c* | ribonucleoside binding pathway |
| ethambutol | *Rv2339* | integral components of membrane |
| ethambutol | *Rv0431* | integral components of membrane |
| ethambutol | *Rv1258c* | integral components of membrane |
| ethambutol | *Rv1226c* | integral components of membrane |
| ethambutol | *Rv2176* | integral components of membrane |
| ethambutol | *Rv3498c* | integral components of membrane |
| ethambutol | *Rv2219* | integral components of membrane |
| ethambutol | *Rv0107c* | integral components of membrane |
| moxifloxacin | *Rv3201c* | DNA-binding |
| pyrazinamide | *Rv2935* | phosphopantetheine binding |
| faropenem | *Rv350*1 | ATP-binding cassette (ABC) transporter |
